# Supplementary material for: Heterogeneity and Risk of Bias in Studies Examining Risk Factors for Severe Illness and Death in COVID-19: A Systematic Review and Meta-Analysis
Source: Pathogens. 2022 May 10;11(5):563. doi: 10.3390/pathogens11050563 (PMC9147100; doi:10.3390/pathogens11050563)
Supplement: Supplementary file 1 [file pathogens-11-00563-s001.zip › Table S10.pdf]

Heterogeneity and risk of bias in studies examining risk factors for severe illness and death in COVID-19: a systematic review and meta-analysis

Abraham Degarege *, Zaeema Naveed, Josiane Kabayundo, David Brett-Major

Table S10: Forest plot showing the relationship of chronic respiratory disease with the odds of death among COVID-19 patients

Author, year	OR	LCI	UCI
Acar et al., 2020	1.71	0.85	3.41
Alberici et al., 2020	1.07	0.22	4.20
Alser et al., 2021	5.06	1.83	14.00
Altschul et al., 2020	1.46	1.06	2.02
Anaklı et al., 2021	1.40	0.54	3.66
Angelidi et al., 2021	6.69	1.10	40.89
Aoun et al., 2021	2.03	0.85	4.86
Atkins et al., 2020	1.91	1.10	3.32
Attauabi et al., 2021	3.96	0.85	18.71
Auld et al., 2020	0.83	0.20	2.07
Azar et al., 2020	7.44	3.30	16.77
Baggio et al., 2021	1.55	1.13	2.14
Baqui et al., 2020	2.52	2.02	3.12
Barbui et al., 2021	1.49	0.61	3.64
Bartoletti et al., 2020	1.88	1.16	3.90
Berenguer et al., 2020	2.35	1.98	2.77
Bhatt et al., 2021	1.20	1.07	1.33
Bielza et al., 2021	0.66	0.39	1.12
Botta et al., 2021	1.70	0.86	3.36
Brandão et al., 2021	5.39	1.84	15.79
Cabezudo-García et al., 2020	1.58	0.85	2.95
Caillard et al., 2020	0.90	0.47	1.71
Caliskan et al., 2020	3.21	1.22	8.43
Capdevila-Reniu et al., 2021	0.58	0.18	1.88
Castelnuovo et al., 2020	2.30	1.87	2.82
Catteau et al., 2020	1.49	1.31	1.70
Cen et al., 2020	2.25	0.76	6.59
Chen et al., 2021	1.72	0.80	3.71
Chen L et al., 2020	4.02	1.40	11.53
Chen Y et al., 2020	4.38	1.74	11.03
Chilimuri et al., 2020	1.22	0.71	2.11
Choron et al., 2021	1.12	0.31	4.12
Ciceri et al., 2020	10.43	3.95	27.52
Cinar et al., 2021	4.95	2.08	11.80
Covino et al., 2020	3.02	0.61	14.81
Covino et al., 2020	2.38	0.64	8.77
Covino et al., 2020	2.27	0.50	10.21
Covino et al., 2021	1.98	0.71	5.58
De Smet et al., 2020	3.61	1.18	11.08
De Souza et al., 2020	1.27	0.87	1.86
Deeb et al., 2021	3.41	1.72	6.78

Deng et al., 2020	2.91	2.02	4.18
Du et al., 2020	2.54	0.71	9.08
Duarte et al., 2020	1.03	0.47	2.25
Elhadi et al., 2021	0.80	0.41	1.59
Escosteguy et al., 2021	0.15	0.04	0.63
Falcone et al., 2020	4.43	2.19	8.96
Fava et al., 2020	6.48	2.08	20.20
Ferrando et al., 2020	1.07	0.48	2.42
Fox et al., 2020	1.15	0.38	3.48
Foy et al., 2020	16.81	5.44	51.98
Galiero et al., 2020	2.55	1.61	4.04
Genet et al., 2020	0.97	0.43	2.19
Grasselli et al., 2020	2.82	1.78	4.45
Guan et al., 2020	11.53	4.36	30.45
Gupta et al., 2020	1.39	0.95	2.04
Haase et al., 2020	2.15	1.21	3.81
Halvatsiotis et al., 2020	0.81	0.15	4.27
Haq et al., 2020	4.22	1.42	12.55
Hatami et al., 2021	2.09	0.63	6.92
Hendra et al., 2021	1.01	0.21	18.74
Hu et al.,2020	4.00	1.11	15.50
Huang Y et al., 2020	8.40	2.48	28.37
Hwang et al., 2020	8.92	1.61	49.35
Iaccarino et al., 2020	2.43	1.54	3.83
Imam Z et al., 2020	1.86	1.30	2.64
Incerti et al., 2021	3.08	2.78	3.42
Inciardi et al., 2020	4.26	0.70	25.93
Islam et al., 2020	8.14	3.53	18.74
Jimeno et al., 2020	3.70	1.40	9.80
Jung et al., 2020	1.30	0.79	2.15
Kaeuffer et al., 2020	0.90	0.50	1.50
Kim S et al., 2020	2.26	0.86	5.97
Krishnamoorthy et al., 2021	1.37	0.76	2.47
Laake et al., 2020	2.26	0.97	5.27
Lee L et al., 2020	1.80	1.00	3.27
Lee Y et al., 2020	4.52	1.85	11.02
Li et al., 2020	10.86	1.15	102.7
Li et al., 2020	10.86	1.15	102.7
Li G et al., 2021	2.45	0.64	9.35
Lievre et al., 2020	2.15	1.48	3.14
Liu et al., 2020	4.14	0.94	18.22
Liu et al., 2020	0.83	0.24	2.85
Llamas et al., 2021	1.42	0.92	2.20
Lohia et al., 2021	1.36	1.08	1.72
Lorente et al., 2021	2.44	1.02	11.58

Lorente et al., 2021	2.18	0.20	23.36
Lunski et al., 2021	1.34	0.96	1.88
Maguire et al., 2020	1.48	0.69	3.16
Martinez-Lopez et al., 2020	1.33	0.54	3.29
Martins et al., 2021	1.52	0.26	9.03
Mash et al., 2020	1.34	0.59	3.03
Mato et al., 2020	6.70	2.47	18.18
Mayer et al., 2021	1.18	0.92	1.51
Mehra et al., 2020	2.88	1.96	4.23
Mehta et al., 2021	1.02	0.99	1.05
Mendes et al., 2020	0.98	0.40	2.39
Morrison et al., 2020	0.31	0.03	2.89
Mostaza et al., 2020	0.72	0.42	1.22
Nachega et al., 2020	1.39	0.47	4.11
Nachtigall et al., 2020	2.12	1.52	2.97
Nakamura et al., 2020	2.11	0.25	17.49
Namendys-Silva, 2021	2.85	0.29	28.02
Nepogodiev et al., 2020	1.85	1.23	2.78
Nikpouraghdam et al., 2020	2.05	0.99	4.22
Otero et al., 2021	0.91	0.22	3.77
Ozdemir et al., 2021	2.47	1.17	5.18
Ozturk ey al., 2020	2.10	1.38	3.21
Park et al., 2020	1.05	0.26	4.30
Park et al., 2020	4.13	1.08	15.85
Parlak et al., 2021	1.17	0.22	6.16
Passamonti et al., 2020	1.39	0.74	2.61
Patel et al., 2020	1.22	0.77	1.91
Peiro et al., 2021	3.76	1.66	8.56
Pineiro et al., 2021	3.08	0.72	13.30
Plotnikow et al., 2020	0.81	0.07	9.01
Qin W et al., 2021	2.52	1.20	5.29
Quisi et al., 2020	5.05	2.08	12.25
Raimondi et al., 2021	1.98	1.04	3.97
Rokni et al., 2020	3.50	1.13	10.82
Rosenthal et al., 2020	1.16	1.08	1.26
Salazer et al., 2021	1.26	0.98	1.63
Shahriarirad et al., 2020	1.50	0.17	13.54
Shi et al., 2020	2.40	0.68	8.51
Smith et al., 2020	2.70	1.52	4.79
Soleimani et al., 2020	2.50	1.03	6.09
Tessitore et al., 2021	2.00	0.94	4.30
Tu et al., 2020	4.05	1.00	16.33
Turgutalp et al., 2021	2.17	1.24	3.79
Venturini et al., 2021	0.84	0.17	4.15
Vrillon et al., 2021	1.16	0.12	11.61

Vrillon et al., 2021	3.30	0.92	11.76
Wang et al., 2020	5.37	2.17	13.29
Wang Junhong et al., 2020	4.34	1.34	14.04
Wu F et al., 2020	7.56	3.67	15.57
Xu Jiqian et al., 2020	1.93	0.51	7.34
Yan et al., 2020	3.09	0.83	11.48
Yang C et al., 2021	5.50	0.98	30.92
Zhou S et al., 2020	10.22	1.04	100.51
Zhou Wei et al., 2021	1.35	0.29	6.20
Zou X. et al., 2020	4.30	1.03	17.97
**Total (I squared=72.5%, p<0.01)**	**1.55**	**1.42**	**1.68**
