# Supplementary material for: Heterogeneity and Risk of Bias in Studies Examining Risk Factors for Severe Illness and Death in COVID-19: A Systematic Review and Meta-Analysis
Source: Pathogens. 2022 May 10;11(5):563. doi: 10.3390/pathogens11050563 (PMC9147100; doi:10.3390/pathogens11050563)
Supplement: Supplementary file 1 [file pathogens-11-00563-s001.zip › Table S11.pdf]

Heterogeneity and risk of bias in studies examining risk factors for severe illness and death in COVID-19: a systematic review and meta-analysis

Abraham Degarege *, Zaeema Naveed, Josiane Kabayundo, David Brett-Major

Table S11: Forest plot showing the relationship of cancer with the odds of death among COVID-19 patients

Author, year	OR	LCI	UCI	% Weight
Acar et al., 2020	11.96	6.34	22.54	0.07
Akhtar et al., 2021	1.92	1.04	3.55	1.56
Alberici et al., 2020	1.5	0.36	5.4	0.58
Anaklı etal., 2021	2.64	0.8	8.69	0.26
Aoun et al., 2021	1.81	0.68	4.76	0.82
Attauabi et al., 2021	3.18	0.8	12.78	0.12
Azar et al., 2020	5.18	2.44	11	0.23
Bernard et al., 2021	2.7	2.5	2.8	3.40
Bhatt et al., 2021	1.33	1.02	1.74	3.14
Bottio et al., 2021	1.67	0.25	11.26	0.14
Brandão et al., 2021	2.96	1.53	5.73	0.78
Cai et al., 2021	1.93	0.49	7.49	0.33
Caillard et al., 2020	0.85	0.41	1.81	2.50
Capdevila-Reniu et al., 2021	2.01	0.79	5.08	0.76
Castelnuovo et al., 2020	2.46	1.96	3.1	2.76
Catteau et al., 2020	1.08	0.8	1.46	3.19
Chand et al., 2020	2.49	0.86	7.17	0.40
Chang et al., 2020	2.18	0.54	8.76	0.25
Chen et al., 2021	3.17	1.84	5.46	0.98
Ciceri et al., 2020	3.62	1.51	8.63	0.32
Covino et al., 2020	1	0.08	11.64	0.13
Covino et al., 2020	2.05	0.43	9.72	0.20
Covino et al., 2021	1	0.27	3.71	1.05
De Souza et al., 2021	3.49	0.99	12.23	0.14
Deeb et al., 2021	5.06	1.86	13.79	0.12
Deng et al., 2020	2.54	1.11	5.81	0.65
Du et al., 2020	2.58	0.25	26.04	0.03
Du et al., 2020	1.44	0.37	5.61	0.55
Elhadi et al., 2021	3.41	0.97	11.92	0.14
Escosteguy et al., 2021	2.58	1.48	4.52	1.24
Falcone etal., 2020	1.38	0.67	2.84	1.81
Fava et al., 2020	5.28	1.17	23.85	0.03
Ferrando et al., 2020	1.86	0.36	9.67	0.19
Galiero et al., 2020	2.62	1.21	5.68	0.71
Genet et al., 2020	1.59	0.76	3.34	1.51
Goodall et al., 2020	1.75	0.97	3.14	1.81
Grasselli et al., 2020	1.75	1.39	2.21	3.06
Guan et al., 2020	5.4	1.53	18.99	0.06
Hwang et al., 2020	2.62	0.64	10.64	0.17
Imam Z et al., 2020	1.84	1.09	3.11	1.93
Incerti et al., 2021	1.78	1.58	2.02	3.33

Inciardi et al., 2020	2.72	0.75	9.82	0.20
Kim E et al., 2021	2.16	0.57	8.13	0.29
Kim S et al., 2020	4.06	1.35	10.98	0.18
Krause et al., 2020	5.75	1.16	28.44	0.02
Li et al., 2021	1.28	0.51	3.16	1.46
Liu et al., 2020	1.89	0.38	9.51	0.20
Lunski et al., 2021	2.03	1.44	2.87	2.48
Maguire et al., 2020	1.69	0.41	7.02	0.37
Martins et al., 2021	7.6	1.75	32.93	0.02
Mash et al., 2020	7.45	1.87	29.89	0.02
Mayer et al., 2021	1.19	0.99	1.43	3.33
Mehta et al., 2021	1.2	1.14	1.26	3.45
Menager et al., 2021	4.35	1	18.98	0.05
Molinero et al., 2020	4.36	0.45	42.37	0.01
Molinero et al., 2020	1.41	0.49	3.49	1.26
Nachega et al., 2020	5.11	0.84	30.97	0.02
Nachtigall et al., 2020	2.36	1.49	3.74	1.75
Nepogodiev et al., 2020	0.91	0.63	1.32	3.16
Ng et al., 2020	1.3	1.07	1.57	3.30
Okoh et al., 2020	2.72	1.11	6.65	0.50
Otero et al., 2021	0.24	0.02	2.02	1.95
Ozturk et al., 2020	1.57	0.81	3.04	1.76
Park et al., 2020	2.12	0.94	4.76	0.90
Patel et al., 2020	1.36	0.71	2.58	2.06
Ramos-Rincon et al., 2020	1.21	0.97	1.51	3.27
Rosenthal et al., 2020	1.27	1.09	1.47	3.36
Rossi et al., 2020	1.4	0.77	2.54	2.15
Ruthrich et al., 2021	0.56	0.44	0.72	3.40
Sai et al., 2021	1.49	0.22	9.99	0.18
Smith et al.,2020	1.93	1.06	3.53	1.58
Stefan et al., 2020	4.14	0.22	74.7	0.00
Talavera et al., 2020	1.4	0.76	2.56	2.12
Tian et al., 2020	2.04	1.34	3.13	2.13
Turgutalp et al., 2021	1.29	0.51	3.26	1.40
Vrillon et al., 2021	0.29	0.04	2.35	1.70
Wang et al.,2020	1.05	0.28	3.85	1.00
Xu Jingyuan. et al., 2020	0.38	0.09	1.55	2.45
Xu Jiqian et al., 2020	3.64	0.79	16.81	0.07
Xu Peng et al., 2020	2.54	0.31	20.95	0.04
Yarza et al., 2020	2.35	0.58	9.51	0.21
Yildirim et al., 2021	0.14	0	4.46	0.71
Zhao Y et al., 2020	3.23	1.39	7.52	0.42
Zhou Wei et al., 2021	0.82	0.24	2.8	1.52
Zhu Y et al., 2021	1.51	0.66	3.46	1.37
Zou X et al., 2020	0.48	0.05	4.41	0.74

**Total (I squared=85.1%, p<0.01)**	**1.55**	**1.34**	**1.76**	**100.00**
