# Supplementary material for: Heterogeneity and Risk of Bias in Studies Examining Risk Factors for Severe Illness and Death in COVID-19: A Systematic Review and Meta-Analysis
Source: Pathogens. 2022 May 10;11(5):563. doi: 10.3390/pathogens11050563 (PMC9147100; doi:10.3390/pathogens11050563)
Supplement: Supplementary file 1 [file pathogens-11-00563-s001.zip › Table S12.pdf]

Heterogeneity and risk of bias in studies examining risk factors for severe illness and death in COVID-19: a systematic review and meta-analysis

Abraham Degarege *, Zaeema Naveed, Josiane Kabayundo, David Brett-Major

Table S12: Forest plot showing the relationship of chronic kidney disease with the odds of death among COVID-19 patients

Author, year	OR	LCI	UCI	% Weight
Acar et al., 2020	5.56	2.53	12.21	0.14
Akhtar et al., 2021	0.77	0.42	1.42	2.08
Albalawi et al., 2021	0.80	0.21	3.06	1.01
Alguwaihes et al., 2021	3.50	1.40	8.70	0.24
Anaklı etal., 2021	1.54	0.37	6.34	0.34
Alser et al., 2021	2.17	0.66	9.83	0.16
Atkins et al., 2020	0.88	0.35	2.24	1.51
Attauabi et al., 2021	3.63	0.76	13.36	0.09
Auld et al., 2020	2.68	1.39	5.17	0.70
Baggio et al., 2021	2.55	1.73	3.75	1.43
Baqui et al., 2020	3.74	2.97	4.73	1.59
Barbui et al., 2021	3.22	1.22	8.49	0.24
Bartoletti et al., 2020	0.75	0.28	2.01	1.61
Becerra-Munoz et al., 2021	3.36	1.00	11.33	0.12
Berenguer et al., 2020	3.68	2.76	4.92	1.35
Bertsimas et al., 2020	2.48	1.55	3.98	1.21
Betech et al., 2020	2.00	1.84	2.17	2.40
Bhatt et al., 2021	1.45	1.29	1.62	2.40
Bielza et al., 2021	1.39	0.84	2.32	1.77
Botta et al., 2021	0.89	0.30	2.61	1.27
Caliskan et al., 2020	3.98	0.93	16.89	0.05
Capdevila-Reniu et al., 2021	1.40	0.67	2.93	1.29
Catteau et al., 2020	2.46	2.16	2.81	2.28
Cen et al., 2020	3.86	0.83	17.85	0.05
Chand et al., 2020	1.21	0.61	2.37	1.59
Chang et al., 2020	0.40	0.11	1.53	1.81
Chen et al., 2021	2.85	1.42	5.73	0.58
Chen L et al., 2020	2.65	0.96	7.28	0.31
Chen Y et al., 2020	13.91	7.23	26.8	0.04
Chilimuri et al., 2020	1.33	0.63	2.77	1.36
Choron et al., 2021	0.59	0.17	1.99	1.55
Ciceri et al., 2020	4.29	2.29	8.04	0.36
Cinar et al., 2021	7.34	2.17	28.84	0.02
Covino et al., 2020	4.89	1.21	19.69	0.04
De Souza et al., 2020	2.02	1.27	3.20	1.48
De Souza et al., 2021	1.14	0.41	3.19	1.04
Du et al., 2020	4.21	1.65	10.72	0.16
Elhadi et al., 2021	1.51	0.78	2.91	1.37
Falcone etal., 2020	3.76	1.67	8.44	0.27
Ferrando et al., 2020	0.91	0.22	3.69	0.79
Fox et al., 2020	1.44	0.61	3.40	1.04
Foy et al., 2020	13.96	6.12	31.88	0.02

Frontera et al., 2020	0.82	0.44	1.53	2.03
Galiero et al., 2020	1.01	0.44	2.34	1.50
Giannoglou et al., 2020	6.15	2.73	13.82	0.11
Grasselli et al., 2020	4.89	2.83	8.44	0.38
Guan et al., 2020	10.58	3.71	30.15	0.02
Gupta et al., 2020	1.75	1.36	2.26	2.14
Haase et al., 2020	1.95	0.98	3.86	1.00
Halvatsiotis et al., 2020	8.21	0.81	83.03	0.00
Haq et al., 2020	5.04	0.45	56.85	0.00
Hatami et al., 2021	1.07	0.51	2.28	1.58
Hwang et al., 2020	0.58	0.15	2.23	1.39
Iaccarino et al., 2020	1.58	0.14	2.43	1.28
Iaccarino et al., 2020	4.74	2.97	7.59	0.52
Imam Z et al., 2020	1.86	1.30	2.64	1.86
Incerti et al., 2021	3.09	2.81	3.42	2.30
Inciardi et al., 2020	6.44	1.74	23.84	0.03
Jung et al., 2020	2.35	1.25	4.40	0.90
Kaeuffer et al., 2020	2.30	1.30	3.90	1.12
Karahan etal., 2020	3.22	1.35	7.66	0.31
Kim E et al., 2021	4.10	1.03	16.41	0.06
Kim S et al., 2020	8.30	4.14	16.62	0.09
Krishnan et al., 2020	0.93	0.37	2.34	1.46
Laake et al., 2020	4.18	1.82	9.58	0.21
Liu et al., 2020	0.46	0.11	1.98	1.52
Llamas et al., 2021	4.13	2.22	7.69	0.39
Lucar et al., 2021	3.11	0.62	15.43	0.06
Lunski et al., 2021	3.84	2.98	4.93	1.47
Maguire et al., 2020	2.04	0.88	4.74	0.68
Martinez-Lopez et al., 2020	4.60	1.90	11.30	0.15
Martins et al., 2021	2.67	0.39	18.17	0.04
Mash et al., 2020	5.16	2.82	9.43	0.28
Mather et al., 2020	2.50	1.60	3.93	1.26
Mato et al., 2020	2.02	0.95	4.30	0.83
Mehta et al., 2021	1.24	1.20	1.29	2.44
Mendes et al., 2020	1.10	0.59	2.03	1.80
Molinero et al., 2020	4.64	2.57	8.34	0.36
Mostaza et al., 2020	0.80	0.45	1.22	2.22
Murthy et al., 2021	2.11	1.34	3.32	1.45
Nachega et al., 2020	10.43	2.30	47.31	0.01
Namendys-Silva, 2021	0.45	0.08	2.54	1.19
Nepogodiev et al., 2020	1.74	1.21	2.49	1.90
Nikpouraghdam et al., 2020	2.29	0.66	7.98	0.24
Okoh et al., 2020	3.43	1.78	6.80	0.45
Ozdemir et al., 2021	6.39	2.35	12.57	0.13
Park et al., 2020	2.19	0.85	5.63	0.49

Park et al., 2020	0.69	0.14	3.25	0.91
Parlak et al., 2021	34.83	4.11	295.58	0.00
Passamonti et al., 2020	2.45	1.30	4.64	0.83
Patel et al., 2020	2.29	1.30	4.01	1.07
Peiro et al., 2021	8.07	3.18	20.46	0.05
Pepe et al., 2021	2.47	1.78	3.42	1.67
Pineiro et al., 2021	6.86	0.76	61.67	0.00
Qin W et al., 2021	5.33	1.25	22.72	0.03
Raimondi et al., 2021	2.56	1.22	5.36	0.61
Recinella et al., 2020	0.78	0.30	2.03	1.61
Rodriguez et al., 2020	3.00	0.17	53.05	0.01
Rokni et al., 2020	2.96	0.74	11.87	0.11
Rossi et al., 2020	2.76	1.73	4.41	1.09
Rottoli et al., 2020	2.35	0.90	6.17	0.42
Russo et al., 2020	2.46	1.41	4.29	1.00
Sai et al., 2021	0.09	0.01	0.78	2.22
Saifi et al., 2021	0.45	0.11	1.85	1.60
Sands et al., 2020	0.96	0.71	1.29	2.31
Smith et al., 2020	1.89	1.14	2.80	1.65
Wang et al., 2020	1.93	0.57	6.47	0.35
Xu B. et al., 2020	2.98	0.52	17.11	0.05
Xu Peng et al., 2020	10.88	2.60	45.63	0.01
Y.Wang et al., 2020	0.49	0.11	2.24	1.37
Yarza et al., 2020	2.39	0.29	19.85	0.04
Yildirim et al., 2021	0.14	0.00	80.73	0.00
Zhang Nan et al., 2020	3.16	0.27	37.27	0.01
Zhao Y et al., 2020	10.39	2.07	52.13	0.01
Zhou S et al., 2020	10.22	1.04	100.51	0.00
Zhou Wei et al., 2021	0.66	0.11	4.04	0.66
Zhu Y et al., 2021	1.42	0.77	2.61	1.54
**Total (I squared=79.3%, p<0.01)**	**1.73**	**1.54**	**1.92**	100.00
