# Supplementary material for: Heterogeneity and Risk of Bias in Studies Examining Risk Factors for Severe Illness and Death in COVID-19: A Systematic Review and Meta-Analysis
Source: Pathogens. 2022 May 10;11(5):563. doi: 10.3390/pathogens11050563 (PMC9147100; doi:10.3390/pathogens11050563)
Supplement: Supplementary file 1 [file pathogens-11-00563-s001.zip › Table S13.pdf]

Heterogeneity and risk of bias in studies examining risk factors for severe illness and death in COVID-19: a systematic review and meta-analysis

Abraham Degarege *, Zaeema Naveed, Josiane Kabayundo, David Brett-Major

Table S13: Forest plot showing the relationship of smoking with the odds of death among COVID-19 patients

Author, year	OR	LCI	UCI	% Weight
Alguwaihes et al., 2021	1.70	0.20	20.00	0.04
Alser et al., 2021	2.76	1.50	5.17	0.80
Amanat et al., 2021	2.69	1.73	4.19	1.22
Anantharamna et al., 2020	1.15	0.76	1.73	1.89
Angelidi et al., 2021	1.26	0.23	7.01	0.32
Aoun et al., 2021	1.12	0.56	2.27	1.56
Attauabi et al., 2021	1.31	0.49	4.86	0.64
Berenguer et al., 2020	1.84	1.56	2.17	2.02
Bhatt et al., 2021	0.96	0.86	1.07	2.10
Bielza et al., 2021	0.90	0.31	2.63	1.28
Bottio et al., 2021	0.85	0.19	3.84	0.81
Brandão et al., 2021	1.02	0.67	1.55	1.93
Brenner et al., 2020	1.47	0.12	17.53	0.06
Caillard et al., 2020	1.25	0.56	2.82	1.30
Caliskan et al., 2020	13.01	5.05	33.48	0.02
Cariou et al., 2020	1.20	0.49	2.93	1.22
Castelnuovo et al., 2020	0.93	0.68	1.25	2.03
Cen et al., 2020	0.24	0.03	1.76	1.55
Chand et al., 2020	2.03	1.15	3.57	1.23
COVID-ICU group, 2020	1.18	0.85	1.62	1.97
Di Cosimo et al., 2021	1.68	0.74	3.70	1.02
Du H et al., 2020	0.59	0.13	2.74	1.15
Duclos et al, 2021	2.71	0.64	11.52	0.14
Ferrari et al., 2020	3.40	1.30	8.60	0.28
Galiero et al., 2020	1.35	0.84	2.16	1.74
Gu et al., 2020	1.67	1.06	2.20	1.82
Guarneri et al., 2021	0.75	0.29	1.91	1.60
Gupta et al., 2020	1.35	1.21	3.43	1.32
Halvatsiotis et al., 2020	1.87	0.71	4.95	0.66
Haq et al., 2020	1.79	0.88	3.62	1.10
Hu J et al., 2020	1.27	0.36	4.41	0.71
Imam Z et al., 2020	1.19	0.55	2.60	1.40
Incerti et al., 2021	1.28	1.16	1.41	2.09
Islam et al., 2020	0.85	0.28	2.51	1.32
Jimeno et al., 2020	2.40	0.60	9.60	0.19
Karahan et al., 2020	2.16	0.19	4.30	0.69
Kim E et al., 2021	1.40	0.61	3.17	1.17
Kim S et al., 2020	1.58	0.94	2.65	1.56
Krishnan et al., 2020	1.13	0.56	2.28	1.55
Li K et al., 2020	0.96	0.11	8.63	0.22
Lievre et al., 2020	2.03	1.59	2.59	1.88

Liu D et al., 2020	0.98	0.55	1.75	1.80
Liu Y et al., 2020	2.93	0.72	11.94	0.13
Llamas et al., 2021	1.47	0.97	2.24	1.76
Lohia et al., 2021	1.14	0.91	1.42	2.04
Lorente et al., 2021	1.61	0.16	16.08	0.07
Louapre et al., 2020	0.65	0.24	1.74	1.66
Lu Y et al., 2021	0.45	0.04	5.17	0.50
Lucar et al., 2021	5.85	1.20	28.57	0.02
Maguire et al., 2020	1.06	0.57	1.98	1.70
Mak et al., 2020	1.29	0.89	1.93	1.86
Marouf et al., 2021	5.07	1.71	15.07	0.09
Mash et al., 2020	1.01	0.46	2.21	1.54
Mato et al., 2020	1.07	0.57	1.99	1.69
Mayer et al., 2021	0.98	0.67	1.39	1.98
Mendes et al., 2020	0.93	0.52	1.65	1.83
Molinero et al., 2020	0.228	0.09	0.38	2.09
Murthy et al., 2021	1.87	0.92	3.81	1.05
Nakamura et al., 2020	0.60	0.13	2.67	1.18
Namendys-Silva, 2021	0.75	0.32	1.80	1.67
Nepogodiev et al., 2020	1.02	0.64	1.63	1.88
Nie et al., 2020	1.22	0.26	5.72	0.46
Otero et al., 2021	0.53	0.16	1.81	1.59
Ozdemir et al., 2021	0.99	0.54	1.80	1.77
Ozturk et al., 2020	0.93	0.65	1.33	2.00
Peiro et al., 2021	2.43	1.11	5.33	0.67
Pinato et al., 2020	1.64	0.88	3.04	1.35
Pongpirul et al., 2020	2.02	0.88	4.63	0.78
Quisi et al., 2020	4.28	2.14	8.58	0.35
Raimondi et al., 2021	2.17	1.31	3.58	1.30
Ramaswamy et al., 2020	1.12	1.07	1.18	2.11
Rajter et al., 2021	3.94	0.71	17.32	0.06
Rivera-Izquierdo et al., 2020	0.57	0.16	2.07	1.46
Singh et al., 2021	0.01	0.00	0.04	2.11
Smith et al., 2020	1.22	0.77	1.90	1.83
Stefan et al., 2020	4.48	0.54	36.92	0.01
Talavera et al., 2020	1.71	0.93	3.12	1.33
Tessitore et al. 2021	1.10	0.31	3.10	1.08
Webb et al., 2020	0.95	0.03	29.82	0.02
Yang C et al., 2021	1.89	0.74	4.87	0.69
Yao et al., 2021	1.22	0.12	12.34	0.11
Yarza et al., 2020	2.48	0.50	12.54	0.11
Zhang N et al., 2020	0.66	0.19	2.30	1.37
Zhou S et al., 2020	3.76	1.47	9.63	0.23
Zhou W et al., 2021	2.15	0.77	5.99	0.49
Zou X et al., 2020	1.77	0.56	5.57	0.52

**Total (I squared=96.6%, p<0.01)**	**1.22**	**1.01**	**1.43**	**100.00**
