# Supplementary material for: Heterogeneity and Risk of Bias in Studies Examining Risk Factors for Severe Illness and Death in COVID-19: A Systematic Review and Meta-Analysis
Source: Pathogens. 2022 May 10;11(5):563. doi: 10.3390/pathogens11050563 (PMC9147100; doi:10.3390/pathogens11050563)
Supplement: Supplementary file 1 [file pathogens-11-00563-s001.zip › Table S14.pdf]

Heterogeneity and risk of bias in studies examining risk factors for severe illness and death in COVID-19: a systematic review and meta-analysis

Abraham Degarege *, Zaeema Naveed, Josiane Kabayundo, David Brett-Major

Table S14: Forest plot showing the relationship of age with the odds of death among COVID-19 patients

Author, year	OR	LCI	UCI	% Weight
**Age in years ≥60 vs <60**				
Alahmari et al., 2021	9.18	8.4	10.03	3.10
Alguwaihes et al., 2021	1.4	0.63	3.1	3.01
Allameh et al., 2020	3.72	2.14	6.43	2.72
Alser et al., 2021	4.18	2.13	8.17	2.38
Altschul et al., 2020	2.4	1.18	5.16	2.78
Amanat et al., 2021	8.79	4.94	15.62	1.56
Azar et al., 2020	14.17	5.98	33.55	0.40
Baggio et al., 2021	11.87	10.5	13.36	2.96
Barbui et al., 2021	11.05	2.55	47.81	0.16
Berenguer et al., 2020	7.53	6.03	9.41	2.88
Bossini et al., 2020	1.12	1.03	1.24	3.17
Caillard et al., 2020	3.93	2.27	6.79	2.68
Chand et al., 2020	2.15	1.35	3.42	3.06
Dagher et al., 2021	2.46	1.35	6.63	2.53
Deng G et al., 2020	9.98	8.53	11.69	2.91
Del Amo et al., 2020	4.64	1.81	11.98	1.64
Di Cosimo et al., 2021	2.92	1.14	7.5	2.32
Elhadi et al., 2021	2.28	1.53	3.39	3.08
Ferrari et al., 2020	3.1	1.1	9.7	1.90
Grasselli et al., 2020	3.29	2.52	4.3	3.09
Gupta et al., 2020	2.91	2.41	3.51	3.14
Haq et al., 2020	2.7	0.5	14	1.19
Hatami et al., 2021	2.68	1.74	4.14	3.02
Imam Z et al., 2020	1.93	1.26	2.94	3.10
Islam et al., 2020	7.55	3.19	17.92	1.07
Jegede et al., 2020	7.93	2.91	21.59	0.76
Krause et al., 2020	9.45	1.43	62.31	0.09
Long et al., 2020	2.66	0.83	8.47	2.07

Martinez-Lopez et al., 2020	3	1.4	8.4	2.20
Murthy et al., 2021	3.69	2.37	5.74	2.88
Nachega et al., 2020	6.86	4.43	10.64	2.35
Nikpouraghdam et al., 2020	3.09	2.33	4.09	3.09
Oto et al., 2021	2.74	0.81	9.27	1.92
Passamonti et al., 2020	3.17	2.01	4.98	2.94
Pineiro et al., 2021	4.16	0.65	26.6	0.45
Pongpirul et al., 2020	3.24	1.24	8.48	2.15
Ramaswamy et al., 2020	1.9	0.66	5.65	2.59
Richardson et al., 2020	4.14	3.27	5.24	3.07
Rivera-Izquierdo et al., 2020	6.53	1.88	22.7	0.64
Verity et al., 2020	9.98	8.53	11.69	2.91
Wang D et al., 2020	18.12	4.79	68.57	0.08
Yang K et al., 2020	0.97	0.48	1.96	3.11
Zhang S et al.,2020	4.63	2.99	7.18	2.74
Zhao M et al., 2020	4.11	2.58	6.56	2.78
Zou X et al., 2020	6.44	2.76	15.03	1.34
**Total (I squared=95.7%, p<0.01)**	**4.32**	**3.39**	**5.25**	100.00
**Age in years ≥ 65 *vs* <65**				
Acar et al., 2020	4.77	2.9	7.86	2.51
Albalawi et al., 2021	5.2	1.74	15.49	0.88
Aliberti et al., 2021	2.27	1.82	2.85	3.42
Almaghlouth et al., 2020	3.2	0.58	17.6	0.63
Attauabi et al., 2021	4.82	1.22	24.22	0.38
Auld et al., 2020	4.06	2.12	7.77	2.32
Betech et al., 2020	1.28	0.84	1.94	3.41
Bezzio et al., 2020	19.6	2.95	130.6	0.01
Castelnuovo et al., 2020	8.71	6.84	11.09	2.71
Catteau et al., 2020	7.74	6.63	9.04	3.19
Cen et al., 2020	6.62	3.14	13.97	1.23
Ciceri et al., 2020	6.08	3.47	10.65	1.93
De Souza et al., 2021	4.06	2.72	6.05	2.96
Du et al., 2020b	3.76	1.14	17.39	0.68
Duarte et al., 2020	1.3	0.93	1.82	3.43
Giannoglou et al., 2020	8.22	3.88	17.42	0.90
Grasselli et al., 2020	3.49	3.07	3.98	3.43
Gu et al., 2020	8.77	5.34	14.41	1.52
Halvatsiotis et al., 2020	0.65	0.25	1.62	3.38
Kim E et al., 2021	1.88	0.81	4.39	2.90
Laake et al., 2020	3.44	1.99	5.96	2.79
Lee Y et al., 2020	4.96	2.65	9.3	2.06
Li K et al., 2020	4.17	1.34	13.02	1.11
Lievre et al., 2020	1.68	1.3	2.16	3.44
Lucar et al., 2021	0.59	0.11	3.24	3.02
Lunski et al., 2021	5.96	4.71	7.54	3.09
Mato et al., 2020	2.05	1.05	4.02	3.06

Pascual et al., 2020	6	1.02	35.38	0.18
Pinato et al., 2020	3.07	1.5	6.25	2.57
Rastad et al., 2021	1.62	0.6	4.41	2.84
Ren et al., 2020	20.96	3.69	119.16	0.02
Rosenthal et al., 2020	8.07	7.62	8.55	3.43
Santos et al., 2020	1.08	0.1	11.76	1.12
Shahriarirad et al., 2020	1.58	0.37	6.78	2.12
Wang Sufei et al., 2020	3.61	2.37	5.5	3.02
Wang Tao et al., 2020	1.03	0.98	1.07	3.48
Xu Jiqian et al., 2020	2.61	1.51	4.49	3.05
Yang D et al., 2020	3.26	1.79	5.94	2.74
Yang R et al., 2020	5.91	2.46	14.19	1.11
Yao et al., 2020	7.72	2.11	28.18	0.30
Yarza et al., 2020	1.59	0.5	4.99	2.65
Ye et al., 2020	9.95	5.76	17.29	1.13
Yu, Caizheng et al., 2020	2.11	1.39	3.21	3.31
Zeng H. et al., 2020	2.62	1.57	4.38	3.10
**Total (I squared= 96.7%, p<0.01)**	**3.51**	**2.76**	**4.26**	100.00
**Age in years continuous**				
Akhtar et al., 2021	1.11	1.07	1.16	1.99
Aloisio et al., 2020	1.14	1.03	1.27	0.55
Angelidi et al., 2021	1.81	1.05	3.13	0.01
Angelis et al., 2020	1.06	1.02	1.09	2.39
Annweiler et al., 2020	1.05	0.88	1.25	0.26
Bartoletti et al., 2020	1.06	1.03	1.09	2.61
Bianchetti et al., 2020	1.09	1.07	1.12	2.82
Bielza et al., 2021	1.01	0.98	1.03	2.82
Botta et al., 2021	2.19	1.65	2.9	0.02
Brenner et al., 2020	1.07	1.03	1.11	2.19
Brouns et al., 2020	1.04	0.39	2.77	0.01
Cabezudo-García et al., 2020	1.07	1.04	1.09	2.82
Caliskan et al., 2020	1.08	1.05	1.11	2.61
De Smet D et al., 2021	1.09	1.03	1.14	1.65
De Smet R et al., 2020	1.13	1.02	1.33	0.35
Duclos et al, 2021	1.23	1.06	1.43	0.26
Ferrando et al., 2020	1.05	1.01	1.09	2.19
Fox et al., 2020	1.03	1	1.07	2.39
Frontera et al., 2020	1.05	1.03	1.07	3.02
Galiero et al., 2020	1.05	1.02	1.07	2.82
Gomez et al., 2021	1.05	1.04	1.07	3.20
González-Gancedo et al., 2021	1.09	1.07	1.19	1.50
Guarneri et al., 2021	1.03	1	1.05	2.82
Hashemi et al., 2020	1.08	1.05	1.12	2.39
Hendra et al., 2021	1.05	1	1.1	1.81
Huang S et al., 2020	1.04	1.02	1.06	3.02
Jose et al., 2021	1.04	1.02	1.06	3.02

Karahan etal., 2020	1.01	0.06	1.03	0.04
Krishnamoorthy et al., 2021	1.06	1.04	1.08	3.02
Krishnan et al., 2020	1.08	1.04	1.12	2.19
Lee L et al., 2020	1.67	1.19	2.34	0.03
Lee S et al., 2020	1.06	1.05	1.07	3.34
Leung et al., 2020	1.04	1	1.1	1.81
Llamas et al., 2021	1.06	1.01	1.1	1.99
Mak et al., 2020	1.11	1.09	1.13	3.02
Mather et al., 2020	1.06	1.04	1.08	3.02
Messika et al., 2021	1.02	0.94	1.1	1.04
Molinero et al., 2020	1.08	1.05	1.12	2.39
Namendys-Silva, 2021	1.04	1.01	1.07	2.61
Okoh et al., 2020	1.04	1.01	1.06	2.82
Saleh et al., 2021	1.07	0.58	1.99	0.02
Sands et al., 2020	1.07	1.06	1.08	3.34
Sun et al., 2020	1.12	1.01	1.25	0.55
Talavera et al., 2020	1.06	1.03	1.08	2.82
Turgutalp et al., 2021	1.02	1	1.04	3.02
Wargny et al., 2021	1.84	1.49	2.27	0.06
Webb et al., 2020	1.06	1.01	1.11	1.81
Yang C et al., 2021	1.03	0.99	1.06	2.39
Yildirim et al., 2021	1.13	0.03	1.25	0.03
Zhao Y et al., 2020	0.92	0.88	0.94	2.61
Zhou S et al., 2020	1.09	1.03	1.15	1.50
Zhu Y et al., 2021	1.04	1.02	1.06	3.02
**Total (I squared=77.8%, p<0.01)**	**1.06**	**1.05**	**1.07**	100.00
**Age in years ≥ 50 vs <50**				
Aiswarya et al., 2020	1	0.24	4.02	51.00
Alasia et al., 2021	10.81	3.27	35.75	13.50
Nepogodiev et al., 2020	9.03	4.56	17.89	35.51
**Total (I squared= 68.7%, p=0.041)**	**5.18**	**-1.74**	**12.09**	100.00
**Age in years ≥ 75 vs <75**				
Adamuz et al., 2020	3.04	1.79	5.15	3.31
Becerra-Munoz et al., 2021	3.54	1.76	8.38	0.85
De Souza et al., 2020	2.4	2.1	2.74	91.13
Ling et al., 2020	1.71	0.8	3.7	4.44
Sano et al., 2020	1.65	0.23	11.99	0.27
**Total (I squared=0.0%, p=0.746)**	**2.40**	**2.09**	**2.70**	**100.00**
**Age in years ≥ 70 vs <70**				
Kim S et al., 2020	4.77	2.21	11.05	10.71
Maguire et al., 2020	4.49	2.33	8.64	16.08
Nakamura et al., 2020	2	0.41	9.74	9.94
Shi et al., 2020	5.28	1.83	15.21	5.70
Zhang Lin et al., 2020	12.81	5.7	28.8	2.16
Zhong Y et al., 2020	1.83	0.66	5.07	21.91
Zuo P et al., 2020	0.98	0.97	0.99	33.51

Chen J et al., 2020a	9.08	4.44	18.59	10.71
**Total (I squared= 57.0%, p=0.03)**	**2.74**	**0.98**	**4.492**	**100.00**
