# Supplementary material for: Heterogeneity and Risk of Bias in Studies Examining Risk Factors for Severe Illness and Death in COVID-19: A Systematic Review and Meta-Analysis
Source: Pathogens. 2022 May 10;11(5):563. doi: 10.3390/pathogens11050563 (PMC9147100; doi:10.3390/pathogens11050563)
Supplement: Supplementary file 1 [file pathogens-11-00563-s001.zip › Table S15.pdf]

Heterogeneity and risk of bias in studies examining risk factors for severe illness and death in COVID-19: a systematic review and meta-analysis

Abraham Degarege *, Zaeema Naveed, Josiane Kabayundo, David Brett-Major

Table S15. Forest plot showing the relationship of age with the odds of severe illness among COVID-19 patients

Author, year	OR	LCI	UCI	% Weight
**Age in years ≥60 vs <60**				
Allenbach et al., 2020	2.94	1.19	7.26	2.37
Amanat et al., 2021	1.63	1.23	2.16	11.32
Bhargava et al., 2020	1.84	1.02	3.31	7.75
Cheng et al., 2020	6.82	3.52	13.21	1.05
Dai et al., 2020	3.87	1.28	11.68	0.92
Hoffmann et al., 2021	1.85	0.79	4.31	5.12
Huang R et al., 2020	2.09	0.7	6.22	2.75
Li H et al., 2020	1.26	0.6	1.26	11.86
Lian et al., 2020	4.61	2.81	7.55	3.46
Nachega et al., 2020	4.76	3.31	6.85	5.09
Pereira et al., 2020	3.85	1.46	10.18	1.27
Rivinius et al., 2020	15.75	1.42	174.25	0.00
Tao et al., 2020	2.66	1.02	6.96	2.45
Wang J et al., 2020	6.21	2.11	18.23	0.40
Wang Juan et al., 2020	3.75	2.07	6.8	3.47
Wang X et al., 2020	2.1	1.36	3.25	8.81
Yu et al., 2020	10.79	3.94	29.53	0.16
Zhang G et al., 2021	0.54	0.23	1.28	11.05
Zhang Y et al., 2020	11.47	4.4	29.88	0.16
Zhao M et al., 2020	2.8	2.08	3.79	9.31
Zheng T et al., 2020	1.11	0.72	1.7	11.21
**Total (I squared=69.5%, p<0.01)**	**2.14**	**1.63**	**2.66**	100.00
**Age in years ≥ 65 *vs* <65**				
Algado-Sellés et al., 2020	2.9	0.9	9.3	1.43
Bezzio et al., 2020	5.87	1.15	29.66	0.15
Cen et al., 2020	5	3.66	6.83	4.99
Çalık Başaran N et al., 2021	4.15	1.64	10.51	1.30
Colaneri et al., 2020	4.06	1.04	15.72	0.53
Derikx et al., 2020	2.94	1.06	8.17	1.87
Du et al., 2020a	0.84	0.34	2.03	7.10
Etienne et al., 2020	0.92	0.15	5.5	2.82
Feng et al., 2020	2.17	1.39	3.4	6.64
Gao et al., 2020	2.72	1.21	6.12	3.16
Guan et al., 2020b	2.55	1.72	3.79	6.55
Hu L et al., 2020	2.96	1.81	4.83	5.19
Ji D et al., 2020	9.75	4.2	22.63	0.34
Jinag N et al., 2021	2.72	1.31	5.63	3.68
Khan et a., 2020	3.15	1.4	7.09	2.60
Lee Y et al., 2020	4.32	2.73	6.84	3.89
Li X et al., 2020	2.24	1.94	2.78	8.13
Liu J etal., 2021	1.09	1.05	1.46	8.43

Liu S et al., 2020	6.56	3.7	11.64	1.57
Ren et al., 2020	5.81	2.14	15.78	0.60
Scarfo et al., 2020	3.72	1.79	7.71	2.46
Shahriarirad et al., 2020	0.87	0.21	3.51	4.82
Sun L et al., 2020	21.7	3.78	124.5	0.01
Tian S et al,. 2020	5.16	2.55	10.45	1.58
Vee et al.,2020	3.8	0.74	19.58	0.33
Wang Fang et al., 2020	3.22	1.51	6.87	2.82
Wang Wen et al., 2021	1.92	0.5	6.24	2.57
Wei Z et al, 2021	1.68	0.55	5.07	3.49
Wu J et al., 2020	12.75	6.74	24.1	0.38
Xiong S et al., 2020	3.48	1.57	7.73	2.32
Zhao J et al., 2021	2.75	1.57	4.81	4.90
Zhou Yiwu et al., 2020	2.41	1.02	5.69	3.36
**Total (I squared=69.5%, p<0.01)**	2.63	2.08	3.18	100.00
**Age in years continuous**				
Annweiler et al., 2020	1.05	0.88	1.25	1.14
Fernandes et al., 2021	1.09	1.01	1.18	4.16
He et al., 2020	1.07	1.05	1.08	13.18
Huang S et al., 2020	1.05	0.68	2.33	0.06
Jain et al., 2021	1.01	0.98	1.04	10.91
Molinero et al., 2020	1.01	0.1	1.02	0.20
Nguyen et al., 2020	1.49	1.3	1.7	0.99
Stachura et al., 2021	1.1	1.01	1.15	5.39
Shao et al., 2020	1.06	1.02	1.086	10.40
Tian J et al., 2020	1.04	1	1.07	10.07
Wang F et al., 2021	1.07	1.03	1.1	10.07
Yu Y et al., 2020	1.03	1.02	1.05	13.18
Zhang R et al., 2020	1.1	1	1.1	7.74
Zhang Y et al., 2021	1.03	1.01	1.05	12.51
**Total (I squared= 74.7%, p<0.01)**	1.06	1.04	1.08	100.00
**Age in years ≥ 50 vs <50**				
Aiswarya et al., 2020	1.18	0.37	3.71	42.98
Alasia et al., 2021	10.42	5.9	18.42	20.62
Betti et al., 2021	3.23	1.42	7.37	36.40
**Total (I squared= 76.5%, p<0.01)**	3.83	0.03	7.63	100.00
**Age in years ≥ 75 vs <75**				
Adamuz et al., 2020	1.52	1.02	2.26	99.77
Wang Sijiao et al., 2020	5.61	1.16	27.12	0.23
**Total (I squared=** 0.0%, **p=0.537)**	1.53	0.91	2.15	100.00
**Age in years ≥ 70 vs <70**				
Jang et al., 2020	3.37	0.26	43.58	75.73
Zhang Lin et al., 2020	16.19	3.28	79.8	24.27
**Total (I squared=** 0.0**%, p= 0.568)**	6.48	-12.37	25.33	100.00
