# Supplementary material for: Heterogeneity and Risk of Bias in Studies Examining Risk Factors for Severe Illness and Death in COVID-19: A Systematic Review and Meta-Analysis
Source: Pathogens. 2022 May 10;11(5):563. doi: 10.3390/pathogens11050563 (PMC9147100; doi:10.3390/pathogens11050563)
Supplement: Supplementary file 1 [file pathogens-11-00563-s001.zip › Table S16.pdf]

Heterogeneity and risk of bias in studies examining risk factors for severe illness and death in COVID-19: a systematic review and meta-analysis

Abraham Degarege *, Zaeema Naveed, Josiane Kabayundo, David Brett-Major

Table S16: Forest plot showing the relationship of gender with the odds of death among COVID-19 patients

Author, year	OR	LCI	UCI	% Weight
Adamuz et al., 2020	1.86	1.11	3.11	0.27
Ahmad et al., 2020	0.38	0.14	1.05	0.61
Aiswarya et al., 2020	0.28	0.06	1.31	0.47
Akhtar et al., 2021	0.85	0.53	1.36	0.65
Alahmari et al., 2021	1.52	1.36	1.69	0.86
Alasia et al., 2021	4.44	0.57	34.41	0.00
Albalawi et al., 2021	0.48	0.2	1.16	0.59
Albani et al., 2020	1.62	1.23	2.12	0.62
Alberici et al., 2020	1.05	0.41	2.78	0.21
Alfraij et al., 2021	2.25	0.19	25.36	0.00
Alguwaihes et al., 2021	3.3	1.2	9.2	0.02
Aliberti et al., 2021	1.25	1.01	1.56	0.77
Allameh et al., 2020	1.39	0.82	2.35	0.38
Almaghlouth et al., 2020	0.27	0.05	1.53	0.39
Al-Rousan, 2020	1.14	0.97	1.35	0.84
Alser et al., 2021	2.49	0.89	7	0.04
Altschul et al., 2020	1.05	0.63	1.72	0.53
Amanat et al., 2021	1.36	0.9	2.06	0.50
Anakli etal., 2021	0.95	0.4	2.27	0.29
Anantharamna et al., 2020	1.72	1.17	2.56	0.42
Angelidi et al., 2021	2	0.47	8.63	0.02
Angelis et al., 2020	2.12	0.86	5.18	0.07
Aoun et al., 2021	1.16	0.63	2.14	0.38
Atkins et al., 2020	2.12	1.45	3.09	0.35
Auld et al., 2020	0.77	0.42	1.39	0.58
Aw et al., 2020	2.02	1.47	2.77	0.45
Azar et al., 2020	1.95	1.08	3.51	0.20
Azwar et al., 2020	6.3	0.71	55.5	0.00
Baggio et al., 2021	1.64	1.47	1.82	0.85
Baqui et al., 2020	1.12	1.03	1.23	0.89
Barbui et al., 2021	2.48	1.01	6.07	0.06
Bartoletti et al., 2020	1.43	0.72	2.83	0.25
Berenguer et al., 2020	1.57	1.35	1.82	0.81
Bertsimas et al., 2020	1.46	1.23	1.73	0.80
Betech et al., 2020	1.24	1.2	1.29	0.91
Bhatt et al., 2021	1.26	1.13	1.4	0.88
Bianchetti et al., 2020	1.15	0.78	1.66	0.62

Bielza et al., 2021	1.56	1.07	2.28	0.49
Botta et al., 2021	2.16	1.24	3.78	0.19
Bottio et al., 2021	1.92	0.43	6.09	0.04
Brenner et al., 2020	2.78	0.76	10.14	0.02
Brouns et al., 2020	4.79	1.74	13.18	0.01
Byeon et al., 2021	1.53	1.22	1.92	0.71
Cabezudo-García et al., 2020	1.39	0.79	2.44	0.35
Caillard et al., 2020	0.96	0.6	1.53	0.60
Capdevila-Reniu et al., 2021	1.52	0.78	2.94	0.24
Cariou et al., 2020	1.25	0.85	1.81	0.59
Carubbi et al., 2021	0.83	0.12	5.41	0.05
Castelnuovo et al., 2020	1.24	1.05	1.48	0.82
Catteau et al., 2020	1.15	1.04	1.28	0.88
Cen Y et al., 2020	2.81	1.42	5.53	0.08
Chand et al., 2020	1.83	1.14	2.92	0.31
Chang et al., 2020	2.02	0.84	4.82	0.09
Chen J et al., 2020	2.27	1.72	2.94	0.48
Chen L et al., 2020	0.81	0.49	1.34	0.64
Chen T et al., 2020	5.33	1.32	21.52	0.00
Chen Y et al., 2020	2.63	1.63	4.16	0.19
Chilimuri et al.,2020	1.37	0.79	2.37	0.36
Choron et al., 2021	2.26	1.01	2.61	0.36
Choudhuri et al., 2020	1.88	1.37	2.59	0.48
Ciceri et al., 2020	1.05	0.62	1.76	0.51
Cinar et al., 2021	3.94	1.56	9.95	0.02
COVID-ICU group, 2020	1.11	0.95	1.29	0.86
Covino et al., 2020a	0.91	0.33	2.49	0.24
Covino et al., 2020b	1.55	0.63	3.8	0.13
Covino et al., 2020c	1.41	0.51	3.83	0.12
Covino et al., 2021	1.05	0.52	2.13	0.36
De Smet D et al., 2020	1.02	0.37	2.84	0.19
De Smet R et al., 2020	1.07	0.38	3.05	0.17
De Souza et al., 2020	0.65	0.57	0.74	0.90
De Souza et al., 2021	1.79	1.25	2.58	0.44
Deng G et al, 2020	1.6	1.48	1.92	0.82
Del Amo et al., 2020	0.59	0.18	1.91	0.33
Di Cosimo et al., 2021	1.67	0.97	2.91	0.28
Du H et al., 2020	2.46	1.04	5.8	0.06
Du H et al.,2020	2.46	1.04	5.8	0.06
Duarte et al., 2020	0.84	0.61	1.15	0.78
Duclos et al, 2021	1.53	0.12	18.8	0.00
Elhadi et al., 2021	1.33	0.91	1.93	0.56
Escobar et al., 2021	1.93	1.69	2.19	0.80
Escosteguy et al., 2021	1.64	1.06	2.53	0.40
Falcone etal., 2020	2.13	1.14	2.98	0.30

Fava et al., 2020	0.79	0.33	1.9	0.37
Ferrando et al., 2020	0.93	0.65	1.32	0.72
Ferrando-Vivas et al., 2021	1.3	1.19	1.42	0.89
Ferrari et al., 2020	1.47	0.68	3.16	0.19
Fox et al., 2020	1.36	0.62	2.99	0.21
Foy et al., 2020	1.28	0.98	1.66	0.72
Frontera et al., 2020	0.79	0.53	1.18	0.73
Fu et al., 2020	1.14	0.36	3.56	0.13
Galiero et al., 2020	2.63	1.42	4.9	0.11
Giannoglou et al., 2020	1.46	0.88	2.43	0.37
Goicoechea et al., 2020	0.56	0.13	2.41	0.22
Gomez et al., 2021	1.96	1.34	2.9	0.37
Goodall et al., 2020	1.02	0.78	1.34	0.77
Grasselli et al., 2020	1.28	1.1	1.51	0.83
Gu et al., 2020	2.12	1.35	3.33	0.27
Guarneri et al., 2021	0.71	0.37	1.36	0.57
Guo et al., 2020	1.92	1.56	2.36	0.66
Gupta et al., 2020	1.5	1.19	1.9	0.70
Haase et al., 2020	1.71	0.99	2.96	0.27
Halvatsiotis et al., 2020	1.07	0.34	3.38	0.14
Haq et al., 2020	0.36	0.16	0.82	0.72
Hashemi et al., 2020	1.62	0.8	3.26	0.20
Hatami et al., 2021	1.48	0.98	2.24	0.47
Hendra et al., 2021	1.47	0.51	4.34	0.09
Hu H et al.,2020	2.21	0.73	6.7	0.04
Hu J et al., 2020	5.21	2.41	11.27	0.02
Huang S et al., 2020	2.1	1.12	3.94	0.16
Huang Y et al., 2020	6.5	1.41	29.9	0.00
Hwang et al., 2020	1.82	0.73	4.15	0.11
Iaccarino et al., 2020	1.87	1.41	2.3	0.62
Iaccarino et al., 2020	1.13	0.82	1.56	0.69
Incerti et al., 2021	1.28	1.17	1.41	0.88
Inciardi et al., 2020	1.82	0.33	10.07	0.02
Islam et al., 2020	1.79	0.71	4.54	0.09
Jegede et al., 2020	0.82	0.34	2	0.34
Jimeno et al., 2020	0.6	0.3	1.3	0.57
Jose et al., 2021	0.59	0.36	0.94	0.76
Jung et al., 2020	1.46	0.9	2.35	0.40
Kaeuffer et al., 2020	1.7	1.1	2.7	0.36
Karahan etal., 2020	1.46	0.76	2.81	0.26
Karimi et al., 2020	1.76	0.36	8.55	0.02
Karlsson et al., 2020	4	1.8	9.5	0.02
Kaya et al., 2021	1.53	0.44	5.38	0.06
Kim D et al., 2020	2.18	1.5	3.17	0.34
Kim E et al., 2021	0.91	0.42	1.97	0.37

Kim S et al., 2020	2.16	1.59	2.94	0.44
Krause et al., 2020	0.5	0.18	1.41	0.48
Krishnan et al., 2020	1.19	0.61	2.33	0.33
Kunal et al., 2020	0.91	0.38	2.2	0.30
Kute et al., 2021	1.47	0.42	5.13	0.06
Laake et al., 2020	0.67	0.38	1.19	0.66
Lee J et al., 2021	2.12	1.47	3.12	0.35
Lee L et al., 2020	1.67	1.19	2.34	0.51
Lee S et al., 2020	2.31	1.58	3.37	0.31
Leung et al., 2020	1.19	0.62	2.26	0.35
Li G et al., 2020	1.85	1.23	2.8	0.37
Li G et al., 2021	1.49	0.66	3.36	0.17
Li K et al., 2020	2.22	0.65	7.69	0.03
Lievre et al., 2020	1.61	1.25	2.08	0.65
Ling et al., 2020	0.68	0.33	1.37	0.55
Liu et al., 2020	1.51	0.94	2.45	0.38
Llamas et al., 2021	1.61	1.04	2.51	0.40
Long et al., 2020	0.77	0.31	1.92	0.36
Lorente L et al, 2021a	0.48	0.19	1.23	0.55
Lorente L et al., 2021b	0.52	0.12	2.2	0.25
Louapre et al., 2020	2.11	1.23	3.63	0.20
Lu Y et al., 2021	2.01	0.77	5.19	0.07
Lucar et al., 2021	2.05	0.55	7.52	0.03
Lunski et al., 2021	2.15	1.73	2.67	0.60
Maguire et al., 2020	1.55	0.82	2.93	0.25
Mak et al., 2020	1.48	1.13	1.94	0.66
Marengoni et al., 2020	3.08	0.87	12.8	0.01
Marouf et al., 2021	0.82	0.28	2.37	0.25
Martinez-Lopez et al., 2020	3.8	1.7	8.4	0.03
Martins et al., 2021	10.5	1.24	88.59	0.00
Mash et al., 2020	2.02	1.37	2.98	0.36
Mato et al., 2020	0.77	0.42	1.42	0.57
Mayer et al., 2021	2.22	1.9	2.6	0.71
Mehta et al., 2021	1.45	1.41	1.49	0.91
Mehta V et al., 2020	1.04	0.57	1.9	0.44
Menager et al., 2021	1.15	0.29	4.63	0.07
Mendes et al., 2020	3.33	1.88	5.89	0.09
Messika et al., 2021	1.31	0.19	9.02	0.02
Moghaddam et al., 2020	0.63	0.1	4.01	0.09
Molinero et al., 2020	0.99	0.61	1.64	0.56
Morrison et al., 2020	1.98	0.73	5.33	0.07
Mostaza et al., 2020	2.31	1.51	3.52	0.27
Murthy et al., 2021	0.76	0.54	1.08	0.78
Nachega et al., 2020	1.5	0.97	2.32	0.44
Nachtigall et al., 2020	1.15	0.9	1.47	0.76

Nakamura et al., 2020	7.5	0.81	69.7	0.00
Nepogodiev et al., 2020	1.79	1.35	2.39	0.55
Neto et al., 2021	0.9	0.61	1.32	0.70
Ng et al., 2020	1.39	1.23	1.58	0.85
Nie et al., 2020	6.19	0.71	54.16	0.00
Nikpouraghdam M et al., 2020	1.21	0.91	1.62	0.70
Okoh et al., 2020	1.99	1.19	3.37	0.24
Otero et al., 2021	0.97	0.37	2.54	0.24
Oto et al., 2021	0.5	0.16	1.55	0.42
Ozdemir et al., 2021	0.88	0.49	1.57	0.54
Ozturk ey al., 2020	1.57	1.12	2.19	0.54
Pascual et al., 2020	0.97	0.19	4.87	0.06
Park et al., 2020a	2.03	1.29	3.2	0.29
Park et al., 2020b	2.11	1.22	3.65	0.20
Parlak et al., 2021	1.06	0.42	2.67	0.23
Passamonti et al., 2020	1.39	0.96	2.01	0.55
Peiro et al., 2021	1.14	0.54	2.37	0.30
Pineiro et al., 2021	1.1	0.3	4.02	0.10
Pizzini et al., 2020	6.65	2.78	15.9	0.01
Plotnikow et al., 2020	0.55	0.06	5.35	0.05
Pongpirul et al., 2020	2.02	0.88	4.63	0.10
Qian et al., 2020	1.25	1.2	1.29	0.91
Qin et al., 2020	2.44	1.5	3.95	0.20
Qin W et al., 2021a	0.86	0.36	2.03	0.34
Qin W et al., 2021b	3.15	1.88	5.27	0.11
Quisi et al., 2020	0.72	0.36	1.45	0.53
Raimondi et al., 2021	1.05	0.47	2.37	0.29
Ramaswamy et al., 2020	1.12	0.47	2.68	0.23
Ramos-Rincon et al., 2020	1.27	1.01	1.59	0.76
Rastad et al., 2021	2.94	0.95	9.15	0.02
Rajter et al., 2021	2.38	0.59	4.16	0.10
Recinella et al., 2020	1.11	0.52	2.4	0.29
Richardson et al.,2020	1.23	1.01	1.49	0.80
Rivera-Izquierdo et al., 2020	1.49	0.82	2.71	0.29
Rodilla et al., 2021	1.6	1.43	1.75	0.86
Rodrigues et al.,2020	2.24	1.91	2.62	0.70
Rodriguez et al., 2020	0.56	0.13	2.39	0.22
Roedl et al., 2020	1.78	0.87	3.65	0.16
Rokni et al., 2020	1.47	0.62	3.51	0.15
Rosenthal et al., 2020	1.18	1.1	1.26	0.90
Rossi et al., 2020	1.56	1.09	2.22	0.52
Ruthrich et al., 2021	1.73	0.55	5.36	0.06
Saeed et al., 2020	1.34	1.12	1.59	0.81
Saez-Giménez et al., 2020	2.23	0.61	8.08	0.03
Sai et al., 2021	0.34	0.1	1.22	0.52

Salazer et al., 2021	0.97	0.84	1.11	0.88
Saleh et al., 2021	1.14	0.68	2.3	0.35
Santos et al., 2020	3.6	0.77	7	0.04
Shahriarirad et al., 2020	0.72	0.18	2.84	0.17
Sharma.,2020	1.02	0.92	1.12	0.89
Shi et al.,2020	0.65	0.24	1.79	0.37
Singh et al.,2021	1.91	1.3	2.83	0.38
Smith et al.,2020	1.13	0.72	1.78	0.54
Soleimani et al.,2020	0.86	0.49	1.5	0.57
Stefan et al., 2020	0.66	0.12	3.45	0.12
Sun et al.,2020	0.32	0.04	2.41	0.21
Tang et al., 2020	1.57	1.03	2.4	0.43
Tatum et al., 2020	1.41	0.57	3.57	0.14
Tejpal et al., 2021	1.37	1.23	1.52	0.87
Tessitore et al., 2021	1.6	0.98	2.6	0.35
Trifan et al., 2020	4.02	1.14	14.18	0.01
Tu Y et al., 2020	3.65	1.1	12.12	0.01
Turgutalp et al., 2021	1.33	0.85	2.08	0.48
Venturini et al., 2021	1.06	0.5	2.25	0.32
Vrillon et al., 2021	1.12	0.38	3.31	0.15
Vrotsou et al., 2021	1.94	1.68	2.25	0.76
Vu et al., 2020	4.75	0.55	40.98	0.00
Wang D et al., 2020	6.11	1.66	22.48	0.00
Wang Junhong et al., 2020	2.07	0.84	5.09	0.08
Wang L et al., 2020	1.73	1.00	3.00	0.27
Wang Tao et al., 2020	1.67	0.49	5.62	0.05
Wang Y et al., 2020	1.71	0.96	3.04	0.25
Wargny et al., 2021	1.01	0.7	1.45	0.68
Webb et al., 2020	0.79	0.24	2.62	0.21
Williams et al., 2021	1.77	1.73	1.8	0.91
Xu B et al., 2020	1.31	0.58	2.98	0.20
Xu Jingyuan et al., 2020	2.37	1.36	4.12	0.16
Xu Jiqian et al., 2020	1.16	0.68	1.97	0.46
Xu Peng et al., 2020	2.37	1.08	5.17	0.08
Xu Xunhua et al., 2020	0.91	0.21	4.01	0.09
Yan Y et al., 2020	2.93	1.62	5.32	0.10
Yang C et al., 2021	1.68	0.89	3.15	0.22
Yang D et al., 2020	2.77	1.54	5	0.11
Yang K et al., 2020	3.86	1.8	8.25	0.03
Yang R et al., 2020	6.16	2.03	18.65	0.01
Yang X et al., 2020	1.89	1.41	2.53	0.52
Yang X et al., 2020	1.9	1.42	2.53	0.52
Yao Q et al., 2020	2.33	0.68	7.9	0.03
Yarza et al., 2020	1.17	0.37	3.57	0.13
Ye et al., 2020	1.82	1.13	2.91	0.31

Yu, Caizheng (2020)	2.02	1.37	2.99	0.35
Zeng H et al., 2020	2.08	1.26	3.44	0.24
Zhang Nan et al., 2020	1.26	0.4	4.03	0.10
Zhao Y et al., 2020	1.64	1.1	2.46	0.43
Zhong Y et al., 2020	5.49	1.78	16.94	0.01
Zhou S et al., 2020	3.15	1.62	6.12	0.07
Zhou Wei et al., 2021	2.19	1.21	3.95	0.16
Zhu Y et al., 2021	0.94	0.6	1.47	0.63
Zou X et al., 2020	0.73	0.37	1.45	0.54
Zuo P et al., 2020	0.98	0.96	1	0.91
**Total (I squared= 89.3%, p<0.01)**	**1.32**	**1.26**	**1.39**	100.00
