# Supplementary material for: Heterogeneity and Risk of Bias in Studies Examining Risk Factors for Severe Illness and Death in COVID-19: A Systematic Review and Meta-Analysis
Source: Pathogens. 2022 May 10;11(5):563. doi: 10.3390/pathogens11050563 (PMC9147100; doi:10.3390/pathogens11050563)
Supplement: Supplementary file 1 [file pathogens-11-00563-s001.zip › Table S17.pdf]

# Heterogeneity and risk of bias in studies examining risk factors for severe illness and death in COVID-19: a systematic review and meta-analysis

Abraham Degarege *, Zaeema Naveed, Josiane Kabayundo, David Brett-Major

**Table S17: Forest plot showing the relationship of gender with the odds of severe illness among COVID-19 patients**

| Author, year | OR | LCI | UCI | % Weight |
|---|---|---|---|---|
| Adamuz et al., 2020 | 1.2 | 0.87 | 1.67 | 1.55 |
| Aiswarya et al., 2020 | 0.82 | 0.19 | 3.39 | 0.37 |
| Akbariqomi et al., 2020 | 3.05 | 1.29 | 7.23 | 0.12 |
| Alasia et al., 2021 | 1.95 | 0.96 | 3.93 | 0.42 |
| Algado-Sellés et al., 2020 | 4.9 | 1.8 | 13.6 | 0.03 |
| Allard et al., 2020 | 2.04 | 0.85 | 4.85 | 0.25 |
| Allenbach et al., 2020 | 1.34 | 0.65 | 2.77 | 0.68 |
| Alqahtani et al., 2020 | 0.51 | 0.24 | 1.09 | 1.51 |
| Al-Samkari et al., 2020 | 0.61 | 0.41 | 0.93 | 1.77 |
| Alsharidah et al., 2021 | 1.12 | 0.63 | 1.97 | 1.12 |
| Amanat et al., 2021 | 1.38 | 1.03 | 1.86 | 1.52 |
| Bailey et al., 2021 | 0.54 | 0.45 | 0.65 | 1.94 |
| Bauer et al., 2020 | 1.29 | 0.99 | 1.69 | 1.63 |
| Bhargava et al., 2020 | 0.57 | 0.32 | 1.03 | 1.62 |
| Bi et al., 2020 | 2.5 | 1.1 | 6.1 | 0.17 |
| Boero et al., 2020 | 1.25 | 0.73 | 2.14 | 1.07 |
| Bolouri et al., 2021 | 1.33 | 0.94 | 2.67 | 0.87 |
| Bravi et al., 2020 | 1.76 | 1.4 | 2.23 | 1.52 |
| Cai et al., 2020 | 2.34 | 1.44 | 3.81 | 0.58 |
| Çalık Başaran N et al., 2021 | 2.32 | 1.02 | 5.26 | 0.23 |
| Cekerevac et al., 2021 | 1.16 | 0.55 | 2.47 | 0.77 |
| Cen Y et al., 2020 | 1.69 | 1.26 | 2.28 | 1.37 |
| Chaudhry et al., 2020 | 1.61 | 0.28 | 9.23 | 0.06 |
| Chen et al., 2020 | 0.99 | 0.46 | 1.93 | 1.03 |
| Chen J et al., 2020 | 1.14 | 0.97 | 1.34 | 1.86 |
| Chen Q et al., 2020 | 0.94 | 0.46 | 1.93 | 1.03 |
| Chen Z et al., 2020 | 1.71 | 1.21 | 2.44 | 1.20 |
| Cheng et al., 2020 | 1.21 | 0.65 | 2.24 | 0.95 |
| Cizmecioglu et al., 2020 | 1.54 | 0.36 | 6.44 | 0.12 |
| Colaneri et al., 2020 | 2.6 | 0.67 | 10.06 | 0.05 |
| Deng et al., 2020 | 1.79 | 0.83 | 3.84 | 0.41 |
| Derikx et al., 2020 | 2.03 | 0.73 | 5.51 | 0.18 |
| Du et al., 2020a | 1.26 | 0.56 | 2.84 | 0.61 |
| Duan et al., 2020 | 2.16 | 0.81 | 5.78 | 0.17 |
| Etienne et al., 2020 | 2.36 | 0.69 | 7.97 | 0.08 |
| Feng et al., 2020 | 1.6 | 1.04 | 2.45 | 1.07 |
| Fernandes et al., 2021 | 0.91 | 0.45 | 1.89 | 1.05 |
| Ghaffari et al., 2021 | 0.77 | 0.49 | 1.2 | 1.62 |
| Gong et al., 2020 | 1.64 | 0.73 | 3.7 | 0.42 |
| Guan et al., 2020b | 0.97 | 0.7 | 1.36 | 1.66 |

| | | | | |
|---|---|---|---|---|
| Han et al., 2020 | 0.94 | 0.54 | 1.65 | 1.29 |
| He et al., 2020 | 2.52 | 1.8 | 3.54 | 0.86 |
| Hoffmann et al., 2021 | 1.21 | 0.5 | 2.9 | 0.57 |
| Hu F et al., 2021 | 2.89 | 1.07 | 7.83 | 0.10 |
| Hu L et al., 2020 | 1.13 | 0.73 | 1.76 | 1.36 |
| Hu X et al., 2021 | 4.41 | 1.19 | 16.24 | 0.02 |
| Huang R et al., 2020 | 2.29 | 0.86 | 6.07 | 0.16 |
| Huang S et al., 2020 | 1.91 | 1.16 | 3.13 | 0.74 |
| Jang et al., 2020 | 2.98 | 0.23 | 38.81 | 0.00 |
| Ji et al., 2020 | 2.07 | 0.98 | 4.34 | 0.34 |
| Ji et al., 2021 | 0.78 | 0.15 | 4.21 | 0.25 |
| Jinag N et al., 2021 | 1.87 | 1 | 3.52 | 0.53 |
| Kaeuffer et al., 2020 | 2.52 | 1.94 | 3.28 | 1.12 |
| Karahan et al., 2020 | 1.37 | 0.69 | 2.75 | 0.70 |
| Khan et al., 2020 | 2.02 | 1.22 | 3.34 | 0.68 |
| Lei C et al., 2020 | 1.93 | 1.05 | 3.56 | 0.54 |
| Lei et al., 2020 | 1.51 | 1.32 | 1.71 | 1.85 |
| Lei F et al., 2020 | 1.51 | 1.32 | 1.71 | 1.85 |
| Li H et al., 2020 | 5.31 | 2.21 | 12.75 | 0.04 |
| Li K et al., 2020 | 1.5 | 0.57 | 3.88 | 0.35 |
| Li T et al., 2020 | 1.28 | 0.79 | 2.07 | 1.16 |
| Li X et al., 2020 | 1.6 | 1.14 | 2.24 | 1.30 |
| Liu J et al., 2021 | 1.04 | 0.76 | 1.41 | 1.67 |
| Liu S et al., 2020 | 1.69 | 0.99 | 2.89 | 0.78 |
| Molinero et al., 2020 | 1.73 | 1.17 | 2.57 | 1.08 |
| Nachega et al., 2020 | 1.39 | 0.97 | 1.98 | 1.38 |
| Nguyen et al., 2020 | 2.01 | 1.34 | 3.04 | 0.89 |
| Nie Y et al., 2020 | 0.84 | 0.51 | 1.38 | 1.49 |
| Ouldali et al., 2021 | 0.5 | 0.2 | 1.3 | 1.30 |
| Pei et al., 2020 | 1.78 | 1.15 | 2.76 | 0.94 |
| Pereira et al., 2020 | 1.02 | 0.4 | 2.55 | 0.67 |
| Pereira et al., 2020 | 1.02 | 0.4 | 2.55 | 0.67 |
| Qin C et al., 2020 | 1.27 | 0.86 | 1.86 | 1.38 |
| Ren et al., 2020 | 0.54 | 0.25 | 1.15 | 1.47 |
| Sadeghi et al., 2020 | 0.98 | 0.27 | 3.5 | 0.36 |
| Sadeghi-Haddad-Zavareh et al., 2020 | 1.35 | 0.91 | 2 | 1.31 |
| Santus et al., 2020 | 2.22 | 1.05 | 4.72 | 0.29 |
| Scarfo et al., 2020 | 0.73 | 0.34 | 1.57 | 1.20 |
| Shah et al., 2020 | 1.02 | 0.38 | 2.76 | 0.58 |
| Shahriarirad et al., 2020 | 1.04 | 0.29 | 3.78 | 0.32 |
| Shao et al., 2020 | 0.94 | 0.43 | 2.02 | 0.95 |
| Shi et al., 2020 | 2.66 | 1.37 | 5.17 | 0.28 |
| Shi et al., 2020 | 6.8 | 1.44 | 32.2 | 0.00 |
| Siepmann et al., 2020 | 2.77 | 1.1 | 6.99 | 0.13 |

| | | | | |
|---|---|---|---|---|
| Soleimani et al., 2020 | 0.4 | 0.22 | 0.71 | 1.79 |
| Song et al, 2020 | 1.41 | 0.34 | 5.78 | 0.15 |
| Stachura et al., 2021 | 1.07 | 0.47 | 2.41 | 0.76 |
| Stefan et al., 2020 | 0.29 | 0.07 | 1.14 | 1.33 |
| Sun L et al., 2020 | 0.84 | 0.25 | 2.78 | 0.53 |
| Sun S et al., 2020 | 2.23 | 0.9 | 5.1 | 0.23 |
| Tao et al., 2020 | 0.83 | 0.33 | 2.13 | 0.83 |
| Tian et al., 2020 | 1.48 | 0.77 | 2.81 | 0.71 |
| Tian et al., 2020 | 1.46 | 0.85 | 2.51 | 0.91 |
| To et al., 2020 | 1.28 | 0.24 | 6.83 | 0.10 |
| Tong et al., 2020 | 0.7 | 0.16 | 3.13 | 0.42 |
| Vizcarra et al., 2020 | 2.71 | 0.3 | 24.42 | 0.01 |
| Wan et al., 2020 | 0.91 | 0.11 | 1.91 | 0.83 |
| Wang et al., 2020 | 1.62 | 0.56 | 4.71 | 0.24 |
| Wang Fang et al., 2020 | 2.21 | 1.2 | 4.07 | 0.44 |
| Wang Juan et al., 2020 | 0.66 | 0.37 | 1.2 | 1.52 |
| Wang Junhong et al., 2020 | 1.68 | 0.93 | 3.02 | 0.69 |
| Wang Peng et al., 2020 | 1.5 | 0.5 | 4.55 | 0.25 |
| Wang Sijiao et al., 2020 | 2.23 | 0.56 | 8.77 | 0.07 |
| Wang Wen et al., 2021 | 2.27 | 0.85 | 6.1 | 0.16 |
| Wang Wenjun et al., 2021 | 0.85 | 0.71 | 1.02 | 1.89 |
| Wang X et al., 2020 | 1.59 | 1.04 | 2.43 | 1.08 |
| Wei et al., 2020 | 1.73 | 1.05 | 2.85 | 0.83 |
| Wei X et al., 2020 | 1.6 | 1.12 | 2.3 | 1.24 |
| Wei Z et al, 2021 | 0.86 | 0.31 | 2.21 | 0.78 |
| Wu et al., 2020 | 1.016 | 0.6 | 1.7 | 1.30 |
| Xie Y et al., 2020 | 2.03 | 0.72 | 5.73 | 0.17 |
| Xiong S et al., 2020 | 1.01 | 0.46 | 2.22 | 0.85 |
| Xu B et al., 2020 | 1.55 | 0.64 | 3.73 | 0.39 |
| Yang Q et al., 2020 | 1.38 | 0.63 | 3.02 | 0.58 |
| Yayla et al., 2020 | 1.15 | 0.1 | 13.25 | 0.03 |
| Yu et al., 2020 | 4.6 | 1.7 | 12.6 | 0.04 |
| Zachariah et al., 2020 | 0.63 | 0.15 | 2.68 | 0.53 |
| Zhang G et al., 2020 | 1.85 | 0.77 | 4.45 | 0.29 |
| Zhang Hongmei et al., 2020 | 1.46 | 0.54 | 3.96 | 0.33 |
| Zhang Hongyan et al., 2020 | 2.37 | 1.09 | 5.18 | 0.24 |
| Zhang Jing et al., 2020 | 1.22 | 0.92 | 1.62 | 1.63 |
| Zhang KI et al., 2020 | 24.8 | 1.8 | 342.1 | 0.00 |
| Zhang Lijuan et al., 2020 | 3.6 | 0.96 | 13.52 | 0.03 |
| Zhang Nannan et al., 2021 | 3 | 0.33 | 27.06 | 0.01 |
| Zhang Q et al., 2020 | 3.22 | 1.19 | 8.7 | 0.08 |
| Zhang R et al., 2020 | 1.52 | 0.65 | 3.55 | 0.43 |
| Zhang Y et al., 2020 | 3.45 | 1.46 | 8.17 | 0.10 |
| Zhang Y et al., 2021 | 2.61 | 0.84 | 7.45 | 0.10 |
| Zhao J et al., 2021 | 1.28 | 0.8 | 2.04 | 1.19 |

| | | | | |
|---|---|---|---|---|
| Zheng B et al., 2021 | 0.8 | 0.38 | 1.69 | 1.14 |
| Zheng T et al., 2020 | 2.34 | 1.64 | 3.35 | 0.88 |
| Zhou Jun et al., 2021 | 2.39 | 0.74 | 7.77 | 0.09 |
| Zhou Yiwu et al., 2020 | 1.2 | 0.63 | 2.3 | 0.90 |
| Zhu B et al., 2020 | 1.41 | 0.83 | 2.4 | 0.96 |
| Zou et al., 2020 | 3.36 | 1.31 | 8.61 | 0.08 |
| **Total (I squared= 65.2%, p<0.01)** | **1.26** | **1.16** | **1.37** | **100.00** |
