# Supplementary material for: Heterogeneity and Risk of Bias in Studies Examining Risk Factors for Severe Illness and Death in COVID-19: A Systematic Review and Meta-Analysis
Source: Pathogens. 2022 May 10;11(5):563. doi: 10.3390/pathogens11050563 (PMC9147100; doi:10.3390/pathogens11050563)
Supplement: Supplementary file 1 [file pathogens-11-00563-s001.zip › Table S18.pdf]

Heterogeneity and risk of bias in studies examining risk factors for severe illness and death in COVID-19: a systematic review and meta-analysis

Abraham Degarege *, Zaeema Naveed, Josiane Kabayundo, David Brett-Major

Table S18: Heterogeneity analyses of studies that examined the relationship of comorbidities and demography factors with death among COVID-19 patients

Exposure	Overall I^2 (p-value)	I^2 from subgroup analyses				
		Study design (I^2, p-value)	Sample size (I^2, p-value)	Study area /country (I^2, p-value)	Income group (I^2, p-value)	Year of publication (I^2, p-value)
Hypertension	77.3 (<0.01)	CS (84.9%, <0.01)	≤500 (24.5%, p=0.009)	China (72.7%, <0.01)	Middle (79.1%, <0.01)	2020 (76.4%, <0.01)
		P (60.9%, <0.01)	501 to 1499 (75.8%, <0.01)	Italy (85.0%, <0.01)	High (73.2%, <0.01)	2021 (77.1%, <0.01)
		R (77.5%, <0.01)	1500 to 3000 (80.4%, <0.01)	USA (86.8%, <0.01)		
			3001 to 5000 (93.0%, <0.01)	Iran (0.0%, <0.01)		
			5001 to 10000 (81.8%, <0.01)	UK (0.0%, p= 0.587)		
			10001 to 50000 (98.6%, <0.01)	Brazil (36.0%, p= 0.196)		
			500001 to 100000 (96.7%, <0.01)	Spain (54.5%, p= 0.002)		
				Turkey (53.1%, p= 0.030		
				Korea (0.0%, p= 0.788)		
				France (68.8%, <0.01)		
				India (45.0%, p= 0.122)		
Cancer	85.1 (<0.01)	CS (0.0%, p=0.622)	≤500 (0.0%, p= 0.977)	China (17.0%, p= 0.259)	Middle (89.8%, p<0.01)	2020 (16.1%, p= 0.162)
		P (0.0%, p= 0.728)	501 to 1499 (75.8%, p<0.01)	Italy (0.0%, p=0.625)	High (4.6%, p= 0.396)	2021 (93.8%, p<0.01)
		R (88.3%, <0.01)	1500 to 3000 (10.0%, p=0.329)	USA (72.6%, p<0.01)		
			3001 to 5000 (37.3%, p=0.188)	UK (0.0%, p=0.943)		
			5001 to 10000 (95.8%, p<0.01)	Spain (0.0%, p=0.810)		
			10001 to 50000 (47.2%, p= 0.108)	France (90.8%, p<0.01)		
			500001 to 100000 (76.8%, p= 0.002)	Turkey (0.0%, p=0.637)		
Diabetes	95.5% (<0.01)	CS (99.4%, p<0.01)	≤500 (7.2%, p= 0.258)	China (57.9%, p<0.01)	Middle (97.1%, p<0.01)	2020 (96.8%, p<0.01)
		P (45.6%, p= 0.001)	501 to 1499 (32.3%, 0.017)	Italy (43.3%, p=0.015)	High (82.6%, p<0.01)	2021 (69.5%, p<0.01)
		R (70.0%, p<0.01)	1500 to 3000 (67.4%, p= 0.001)	USA (67.4%, p<0.01)		
			3001 to 5000 (87.7%, p<0.01)	UK (99.6%, p<0.01)		
			5001 to 10000 (87.5%, p<0.01)	Spain (58.5%, p<0.01)		
			10001 to 50000 (94.2%, p<0.01)	France (42.2%, p=0.086)		
			500001 to 100000 (99.9%, p<0.01)	Turkey (45.4%, p=0.050)		
				Iran (0.0%, p=0.574)		

				Brazil (96.1%, p<0.01)		
Ages (≥60 vs. <60 years)	95.7% (<0.01)	CS (95.9%, p<0.01)	≤500 (43.6%, p= 0.014)	China (95.6%, p<0.01)	Middle (87.1%, p<0.01)	2020 (93.1%, p<0.01)
		P (64.3%, p< 0.01)	501 to 1499 (60.3%, p=0.005)	Italy (90.3%, p<0.01)	High (95.5%, p<0.01)	2021 (95.9%, p<0.01)
		R (67.3%, p<0.01)	1500 to 3000 (0.0%, p= 0.754)	USA (54.6%, p=0.024)		
			5001 to 10000 (91.3%, p=0.01)	Spain (21.6%, p=0.271)		
			10001 to 50000 (98.6%, p<0.01)	Iran (41.8%, p= 0.161)		
			500001 to 100000 (0.0%, p=1.00)			
Age (≥ 65 vs. < 65 years	96.7% (<0.01)	P (92.7%, p< 0.01)	≤500 (50.9%, p= 0.002)	China (75.1%, p<0.01)	Middle (97.0%, p<0.01)	2020 (97.2%, p<0.01)
		R (95.4%, p<0.01)	501 to 1499 (69.2%, p<0.01)	Italy (87.5%, p<0.01)	High (78.9%, p<0.01)	2021 (79.9%, p<0.01)
			5001 to 10000 (97.8%, p<0.01)	USA (98.4%, p<0.01)		
Gender	89.3% (<0.01)	CS (91.5%, p<0.01)	≤500 (31.7%, p<0.01)	China (96.5%, p<0.01)	Middle (64.3%, p<0.01)	2020 (76.5%, p<0.01)
		P (58.1%, p= 0.001)	501 to 1499 (50.4%, p<0.01)	Italy (2.7%, p= 0.424)	High (94.6%, p<0.01)	2021 (85.4%, p<0.01)
		R (90.9%, p<0.01)	1500 to 3000 (0.0%, p= 0.670)	USA (75.3%, p<0.01)		
			3001 to 5000 (79.1%, p<0.01)	UK (59.1%, p=0.007)		
			5001 to 10000 (93.8%, p<0.01)	Spain (68.3%, p<0.01)		
			10001 to 50000 (90.4%, p<0.01)	France (42.2%, p=0.086)		
			500001 to 100000 (90.8%, p<0.01)	Turkey (27.9%, p= 0.196		
				Iran (0.0%, p=0.817)		
				Brazil (96.0%, p<0.01)		
Smoking	96.6% (<0.01)	CS (95.7%, p<0.01)	≤500 (67.6%, p<0.01)	China (0.0%, p=0.827)	Middle (76.2%, p<0.01)	2020 (75%, p<0.01)
		P (99.0%, p= 0.001)	501 to 1499 (48.4%, p=0.011)	Italy (54.6%, p= 0.007)	High (4.1%, p<0.01)	2021 (96%, %, p<0.01)
		R (0.6%, p=0.462)	1500 to 3000 (0.0%, p= 0.879)	USA (54.6%, p= 0.007)		
			3001 to 5000 (79.2%, p=0.001)	Spain (89.8%, p<0.01)		
			5001 to 10000 (99.2%, p<0.01)	Turkey (50.7%, p= 0.087		
			500001 to 100000 (90.8%, p<0.01)			
Cardiovascular disease	80.2% (<0.01)	CS (87.6%, p<0.01)	≤500 (0.0%, p= 0.692)	China (76.5%, p<0.01)	Middle (82.6%, p<0.01)	2020 (82.3%, p<0.01)
		P (73.5%, p<0.01)	501 to 1499 (75.1%, p<0.01)	Italy (60.0%, p= 0.005)	High (78.7%, p<0.01)	2021 (65.1%, p<0.01)
		R (72.9%, p<0.01)	1500 to 3000 (90.7%, p<0.01)	USA (2.7%, p= 0.422)		
			3001 to 5000 (80.4%, p<0.01)	Spain (74.2%, p<0.01)		
			5001 to 10000 (95.6%, p<0.01)	Turkey (50.7%, p= 0.087)		
			500001 to 100000 (96.2%, p<0.01)	Iran (0.0%, p=0.983)		
				Turkey (0.0%, p=0.458)		

				Brazil (93.3%, p<0.01		
				UK (19.5%, p= 0.286)		
Chronic respiratory disease	72.5% (<0.01)	CS (88.3%, p<0.01)	≤500 (15.2%, p= 0.139)	China (9.8%, p=0.321)	Middle (78.3%, p<0.01)	2020 (52.3%, p<0.01)
		P (4.3%, p=0.401)	501 to 1499 (36.0%, p=0.021)	Italy (0.0%, p=0.726)	High (59.5%, p<0.01)	2021 (80.6%, p<0.01)
		R (71.8%, p<0.01)	1500 to 3000 (92.1%, p<0.01)	USA (89.6%, p<0.01)		
			3001 to 5000 (73.7%, p<0.01)	Spain (73.8%, p<0.01)		
			5001 to 10000 (64.1%, p=0.016)	Turkey (0.0%, p= 0.715)		
			500001 to 100000 (74.7%, p= 0.008)	Iran (0.0%, p=0.983)		
Cerebrovascular disease	61.0% (<0.01)	P (0.0%, p=0.590)	≤500 (0.0%, p=0.950)	China (0.0%, p= 0.699)	Middle (72.3%, p<0.01)	2020 (5.7%, p=0.387)
		R (68.1%, p<0.01)	501 to 1499 (71.3%, p= 0.001)	Italy (0.0%, p=0.875)	High (0.0%, p=0.835)	2021 (78.2%, p<0.01)
				USA (92.0%, p<0.01)		
				Spain (0.0%, p=0.507)		
Chronic kidney disease	79.2% (<0.01)	CS (78.2%, p<0.01)	≤500 (43.5%, p<0.01)	China (58.7%, p=0.001)	Middle (81.2%, p<0.01)	2020 (68.9%, p<0.01)
		P (58.5%, p=0.001)	501 to 1499 (37.0%, p=0.019)	Italy (64.5%, p<0.01)	High (74.3%, p<0.01)	2021 (84.5%, p<0.01)
		R (80.4%, p<0.01)	1500 to 3000 (57.7%, p= 0.037)	USA (90.6%, p<0.01)		
			3001 to 5000 (83.7%, p<0.01)	Spain (69.8%, p<0.01)		
			5001 to 10000 (92.4%, p<0.01)	Turkey (0.0%, p= 0.763)		
				Iran (0.0%, p= 0.668)		
				Brazil (80.5%, p= 0.002)		
				UK (56.1%, p= 0.058)		

CS: cross-sectional; P: prospective; R: Retrospective
