# Supplementary material for: Heterogeneity and Risk of Bias in Studies Examining Risk Factors for Severe Illness and Death in COVID-19: A Systematic Review and Meta-Analysis
Source: Pathogens. 2022 May 10;11(5):563. doi: 10.3390/pathogens11050563 (PMC9147100; doi:10.3390/pathogens11050563)
Supplement: Supplementary file 1 [file pathogens-11-00563-s001.zip › Table S19.pdf]

Heterogeneity and risk of bias in studies examining risk factors for severe illness and death in COVID-19: a systematic review and meta-analysis

Abraham Degarege *, Zaeema Naveed, Josiane Kabayundo, David Brett-Major

Table S19: Heterogeneity analyses of studies that examined the relationship of chronic kidney disease and demographic factors with severe illness among COVID-19 patients

Exposure	Overall I^2 (p-value)	I^2 from subgroup analyses				
		Study design (I^2, p-value)	Sample size (I^2, p-value)	Study area /country (I^2, p-value)	Income group (I^2, p-value)	Year of publication (I^2, p-value)
Ages (≥60 vs. <60 years)	61.9% (<0.01)	CS (0.0%, p= 0.940)	≤500 (16.7%, p= 0.271)	China (67.8%, p<0.01)	High (0.0%, p= 0.582)	2020 (65.8%, p<0.01)
		P (0.0%, p= 0.403)	501 to 1499 (84.4%, p<0.01)		Middle (72.3%, p<0.01)	2021 (0.0%, p= 0.813)
		R (70.6%, p<0.01)				
Age (≥ 65 vs. < 65 years	69.5% (<0.01)	CS (0.0%, p= 0.571)	≤500 (47.2%, p= 0.007)	China (77.0%, p<0.01)	High (0.0%, p=0.531)	2020 (50.3%, p=0.002)
		P (55.2%, p= 0.082)	501 to 1499 (67.2% p= 0.006)		Middle (73.5%, p<0.01)	2021 (40.1%, p=0.138)
		R (71.3%, p<0.01)				
Gender	65.2% (<0.01)	CS (30.4%, p=0.084)	≤500 (15.4%, p= 0.096)	China (36.1%, p=0.001)	High (76.9%, p<0.01)	2020 (55.5%, p<0.01)
		P (84.2%, p<0.01)	501 to 1499 (81.2%, p<0.01)	Italy (0.0%, p= 0.565)	Middle (47.3%, p<0.01)	2021 (54.0%, p=0.01)
		R (50.4%, p<0.01)	1500 to 3000 (85.1%, p<0.01)	USA (68.0%, p= 0.001)		
				UK (59.1%, p=0.007)		
				Spain (3.6%, p=0.375)		
				France (42.2%, p=0.086)		
				Turkey (0.0%, p= 0.896)		
				Iran (74.8%, p= 0.001)		
Chronic kidney	76.0% (<0.01)	CS (64.9%, p=0.022)	≤500 (32.8%, p=0.078)	China (28.6%, p=0.131)	High (0.0%, p=0.449)	2020 (77.5%, p<0.01)
		P (44.3%, p= 0.166)	501 to 1499 (0.0%, p= 0.808)	Iran (88.5%, p<0.01)	Middle (64.5%, p<0.01)	2021 (0.0%, p=0.975)
		R (67.0%, p=0.462)	1500 to 3000 (0.567, p= 0.0%)	USA (0.0%, p= 0.839)		

CS: cross-sectional; P: prospective; R: Retrospective
