# Supplementary material for: Heterogeneity and Risk of Bias in Studies Examining Risk Factors for Severe Illness and Death in COVID-19: A Systematic Review and Meta-Analysis
Source: Pathogens. 2022 May 10;11(5):563. doi: 10.3390/pathogens11050563 (PMC9147100; doi:10.3390/pathogens11050563)
Supplement: Supplementary file 1 [file pathogens-11-00563-s001.zip › Table S20.pdf]

Heterogeneity and risk of bias in studies examining risk factors for severe illness and death in COVID-19: a systematic review and meta-analysis

Abraham Degarege *, Zaeema Naveed, Josiane Kabayundo, David Brett-Major

Table S20: Heterogeneity analyses of studies that examined the relationship of comorbidities and demography factors with admission to the ICU among COVID-19 patients

Exposure	Overall I^2 (p-value)	I^2 from subgroup analyses				
		Study design (I^2, p-value)	Sample size (I^2, p-value)	Study area /country (I^2, p-value)	Income group (I^2, p-value)	Year of publication (I^2, p-value)
Hypertension	63.1% (<0.01)	CS (75.4%, p= 0.003	≤500 (18.8%, p= 0.193)	China (72.3%, p<0.01	High (60.6%, p<0.01)	2020 (69.7%, p<0.01)
		P (62.8%, p<0.01)	501 to 1499 (36.2%, p=0.195)	USA (79.3%, p<0.01)	Middle (63.5%, p<0.01)	2021 (0.0%, p= 0.790)
			1500 to 3000 (88.7%, p=0.03)	Italy (54.7%, p= 0.085)		
Diabetes	53.7 (<0.01)	CS (37.4%, p= 0.157	≤500 (0.0%, p= 0.796)	China (56.2%, p= 0.009)	High (68.8%, p<0.01)	2020 (41.0%, p=0.015)
		P (17.2%, p= 0.305)	501 to 1499 (69.9%, p= 0.003)	Italy (0.0%, p= 0.407)	Middle (63.5%, p<0.01)	2021 (53.2%, p= 0.004)
		R (54.2%, p<0.01)	1500 to 3000 (55.7%, p= 0.105)	USA (11.5%, p= 0.335)		
			5001 to 10000(57.6%, p=0.095)	France (17.0%, p= 0.306)		
Chronic Kidney disease	53.1 (<0.01)	CS (0.0%, p= 0.641)	≤500 (0.0%, p= 0.951)	China (0.0%, p= 0.980)	High (68.8%, p<0.01)	2020 (58.8%, p= 0.002)
		P (0.0%, p= 0.847)	501 to 1499 (90.1%, p<0.01)	USA (29.6%, p= 0.213)	Middle (0.0%, p=0.926)	2021 (5.9%, p= 0.385)
		R (56.0%, p=0.002)				
Smoking	59.9% (<0.01)	CS (29.4%, p= 0.234	≤500 (0.0%, p= 0.676)	China (6.9%, p=0.342)	High (36.6%, p=0.126)	2020 (66.5%, p= 0.004)
		R (66.0%, p=0.001)	501 to 1499 (38.7%, p=0.163)	USA (14.0%, p= 0.325)	Middle (61.7%, p=0.034	2021 (0.0%, p=0.625)
Gender	70.0% (<0.01)	CS (0.0%, p= 0.383)	≤500 (23.4%, p= 0.099)	China (67.2%, p<0.001)	High (44.0%, p=0.005)	2020 (68.8%, p<0.01)
		P (0.0%, p=0.820)	501 to 1499 (76.2%, p=0.02)	Italy (0.0%, p= 0.883)	Middle (62.1%, p<0.01)	2021 (42.0%, p= 0.028)
		R (74.6%, p<0.01)	1500 to 3000 (85.1%, p<0.01)	USA (57.5%, p= 0.009)		
				Spain (65.2%, p= 0.035)		
				France (0.0%, p= 0.823)		

CS: cross-sectional; P: prospective; R: Retrospective
