# Supplementary material for: Heterogeneity and Risk of Bias in Studies Examining Risk Factors for Severe Illness and Death in COVID-19: A Systematic Review and Meta-Analysis
Source: Pathogens. 2022 May 10;11(5):563. doi: 10.3390/pathogens11050563 (PMC9147100; doi:10.3390/pathogens11050563)
Supplement: Supplementary file 1 [file pathogens-11-00563-s001.zip › Table S21.pdf]

Heterogeneity and risk of bias in studies examining risk factors for severe illness and death in COVID-19: a systematic review and meta-analysis

Abraham Degarege *, Zaeema Naveed, Josiane Kabayundo, David Brett-Major

Table S21: Meta-regression analysis examining factors affecting the odds ratio of death among COVID-19 patients with comorbidities at the study level

	Hypertension	Cancer	Diabetes	Cardiovascular disease	Chronic respiratory disease	Chronic kidney disease	Smoking	Gender	Age ≥60 vs <60 years	Age ≥65 vs <65 years	Age continuous
Study area (β & p-value)	0.0003 (0.944)	0.002 (0.873)	0.01 (0.009)	-0.001 (0.890)	-0.01 (0.183)	-0.003 (0.733)	0.001 (0.821)	-0.007 (0.024)	0.01 (0.360)	0.001 (0.911)	0.001 (0.257)
Study design (β & p-value)	0.01 (0.831)	-0.02 (0.877)	-0.02 (0.877)	0.09 (0.272)	0.07 (0.412)	-0.01 (0.918)	0.08 (0.409)	0.06 (0.108)	-0.10 (0.519)	0.29 (0.147)	-0.005 (0.730)
Sample size (β & p-value)	-0.04 (0.228)	-0.04 (0.245)	0.02 (0.349)	-0.03 (0.392)	-0.03 (0.440)	0.03 (0.490)	-0.05 (0.250)	0.003 (0.809)	0.128 (0.028)	0.04 (0.497)	0.001 (0.888)
Year of publication (β & p-value)	-0.15 (0.089)	-0.03 (0.835)	-0.18 (0.024)	-0.26 (0.059)	-0.28 (0.015)	-0.19 (0.234)	0.06 (0.585)	-0.03 (0.635)	0.08 (0.705)	-0.18 (0.559)	-0.006 (0.735)
