# Supplementary material for: Heterogeneity and Risk of Bias in Studies Examining Risk Factors for Severe Illness and Death in COVID-19: A Systematic Review and Meta-Analysis
Source: Pathogens. 2022 May 10;11(5):563. doi: 10.3390/pathogens11050563 (PMC9147100; doi:10.3390/pathogens11050563)
Supplement: Supplementary file 1 [file pathogens-11-00563-s001.zip › Table S22.pdf]

Heterogeneity and risk of bias in studies examining risk factors for severe illness and death in COVID-19: a systematic review and meta-analysis

Abraham Degarege *, Zaeema Naveed, Josiane Kabayundo, David Brett-Major

Table S22: Risk of bias and quality of the studies included in this review

References	Selection bias	Design	Confounders	Blinding (detection and reporting bias)	Data collection	Withdrawals and dropouts	Overall quality
Abadia-Otero et al., 2021	2	2	1	2	1	3	2
Abbas et al., 2021	2	2	3	3	1	3	3
Acar et al., 2021	2	2	2	2	1	2	1
Adamuz et al., 2021	2	2	1	2	1	2	1
Agarwal et al., 2020	2	2	1	2	1	3	2
Aggarwal et al. 2020	2	2	1	2	1	3	2
Ahmad et al., 2021	2	2	3	3	1	3	3
Aiswarya et al., 2021	2	2	3	3	1	3	3
Akbariqomi et al., 2020	2	2	1	3	1	3	3
Akhtar et al., 2021	2	2	3	2	1	2	2
Alahmari et al., 2020	2	3	1	1	1	3	2
Alasia et al., 2020	2	2	1	3	1	3	3

Albani et al., 2020	2	2	1	3	1	1	2
Alberici et al., 2020	2	2	3	3	1	3	3
Alfraij et al., 2020	2	2	3	2	1	3	3
Algado-Selles et al., 2020	2	2	1	3	1	3	3
Algahtani et al., 2020	2	3	3	2	1	N/A	3
Alguwaihes et al., 2021	2	2	1	2	1	3	2
Aliberti et al., 2021	2	2	1	2	1	2	1
Aljishi et al., 2020	2	2	1	2	1	3	2
Allemah et al., 2020	2	2	1	2	1	3	2
Allard et al., 2020	1	2	3	2	2	1	2
Allenbach et al., 2020	2	2	3	3	1	3	3
Almaghlouth et al., 2021	2	2	2	3	2	3	3
Aloisio et al., 2020	2	2	3	2	1	1	2
Al-Salameh et al., 2021	2	2	1	2	1	3	2
Al-Samkari et al., 2020	2	2	1	2	1	1	1
Alser et al., 2021	2	2	1	3	1	3	3
Alsharidah et al., 2020	2	1	1	3	1	3	3
Altschul et al., 2020	2	2	2	2	1	3	2
Alzaid et al., 2020	2	2	1	3	3	3	3
Amanat et al., 2021	2	2	1	3	2	1	2
Amo et al., 2020	2	2	2	2	1	3	2
Anakli et al., 2021	2	2	3	2	1	3	3
Anantharaman et al., 2020	2	2	1	2	1	3	2

Andargie et al., 2021	2	2	1	3	1	3	3
Angelidi et al., 2021	2	2	1	2	1	3	2
Angelis et al., 2020	2	2	1	3	1	3	3
Annweiler et al., 2020	1	2	1	2	1	3	2
Antunez-Montes et al., 2021	2	2	1	2	1	3	2
Aoun et al., 2021	2	2	2	2	1	3	2
Apea et al., 2021	2	2	1	2	1	2	1
Attauabi et al., 2021	2	2	1	2	1	3	2
Auld et al., 2020	2	2	3	2	1	3	3
Avci et al., 2020	2	2	2	2	1	2	1
Avruscio et al., 2020	2	2	1	2	1	3	2
Aw et al., 2020	2	2	1	2	1	3	2
Awad et al., 2021	2	2	2	2	1	3	2
Azar et al., 2020	2	2	1	2	1	1	1
Azwar et al., 2020	2	2	3	2	1	3	3
Baggio et al., 2021	2	3	2	2	1	2	2
Bailey et al., 2021	2	2	1	2	1	3	2
Bain et al., 2020	2	2	3	2	2	3	3
Bannaga et al., 2020	2	2	2	2	1	3	2
Baqui et al., 2020	2	3	3	2	1	3	3
Barron et al., 2020	2	2	1	2	1	3	2
Bartoletti et al., 2020	2	2	1	2	3	3	3
Basaran et al., 2021	2	2	2	2	1	3	2
Bauer et al. 2021	2	2	1	2	1	3	2

Bezzio et al. 2020	1	2	3	2	2	3	3
Becerra-Munoz et al., 2021	2	2	2	2	1	1	1
Bedock et al., 2020	2	2	2	2	1	2	1
Belli et al., 2021	2	2	2	2	1	2	1
Bellino et al. 2020	2	2	2	2	1	3	2
Berenguer et al., 2020	2	2	2	2	1	2	1
Bernard et al., 2021	2	2	1	2	1	3	2
Bertsimas et al., 2020	2	2	2	2	1	3	2
Betti et al., 2020	2	2	3	2	1	3	3
Bhargava et al., 2020	2	2	1	2	1	3	2
Bhatt et al., 2020	2	2	1	2	1	3	2
Bianchetti et al., 2020	2	2	3	3	3	3	3
Bielza et al., 2020	2	2	1	2	1	2	1
Birati et al., 2021	2	3	3	2	1	3	3
Bode et al., 2020	2	2	3	2	1	3	3
Boero et al., 2021	2	2	2	2	1	3	2
Bolouri et al., 2020	2	2	2	3	1	3	3
Bossini et al., 2020	2	2	2	3	1	3	3
Botta et al., 2021	2	2	1	2	1	1	1
Bottio et al., 2020	2	2	3	3	3	3	3
Brandao-Neto et al., 2021	2	2	2	3	1	1	2
Bravi et al., 2020	2	2	2	2	1	3	2
Brenner et al., 2020	2	2	1	2	2	3	2
Brouns et al., 2020	2	3	2	3	1	3	3

Bruggemann et al., 2020	2	2	2	3	1	3	3
Byeon et al., 2021	1	3	2	3	1	1	3
Cabello et al., 2020	2	2	2	2	1	3	2
Cabezudo-Garcia et al., 2020	2	3	2	3	1	3	3
Cai et al., 2021	2	2	1	2	1	1	1
Cai et al., 2021	2	2	1	3	1	3	3
Cai et al., 2020	2	2	2	3	1	3	3
Cai et al., 2020	2	2	2	2	1	3	2
Caillard et al., 2020	2	2	1	3	1	3	3
Calik-Basaran et al., 2021	2	2	3	3	1	3	3
Caliskan & Saylan, 2020	2	2	2	2	2	3	2
Cao et al., 2020	2	2	3	3	1	1	3
Capdedila-Reniu et al., 2021	2	2	2	2	1	3	2
Cariou et al., 2020	2	2	2	3	1	1	2
Carter et al., 2020	2	2	1	3	1	1	2
Carubbi et al., 2021	2	2	3	3	1	2	3
Catteau et al., 2020	2	2	1	2	2	3	2
Cattelan et al., 2020	2	2	3	3	1	3	3
Cekerevac et al., 2021	2	3	3	3	1	3	3
Cen et al., 2020	2	2	1	3	1	3	3
Chand et al., 2020	2	2	1	2	1	3	2
Chang et al., 2020	2	2	3	2	1	3	3
Chaudhry et al., 2020	2	2	3	3	3	3	3
Chen et al., 2020	2	2	1	2	1	3	2

Chen et al., 2020	2	2	2	2	1	3	2
Chen et al., 2020	2	2	2	3	1	3	3
Chen et al., 2020	2	2	2	2	1	3	2
Chen et al., 2020	2	2	2	2	1	3	2
Chen et al., 2020	2	2	2	2	1	3	2
Chen et al., 2021	2	2	2	2	1	3	2
Chen et al., 2020	2	2	2	3	1	3	3
Cheng et al., 2020	2	2	1	2	1	3	2
Chilimuri et al., 2020	2	2	1	1	1	3	2
Choron et al., 2021	2	2	1	2	1	1	1
Choudhuri et al., 2020	2	2	2	2	1	3	2
Ciceri et al., 2020	2	2	2	3	2	2	2
Cinar et al., 2021	2	3	2	2	1	3	3
Cizmecioglu et al., 2021	2	2	3	3	1	3	3
Collaborative Geriatric Medicine Research et al., 2021	2	2	1	3	1	3	3
COVID-ICU Group, 2021	2	2	2	2	1	1	1
COVIDSurg Collaborative, Lancet, 2020	2	2	2	2	1	1	1
Covino et al., 2021	2	2	3	2	1	3	3
Covino et al., 2020	2	2	2	2	1	3	2
Covino et al., 2020	2	2	2	2	2	3	2
Covino et al., 2020	2	2	1	2	1	2	1
Cummings et al., 2020	2	2	1	2	1	3	2

Dagher et al., 2021	2	2	2	2	1	3	2
Dai et al., 2020	2	2	3	2	3	3	3
DeSemet et al., 2020	2	2	2	2	1	3	2
DeSouza et al., 2020	2	3	2	2	2	3	3
DeSouza et al., 2021	2	2	2	2	1	3	2
Deeb et al., 2021	2	2	2	3	2	3	3
Del Amo et al., 2020	2	2	3	3	1	3	3
Di Castelnuovo et al., 2020	2	2	1	2	1	1	1
Di Cosimo et al., 2021	2	2	2	3	3	3	3
Du et al., 2020	2	2	2	3	1	3	3
Du et al., 2020	2	2	3	2	1	3	3
Duan et al., 2020	2	2	2	2	1	1	1
Duarte et al., 2020	2	2	2	3	3	3	3
Duclos et al., 2021	2	2	2	2	1	3	2
Ebinger et al., 2021	1	3	1	2	1	3	3
Elhadi et al., 2021	2	2	3	2	1	1	2
Escobar et al., 2020	2	3	3	3	3	3	3
Escosteguy et al., 2020	2	3	2	3	1	3	3
Etienne et al., 2020	2	2	2	3	2	3	3
Fadini et al., 2020	2	2	1	2	1	3	2
Falconi et al., 2020	2	2	1	3	1	3	3
Fava et al., 2020	2	2	2	3	1	1	2
Ferguson et al., 2020	2	2	3	3	1	3	3

Fernandes et al., 2021	2	2	1	2	1	1	1
Ferrando et al., 2020	2	2	2	2	1	3	2
Ferrando-Vivas et al., 2021	2	2	1	2	1	1	1
Ferrari et al., 2021	2	2	2	2	1	3	2
Fox et al., 2021	2	3	1	3	1	1	3
Foy et al., 2020	2	2	2	3	1	3	3
Freites et al., 2020	2	2	2	3	1	3	3
Frontera et al., 2020	2	2	1	2	1	1	1
Fu et al., 2020	2	2	3	3	1	3	3
Galiero et al., 2020	2	2	2	3	1	1	2
Gao et al., 2020	2	2	2	2	1	3	2
Gao et al., 2020	2	3	2	3	1	3	3
Genet et al., 2020	2	2	1	2	1	2	1
Ghaffari et al., 2021	2	2	2	3	1	3	3
Giannoglou et al., 2020	2	3	2	3	3	3	3
Goicoechea et al., 2020	2	2	3	3	3	3	3
Gomez et al., 2021	2	2	2	2	1	1	1
Gonzalez-Gancedo et al., 2021	2	3	3	2	2	1	3
Goodall et al, 2020	2	2	2	3	1	3	3
Grasselli et al., 2020	2	2	1	3	1	1	2
Gu et al., 2020	2	2	1	2	1	3	2
Guarneri et al., 2021	2	3	3	3	1	3	3
Guo et al., 2020	2	3	3	3	1	3	3
Gupta et al., 2020	2	2	1	2	1	1	1

Haase et al., 2021	2	2	2	2	1	3	2
Halvatsiotis et al., 2020	2	2	3	2	2	3	3
He et al., 2020	2	2	1	3	1	3	3
Hendra et al., 2021	2	2	2	3	1	1	2
Hashemi et al., 2020	2	2	1	3	1	3	3
Hoffmann et al., 2021	2	3	1	2	1	3	3
Hu et al., 2021	2	2	2	3	1	1	2
Hu et al., 2020	2	3	2	2	1	2	2
Hu et al., 2021	2	2	2	2	1	3	2
Huang et al., 2020	2	2	1	2	1	3	2
Huang et al., 2020	2	2	2	3	1	3	3
Huang et al., 2020	2	2	3	2	1	3	3
Hussein et al., 2020	2	2	2	3	1	3	3
Hwang et al., 2020	2	2	2	2	1	3	2
Iaccarino et al., 2020	2	3	2	2	1	1	2
Imam et al., 2020	2	2	2	3	1	3	3
Incerti et al., 2021	2	2	1	2	1	2	1
Inciarbi et al., 2020	2	2	2	3	1	3	3
Islam et al., 2020	2	2	2	3	1	1	2
Jain et al., 2021	2	2	2	3	1	3	3
Jang et al., 2020	2	2	2	2	1	1	1
Jegede et al., 2021	2	2	3	3	2	3	3
Jiang et al., 2021	2	2	2	2	1	1	1
Jiang et al., 2020	2	2	3	2	1	1	2

Ji et al., 2021	2	2	3	3	1	3	3
Jimeno et al., 2021	2	2	2	3	1	3	3
Jorgensen et al., 2020	2	2	3	3	1	3	3
Jung et al., 2021	2	2	2	3	1	3	3
Kabarriti et al., 2020	2	2	2	2	1	3	2
Kaeuffer et al., 2020	2	2	1	2	1	3	2
Kalligeros et al., 2020	2	2	1	2	1	3	2
Karahan et al., 2021	2	3	2	3	1	3	3
Karimi et al., 2020	2	2	3	3	1	3	3
Kaya et al., 2021	2	2	2	2	1	3	2
Khan et al., 2020	2	2	1	2	1	3	2
Kim et al., 2020	2	2	1	2	1	3	2
Kim et al., 2021	2	2	2	2	1	3	2
Kim et al., 2021	2	2	2	2	1	1	1
Krause et al., 2021	2	2	1	2	1	3	2
Krishnamoorthy et al., 2021	2	2	2	2	1	3	2
Krishnan et al., 2020	2	2	3	2	2	3	3
Kunal et al., 2020	2	2	1	2	1	1	1
Kute et al., 2021	2	2	2	3	1	3	3
Laccarino et al., 2020	2	3	2	3	3	1	3
Laake et al., 2021	2	3	3	2	1	3	3
Lara et al., 2020	2	2	1	2	1	3	2
Lauranne et al., 2020	2	2	3	3	1	3	3
Lee et al., 2021	2	2	1	2	1	3	2

Lee et al., 2020	2	2	1	2	1	3	2
Lee et al., 2020	2	2	1	3	1	3	3
Lee et al., 2020	2	2	2	2	1	1	1
Lei et al., 2020	2	2	2	3	1	3	3
Lei et al., 2020	2	2	1	2	1	1	1
Li et al., 2020	2	3	2	2	1	1	2
Li et al., 2021	2	2	2	2	1	1	1
Li et al., 2021	2	2	3	3	1	3	3
Li et al., 2020	2	2	2	2	1	1	1
Lian et al., 2020	2	2	2	2	1	3	2
Lievre et al., 2020	2	2	2	3	1	1	2
Ling et al., 2020	2	2	1	2	1	3	2
Liu et al., 2020	2	2	2	3	2	3	3
Liu et al., 2021	2	2	2	3	1	3	3
Liu et al., 2020	2	2	2	2	2	1	1
Lohia et al., 2021	2	2	1	2	1	1	1
Long et al., 2020	2	2	3	3	1	3	3
Lorente et al., 2021	2	2	2	2	1	3	2
Lorente et al., 2021	2	2	2	2	1	3	2
Louapre et al., 2020	2	2	2	3	2	1	2
Lu et al., 2021	2	2	2	2	1	3	2
Lucar et al., 2021	2	2	2	3	1	3	3
Lunski et al., 2021	2	2	2	3	1	3	3
Luo et al., 2020	2	2	2	2	1	1	1

Maguire et al., 2020	2	2	2	3	1	3	3
Mak et al., 2021	2	2	2	3	1	3	3
Marengoni et al., 2020	2	2	2	3	1	3	3
Marouf et al., 2021	2	2	2	3	1	3	3
Martins et al., 2021	2	2	3	3	1	3	3
Martinez-Lopez et al., 2020	2	2	2	3	1	2	2
Mash et al., 2021	2	3	2	2	1	3	3
Mather et al., 2020	2	2	2	2	1	3	2
Mato et al., 2020	2	2	2	3	1	3	3
Mayer et al., 2021	2	2	2	3	1	3	3
Mehta et al., 2021	2	2	2	2	2	3	2
Mehta et al., 2020	2	2	1	2	1	3	2
Menager et al., 2020	2	2	2	3	1	1	2
Mendes et al., 2020	2	2	2	2	1	3	2
Messika et al., 2021	2	2	2	3	1	3	3
Moghaddam et al., 2020	2	2	3	3	1	3	3
Morrison et al., 2020	2	2	2	3	2	3	3
Murthy et al., 2021	2	2	2	2	1	3	2
Nachega et al., 2020	2	2	1	2	1	1	1
Nachtigall et al., 2020	2	2	2	2	1	1	1
Nakamura et al., 2021	2	2	3	2	1	3	3
Namendys-Silva et al., 2021	2	2	2	3	1	1	2
Ng et al., 2020	2	2	1	3	3	1	3
Nie et al., 2021	2	2	1	2	1	1	1

Nie et al., 2020	2	2	2	3	1	3	3
Nunez et al., 2021	2	2	2	2	1	3	2
O'Horo et al., 2021	2	2	2	2	1	1	1
Okoh et al., 2020	2	2	2	2	1	1	1
Oto et al., 2021	2	2	2	3	1	1	2
Ouldali et al., 2021	2	2	2	3	1	2	2
Ozdemir et al., 2021	2	2	2	2	1	1	1
Ozturk et al., 2020	2	2	2	2	1	1	1
Park et al., 2021	2	2	2	2	1	3	2
Park et al., 2020	2	2	2	2	2	3	2
Parlak et al., 2021	2	2	2	2	1	3	2
Pascual et al., 2020	2	2	3	3	2	3	3
Passamonti et al., 2020	2	2	2	3	1	1	2
Patel et al., 2020	2	2	2	2	1	2	1
Pepe et al., 2021	2	2	1	2	1	1	1
Peiro et al., 2021	2	2	2	3	1	3	3
Pinato et al., 2020	2	2	3	3	3	3	3
Pineiro et al., 2021	2	2	1	3	1	3	3
Pizzini et al., 2020	2	2	3	3	1	3	3
Plotnikow et al., 2020	2	2	3	3	1	2	3
Pongpirul et al., 2020	2	2	1	2	1	3	2
Prado-Galbarro et al., 2020	2	2	2	2	1	3	2
Qian et al., 2020	2	2	2	2	1	3	2
Qin et al., 2020	2	2	2	2	1	3	2

Qin et al., 2021	2	2	1	2	1	3	2
Qin et al., 2021	2	2	2	3	1	1	2
Quisi et al., 2020	2	2	1	2	1	3	2
Raimondi et al., 2021	2	2	2	3	1	3	3
Ramaswamy et al., 2020	2	2	3	3	1	3	3
Ramos-Rincon et al., 2020	2	2	1	3	1	3	3
Rastad et al., 2021	2	2	2	3	2	3	3
Aggarwal et al. 2020	2	2	1	2	1	3	2
Al-Rousan, 2020	1	3	3	2	3	2	3
Bezzio et al. 2020	1	2	3	2	2	3	3
Bi et al. 2020	1	2	1	1	1	1	1
Bi et al. 2020	2	2	3	2	1	3	3
MMWR, 2020	1	3	3	2	1	2	3
MMWR,2020	1	3	3	2	1	2	3
Chen et al. 2020	2	2	1	2	2	1	1
Colaneri et al. 2020	2	2	3	3	1	1	3
Deng G et al., 2020	1	3	3	3	3	2	3
Deng et al, 2020	2	2	1	3	1	1	2
Du et al. 2020b	2	2	1	2	1	1	1
Du et al., 2020a	2	2	3	2	1	3	3
Dudley et al. 2020	1	3	3	3	3	3	3
Feng et al. 2020	2	2	1	2	2	1	1
Gong et al., 2020	2	2	3	3	2	3	3
Grasselli et al.2020	2	2	1	2	2	3	2

Guan et al., 2020a	1	2	1	2	2	3	2
Guan et al, 2020b	2	2	3	2	2	3	3
Guo et al., 2020	1	2	1	1	2	3	2
Han et al., 2020	1	2	1	3	1	3	3
Hu et al., 2020	1	2	1	3	1	1	3
Ji et al., 2020	1	2	3	2	1	1	2
Kalligeros et al., 2020	2	2	1	1	1	3	2
Lei et al., 2020	1	2	1	1	1	1	1
Leung et al., 20202	2	2	1	1	3	2	2
Li et al., 2020	2	2	1	2	1	3	2
Li et al., 2020	2	2	1	3	1	3	3
Lian et al., 2020	1	2	3	1	1	3	3
Liu et al., 2020	2	2	1	1	1	1	1
Mehta et al., 2020	2	2	1	2	1	3	2
Middeldrop et al., 2020	2	2	1	2	2	1	1
Nikpouraghdam et al., 2020	2	2	1	1	1	3	2
Pei et al., 2020	2	2	1	2	1	1	1
Pereira et al., 2020	2	2	1	3	1	1	2
MMWR, 2020	1	3	3	2	1	3	3
Qin et al., 2020	2	2	1	2	1	3	2
Qu et al., 2020	2	3	1	3	1	2	3
Richardson et al., 2020	2	3	3	2	1	2	3
Simonnet et al., 2020	2	2	1	3	1	3	3
Sun et al., 2020	2	3	3	2	1	2	3

Sun et al., 2020	2	2	1	3	1	2	2
Tang et al., 2020	2	2	1	3	1	1	2
Tedeschi et al., 2020	2	2	1	3	3	3	3
Tian et al., 2020	2	2	3	2	1	1	2
To et al., 2020	2	2	3	2	1	3	3
Verity et al., 2020	2	3	1	3	3	2	3
Wan et al., 2020	2	2	3	3	1	1	3
Wang et al., 2020	2	2	1	3	1	1	2
Wang et al., 2020	2	3	1	3	1	2	3
Wang et al., 2020	2	2	1	2	1	1	1
Wei et al, 2020	2	2	1	1	1	3	2
Wu et al.,2020	2	2	1	3	1	1	2
Yan et al., 2020	2	2	1	3	1	1	2
Yang G et al., 2020	2	2	1	2	1	1	1
Yang R et al., 2020	2	2	3	3	1	1	3
Yang X et al., 2020	2	3	1	2	1	2	2
Yao et al., 2020	2	2	1	2	1	1	1
Zhang R et al., 2020	2	2	3	2	1	3	3
Zou et al.2020	2	2	3	3	1	1	3
Zhang et al. 2020	2	2	3	3	1	3	3
Zhang et al. 2020	2	2	1	3	1	3	3
Zhang et al.2020	2	1	1	3	1	2	2
Zhang et al. 2020	2	2	3	3	1	3	3

Yu et al. 2020	1	3	1	2	1	2	2
Hu et al., 2020	2	2	1	1	1	1	1
Shi et al., 2020	2	3	1	3	3	3	3
Chen R et al., 2020	1	2	1	1	1	1	1
Dong Y et al., 2020	2	2	3	3	3	3	3
MMWR 2020	1	3	3	2	1	2	3
Tao et al. 2020	2	2	3	2	1	2	2
Tatum et al. 2020	2	3	3	2	3	2	3
Tehrani et al. 2021	2	1	2	2	3	1	2
Telle et al. 2021	1	3	2	2	3	2	3
Temkin-Greener et al. 2020	1	3	3	2	3	2	3
Tesoriero et al. 2020	1	2	1	2	3	2	2
Tessitore et al. 2021	2	2	2	2	3	2	2
Recinella et all., 2020	2	3	3	3	1	2	3
Ren et all., 2020	2	2	3	3	1	2	3
Rieg et all., 2020	2	2	2	2	1	1	2
Rivera-Izquierdo et al., 2020	2	2	2	3	1	1	2
Rivinius et al., 2020	2	2	3	2	2	1	3
Roberts et al., 2020	2	2	3	3	2	2	3
Gostner et al., 2020	2	3	3	3	1	2	3
Rodilla et al., 2021	2	2	1	2	1	1	1
Rodriguez et al., 2020	2	2	3	2	1	1	2
Diaz et all., 2020	2	3	1	2	1	1	2
Molinero et al., 2020	2	2	1	2	1	1	1

Roedl et al., 2020	2	2	1	2	2	1	1
Rokni et al., 2020	2	2	2	2	1	1	1
Rosenthal et al., 2020	1	2	1	2	1	1	1
Rossi et al., 2020	2	2	3	2	2	1	2
Rottoli et al., 2020	2	2	1	2	3	1	2
Russo et al., 2020	2	3	1	3	3	1	3
Ruthrich et al., 2021	2	2	1	2	2	1	1
Rutten et al., 2020	2	2	2	3	1	2	2
Saand et al., 2020	2	3	2	2	1	2	2
Sadeghi et al., 2020	3	3	3	2	1	1	3
Saeed et al., 2020	3	2	1	2	2	1	2
Saez-Giménez et al.,	2	2	3	2	2	1	2
Saggi et al., 2020	2	3	2	3	1	1	3
Saha et al., 2021	2	3	3	3	1	1	3
Sai et al., 2021	2	2	3	2	1	1	2
Saifi et al., 2021	2	2	2	3	2	1	2
Salazer et al., 2021	2	2	1	2	1	1	1
Saleh et al., 2021	2	2	3	2	1	1	2
Sands et al., 2020	2	2	1	3	1	1	2
Sano et al., 2020	3	2	3	3	2	1	3
Santos et al., 2020	3	2	1	3	2	3	3
M. Santos et al., 2020	2	2	1	3	1	1	2
Santus et al., 2020	2	2	2	2	2	3	2
Sapey et al., 2020	2	2	1	2	2	2	2

Scarfo et al., 2020	3	2	3	3	2	3	3
Schultze et al., 2020	2	2	1	2	1	3	2
Wang et al., 2021	2	2	2	1	1	1	1
Y.Wang et al., 2020	2	2	3	1	2	1	2
Yao et al., 2021	2	2	3	2	3	1	3
Zhou et al., 2020	3	2	1	1	1	1	2
Ye et al., 2020	2	2	3	3	3	1	3
Wang Z et al, 2020	2	2	2	3	2	1	2
Wargny et al., 2021	2	2	2	2	2	3	2
Webb et al., 2020	3	2	1	1	1	2	2
Wei et al.,2020	2	2	3	1	2	1	2
Wei X et al., 2020	2	2	3	3	2	1	3
Wei Z et al, 2021	2	2	1	2	2	1	1
Wen et al., 2021	2	2	1	1	1	1	1
Williams et al., 2021	2	2	1	2	2	1	1
Betech et al., 2020	2	2	3	3	1	1	3
Wu et al., 2020	3	2	3	2	1	1	3
Wu, F et al., 2020	3	2	1	2	2	1	2
Wu, M et al., 2020	2	2	2	2	2	1	1
Xie et al., 2020	2	2	1	2	2	1	1
Xie,Y et al., 2020	3	2	3	3	3	1	3
Xiong et al., 2020	3	2	3	3	1	1	3
Xiong, S et al., 2020	3	2	3	3	1	1	3
Xu B. et al., 2020	2	2	3	3	1	3	3

Xu Jingyuan. et al., 2020	2	2	3	2	2	3	3
Xu Jiqian et al., 2020	2	2	3	2	2	1	2
Xu Peng et al., 2020	2	2	3	2	1	3	3
Yan Chengxi et al., 2021	2	2	2	2	2	3	2
Yan Q. et al., 2020	2	2	1	2	2	1	1
Yang C. et all., 2021	2	2	2	2	2	1	1
Yang D. et al., 2020	2	2	1	2	2	1	1
Yang G. et al., 2020	2	2	1	2	1	1	1
Yang K. et al., 2020	3	2	2	1	1	1	2
Yang Q. et al., 2020	2	2	3	3	2	3	3
Yang X. et al., 2020	2	2	2	3	1	1	2
Yarza et al., 2020	3	2	1	3	2	1	3
Yayla et al., 2020	2	2	2	3	2	1	2
Yehia et al., 2020	2	2	1	2	3	1	2
Yu Caizheng et al., (2020)	2	2	1	3	2	1	2
Yu Y. et al., 2020	2	2	1	2	2	1	1
Yue et al., 2021	3	2	2	2	2	1	2
Zachariah et al., 2020	3	2	3	2	2	1	3
Ziener et al., 2020	2	3	1	3	2	1	3
Zeng H. et al., 2020	2	2	1	2	2	1	1
Zeng Z. et al., 2020	2	2	1	3	1	1	2
Zhang Hongmei et al., 2020	3	2	2	2	2	1	2
Zhang Hongyan et al., 2020	3	2	2	2	2	1	2

Zhang Jing et al., 2020	2	2	3	1	1	1	2
Zhang Lin et al., 2020	2	2	1	2	3	1	2
Zhang L. el al., 2020	3	2	1	2	1	1	2
Zhang Lijuan et al., 2020	2	2	1	1	1	1	1
Zhang Nan et al., 2020	3	2	1	1	1	1	2
Xu Xunhua et al., 2020	3	2	3	2	2	1	3
Zhang Nannan et al., 2021	3	2	3	2	1	3	3
Zhang Q. et al., 2020	3	2	3	3	2	1	3
Zhang S. et al.,2020	2	2	2	2	2	1	1
Zhang W. et al., 2021	3	2	1	2	2	1	2
Zhang Y. et al., 2021	2	2	3	2	1	1	2
Zhao J. et al., 2021	2	2	3	2	2	1	2
Zhao M., et al., 2020	2	2	3	3	3	2	3
Zhao Y. et al., 2020	2	2	3	2	1	1	3
Zheng B. et al., 2021	3	2	1	2	2	1	2
Zheng T. et al., 2020	2	2	2	3	2	1	2
Zhong Y. et al., 2020	3	2	1	3	2	1	3
Zhou Jun et al., 2021	2	2	3	3	2	3	3
Zhou J. et al., 2020	2	2	1	3	2	3	3
Zhou S. et al., 2020	2	2	1	3	2	1	2
Zhou Wei et al., 2021	2	2	2	1	1	1	1
Zhou Yiwu et al., 2020	2	2	3	3	1	1	3
He Y. et al., 2020	1	2	3	1	1	1	2
Zhu B. et al., 2020	2	2	1	1	1	1	1

Zhu Y. et al., 2021	3	2	2	3	2	1	3
Zieleskiewicz et al., 2020	2	2	3	2	2	1	2
Zou X. et al., 2020	2	2	1	3	2	1	2
Zuniga et al., 2021	2	2	1	3	1	1	2
Zuo P. et al., 2020	3	2	1	2	1	1	2
Thompson et al., 2020	2	2	2	2	2	1	1
Tian et al., 2020	2	2	1	2	2	1	1
Tobolowsky et al., 2021	3	2	3	3	2	1	3
Toh et al., 2021	3	2	3	3	2	1	3
Tomasoni et al., 2020	2	2	1	3	2	1	2
Tong et al., 2020	3	2	1	3	1	1	3
Albalawi et al., 2021	2	2	2	2	2	1	1
Trifan et al., 2020	3	2	1	3	2	1	3
Argirios et al., 2020	3	2	3	2	1	1	3
Tu et al., 2021	2	2	3	2	1	1	2
Yildirim et al., 2021	2	2	3	2	2	1	2
Turgutalp et al., 2021	2	2	3	2	1	1	2
Haq et al., 2020	2	2	1	2	1	1	1
Vanhems et al., 2021	2	2	1	2	2	1	1
Venturini et al., 2021	2	2	3	3	2	1	3
Zertuche et al., 2021	2	2	1	3	2	1	2
Verma et al., 2021	2	2	2	2	2	1	1
Llamas et al., 2021	2	2	2	2	2	1	1

Villa et al., 2021	2	2	3	3	2	1	3
Vizcarra et al., 2020	2	2	2	2	2	1	1
Vrillon et al., 2021	2	2	3	2	2	1	2
Vrotsou et al., 2021	2	2	3	2	2	1	2
Vu et al., 2020	3	2	3	2	1	1	3
Wang F et al., 2020	2	2	1	1	1	1	1
Wang Fang et al., 2020	2	2	1	2	1	1	1
Wang Gang et al., 2021	1	2	1	1	1	1	1
Wang Guyi et al.,	2	2	1	2	2	1	1
Wang Juan et al., 2020	2	2	3	2	1	1	2
Wang Junhong et al., 2020	2	2	3	3	2	1	3
Wang Lang et al., 2020	2	2	3	1	1	1	2
Wang Peng et al., 2020	3	2	3	1	2	1	3
Wang Sijiao et al., 2020	2	2	3	2	2	1	2
Wang Sufei et al., 2020	2	2	3	3	2	1	3
Wang Tao et al., 2020	2	2	2	2	2	1	1
Wang Wen et al., 2021	2	2	3	2	1	1	2
Wang Wenjun et al.,2021	2	2	1	2	2	1	1
Wang X. et al., 2020	2	2	3	2	2	1	2
Seo et al., 2021	2	2	1	1	1	1	1
Shah et al., 2020	3	2	3	3	2	1	3
Shahriarirad et al, 2020	3	2	3	1	2	1	3
Shao et al., 2020	3	2	3	2	1	1	3

Stringer et al., 2021	2	2	1	2	3	1	2
Sharma et al., 2021	3	2	2	3	1	1	3
Shi J et al., 2020	2	2	1	2	2	3	2
Shi M et al, 2020	2	2	3	3	2	1	3
Shields et al., 2020	3	3	3	3	3	3	3
Siepmann et al,. 2021	2	2	2	2	1	1	1
Singh B et al., 2020	2	2	1	2	1	1	1
Singh S et al., 2020	2	2	3	2	1	1	2
Singh Shailendra et al., 2020	2	2	2	2	2	1	1
Singh Shailendra et al., 2020	2	2	3	2	2	1	2
Smati et al., 2020	2	2	1	1	1	1	1
Smith A et al., 2020	2	2	3	3	2	1	3
Smith M et al., 2020	2	2	3	3	1	1	3
Snipelisky et al., 2020	3	2	3	1	1	1	2
Soh et al., 2020	2	2	2	2	1	1	1
Soleimani, 2020	2	2	1	3	1	1	2
Song Jia et al., 2020	3	2	3	1	2	1	3
Song Jin-Wen 2020	3	2	3	2	1	1	2
Stachura et al., 2021	3	2	1	2	2	1	2
Stall et al., 2020	2	2	1	1	2	1	2
Stefan et al., 2021	3	2	1	3	2	1	3
Steinberg et al., 2020	2	2	3	3	2	1	3
Sulyeman et al., 2020	2	2	1	1	1	1	1
Sun et al., 2020	2	2	3	2	2	1	2

Suresh et al., 2021	2	2	1	2	1	1	1
Swan et al., 2020	2	2	3	2	2	3	3
Szymanski et al., 2021	2	2	3	2	1	2	2
Talavera et al., 2020	2	2	1	2	1	1	1
Tang et al., 2020	2	2	2	3	2	1	2
Botero et al, 2020	2	2	2	3	2	1	2
Zhao X. et al, 2020	2	2	1	1	1	1	1
