# Supplementary material for: Heterogeneity and Risk of Bias in Studies Examining Risk Factors for Severe Illness and Death in COVID-19: A Systematic Review and Meta-Analysis
Source: Pathogens. 2022 May 10;11(5):563. doi: 10.3390/pathogens11050563 (PMC9147100; doi:10.3390/pathogens11050563)
Supplement: Supplementary file 1 [file pathogens-11-00563-s001.zip › Table S3.pdf]

Heterogeneity and risk of bias in studies examining risk factors for severe illness and death in COVID-19: a systematic review and meta-analysis

Abraham Degarege [*], Zaeema Naveed, Josiane Kabayundo, David Brett-Major

Table S3: Characteristics of the studies

				Outcomes (95% CI)		
**References (Study area)**	**Age in years**	**Sample size**	**Study design**	**Death *vs* survival**	**Severe illness *vs* mild/moderate**	**Admission in ICU *vs* not in ICU**
Aggarwal et al. 2020 (USA)	38-95 mean=65.5 median=67	16	CS			○ Male 0.23, 0.02 – 3.01<br>○ Obese 2.77, 0.37 – 21.02<br>○ Hypertension 0.2, 0.02 – 1.71<br>○ Diabetic 0.55, 0.06 – 4.75<br>○ Coronary artery 2.33, 0.16-32.58<br>○ Cerebrovascular 1, 0.05 – 19.36<br>○ Congestive cardiac failure<br>○ 4.2, 0.33 – 53.12<br>○ Chronic kidney 5, 0.58-42.79<br>○ COPD 1, 0.05 – 19.36<br>○ Cancer 2.33, 0.16 – 32.58
Al-Rousan, 2020 (South Korea)	Not provided	2765	CS	○ Males 1.14, 0.97 – 1.35		
Bezzio et al. 2020 (Italy)		79	P	○ Age>65 19.6, 2.95 – 130.6<br>○ Charlson comorbidity index (CCI) >1 16.66, 1.80 – 153.9	○ Age>65 5.87, 1.15 – 29.66<br>○ Carlson comorbidity index CCI>1 2.9 1.06 – 9.21	
Bi et al. 2020 (china)	Mean 45	391	R		○ Male 2.5, 1.1-6.1	
Bi et al. 2020 (China)	Median 46	113	R		○ Age>50 5.42, 1.92 15.26<br>○ Male 1.13, 0.44-2.91<br>○ Smoker 8.6, 1.88-39.55<br>○ Comorbidity 2.21, 0.86-5.68	
MMWR, 2020 (APRIL3, USA)	All ages	6637	CS			○ Diabetes 1.49, 1.17-1.91<br>○ Chronic lung disease 1.50,1.13-2.00<br>○ Cardiovascular disease 1.33,1.04-1.71<br>○ Immunosuppression 1.52, 1.01-2.29<br>○ Chronic renal disease 1.38,0.97-1.96<br>○ Pregnancy 0.28, 0.10-0.81<br>○ Chronic liver disease 1.77, 0.65-4.80

						o Other comorbidities 1.11, 0.88-1.40<br>o Smoking 0.51, 0.19-1.35
MMWR,2020 (APRIL14, USA)	All ages	8945	CS	o Age>65 9.94, 4.53-21.8		o Age>65 1.81, 1.14-2.84
Chen Q et al. 2020a china)	Mean=47.5<br>Sd= 14.6	145	R		o Continuous age<br>o Median severely ill (52.8 year)<br>o Median non-severely ill (45.3)<br>o P value=0.0001<br>o Males 0.99, 0.46-1.93<br>o Hypertension, 1.81, 0.71-4.62<br>o Diabetes 2.63, 0.86-8.05<br>o Chronic liver disease 5.12, 0.90-29.14<br>o Chronic kidney disease 1.19, 0.11-13.48<br>o Smoking 0.59, 0.15-2.10	
Colaneri et al. 2020 (Italy	Median= 67.5<br>Range 10-94	44	R		o Male 2.6, 0.67-10.06<br>o Age>65 4.06, 1.04-15.72<br>o Other comorbidities 2.6, 0.67-10.06<br>o Cancer 3.8, 0.62-23.85<br>o Heart disease 2.4, 0.59-9.63<br>o Hypertension 0.70, 0.19-2.61<br>o Diabetes 0.21, 0.02-2.00	
Deng G et al., 2020 IChina)		44,672	CS	o Males 1.6, 1.48-1.92<br>o Age>=60 9.98, 8.53-11.69<br>o Hypertension 2.87,2.39-3.45<br>o Diabetes 3.54, 2.71-4.48<br>o Cardiovascular disease 5.00 3.96-6.31<br>o Respiratory disease 2.58, 1.78-3.72<br>o Cancer 2.23, 0.97-5.11		
Deng Q et al, 2020 (China)	Median 65<br>Range 24-92	112	R		o Continuous age<br>o Severe median=68<br>o Non-severe median=56<br>o P value <0.01<br>o Male1.79 0.83-3.84<br>o COPD 2.06 0.21-20.48<br>o Hypertension1.53, 0.67-3.51<br>o Diabetes 2.11, 0.70-6.35<br>o Cardiovascular disease	

					1.73,0.51-5.81	
Du et al. 2020<br>(China)	Mean<br>57.6 ±<br>13.7<br>Range<br>18-87	179	P	o Age >=65 3.76, 1.14-17.39<br>o Cardiovascular disease 2.46, 0.75 – 8.04<br>o Hypertension 4.08,1.58-10.5<br>o Male 0.74, 0.29-1.84<br>o Diabetes 1.94, 0.69-5.45<br>o Cancer 2.58, 0.25-26.04		
Du et al., 2020a<br>(China)	Mean<br>70.7	109	R		o Age >=65 0.84, 0.34-2.03<br>o Male 1.26, 0.56, 2.84<br>o Hypertension 0.80, 0.37-1.73<br>o Cardiovascular disease 0.68, 0.30-1.52<br>o Diabetes1.43, 0.63-3.22<br>o Chronic respiratory disease 0.29, 0.08-0.97	
Dudley et al. 2020<br>China, South Korea)		52427	CS	o Sex specific case fatality rate in china<br>o Males 2.8%<br>o Females 1.7%<br>o Sex specific case fatality rate in Korea<br>o Males 1.19%<br>o Female 0.52%		
Feng et al. 2020<br>(China)	Median<br>53<br>IQR 40-64	476	CS		o Age >=65 2.17,1.39-3.40<br>o Male1.60, 1.04 – 2.45<br>o Smoker 1.85, 0.97-3.53<br>o Other comorbidity 2.18, 1.37-3.47<br>o Hypertension1.82, 1.15-2.87<br>o Cardiovascular disease 2.50,1.27-4.9<br>o Diabetes 1.58, 0.84-2.97<br>o Cancer 4.15, 1.29-13.33<br>o Cerebrovascular disease 3.36,1.28-8.92<br>o Immunosuppression 7.35, 1.40-39.4[illegible]<br>o COPD 5.47, 2.23-13.39<br>o Chronic renal 2.86, 0.39-20.58	
Gong et al., 2020<br>(china)	Median<br>49	189	R		o Median age for severe 63.5<br>o Median age for non-severe 45<br>o P value <0.01<br>o Male 1.64, 0.73-3.70	

Grasselli et al.2020<br>(Italy)	Median<br>63<br>IQR 56-<br>70	1591	R	o Age >60 3.29, 2.52-4.30<br>o Hypertension 2.24,1.70-2.95		
Guan et al., 2020a<br>(China)	Mean<br>48.9	1590	CS	o COPD 11.53, 4.36-30.45<br>o Diabetes 4.27, 2.20-8.26<br>o Hypertension 6.86, 3.85-12.19<br>o Cardiovascular disease 5.56, 2.48-12.45<br>o Cerebrovascular disease 8.61, 3.35-22.12<br>o Cancer 5.40, 1.53-18.99<br>o Chronic renal disease10.58, 3.71-30.15	o COPD 9.25, 4.00-21.38<br>o Diabetes 3.16, 2.14-4.67<br>o Hypertension 3.38, 2.50-4.57<br>o Cardiovascular disease 2.84,1.62-4.96<br>o Cerebrovascular disease 5.52, 2.66-11.45<br>o Cancer 5.41, 2.12-13.78<br>o Chronic renal disease3.30, 1.35-8.0[illegible]<br>o Immunodeficiency 2.630.23-29.18	o COPD 6.59, 2.66-16.30<br>o Diabetes 2.95, 1.72-5.04<br>o Hypertension 3.39, 2.21-5.21<br>o Cardiovascular disease 2.48, 1.14-5.38<br>o Cerebrovascular disease 4.85, 2.03-11.61<br>o Cancer 6.04, 2.11-17.32
Guan et al, 2020b<br>(China)	Median<br>47	1099	R		o Age >=65 2.55, 1.72-3.79<br>o Male 0.97, 0.70-1.36<br>o Smoking 1.87, 1.24-2.81	
Guo et al., 2020<br>(China)	Median<br>59	24	R	o Diabetes 3.2, 0.81-12.58		
Han et al., 2020<br>(China)	Median:<br>58.95<br>±10.80<br>Severe<br>cases:<br>58.97 ±<br>14.38<br>Critical<br>Cases:<br>57.27 ±<br>17.25	273	R		o Male 0.94, 0.54-1.65	
Hu L et al., 2020<br>(China)	Median<br>61	323	R		o Age>=65 2.96, 1.81-4.83<br>o Male 1.13, 0.73-1.76<br>o Smoking 2.06, 1.0-4.2<br>o Hypertension 1.78, 1.11-2.88<br>o Diabetes 2.32, 1.19-4.53	

Ji D et al., 2020 (China)	Mean 44 ±16.3	208	R		○ Age<br>○ Severe mean 57.7<br>○ Non-severe mean 40.7<br>○ P value <0.001<br>○ Age (category)>65 9.75, 4.20-22.63<br>○ Male 2.07, 0.98-4.34<br>○ Smoking 2.10, 0.74-5.92	
Kalligeros et al., 2020 (China)	Median 60	103	R			○ Continuous age 1.03, 1.0-1.07<br>○ Non-Hispanic black 0.80, 0.26-2.45<br>○ Male 2.40, 0.87-6.64<br>○ Obesity 5.39,1.13-25.64<br>○ Diabetes 1.91, 0.71-5.19<br>○ Hypertension 0.79, 0.27-2.28<br>○ Lung disease 1.50, 0.47-4.82<br>○ Cardiovascular disease 1.52, 0.47-4.82
Lei et al., 2020 (China)	Median 56	5771	CS		○ Age continuous<br>○ Severe median 59<br>○ Non-severe median 55<br>○ P value <0.001<br>○ Male 0.67, 0.59-0.76<br>○ Chronic liver disease 1.74, 1.08-2.80	
Leung et al., 20202 (China)		154	CS	○ Age continuous 1.04, 1.00-1.10<br>○ Male 1.19, 0.62-2.26		
Li K et al., 2020 (China)		83	CS		○ Age continuous<br>○ Severe mean age 25<br>○ Non severe mean age 41.9<br>○ P value <0.001<br>○ Male 1.5, 0.57-3.88<br>○ Hypertension 1.59, 0.24-10.18	
Li X et al., 2020 China)	Median 60 IQR 48-69	548	R		○ Age>=65 2.24, 1.94-2.78<br>○ Male 1.60, 1.14-2.24<br>○ Smoking1.38, 0.88-2.17<br>○ Chronic lung disease 3.49, 1.12-10.8<br>○ Diabetes 1.91,1.18-3.1<br>○ Hypertension 2.20, 1.51-3.20<br>○ Chronic kidney disease 1.56, 0.43-5.60	
Lian et al., 2020 (China)		788	R		○ Age>=60 4.60, 2.80-7.55	○ Age>=60 7.55, 3.15-18.05

Liu Y et al., 2020 China)	Mean 53.95 (16.90)	245	R	o Age continuous 1.09, 1.06-1.13<br>o chronic lung disease 4.14, 0.94-18.22<br>o Hypertension 3.94, 1.82-8.53<br>o Diabetes 3.30, 1.24-8.77<br>o Cancer 1.89, 0.38-9.51<br>o Chronic liver disease 2.67, 0.50-14.37<br>o Smoking 2.93, 0.72-11.94		
Mehta et al., 2020 (USA)	Median 69	218	CS	o Males 1.04, 0.57-1.90<br>o Age<br>o Survived median 66<br>o Died median 76		
Middeldrop et al., 2020 (Netherlands)	Mean 61 ± 14	198	R			o Age<br>o Mean for intensive care=62<br>o Mean for non-intensive= 60<br>o P value 0.28<br>o Male 2.41, 1.26-4.62<br>o Cancer 1.23, 0.26-5.69
Nikpouraghdam et al.,2 020 (Iran)	Mean 55.5 ± 15.15	2968	R	o Age>=60 3.95, 2.98-5.23<br>o Male1.45,1.08 - 1.96<br>o Diabetes 1.35, 0.71-2.55<br>o Chronic respiratory disease 2.23, 1.08-4.59<br>o Hypertension 1.97, 0.92-4.21<br>o Cardiovascular disease 1.51, 0.53-4.30<br>o Chronic renal disease 2.49, 0.71-8.69		
Pei et al., 2020 (China)	Mean 56.3	333	CS		o Male 1.78, 1.15-2.76<br>o Hypertension 1.70, 1.05-2.73<br>o Diabetes 2.61, 1.48-4.59	
Pereira et al., 2020 (USA)	Median 57	90	R		o Age >60 1.02, 0.40-2.55<br>o Hispanic 0.68, 0.27-1.68<br>o Hypertension 1.66, 0.64-4.38<br>o Diabetes 1.43, 0.58-3.54<br>o Chronic Lung disease 1.35, 0.44-4.1[illegible]<br>o Chronic kidney disease 1.56, 0.59-4.11	
MMWR, 2020 (USA)		7,162	CS			o Age>=65 1.20, 0.95-1.51

Qin C et al., 2020 (China)	Median 58	452	R		o Male1.27, 0.86-1.86<br>o Smoking 0.42, 0.09-1.94<br>o Chronic lung disease 1.76, 0.47-6.61<br>o Hypertension 2.62, 1.65-4.17<br>o Cardiovascular disease 4.97, 1.47-19.79<br>o Cerebrovascular disease 1.56, 0.40-5.97<br>o Chronic liver disease 0.57, 0.11-2.88<br>o Diabetes 1.48, 0.86-2.55<br>o Cancer 1.46, 0.45-4.75<br>o Chronic kidney disease 0.86, 0.24-3.12	
Qu et al., 2020 (China)	Median 20.5	30	CS		o Age continuous<br>o Median age severe=60<br>o Median age non-severe 49.44<br>o P value 0.041	
Richardson et al., 2020 (USA)	Median age 63 IQR 52-75	5700	CS	o Age >=60 4.14, 3.27-5.24<br>o Male 1.23, 1.01-1.49		
Simonnet et al., 2020 (France)	Median 60 IQR 51-70	124	R			o Age continuous1.00, 0.97-1.04<br>o Male 2.83, 1.02-7.85<br>o Diabetes 1.60, 0.44-6.83<br>o Hypertension 2.29, 0.89-5.84
Sun L et al., 2020 (China)	Median 44 IQR 34-56	55	CS		o Age >65 21.71, 3.78-124.54<br>o Male 0.84, 0.25-2.78<br>o Hypertension 12.6, 2.18-73.4<br>o Diabetes 1.89, 0.28-12.65	
Sun S et al., 2020 (China)	Median 50 IQR 41-57	116	R		o Age continuous<br>o Median non-severe<br>o 47<br>o Median severe 62<br>o P value <0.001<br>o Male 2.23, 0.90-5.51	o Age continuous<br>o Median non-severe 57<br>o Median 71<br>o P value 0.008<br>o Male 1.00, 0.18-5.45

Tang et al., 2020 (China)	Mean 65.1 ± 12.0	449	CS	o Age continuous<br>o Mean survivors 63.7<br>o Mean non-survivors 68.7<br>o Pvalue= <0.001<br>o Male 1.57, 1.03-2.40		
Tedeschi et al., 2020 (Italia)		609	R	o Hypertension 4.31, 2.91-6.39		
Tian et al., 2020 (China)	Median 47.5	262	R		o Age >=65 5.16, 2.55-10.45<br>o Male1.48, 0.77-2.81	
To et al., 2020 (China)	Median age 62	23	R		o Male1.28, 0.24-6.83<br>o Hypertension 3.66, 0.51-26.2<br>o Diabetes 1.37, 0.15-11.93	
Verity et al., 2020 (China)		45,695	CS	o Age >=60 9.98, 8.53-11.69		
Wan et al., 2020 (China)	Median 47	135	CS		o Age continuous<br>o Median mild 44<br>o Median severe 56<br>o P value <0.0001<br>o Male 0.91, 0.43-1.91<br>o Smoking 1.19, 0.11-13.53<br>o Diabetes 8.90, 2.26-34.9<br>o Hypertension 1.06, 0.30-3.67	
Wang D et al., 2020 China)	Median 51 IQR 36-65	107	R	o Age >=60 18.12, 4.79-68.57<br>o Male 6.11, 1.66-22.48<br>o Hypertension 5, 1.74-14.30<br>o Cardiovascular disease 7.97, 2.29-27.75<br>o Diabetes 4.88, 1.31-18.18<br>o Chronic liver disease 0.94, 0.10-8.57<br>o Cerebrovascular diseases 5.31, 0.98-28.71		
Wang J et al., 2020 (China)	Mean 52.1 Sd 18.07	93	CS		o Age >60 6.21, 2.11-18.23<br>o Male 1.62, 0.56-4.71	

Wang L et al., 2020 (China)	Mean 71+-8	393	P	o Age continuous<br>o Survived median 68<br>o Died median 76<br>o P value <0.001<br>o Male 1.73, 1.00-3.00<br>o Hypertension 1.53, 0.89-2.64<br>o Diabetes 1.09, 0.52-2.26<br>o Cardiovascular disease 3.60, 1.90-6.82<br>o Cerebrovascular disease 4.34, 1.75-10.73<br>o Chronic kidney disease 1.93, 0.57-6.47<br>o COPD 5.37, 2.17-13.29<br>o Cancer 1.05, 0.28-3.85		
Wei et al, 2020 (China)	Mean 64.8 ± 13.3	252	R		o Male 1.73, 1.05-2.85<br>o Diabetes 1.01, 0.47-2.15<br>o Hypertension 2.10, 1.22-3.60<br>o Cardiovascular disease<br>o 3.56, 1.25-10.13	
Wu J et al.,2020 (China)	Mean 43.12 SD 10.02	280	R		o Age ≥65 12.75. 6.74-24.1<br>o Male 1.02, 0.60-1.70	
Yan Y et al., 2020 (China)	Median 64 (IQR 49–73)	193	R	o Age continuous<br>o Survivor median 46<br>o Non-survivor median 70<br>o P value <0.01<br>o Diabetes 4.77, 2.15-10.56<br>o Male 2.93, 1.62-5.32<br>o Diabetes 2.83, 1.42-5.<br>o Hypertension 4.81, 2.48-9.35<br>o Cardiovascular disease 6.75, 2.25-20.16<br>o Chronic respiratory disease 3.09, 0.83-11.48	o Diabetes 4.77, 2.15-10.56	o Diabetes 2.83, 1.42-5.62
Yang G et al., 2020 (China)		462	R	o Hypertension 1.68, 0.67-4.21	o Hypertension 1.13, 0.65-1.98	

Yang R et al., 2020 (China)	Median 55.6 IQR=40-67	212	R	o Age ≥65 5.91, 2.46-14.19<br>o Male 6.16, 2.03-18.65		
Yang X et al., 2020 (China)	Median 57 IQR 47-67	1476	CS	o Continuous age<br>o Non survivors mean 67<br>o Survivors mean 56<br>o Male 1.89, 1.41-2.53		
Yao et al., 2020 (China)	Median 52 IQR 37-58	108	R	o Age>65 7.72, 2.11-28.18<br>o Male 2.33, 0.68-7.9<br>o Hypertension 13.53, 3.55-51.5		
Zhang R et al., 2020 (China)	Mean 45.4 SD 15.6	120	R		o Age continuous 1.1, 1.0-1.1<br>o Male 1.52, 0.65-3.55<br>o Hypertension 10.70, 3.56-32.12<br>o Cardiovascular disease 4.3, 1.07-17.[illegible]<br>o Malignancy 8.8, 1.61-48.12	
Zou Y et al.2020 (China)		303	CS		o Male 3.36, 1.31-8.61<br>o Age: Mild (50 years), severe (65), P[illegible] < 0.001<br>o Chronic underlying disease 4.8, 1.95[illegible] 11.82<br>o Smokers 1.84, 0.39-8.7	
Zhang J et al. 2020 (China)	38-91	19	R	o Hypertension 0.72, 0.11-4.62<br>o Diabetes 0.17, 0.01-2.04<br>o Cerebrovascular disease 0.[illegible] 0.02-4.06		
Zhang Y et al. 2020 (China)	20-86	115	R		o >=60 years 11.47, 4.40-29.88<br>o Male 3.45, 1.46-8.17	
Zheng KI et al.2020 (China)	32-81	111	R		o Male 24.8, 1.8−342.1<br>o Comorbidities 52.6, 3.6−776.4<br>o Age as a continuous P<0.001<br>o Hypertensive 22, 6.03-80.3<br>o Cardiovascular disease 11.5, 0.98-134.4	

					○ Chronic obstructive pulmonary disease: 2.68 CI: 0.23-31.20<br>○ Diabetes 17.6, 4.84- 63.98	
Zhang G et al. 2020 (China)	23-88	95	R		○ Age 40–60 years 0.54 CI: 0.23-1.28<br>○ Male 1.85 CI: 0.77-4.46	
Yu et al. 2020 (China)	0-88 years	333	CS		○ <60 years10.79, 3.94- 29.53<br>○ Male 4.6, 1.7-12.6)<br>○ Heart disease 4.2, 1.2-14.2<br>○ Diabetes 1.1 CI: 0.3-3.6<br>○ Hypertensive 0.7, 0.2-2.0<br>○ Respiratory disease 2.0, 0.2-18.3<br>○ Smokers 0.89, 0.20 -4.01<br>○ Alcohol 0.62, 0.22-1.70	
Hu H et al., 2020 (China)	Mean 60 Sd 16.32	105	R	○ Male 2.21, 0.73-6.70<br>○ Age continuous<br>○ Mean non survivors=75<br>○ Mean survivors=57<br>○ P value<0.01<br>○ Hypertension 1.34, 0.45-3.96<br>○ Chronic pulmonary disease 4.0, 1.11-15.50		
Shi et al., 2020 (China)		487	CS		○ Age continuous<br>○ Mean severe=56<br>○ Mean non severe=45<br>○ P value <0.001<br>○ Male 2.66, 1.37-5.17<br>○ Smoking 1.60, 0.63-4.03<br>○ Hypertension 5.65, 3.05-10.45<br>○ Diabetes 3.15, 1.27-7.81<br>○ Cardiovascular disease 5.47, 1.54-19.41<br>○ Cancer 6.17 1.00-37.86<br>○ Chronic liver disease 0.88, 0.20-3.92<br>○ Chronic renal disease 3.68, 0.69-19.5	
Chen R et al., 2020 (China)	65 to 74 years	1590	R		○ Age≥65 (HR*, 3.43; 95% CI, 1.24-9.5),<br>○ Coronary heart disease (HR* 4.28; 95% CI, 1.14-16.13)<br>○ Cerebrovascular disease (HR* 3.1; 95% CI, 1.07-8.94)	

Dong Y et al., 2020 (China)	Median 7 years	2135	R		○ Children showed less severe COVID 19 illness than those of adult patients	
MMWR 2020 (USA)	All age	4,226	R	○ Age ≥ 65 vs <65= 8.84, 4.23- 18.48		Age ≥ 65 vs <65= 2.62, 1.81-3.78
Nepogodiev et al., 2020 (International, 24 Countries)	<29 - ≥70	1128	P	○ Age <50 vs ≥50 = 9.03, 4.56 - 17.89<br>○ Male 1.79, 1.35 – 2.39<br>○ Comorbidities 4.78, 2.29 – 9.94<br>○ Smoking 1.02, 0.64 – 1.63<br>○ Asthma 1.17, 0.69 – 1.97<br>○ Cancer 0.91, 0.63 – 1.32<br>○ Chronic kidney disease 1.74, 1.21 – 2.49<br>○ Chronic respiratory disease 1.85, 1.23 – 2.78<br>○ Cardiovascular disease 1.81, 1.13 – 2.89<br>○ Diabetes 1.15, 0.84 - 1.57<br>○ Hypertension 1.73, 1.31 – 2.29<br>○ Cerebrovascular disease 2.15, 1.37 – 3.37		
Abbas et al., 2020 (Iraq)	18-80 Mean 48.60	284	CS		○ Smoking 1.17, 0.65 – 2.07<br>○ Diabetes 1.97, 1.09 – 3.58	
Acar et al,. 2021 (Turkey)	>18 Mean 69 ± 14	709	R	○ Age ≥65 vs <65 = 4.77 – 2.90<br>○ Hypertension 2.16, 1.31 – 3.57<br>○ Diabetes 2.61 1.46 - 4.63		

				o Canver 11.96, 6.34 - 22.54<br>o Chronic respiratory disease 1.71, 0.85 - 3.41<br>o Cardiovascular disease 3.29, 1.85 - 5.85<br>o Chronic kidney disease 5.56, 2.53 - 12.21		
Biran et al., 2020 (USA)	≥18	764	R	o Age ≥65 vs <65 (HR*) 2.10, 1.68 – 2.62<br>o Male (HR*) 1.41, 1.13 – 1.72<br>o Diabetes (HR*) 1.16, 0.95 – 1.41)<br>o Chronic repiratory disease (HR*) 1.07, 0.83 – 1.06)<br>o Hypertension (HR*) 1.32, 1.06 – 1.65)		
Adamuz et al., 2020 (Spain)	Adult Median 66.5 IQR 51-77	1176	R	o Age ≥ 75 vs <75 = 3.04, 1.79 – 5.15<br>o Male 1.86, 1.11 – 3.11<br>o Comorbidities 2.01, 1.03 – 3.90	o Age ≥ 75 vs <75 = 1.52, 1.02 – 2.26)<br>o Male 1.20, 0.87 – 1.67<br>o Comorbidities 1.81, 1.26 – 2.59	
Agarwal et al., 2020 (USA)	>17 Mean 63.6 (62.9-64.3)	21,300	CS	o Cardivascular disease 2.0, 1.2 – 3.4		
Ahmad et al., 2020 (Pakistan)	6 months to 87 years	194	R	o Male 0.38, 0.14 – 1.05		

	Median 34 IQR 27-48					
Aiswarya et al., 2020 (India)	Mean 50.1 ± 12.2	48	P	o Age ≥50 vs <50 = 1.00, 0.24 – 4.02<br>o Male 0.28, 0.06 – 1.30<br>o Diabetes 2.57, 0.61 – 10.71<br>o Hypertension 0.61, 0.09 – 3.71	o Age ≥50 vs <50 =1.18, 0.37 - 3.71<br>o Male 0.82, 0.19 – 3.39<br>o Diabetes 1.3, 0.41 – 4.15<br>o Hypertension 0.95, 0.18 – 4.83	
Akbariqomi et al., 2020 (Iran)	Median 55 IQR 45–63 Range 22–94	595	R	o Diabetes 2.23, 1.30 – 3.81	o Male 3.05, 1.29 – 7.23	
Akhtar et al., 2021 (UK)	Not specified	293	R	o Age (continuous) 1.13, 1.07 – 1.16<br>o Male 0.85, 0.53 – 1.36<br>o Diabetes 0.90, 0.54 – 1.52<br>o Hypertension 1.20, 0.75 – 1.93<br>o Cancer 1.92, 1.04 – 3.55<br>o Stroke 1.81, 0.89 – 3.67<br>o Chronic kidney disease 0.77, 0.42 – 1.42		
Alahmari et al., 2021 (Saudi Arabia)	All ages	240474	CS	o Age≥60 vs <60 = 9.18, 8.40 – 10.03<br>o Male 1.52, 1.36 – 1.69		
Alasia et al., 2021 (Nigeria)	2-77 years	646	P	o Age >50 vs ≤50 = 10.81, 3.27 – 35.75<br>o Male 4.44, 0.57 – 34.41	o Age >50 vs ≤50 = 10.42, 5.90 – 18.42<br>o Male 1.95, 0.96 – 3.93	

	Mean 39.21 ± 12.31					
Albani et al., 2020 (Italy)	≥18	1403	P	○ Male 1.62, 1.23 – 2.12<br>○ Hypertension 0.89, 0.69 – 1.15<br>○ Diabetes 1.26, 0.95 – 1.69		
Alberici et al., 2020 (Italy)	Median 72 IQR 62-79	94	P	○ Age >70 vs ≤70 = 1.85, 0.75 – 4.78<br>○ Male 1.05, 0.41 – 2.78<br>○ Hypertension 0.27, 0.05 – 1.31<br>○ Diabetes 1.7, 0.69 – 4.20<br>○ Cardiovascular disease 3.11, 1.02 – 9.6<br>○ Chronic respiratory disease 1.07, 0.22 – 4.2<br>○ Cancer 1.5, 0.36 – 3.05		
Alfraij et al., 2021 (Kuwait and Saudi Arabia)	<18 years Median 2.78 IQR 0.21-8.51	25	R	○ Male 2.25, 0.19 – 25.36		
Algado-Sellés et al., 2020 (Spain)	Adults	1791	CS	○	○ Age≥60 vs <60 = 2.9, 0.9 – 9.3<br>○ Male 4.9, 1.8 – 13.6<br>○ Cardiovascular disease 2.3, 0.4 – 12.1<br>○ Hypertension 3.2, 0.7 – 13.5	
Aliberti et al., 2021 (Brazil)	Median 66	1428	P	○ Age ≥65 vs <65 = 2.27, 1.82 – 2.85<br>○ Male 1.25, 1.01 – 1.56		
Allard et al., 2020 (France)	Mean 61.8	108	R		○ Male 2.04, 0.85 – 4.85<br>○ Diabetes 1.38, 0.61 – 3.13	

	SD 15.8				○ Hypertension 1.44, 0.63 – 3.32<br>○ Smoking 1.95, 0.55 – 6.91<br>○ Alcohol 2.14, 0.71 – 6.48<br>○ Malnutrition 1.66, 0.77 – 3.56	
Allenbach et al., 2020 (France)	Median 77 Range 60-83	152	P		○ Male 1.34, 0.65 – 2.77<br>○ Age >60 vs ≤60 = 2.94, 1.19 – 7.26<br>○ Hypertension 1.05, 0.52 – 2.10<br>○ Cardiovascular disease 1.44, 0.65 – 3.20<br>○ Cancer 0.95, 0.39 – 2.27	
Almaghlouth et al., 2020 (USA)	Median 63 IQR 51-72	80	P	○ Age ≥65 vs <65 = 3.20, 0.58 – 17.60<br>○ Male 0.27, 0.05 – 1.53<br>○ Hypertension 0.68, 0.14 – 3.32<br>○ Diabetes 1.29, 0.27 – 6.24		
Aloisio et al., 2020 (Italy)	Median 61 IQR 50-73	427	R	○ Age (continuous) 1.14, 1.03 – 1.27		○ Age (continuous) 1.01, 0.98 – 1.02
Alqahtani et al., 2020 (Saudi Arabia)	Not sepcified	458	CS		○ Male 0.51, 0.24 – 1.09<br>○ Diabetes 18.45, 9.28 – 36.69<br>○ Hypertension 92.80, 39.26 – 219.36<br>○ Asthma 3.09, 0.95 – 10.01<br>○ Cardiovascular disease 4.51, 1.09 – 18.67	
Al-Salameh et al.,2020 (France)	Median 73	433	P	○ Diabetes 0.77, 0.44 – 1.34		○ Diabetes 1.78, 1.12 – 2.83
Al-Samkari et al., 2020 (USA)	Adults	400	R		○ Male 0.61, 0.41 – 0.93<br>○ Hispanic vs non-hispanic 1.01, 0.65 – 1.59	

					o Cardiovascular disease 0.90, 0.58 – 1.41<br>o Chronic pulmonary disease 0.80, 0.47 – 1.36<br>o Diabetes 1.98, 1.28 – 3.06<br>o Chronic kidney disease 2.74, 0.76 – 9.87<br>o Chronic liver disease 0.81, 0.30 – 2.18	
Alser et al., 2021 (USA)	Median 60 IQR 47-70	235	P	o Age≥60 vs <60 = 4.18, 2.13 – 8.17<br>o Male 2.49, 0.89 – 7.00<br>o Hispanic vs non-hispanic 0.41, 0.21 – 0.79<br>o Hypertension 2.25, 1.22 – 4.17<br>o Chronic pulmonary disease 5.06, 1.83 – 14.00<br>o Smoker 2.76, 1.50 – 5.27<br>o Stroke 5.94, 1.67 – 21.09<br>o Chronic liver disease 2.55, 0.66 – 9.83<br>o Chronic kidney disease 2.17, 0.66 – 9.83		
Alsharidah etal., 2021 (Kuwait)	Median 54 Range 15-82	368	P		o Male 1.12, 0.63 – 1.97<br>o Diabetes 0.95, 0.62 – 1.48<br>o Hypertension 1.34, 0.86 – 2.09<br>o Obesity 1.28, 0.63-2.61	
Altschul et al., 2020 (USA)	Mean 63.3	4711	P	o Age ≥ 60 vs <60 = 2.4, 1.18 – 5.16<br>o Male 1.05, 0.63 – 1.72<br>o Daibetes 0.95, 0.78 – 1.16		

				○ Chronic respiratory disease 1.46, 1.06 – 2.02		
Alzaid et., 2020 (USA)	Not sepcified	45	P			○ Diabetes 1.37, 0.35 – 5.43
Del Amo et al., 2020 (Spain)	Range 20-79	77590	P	○ Male 0.59, 0.18 – 1.91<br>○ Age ≥60 vs <60 = 4.64, 1.81 – 11.89		○ Male 0.60, 0.16 - 2.27<br>○ Age ≥60 vs <60 = 2.28, 0.77 – 6.71
Anaklı etal., 2021 (Turkey)	Mean 67 SD 16	104	R	○ Male 0.95, 0.40-2.27<br>○ Cardiovascular disease 1.84, 0.81 – 4.18<br>○ Hypertension 1.77, 0.84 – 3.73<br>○ Diabetes 1.79, 0.76 – 4.22<br>○ Chronic respiratory disease 1.40, 0.54 – 3.66<br>○ Cancer 2.64, 0.80 – 8.69<br>○ Chronic renal disease 1.54, 0.37 – 6.34<br>○ Chronic liver disease 0.61, 0.04 – 10.09<br>○ Cerebrovascular disease 1.33, 0.31 – 5.65		
Anantharamna et al., 2020 (USA)	≥18	4627	R	○ Male 1.72, 1.17 – 2.56<br>○ Hypertension 1.71, 1.13 – 2.60<br>○ Diabetes 1.27, 0.81 – 2.00<br>○ Smoking 1.15, 0.76 – 1.73		○ Male 2.08, 1.58 – 2.70<br>○ Hypertension 2.74, 1.99 – 3.76<br>○ Diabetes 1.41, 1.00 – 1.99<br>○ Smoking 1.10, 0.83 – 1.46
Andargie et al., 2021 (USA)	Median 48.5 IQR 37-56	85	P	○		○ Male 3.73, 1.21 – 11.50<br>○ Hypertension 0.64, 0.22 – 1.83<br>○ Diabetes 0.71, 0.23 – 2.00<br>○ Cardiovascular disease 0.57, 0.16 – 2.08

						○ Obesity 2.46, 0.77 – 7.86
Angelidi et al., 2021 (USA)	Median 66	144	R	○ Age continuous, 1.81, 1.05-3.13<br>○ Male 2.00, 0.47 – 8.63<br>○ Smoking 1.26, 0.23 – 7.02<br>○ Cardiovascular disease 2.47, 0.43 – 14.39<br>○ Chronic respiratory disease 6.69, 1.10 – 40.89<br>○ Diabetes 0.49, 0.11 – 2.09<br>○ Hypertension 0.84, 0.15 – 0.84		
Angelis et al., 2020 (UK)				○ Age (continuous) 1.06, 1.02–1.09<br>○ Male 2.12, 0.86 – 5.18		
Antúnez-Montes et al., 2021 (Multinational)	Median 3 years IQR 0.6-9	409	P			○ Male 1.17, 0.65 – 2.11
Aoun et al., 2021 (Labanon)	Mean 61.46 SD 13.99	231	R	○ Male 1.16, 0.63 – 2.14<br>○ Smoking 1.12, 0.56 – 2.27<br>○ Hypertension 1.65, 0.60 – 4.55<br>○ Diabetes 1.34, 0.71 – 2.54<br>○ Obesity 0.88, 0.41 – 1.88<br>○ Cardiovascular disease 4.42, 2.06 – 9.49<br>○ Stroke 2.17, 0.89 – 8.28		

				○ Chronic respiratory disease 2.03, 0.85 – 4.86<br>○ Cancer 1.81, 0.68 – 4.76		
Apea et al., 2021 (UK)	≥16	1737	P	○ Age (HR) 4.50, 3.74 – 5.42<br>○ Male (HR) 1.55, 1.28 – 1.87		
Atkins et al., 2020 (UK)	MEAN 74.3 SD 4.5	507	P	○ Male 2.12, 1.45 – 3.09<br>○ Stroke 0.93, 0.40 – 2.17<br>○ Hypertension 1.42, 0.96 – 2.11<br>○ Diabetes 3.11, 2.06 – 4.71<br>○ Chronic kidney disease 0.88, 0.35 – 2.24<br>○ Asthma 0.59, 0.33 – 1.04<br>○ Chronic respiratory disease 1.91, 1.10 – 3.32		
Attauabi et al., 2021 (Denmark)	Median 55 IQR 41-74	328	P	○ Age>65 vs ≤65 = 4.82, 1.22 – 24.22<br>○ Smoker 1.31, 0.49 – 4.86<br>○ Hypertension 0.75, 0.22 – 2.37<br>○ Diabetes 1.50, 0.39 – 5.29<br>○ Cardiovascular disease 4.28, 1.15 – 16.50<br>○ Chronic renal disease 3.63, 0.76 – 13.36<br>○ Chronic respiratory disease 3.96, 0.85 – 18.71<br>○ Stroke 1.31, 0.20 – 4.99		

				o Cancer 3.18, 0.80 – 12.78		
Auld et al., 2020 (USA)	Median 64 IQR 54-73	217	P	o Age ≥65 vs <65 = 4.06, 2.12 – 7.77<br>o Male 0.77, 0.42 – 1.39<br>o Hypertension 1.15, 0.62 – 2.13<br>o Cardiovascular disease 1.13, 0.54 – 2.35<br>o Diabetes 1.08, 0.59 – 1.95<br>o Chronic kidney disease 2.68, 1.39 – 5.17<br>o Asthma 0.65, 0.20 – 2.07<br>o Chronic pulmonary disease 0.83, 0.28 – 2.42		
Avruscio et al., 2020 (Itlay)	≤18	85	CS	o		o Male 2.36, 0.88 – 6.32<br>o Hypertension 0.86, 0.36-2.01<br>o Diabetes 0.67, 0.25 – 1.79<br>o Chronic respiratory disease 1.39, 0.35 – 5.57<br>Cancer 1.08, 0.28 – 4.05
Aw et al., 2020 (UK)	≥65	677	P	o Male 2.02, 1.47 – 2.77		
Awad et al., 2021 (USA)	Median 64.3	336	R	o		o Age 1.00, 0.98 – 1.02<br>o Male 1.17, 0.66 – 2.10<br>o Obesity 1.26, 0.69 – 2.31<br>o Diabetes 1.09, 0.59 – 2.03
Azar et al., 2020 (USA)	Mean 50.7	14,036	R	o Age ≥60 vs <60 = 14.17, 5.98 – 33.55<br>o Male 1.95, 1.08 – 3.51<br>o Cardiovascular disease 7.95, 4.35 – 14.51<br>o Diabetes 2.41, 1.26 – 4.56		o Age ≥60 vs <60 = 3.69, 2.43 – 5.59<br>o Male 1.93, 1.28 – 2.90<br>o Cardiovascular disease 3.53, 2.14 – 5.81<br>o Diabetes 3.53, 2.26 – 5.51<br>o Cancer 2.27, 1.13 – 4.52<br>o Hypertension 3.05, 2.04 – 4.56

				○ Cancer 5.18, 2.44 – 11.00<br>○ Hypertension 3.26, 1.83 – 5.78<br>○ Chronic respiratory disease 7.44, 3.30 – 16.77<br>○ Asthma 1.99, 0.97 – 4.09		○ Chronic respiratory disease 5.12, 2.53 – 10.39<br>○ Asthma 1.89, 1.12 – 3.21
Azwar et al., 2020 (Indonesia)	≥60	44	CS	○ Male 6.3, 0.71 – 55.50		
Baggio et al., 2021 (Brazil)	41	59,695	CS	○ Male 1.64, 1.47 – 1.82<br>○ Age ≥60 vs <60 = 11.87, 10.5 – 13.36<br>○ Cardiovascular 1.26, 1.07 – 1.49<br>○ Diabetes 2.82, 2.46 – 3.24<br>○ Chronic respiratory disease 1.55, 1.13 – 2.14<br>○ Hypertension 1.75, 1.43 – 2.14<br>○ Obesity 3.22, 1.87 – 5.54<br>○ Chronic kidney disease 2.55, 1.73 – 3.75		
Bailey et al., 2021 (USA)	Mean 8.8 Sd 6.7	135,794	P		○ Male 0.54, 0.45 – 0.65<br>○ Obesity 1.39, 1.13 – 1.72	
Bannaga et al., 2020 (UK)	Median 73	321	R	○ Age >65 vs ≤65 = 5.35, 2.94 – 9.70<br>○ Male 1.04, 0.65 – 1.68		○ Age>65 vs ≤65 = 0.26, 0.13 – 0.51<br>○ Male 2.33, 1.13 – 4.79
Baqui et al., 2020 (Brazil)	Not specified	11,321	CS	○ Male 1.12, 1.03 – 1.23<br>○ Cardiovascular disease 2.32, 2.09 – 2.55<br>○ Asthma 0.66, 0.51 – 0.84		

				○ Diabetes 2.54, 2.28 – 2.83<br>○ Chronic respiratory disease 2.52, 2.02 – 3.12<br>○ Obesity 1.57, 1.25 – 1.96<br>○ Chronic renal disease 3.74, 2.97 – 4.73<br>○ Chronic liver disease 2.28, 1.45 – 3.59		
Barbui et al., 2021 (Multinational)	Adults	175	R	○ Age ≥60 vs <60 = 11.05, 2.55 – 47.81<br>○ Male 2.48, 1.01 – 6.07<br>○ Cardiovascular disease 2.18, 0.64 – 7.36<br>○ Cerebrovascular disease 0.86, 0.32 – 2.34<br>○ Chronic kidney disease 3.22, 1.22 – 8.49<br>○ Chronic respiratory disease 1.49, 0.61 – 3.64<br>○ Hypertension 1.67, 0.83 – 3.33<br>○ Diabetes 2.65, 1.08 – 6.50		
Barron et al., 2020 (UK)	Adults	614,144, 70	CS	○ Diabetes 9.15, 8.91 – 9.41		
Bartoletti et al., 2020 (Italy)	Mean 74 SD 12	513	P	○ Age (continuous) 1.06, 1.03 – 1.09)<br>○ Male 1.43, 0.72 – 2.83<br>○ Obesity 1.40, 0.88 – 2.23<br>○ Hypertension 1.66, 0.93 – 2.96		

				○ Diabetes 1.20, 0.72 – 2.83<br>○ Cerebrovascular disease 2.78, 1.73 – 4.45<br>○ Chronic kidney disease 0.75, 0.28 – 2.01<br>○ Chronic respiratory disease 1.88, 1.16 – 3.90		
Bauer et al., 2020 (USA)	Range 18-100	1449	P		○ Male 1.29, 0.99 – 1.69<br>○ Smoking 1.80, 1.35 – 2.41<br>○ Hypertension 1.33, 0.95 – 1.84<br>○ Diabetes 1.47, 1.06 – 2.04<br>○ Chronic respiratory disease 1.42, 1.03 – 1.97<br>○ Cardiovascular disease 1.72. 1.09 – 2.70<br>○ Chronic kidney disease 1.80, 1.24 – 2.61<br>○ Cancer 1.72, 1.11 – 2.67<br>○ Chronic liver disease 2.14, 0.76 – 6.07	
Becerra-Munoz et al., 2021 (Spain)	Median 76 IQR 71-83	1520	P	○ Age ≥75 vs <75 = 3.54, 1.76 – 8.38<br>○ Chronic kidney disease 3.36, 1.00 – 11.33		
Bedock et al., 2020 (France)	Mean 59.9 SD 15.9	114	CS	○ Malnutrition 1.90, 0.2 – 12.3		○ Malnutrition 0.58, 0.1 – 1.9
Belli et al., 2021 (Multinational)	Adults	243	CS	○ Male (HR) 1.39, 0.71 – 2.73<br>○ Diabetes (HR) 1,98, 1.11 – 3.54<br>○ Hypertension (HR) 1.76, 0.98 – 3.17		○ Age >60 vs ≤60 = 1.40, 0.67 – 2.91<br>○ Male 0.99, 0.46 – 2.14<br>○ Alcohol 0.81, 0.35 – 1.89<br>○ Diabetes 1.84, 0.91 – 3.72<br>○ Hypertension 5.48, 2.38 – 12.58<br>○ Chronic lung disease 0.45, 0.10 – 2.01

				○ Chronic lung disease (HR) 0.55, 0.17 – 1.76<br>○ Chronic kidney disease (HR) 1.72, 0.92 – 3.22<br>○ Smoking (HR) 1.62, 0.72 – 3.63		○ Chronic kidney disease 1.11, 0.47 – 2.61<br>○ Smoking 1.90, 0.71 – 5.14
Otero et al., 2021 (Spain)	Median 82 IQR 75-88	83	P	○ Male 0.97, 0.37 – 2.54<br>○ Diabetes 1.12, 0.42 – 2.93<br>○ Hypertension 4.82, 1.02 – 22.75<br>○ Obesity 0.82, 0.27 – 2.43<br>○ Chronic lung disease 0.91, 0.22 – 3.77<br>○ Cerebrovascular disease 4.91, 1.07 – 22.52<br>○ Cancer 0.24, 0.02 – 2.02<br>○ Smoking 0.53, 0.16 – 1.81		
Alguwaihes et al., 2021 (Saudi Arabia)	Mean 56.6 SD 16.2	222	R	○ Age >60 vs ≤60 = 1.4, 0.63 – 3.1<br>○ Male 3.3, 1.2 – 9.2<br>○ Smoking 1.7, 0.2 – 20.0<br>○ Diabetes 1.7, 0.7 – 4.0<br>○ Hypertension 1.0, 0.4 – 2.6<br>○ Cerebrovascular disease 2.3, 0.8 – 6.1<br>○ Cardiovascular disease 1.4, 0.4 – 4.3<br>○ Chronic kidney disease 3.5, 1.4 – 8.7<br>○ Stroke 2.0, 0.5 – 7.1		
Allameh et al., 2020 (Iran)	Mean 56.9 SD 15.7	396	CS	○ Male 1.39, 0.82 – 2.35<br>○ Age >60 vs ≤60 = 3.72, 2.14 – 6.43		

				○ Diabetes 0.91, 0.54 – 1.55<br>○ Hypertension 1.26, 0.77 – 2.06<br>○ Cardiovascular disease 1.51, 0.88 – 2.62		
Amanat et al., 2021 (Iran)	Mean 60.71 SD 18.14	873	P	○ Age >60 vs ≤60 = 8.79, 4.94 – 15.62<br>○ Male 1.36, 0.90 – 2.06<br>○ Smoking 2.69, 1.73 – 4.19	○ Age >60 vs ≤60 = 1.63, 1.23 – 2.16<br>○ Male 1.38, 1.03 – 1.86<br>○ Smoking 1.62, 1.12 – 2.35	
Annweiler et al., 2020 (France)	Mean 88 SD 5	77	P		○ Age (continuous) 1.05, 0.88 – 1.25<br>○ Cancer 7.30, 1.37 – 38.8<br>○ Hypertension 0.51, 0.11 – 2.33	
Antúnez-Montes et al., 2021	Median 3 IQR 0.6-9	409	P			○ Male 1.17, 0.65 – 2.11
Berenguer et al., 2020 (Spain)	Median 70	4035	R	○ Male 1.57, 1.35 – 1.82<br>○ Age>60 vs ≤60 = 7.53, 6.03 – 9.41<br>○ Smoking 1.84, 1.56 – 2.17<br>○ Hypertension 3.23, 2.78 – 3.74<br>○ Cardiovascular disease 3.21, 2.75 – 3.75<br>○ Diabetes 2.16, 1.84 – 2.53<br>○ Chronic lung disease 2.35, 1.98 – 2.77<br>○ Obesity 1.63, 1.33 – 1.99<br>○ Asthma 0.76, 0.57 – 1.00<br>○ Chronic kidney disease 3.68, 2.76 – 4.92		

				o		
Bernard et al., 2021 (France)	All ages	89530	R	o Cancer 2.7, 2.5 – 2.8		
Bertsimas et al., 2020 (USA)	Median 68<br>IQR 57-79	3062	CS	o Male 1.46, 1.23 – 1.73<br>o Chronic kidney disease 2.48, 1.55 – 3.98<br>o Cardiovascular disease 1.74, 1.20 – 2.54<br>o Diabetes 1.47, 1.16 – 1.85		
Betti et al., 2021	MEAN 53.6<br>SD 9.7	171	R		o Age ≥50 vs <50 = 3.23, 1.42 – 7.37<br>o Cardiovascular disease 0.38, 0.09 – 1.50<br>o Cerebrovascular disease 1.65, 0.27 – 10.14<br>o Diabetes 0.71, 0.19 – 2.61	
Bhargava et al., 2020 (USA)	Mean 60.6	197	R		o Age >60 vs ≤60 = 1.84, 1.02 – 3.31<br>o Male 0.57, 0.32 – 1.03<br>o Cardiovascular disease 1.68, 0.76 – 3.71<br>o Cerebrovascular disease 1.53, 0.56 – 4.17<br>o Diabetes 2.91, 1.62 – 5.25<br>o Chronic renal dsease 3.03, 1.38 – 6.63<br>o Chronic respiratory disease 2.16, 1.05 – 4.42<br>o Cancer 1.11, 0.30 – 4.08<br>o Hypertension 1.55, 0.81-2.98<br>o Obesity 1.45, 0.78-2.72	
Bhatt et al., 2021 (USA)	Not specified	8383	CS	o Age. Reference=<60<br>60-69= 1.65, 1.36 – 2.00<br>70-79=2.26, 1.88 - 2.72<br>>80 = 2.86, 2.38 – 3.45<br>o Male 1.26, 1.13 – 1.40		

				○ Diabetes 1.13, 1.01 – 1.26<br>○ Hypetension 0.99, 0.86 – 1.14<br>○ Chonic kidney disease 1.45, 1.29 – 1.62<br>○ Chronic lung disease 1.20, 1.07 – 1.33<br>○ Smoking 0.96, 0.86 – 1.07<br>○ Cancer 1.33, 1.02 – 1.74		
Bianchetti et al., 2020 (Italy)	Mean 70.7 SD 12.9	627	R	○ Age (continuous) = 1.09, 1.07 – 1.12<br>○ Male 1.15, 0.78 – 1.66		
Bielza et al., 2021 (Spain)	≥70	630	R	○ Age (continuous)=1.01, 0.98 – 1.03<br>○ Male 1.56, 1.07 – 2.28<br>○ Hypertension 1.10, 0.79 – 1.53<br>○ Chronic lung disease 0.66, 0.39 – 1.12<br>○ Diabetes 1.18, 0.78 – 1.78<br>○ Chronic renal disease 1.39, 0.84 – 2.32<br>○ Obesity 1.69, 0.85 – 3.35<br>○ Smoking 0.90, 0.31 – 2.63		
Bode et al., 2020 (USA)		570	R	○ Diabetes 6.10, 3.62 – 20.28		
Boero et al., 2020 (Italy)	Mean 67.7 SD 14.4	274	R		○ Male 1.25, 0.73 – 2.14<br>○ Cancer 1.45, 0.58 – 3.66	
Bolouri et al., 2021 (USA)		59	P		○ Male 1.33, 0.94 – 2.67<br>○ Diabetes 2.11, 0.71 – 6.27	

					○ Hypertension 0.84, 0.29 – 2.38	
Bossini et al., 2020 (Italy)	Median 60 IQR 50-67	53	P	○ Age>60 vs ≤60 = 1.12, 1.03 – 1.24<br>○ Hypertension 0.38, 0.08 – 1.3<br>○ Diabetes 1.61, 0.36 – 6.48<br>○ Cardiovascular disease 1.94, 0.43 – 8.15		
Botta et al., 2021 (Netherlands)	Median 67 IQR 59-73	533	R	○ Age (continuous) = 2.19, 1.65 – 2.90<br>○ Male 2.16, 1.24 – 3.78<br>○ Hypertension 1.16, 0.72 – 1.88<br>○ Diabetes 1.58, 0.93 – 2.67<br>○ Chronic kidney disease 0.89, 0.30 – 2.61<br>○ Chronic lung disease 1.70, 0.86 – 3.36		
Bottio et al., 2021 (Italy)	Mean 61.8 SD 14.5	47	R	○ Male 1.92, 0.43 – 6.09<br>○ Obesity 1.52, 0.31 – 7.51<br>○ Hypertension 0.67, 0.18 – 2.40<br>○ Diabetes 5.56, 1.11 – 27.89<br>○ Smoking 0.85, 0.19 – 3.84<br>○ Cancer 1.67, 0.25 – 11.26		
Brandão Neto RA et al., 2021 (Brazil)	Mean =59.2 years (±16.8)	506	P	○ Male 0.90, 0.61 – 1.32<br>○ Hypertension 1.13, 0.77 – 1.66<br>○ Diabetes 1.34, 0.91 – 1.98<br>○ Smoking 1.02, 0.67 – 1.55		○ Male 1.14, 0.81 – 1.64<br>○ Hypertension 1.06, 0.75 – 1.52<br>○ Diabetes 1.57, 1.08 – 2.28<br>○ Smoking 0.87, 0.59 – 1.29<br>○ Cardiovascular disease 1.11, 0.56 – 2.16

				○ Cardiovascular disease 1.32, 0.67 – 2.62<br>○ Chronic lung disease 5.39, 1.84 – 15.79<br>○ Asthma 1.69, 0.73 – 3.90<br>○ Cancer 2.96, 1.53 – 5.73		○ Chronic lung disease 2.82, 0.78 – 10.12<br>○ Asthma 0.67, 0.28 – 1.58<br>○ Cancer 0.63, 0.33 – 1.21
Bravi et al., 2020 (Italy)	Mean 58	1603	P		○ Male 1.76, 1.40 – 2.23<br>○ Diabetes 1.52, 1.05 – 2.18<br>○ Hypertension 1.10, 0.8 – 1.47<br>○ Cardiovascular disease 1.88, 1.32 – 2.70<br>○ Cancer 1.26, 0.81 – 1.95<br>○ Chronic lung disease 1.88, 1.11 – 3.20<br>○ Chronic renal disease 1.58, 0.90 – 2.76	
Brenner et al., 2020 (USA)	Medin 41	525	R	○ Age (continuous) = 1.07, 1.03 – 1.11<br>○ Male 2.78, 0.76 – 10.14<br>○ Smoking 1.47, 0.12 – 17.53		
Brouns et al., 2020 (Netherlands)	Mean 85<br>SD 8.1	101	R	○ Age (continuous) = 1.04, 0.39 – 2.77<br>○ Male 4.79, 1.74 – 13.18<br>○ Hypertension 0.54, 0.22- 1.37		
Bruggemann et al., 2020 (Netherlands)	Mean 68<br>SD 11.7	24	R			○ Male 2.83, 0.56 – 14.38
Byeon et al., 2021 (Korea)		12,464	R	○ Male 1.53, 1.22 – 1.92		
Cabello et al., 2021 (Spain)	Median 46<br>IQR 6.5-16.8	63	R		○ Hypertension 5.09, 1.34 – 19.02<br>○ Diabetes 6.14, 1.01 – 37.21<br>○ Obesity 5.38, 1.13 – 25.64<br>○ Smoking 8.23, 2.07 – 32.68	

Cabezudo-García et al., 2020 (spain)		1537	CS	o Age (continuous) =1.07, 1.04 – 1.09)<br>o Male 1.39, 0.79 – 2.44<br>o Hypertension 2.96, 1.56 – 5.62<br>o Diabetes 1.54, 0.85 – 2.81<br>o Cardiovascular disease 2.48, 1.38 – 4.46<br>o Chronic lung disease 1.58, 0.85 – 2.95		
Cai et al., 2021 (China)	Median 63 IQR 51-70	2424	R	o Cancer 1.93, 0.49 – 7.49		
Cai et al., 2020 (China)		383	P		o Male 2.34, 1.44 – 3.81<br>o Diabetes 4.28, 1.78 – 10.28<br>o Hypertension 2.28, 1.25 – 4.14<br>o Cardiovascular disease 3.49, 1.71 – 7.12<br>o Liver disease1.07, 0.38 – 3.04<br>o Chronic lung disease 2.39, 1.13 – 5.06	
Cai et al., 2020 (China)		96	R			o Male 1.36, 0.59 – 3.11<br>o Age (continuous) = 1.04, 1.01 – 1.07<br>o Smoking 1.04, 0.23 – 4.63
Cai et al., 2020 (China)		941	R	o Male (HR) 0.90, 0.50 – 1.63<br>o Hypertension (HR) 1.89, 1.04 – 3.45<br>o Diabetes (HR) 0.67, 0.32 – 1.39<br>o Cardiovascular disease (HR) 0.42, 0.18 – 0.96		

				○ Cerebrovascular disease (HR) 0.75, 0.30 – 1.87<br>○ Chronic renal disease (HR) 3.17, 1.48 – 6.80<br>○ Chronic lung disease (HR) 0.40, 0.15 – 1.06		
Caillard et al., 2020 (France)	Median 62 IQR 27.8 – 140.6	306	R	○ Age>60 vs ≤60 = 3.93, 2.27 – 6.79<br>○ Male 0.96, 0.60 – 1.53<br>○ Hypertension 1.66, 0.99 – 2.79<br>○ Cardiovascular disease 2.38, 1.51 – 3.76<br>○ Chronic respiratory disease 0.90, 0.47 – 1.71<br>○ Diabetes 1.86, 1.18 – 2.93<br>○ Cancer 0.85, 0.41 – 1.81<br>○ Smoking 1.25, 0.56 – 2.82		
Çalık Başaran N et al., 2021a (Turkey)	Median 45.5 IQR 19-92	174	P		○ Age ≥65 vs <65 = 4.15, 1.64 – 10.52<br>○ Male 2.32, 1.02 – 5.26<br>○ Hypertension 2.47, 1.03 – 5.94<br>○ Diabetes 3.57, 1.43 – 8.89<br>○ Smoking 1.52, 0.69 – 3.35<br>○ Alcohol 3.31, 1.29 – 8.46	
Caliskan et al., 2020 (Turkey)	Adults	565	R	○ Smoking 13.01, 5.05 – 33.48<br>○ Age (continuous) 1.08, 1.05 – 1.11<br>○ Chronic lung disease 3.21, 1.22 – 8.43<br>○ Diabetes 1.04, 0.44 – 2.42		○ Smoking 5.10, 2.38 – 10.92<br>○ Age 1.06, 1.04 – 1.08<br>○ Chronic lung disease 2.77, 1.12 – 6.82<br>○ Diabetes 1.15, 0.55 – 2.39<br>○ Hypertension 1.57, 0.82 – 3.01

				○ Hypertension 1.45, 0.69 – 3.07<br>○ Chronic renal disease 3.98, 0.93 – 16.89		○ Chronic renal disease 3.68, 0.98 – 13.81
Cao J et al., 2020 (China)	Adult Median 54	102	R	○ Male 3.65, 1.10 – 12.12<br>○ Hypertension 7.33, 2.37 – 22.65<br>○ Diabetes 8.72, 2.27 – 33.45<br>○ Cerebrovascular disease 5.85, 1.07 – 31.98<br>○ Cardiovascular disease 8.89, 1.36 – 58.08<br>○ Chronic respiratory disease 4.05, 1.00 – 16.33		
Capdevila-Reniu et al., 2021 (Spain)	≥80	159	R	○ Male 1.52, 0.78 – 2.94<br>○ Diabetes 1.25, 0.61 – 2.67<br>○ Chronic lung disease 0.58, 0.18 – 1.88<br>○ Cerebrovascular disease 1.31, 0.48 – 3.61<br>○ Cancer 2.01, 0.79 – 5.08<br>○ Chronic kidney disease 1.40, 0.67 – 2.93<br>○ Hypertension 0.86, 0.40 – 1.83		
Cariou et al., 2020 (France)	Mean 69.8 SD 13	1317	P	○ Male 1.25, 0.85 – 1.81<br>○ Hypertension 1.82, 1.11 – 2.8<br>○ Smoking 1.20, 0.49 – 2.93		
Carubbi et al., 2021 (Italy)	Median 65	61	R	○ Male 0.83, 0.12 – 5.41		
Catteau et al., 2020 (Belgium)		8910	P	○ Age ≥65 vs <65 = 7.74, 6.63 – 9.04		

				○ Male 1.15, 1.04 – 1.28<br>○ Cardiovascular disease 2.46, 2.23 – 2.73<br>○ Diabetes 1.50, 1.34 – 1.68<br>○ Chronic renal disease 2.46, 2.16 – 2.81<br>○ Chronic liver disease 1.77, 1.34 – 2.33<br>○ Chronic lung disease 1.49, 1.31 – 1.70<br>○ Cancer 1.08, 0.80 – 1.46		
Cattelan et al., 2020 (Italy)	Median 62	303	R			○ Male 2.69, 1.45 – 4.99<br>○ Hypertension 3.34, 1.86 – 6.02<br>○ Diabetes 1.76, 0.97 – 3.20<br>○ Cardiovascular disease 0.72, 0.32 – 1.63<br>○ Chronic lung disease 1.27, 0.51 – 3.17<br>○ Cancer 0.79, 0.28 – 2.18<br>○ Chronic kidney disease 0.84, 0.23 – 3.06<br>○ Obesity 0.92, 0.44 – 1.91
Cekerevac et al., 2021 (Serbia)		127	CS		○ Male 1.16, 0.55 – 2.47<br>○ Smoking 0.52, 0.18 – 1.47<br>○ Hypertension 3.78, 1.81 – 7.91<br>○ Diabetes 1.39, 0.61 – 3.19<br>○ Obesity 1.10, 0.43 – 2.84<br>○ Chronic lung disease 0.81, 0.15 – 4.15<br>○ Cancer 0.81, 0.11 – 5.92	
Cen Y et al., 2020 (China)	Median 69 IQR 49-68	1007	p	○ Age ≥65 vs <65 = 6.62, 3.14 – 13.97<br>○ Male 2.81, 1.42 – 5.53<br>○ Hypertension 2.48, 1.34 – 4.59	○ Age ≥65 vs <65 = 5.00, 3.66 – 6.83<br>○ Male 1.69, 1.26 – 2.28<br>○ Hypertension 2.90, 2.12 – 3.96<br>○ Diabetes 3.80, 2.51 – 5.74	

				o Diabetes 3.10, 1.54 – 6.21<br>o Chronic lung disease 2.25, 0.76 – 6.59<br>o Cardiovascular disease 5.01, 2.35 – 10.68<br>o Chronic renal disease 3.86, 0.83 – 17.85<br>o Cerebrovascular disease 3.21, 0.92 – 11.17<br>o Smoking 0.24, 0.03 – 1.76	o Chronic lung disease 6.37, 3.29 – 12.31<br>o Cardiovascular disease 2.36, 1.35 – 4.11	
Chand et al., 2020 (USA)	Adults	300	R	o Male 1.83, 1.14 – 2.92<br>o Age ≥60 vs <60 = 2.15, 1.35 – 3.42<br>o Smoking 2.03, 1.15 – 3.57<br>o Alcohol 0.97, 0.45 – 2.09<br>o Diabetes 1.16, 0.74 – 1.84<br>o Hypertension 1.86, 1.15 – 3.03<br>o Asthma 0.67, 0.34 – 1.32<br>o Cardiovascular disease 1.72, 0.62 – 4.78<br>o Chronic kidney disease 1.21, 0.61 – 2.37<br>o Cancer 2.49, 0.86 – 7.17		
Chang et al., 2020 (Korea)	Adults	106	R	o Male 2.02, 0.84 – 4.82<br>o Hypertension 1.89, 0.78 – 4.58<br>o Diabetes 2.66, 1.12 – 6.34<br>o Chronic kidney disease 0.40, 0.11 – 1.53		

				o Cancer 2.18, 0.54 – 8.76		
Chaudhry et al., 2020 (USA)	Adults	40	R		o Male 1.61, 0.28 – 9.23<br>o Smoking 1.16, 0.11 – 12.13<br>o Hypertension 1.61, 0.28 – 9.23	
Chen C et al., 2021 (China)	Median 68	2297	R	o Hypertension 1.98, 1.43 – 2.75		
Chen J et al., 2020a (China)	Medain 62 IQR 49-69	3309	R	o Age >45 vs ≤45 = 9.08, 4.44-18.59<br>o Male 2.27, 1.72 – 2.94<br>o Hypertension 1.14, 0.87 – 1.50<br>o Diabetes 0.97, 0.70 – 1.36<br>o Cardiovascular disease 1.41, 0.94 – 2.13<br>o Cerebrovascular disease 1.25, 0.73 – 2.13<br>o Cancer 3.17, 1.84 – 5.46<br>o Chronic renal disease 2.85, 1.42 – 5.73<br>o Chronic lung disease 1.72, 0.80 – 3.71	o Male 1.14, 0.97 – 1.34	
Chen J et al., 2020b (China)	Median 51 IQR 36-64	249	R			o Age (continuous) = 1.06, 1.00 – 1.12<br>o Male 3.38, 0.77 – 14.9
Chen L et al., 2020 (China)	Median 55 IQR 36-68	792	R	o Male 0.81, 0.49 – 1.34<br>o Hypertension 3.00, 1.81 – 4.96<br>o Diabetes 3.24, 1.91 – 5.50<br>o Chronic lung disease 4.02, 1.40 – 11.53		

				○ Chronic kidney disease 2.65, 0.96 – 7.28		
Rajter et al., 2021 (US)	280		R	○ Male 2.38, 0.59 – 4.16<br>○ Smoking 3.94, 0.71 – 17.32<br>○ Diabetes 1.17, 0.39 – 3.55<br>○ Hypertension 0.72, 0.17 – 3.08		
					○	
Chen Q et al., 2020b (China)	Mean 47.5	145	R		○ Male 0.94, 0.46 – 1.93<br>○ Hypertension 1.81, 0.71 – 4.62<br>○ Smoking 0.56, 0.15 – 2.10	
Chen T et al., 2020 (China)	Median 54 IQR 41-68	203	R	○ Male 5.33, 1.32 – 21.52<br>○ Hypertension 1.80, 0.57 – 5.61<br>○ Diabetes 1.47, 0.39 – 5.50<br>○ Cardiovascualr disease 2.86, 0.74 – 11.06<br>○ Cerebrovascular disease 1.16, 0.24 – 5.94		
Chen Y et al., 2020 (china)	Median 56 IQR 39-67	904	R	○ Male 2.63, 1.63 – 4.16<br>○ Diabetes 2.51, 1.53 – 4.13<br>○ Hypertension 3.48, 2.24 – 5.40<br>○ Cardiovascular disease 8.29, 5.02 – 13.68<br>○ Chronic kidney disease 13.91, 7.23 – 26.80<br>○ Chronic lung disease 4.38, 1.74 – 11.03		
Chen Z et al., 2020 (China)	Median 46	836	R		○ Male 1.71, 1.21 – 2.44<br>○ Hypertension 2.41, 1.68 – 3.43<br>○ Diabetes 1.06, 0.70 – 1.59	

	IQR 36-57				o Chronic liver disease 1.51, 0.94 – 2.41<br>o Chronic renal disease 2.66, 1.08 – 6.51<br>o Cerebrovascular disease 2.91, 1.49 – 5.69<br>o Cardiovascular disease 3.15, 1.90 – 5.23	
Cheng et al., 2020 (China)	Median 49 IQR37-61	252	R		o Age >=60 vs <60 = 6.82, 3.52 – 13.21<br>o Male 1.21, 0.65 – 2.24<br>o Hypertension 1.45, 0.64 – 3.19<br>o Diabetes 1.74, 0.58 – 5.02<br>o Cardiovascular disease 1.33, 0.32 – 6.00<br>o Cerebrovascular disease 7.96, 0.75 – 173.32<br>o Liver disease 4.02, 0.73 – 22.30<br>o Chronic lung disease 1.29, 0.19 – 5.81<br>o Cancer 2.57, 0.29 – 54.99	
Chilimuri et al.,2020 (USA)	Median 63 IQR 19-97	375	R	o Male 1.37, 0.79 – 2.37<br>o Hypertension 1.46, 0.82 – 2.62<br>o Diabetes 1.58, 0.94 – 2.65<br>o Cardiovascular disease 1.56, 0.78 – 3.11<br>o Chronic lng disease 1.22, 0.71 – 2.11<br>o Chronic renal disease 1.33, 0.63 – 2.77<br>o Chronic liver disease 2.19, 0.83 – 5.79		
Choron et al., 2021 (USA)	Median 64	103	R	o Male 2.61, 1.01 – 2.26<br>o Chronic lung disease 1.12, 0.31 – 4.12		

				○ Diabetes 0.78, 0.35 – 1.72<br>○ Hypertension 1.04, 0.45 – 2.36<br>○ Chronic kidney disease 0.59, 0.17 – 1.99		
Choudhuri et al., 2020 (USA)	Mean 65.2	1044	R	○ Male 1.88, 1.37 – 2.59<br>○ Diabetes 1.38, 1.00 – 1.90<br>○ Hypertension 1.03, 0.72 – 1.47		
Ciceri et al., 2020 (Italy)	Median 65 IQR 56-75	500	R	○ Age ≥65 vs <65 = 6.08, 3.47 – 10.65<br>○ Male 1.05, 0.62 – 1.76<br>○ Hypertension 2.77, 1.71 – 4.52<br>○ Diabetes 1.71, 0.97 – 3.03<br>○ Chronic lung disease 10.43, 3.95 – 27.52<br>○ Chronic kidney disease 4.29, 2.29 – 8.04<br>○ Cancer 3.62, 1.51 – 8.63		
Cilloniz et al., 2021 (Spain)	Mean 57.7	240	R			○ Male 1.92, 1.05 – 2.01<br>○ Chronic lung disease 1.29, 0.61 – 2.71<br>○ Cardiac disease 1.03, 0.51 – 2.10<br>○ Hypertension 1.01, 0.59 – 1.69<br>○ Diabetes 1.37, 0.65 – 2.86<br>○ Chronic kidney disease 1.15, 0.52 – 2.51
Cinar et al., 2021 (Turkey)	Mean 55.3 SD 12.8	294	R	○ Male 3.94, 1.56 – 9.95<br>○ Diabetes 4.24, 1.95 – 9.22<br>○ Chronic lung disease 4.95, 2.08 – 11.80		

				○ Chronic renal failure 7.34, 2.17 – 28.84		
Cizmecioglu et al., 2020 (Turkey)	Median 45	33	R		○ Males 1.54, 0.36 – 6.44<br>○ Diabetes 1.25, 0.27 – 5.76<br>○ Hypertension 1.00, 0.22 – 4.50<br>○ Cardiovascular disease 1.90, 0.23 – 15.58<br>○ Chronic lung diseas 0.86, 0.07 – 10.65	
Collaborative GMR et al., 2021 (UK)	Median 74 IQR 54-83	5711	R			○ Male 1.22, 0.97 – 1.52<br>○ Diabetes 1.15, 0.92 – 1.43<br>○ Cardiovascular disease 1.17, 0.94 – 1.47<br>○ Chronic lung disease 1.06, 0.84 – 1.34<br>○ Cancer 1.14, 0.81-1.60
COVID-ICU group, 2020 (France, Belgium, and Switzerland)	Median 63 IQR 54-71	4643	P	○ Male 1.11, 0.95 – 1.29<br>○ Smoking 1.18, 0.85 – 1.62<br>○ Hypertension 1.49, 1.31 – 1.71<br>○ Diabetes 1.76, 1.53 – 2.03		
Covino et al., 2021 (Italy)	≥60	210	R	○ Male 1.05, 0.52 – 2.13<br>○ Hypertension 1.65, 0.81 – 3.36<br>○ Diabetes 0.66, 0.21 – 2.03<br>○ Chronic lung disease 1.98, 0.71 – 5.58<br>○ Chronic renal disease 5.96, 2.53 - 14.02<br>○ Cancer 1.00, 0.27 – 3.71		
Covino et al., 2020a (Italy)	≥80	69	R	○ Male 0.91, 0.33 – 2.49<br>○ Cardiovascular disease 1.00, 0.33 – 2.69		

				○ Hypertension 0.37, 0.13 – 1.04<br>○ Cerebrovascular disease 2.04, 0.69 – 6.01<br>○ Diabetes 0.53, 0.10 – 2.78<br>○ Chronic lung disease 3.02, 0.61 – 14.81<br>○ Cancer 1.00, 0.08 – 11.64		
Covino et al., 2020b (Italy)	Median 66<br>IQR 54-78	334	R	○ Male 1.55, 0.63 – 3.80<br>○ Hypertension 2.84, 1.23 – 6.57<br>○ Cardiovascular disease 2.49, 1.09 – 5.67<br>○ Diabetes 0.67, 0.15 – 2.96<br>○ Chronic lung disease 2.38, 0.64 – 8.77<br>○ Cancer 2.05, 0.43 – 9.72<br>○ Chronic kidney disease 4.89, 1.21 – 19.69		○ Male 1.82, 0.94 – 3.49<br>○ Hypertension 2.29, 1.27 – 4.11<br>○ Cardiovascular disease 2.91, 1.58 – 5.32<br>○ Diabetes 0.99, 0.39 – 2.51<br>○ Chronic lung disease 1.84, 0.64 – 5.36<br>○ Cancer 0.82, 0.18 – 3.77<br>○ Chronic kidney disease 1.11, 0.23 – 5.27<br>○ Obesity 5.18, 1.02 – 26.41
Covino et al., 2020c (Italy)	Median 74	166	R	○ Males 1.41, 0.52 – 3.83<br>○ Chronic lung disease 2.27, 0.50 – 10.21<br>○ Cardiovascular disease 1.35, 0.60 – 3.01<br>○ Diabetes 0.71, 0.19 – 2.60<br>○ Obesity 0.77, 0.09 – 6.73		
Cummings et al., 2020 (USA)	Median 62	1150	P	○ Male (HR) 0.85, 0.57 – 1.27<br>○ Hypertension (HR) = 1.58, 0.89 – 2.81<br>○ Cardiovascular disease (HR) 1.76, 1.08 – 2.86		

				○ Chronic lung disease (HR) 2.94, 1.48 – 5.84<br>○ Chronic kidney disease (HR) 1.50, 0.92 – 2.45<br>○ Diabetes (HR) 1.31, 0.81 – 2.10		
Dagher et al., 2021 (USA)	Mean 61.4	310	R	○ Age ≥60 vs <60 2.46, 1.35 – 6.63<br>○ Hypertension 1.36, 0.55 – 3.37<br>○ Diabetes 1.73, 0.90 – 3.32<br>○ Cardiovascular disease 1.16, 0.59 – 2.28		
Dai et al., 2020 (China)	19-88	68	R		○ Age ≥60 vs <60 = 3.87, 1.28 – 11.68	
De Smet R et al., 2020 (Belgium)	Median 85	81	R	○ Age (continuous) 1.13, 1.02 - 1.33<br>○ Male 1.07, 0.38 – 3.05		
De Smet D et al., 2020 (Belgium)	Median 68 IQR 53-79	186	R	○ Age (continuous) 1.09, 1.03 – 1.14<br>○ Male 1.02, 0.37 – 2.84<br>○ Chronic lung disease 3.61, 1.18 – 11.08<br>○ Diabates 1.16, 0.32 – 4.15		
De Souza et al., 2020 (Brazil)	Mean 70.2	5145	CS	○ Male 0.65, 0.57 – 0.74<br>○ Age ≥75 vs <75 = 2.40, 2.10 – 2.74<br>○ Diabetes 2.33, 1.99 – 2.74<br>○ Cardiovascular disease 1.15, 0.95 – 1.39<br>○ Hypertension 1.53, 1.20 – 1.94<br>○ Chronic lung disease 1.27, 0.87 – 1.86		

				○ Chronic kidney disease 2.02, 1.27 – 3.20<br>○ Obesity 1.77, 0.84 – 3.74		
De Souza et al., 2021 (India)	Median 44	689	P	○ Age ≥65 vs <65 = 4.06, 2.72 – 6.05<br>○ Male 1.79, 1.25 – 2.58<br>○ Diabetes 3.93, 2.34 – 6.61<br>○ Chronic kidney disease 1.14, 0.41 – 3.19<br>○ Hypertension 4.06, 2.41 – 6.84<br>○ Cancer 3.49, 0.99 – 12.23		
Deeb et al., 2021 (UAE)	Mean 51	1075	R	○ Obesity 1.69, 1.07 – 2.68<br>○ Diabetes 2.38, 1.57 – 3.60<br>○ Hypertension 2.42, 1.58 – 3.71<br>○ Cardioavascular disease 2.84, 1.61 – 5.01<br>○ Chronic lung disease 3.41, 1.72 – 6.78<br>○ Cancer 5.06, 1.86 – 13.79		
Derikx et al., 2020 (Netherlands)	Median 62.5	34763	R		○ Age ≥65 vs <65= 2.94, 1.06 – 8.17<br>○ Male 2.03, 0.75 – 5.51<br>○ Smoking 2.71, 0.84 – 8.83	
Di Castelnuovo et al., 2020 (Italy)	Median 67	3894	R	○ Male 1.24, 1.05 – 1.48<br>○ Age ≥65 vs <65 = 8.71, 6.84 – 11.09<br>○ Hypertension 2.38, 2.00 – 2.84		

				○ Diabetes 2.06, 1.71 – 2.49<br>○ Cancer 2.46, 1.96 – 3.10<br>○ Chronic lung disease 2.30, 1.87 – 2.82<br>○ Obesity 0.96, 0.73 – 1.28<br>○ Smoking 0.93, 0.68 – 1.25		
Di Cosimo et al., 2021 (Italy)	Median 61	231	P/R	○ Age ≥60 vs <60 = 2.92, 1.14 – 7.50<br>○ Male 1.67, 0.97 – 2.91<br>○ Smoking 1.68, 0.74 – 3.7		
Du H et al., 2020 (China)	Mean 61.8	164	R	○ Male 2.46, 1.04 – 5.80<br>○ Smoking 0.59, 0.13 – 2.74<br>○ Hypertension 2.00, 0.88 – 4.54<br>○ Diabetes 1.47, 0.56 – 3.83<br>○ Obesity 2.13, 0.94 – 4.83<br>○ Chronic lung disease 2.54, 0.71 – 9.08<br>○ Renal disease 4.21, 1.65 – 10.72<br>○ Cancer 1.44, 0.37 – 5.61		
Du RH et al., 2020 (China)	Mean 70.7	109	R			○ Male 1.26, 0.56 – 2.84<br>○ Age ≥65 vs <65 = 0.84, 0.35 – 2.04<br>○ Hypertension 0.81, 0.37 – 1.73<br>○ Diabetes 1.43, 0.63 – 3.23<br>○ Chronic lung disease 0.29, 0.08 – 0.97

						○ Cancer 0.35, 0.07 – 1.84
Duan et al., 2020 (China)		348	R		○ Male 2.16, 0.81 – 5.78<br>○ Hypertension 3.31, 1.02 – 10.73<br>○ Diabetes 3.93, 0.79 – 19.58	
Duarte et al., 2020 (Brazil)	Adults	43271	R	○ Age ≥65 vs <65 = 1.3, 0.93 – 1.82<br>○ Male 0.84, 0.61 – 1.15<br>○ Cardiovascular disease 1.39, 0.93 – 2.07<br>○ Diabetes 1.14, 0.73 – 1.77<br>○ Chronic lung disease 1.03, 0.47 – 2.25	○	
Duclos et al, 2021 (France)	Mean 62	57	R	○ Age (continuous) 1.23, 1.06 – 1.43<br>○ Male 1.53, 0.12 – 18.8<br>○ Hypertension 2.5, 0.66 – 9.51<br>○ Diabetes 0.45, 0.11 – 1.91<br>○ Smoking 2.71, 0.64 – 11.52<br>○ Obesity 1.58, 0.43 – 5.86		
Nguyen et al., 2020 (USA)	Mean 52.7	442	R		○ Age (continuous) 1.49, 1.30 – 1.70<br>○ Male 2.01, 1.34 – 3.04<br>○ Obesity 1.95, 1.11 – 3.42<br>○ Hypertension 1.19, 0.71 – 1.99<br>○ Diabetes 1.77, 1.03 – 3.03	
Elamari et al., 2020 (Morocco)	Median 53 IQR 36-64	133	R	○ Diabetes 1.86, 0.53 – 6.53		○ Diabates 3.90, 1.58 – 9.63
Elhadi et al., 2021 (Libya)	Median 69	465	P	○ Age ≥60 vs <60 = 2.28, 1.53 – 3.39		

				○ Male 1.33, 0.92 – 1.93<br>○ Diabtes 1.31, 0.90 – 1.91<br>○ Cardiovascular disease 0.92, 0.58 – 1.44<br>○ Hypertension 1.02, 0.70 – 1.48<br>○ Chronic lung disease 0.80, 0.41 – 1.59<br>○ Asthma 0.65, 0.36 – 1.16<br>○ Chronic kidney disease 1.51, 0.78 – 2.91<br>○ Cancer 3.41, 0.97 – 11.92		
Escobar et al., 2021 (Brazil)		50824	CS	○ Male 1.93, 1.69 – 2.19		
Escosteguy et al., 2021 (Brazil)	Median 55	376	CS	○ Male 1.64, 1.06 – 2.53<br>○ Cardiovascular disease 1.31, 0.85 – 2.04<br>○ Diabetes 1.41, 0.87 – 2.29<br>○ Cancer 2.58, 1.48 – 4.52<br>○ Chronic lng disease 0.15, 0.04 – 0.63<br>○ Chronic liver disease 4.02, 1.32 – 12.26		
Etienne et al., 2020 (Paris)	Median 54 IQR 47-60	54	P		○ Age >65 vs ≤65= 0.92, 0.15 – 5.50<br>○ Male 2.36, 0.69 – 7.97	
Fadini et al., 2020 (Italy)	Mean 64.9	413	R	○ Diabetes 1.35, 0.71 – 2.59		
Falcone etal., 2020 (Italy)	Median 70	315	P	○ Male 2.13, 1.14 – 2.98<br>○ Chronic lung disease 4.43, 2.19 – 8.96		

	IQR 57-80			o Hypertension 2.41, 1.39 – 4.17<br>o Cardiovascular disease 2.86, 1.65 – 4.93<br>o Diabetes 1.42, 0.76 – 2.69<br>o Cerebrovascular disease 3.65, 1.72 – 7.78<br>o Cancer 1.38, 0.67 – 2.84<br>o Chronic kidney disease 3.76, 1.67 – 8.44		
Fava et al., 2020 (Spain)	Mean 59.7	104	R	o Male 0.79, 0.33 – 1.90<br>o Diabetes 1.69, 0.68 – 4.21<br>o Obesity 2.24, 0.88 – 5.69<br>o Cardiovascular disease 1.81, 0.72 – 4.52<br>o Chronic lung disease 6.48, 2.08 – 20.20<br>o Cancer 5.28, 1.17 – 23.85		
Ferguson et al., 2020 (USA)	Median 60.4	72	R			o Male 1.68, 0.54 – 5.54<br>o Smoking 1.34, 0.33 – 5.0<br>o Hypertension 2.60, 0.81 – 8.54<br>o Diabetes 3.65, 1.07 – 13.84<br>o Chronic renal disease 0.67, 0.06 – 3.95<br>o Cerebrovascular disease 2.46, 0.03 – 100.75
Fernandes et al., 2021 (USA)	Medain 10 IQR 1-17	281	R		o Age (continuous) 1.09, 1.01- 1.18<br>o Obesity 3.66, 1.14 – 11.78<br>o Male 0.91, 0.45 – 1.89	
Ferrando et al., 2020 (Spain)		663	P	o Age (continuous) 1.05, 1.01 – 1.09		

				○ Diabetes 1.83, 0.42 – 7.83<br>○ Chronic kidney disease 0.91, 0.22 – 3.69<br>○ Cancer 1.86, 0.36 – 9.67<br>○ Male 0.93, 0.65 – 1.32<br>○ Hypertension 1.50, 1.07 – 2.09<br>○ Asthma 0.69, 0.23 – 2.14<br>○ Cardiovascular disease 4.64, 1.15 – 18.74<br>○ Chronic lung disease 1.07, 0.48 – 2.42		
Ferrando-Vivas et al., 2021 (UK)	Median 60 IQR 51-68	9990	R	○ Male 1.30, 1.19 – 1.42		
Ferrari et al., 2020 (Brazil)	Median 61	198	p	○ Age ≥60 vs <60 = 3.1, 1.1 – 9.7<br>○ Male 1.47, 0.68 – 3.16<br>○ Hypertension 1.79, 0.84 – 3.83<br>○ Cardiovascular disease 2.51, 0.94 – 6.68<br>○ Diabetes 3.32, 1.49 – 7.35<br>○ Smoking 3.4, 1.3 – 8.6		
Fox et al., 2020 (USA)	Mean 66.2	355	R	○ Age (continuous) 1.03, 1.00 – 1.07<br>○ Male 1.36, 0.62 – 2.99<br>○ Chronic lung disease 1.15, 0.38 – 3.48<br>○ Asthma 0.71, 0.07 – 6.67		

				○ Hypertension 0.52, 0.13 – 2.02<br>○ Chronic kidney disease 1.44, 0.61 – 3.40		
Foy et al., 2020 (USA)	Mean 62	1641	R	○ Male 1.28, 0.98 – 1.66<br>○ Chronic lung disease 16.81, 5.44 – 51.98<br>○ Diabetes 6.86, 3.59 – 13.11<br>○ Hypertension 8.75, 5.09 – 15.03<br>○ Chronic kidney disease 13.96, 6.12 – 31.88		
Frontera et al., 2020 (USA)		4645	R	○ Age (continuous) 1.05, 1.03 – 1.07<br>○ Male 0.79, 0.53 – 1.18<br>○ Chronic kidney disease 0.82, 0.44 – 1.53<br>○ Hypertension 0.70, 0.47 – 1.04		
Fu et al., 2020 (China)	Mean 46.6	75	R	○ Male 1.14, 0.36 – 3.56		
Galiero et al., 2020 (Italy)	Mean 65	618	R	○ Age (continuous) 1.05, 1.02 – 1.07<br>○ Male 2.63, 1.42 – 4.90<br>○ Chronic kidney disease 1.01, 0.44 – 2.34<br>○ Chronic liver disease 5.88, 2.39 – 14.46<br>○ Cancer 2.62, 1.21 – 5.68<br>○ Hypertension 1.30, 0.89 – 1.89<br>○ Diabetes 1.48, 0.94 – 2.34<br>○ Smoking 1.35, 0.84 – 2.16		

				○ Chronic respiratory disease 2.55, 1.61 – 4.04		
Gao et al., 2020 (China)		2877	R	○ Hypertension 3.79, 2.21 – 6.53	○ Hypertension 1.86, 1.56 – 2.23	
Gao et al., 2020 (China)	Median 60	139	R	○	○ Age ≥65 vs <65 = 2.72, 1.21- 6.12<br>○ Hypertension 3.36, 1.41 – 9.52<br>○ Diabetes 16.53, 2.07 – 132.2<br>○ Chronic lung disease 4.53, 1.21 – 16.93	
Genet et al., 2020 (France)	Mean 86.3	201	R	○ Cancer 1.59, 0.76 – 3.34<br>○ Stroke 1.66, 0.85 – 3.24<br>○ Cardiovascular disease 1.11, 0.59 – 2.04<br>○ Hypertension 0.91, 0.49 – 1.65<br>○ Chronic lung disease 0.97, 0.43 – 2.19<br>○ Diabetes 1.78, 0.87 – 3.64<br>○ Malnutrition 0.82, 0.44 – 1.52		
Ghaffari et al., 2021 (Iran)	Mean 61.9	361	R		○ Male 0.77, 0.49 – 1.20<br>○ Diabetes 1.78, 1.07 – 2.98<br>○ Hypertension 1.75, 1.07 – 2.87<br>○ Cardiovascular disease 1.42, 0.78 – 2.58<br>○ Lung disease 1.01, 0.36 – 2.79<br>○ Cancer 1.22, 0.41 – 3.59<br>○ Chronic kidney disease 1.98, 0.63 – 6.15<br>○ Stroke 2.06, 0.56 – 7.54	
Giannoglou et al., 2020 (Greece)	Mean 60.4	512	R	○ Male 1.46, 0.88 – 2.43<br>○ Age ≥65 vs <65 = 8.22, 3.88 – 17.42<br>○ Cardiovascular disease 2.56, 1.27 – 5.15	○	

				o Hypertension 3.60, 2.21 – 5.88<br>o Diabetes 2.11, 1.13 – 3.96<br>o Asthma 0.85, 0.42 – 1.75<br>o Chronic kidney disease 6.15, 2.73 – 13.82		
Goicoechea et al., 2020<br>(Spain)	Mean 71	36	R	o Male 0.56, 0.13 – 2.41		
Gomez et al., 2021<br>(USA)		8108	R	o Male 1.96, 1.34 – 2.90<br>o Age (continuous)1.05, 1.04 – 1.07		
González-Gancedo et al., 2021<br>(Spain)	Mean 67.9	1987	R	o Age (continuous) 1.09, 1.07 – 1.19		
Goodall et al., 2020<br>(UK)	Medain 69<br>IQR 56-80	981	R	o Male 1.02, 0.78 – 1.34<br>o Diabetes 1.51, 1.15 – 1.97<br>o Hypertension 1.88, 1.45 – 2.46<br>o Cardiovascular disease 2.12, 1.57 – 2.85<br>o Cancer 1.75, 0.97 – 3.14<br>o Asthma 0.95, 0.58 – 1.53		
Grasselli et al., 2020<br>(Italy)	Median 63<br>IQR 56-69	3988	R	o Age ≥64 vs <64 = 3.49, 3.07 – 3.98<br>o Male 1.28, 1.10 – 1.51<br>o Hypertension 2.02, 1.78 – 2.30<br>o Cardiovascular disease 2.11, 1.75 – 2.55<br>o Diabetes 2.07, 1.71 – 2.51<br>o Cancer 1.75, 1.39 – 2.21		

				○ Chronic lung disease 2.82, 1.78 – 4.45<br>○ Chronic kidney disease 4.89, 2.83 – 8.44<br>○ Chronic liver disease 1.02, 0.66 – 1.56		
Gu T et al., 2020 (USA)	Mean 53	1139	R	○ Age ≥65 vs <65 = 8.77, 5.34 – 14.41<br>○ Male 2.12, 1.35 – 3.33<br>○ Smoking 1.67, 1.06 – 2.62		○ Age ≥65 vs <65 = 2.50, 1.88 – 3.32<br>○ Male 2.25, 1.52 – 3.34<br>○ Smoking 1.36, 0.90 – 2.07<br>○ Cardiovascular disease 1.21, 0.71 – 2.05<br>○ Respiratory disease 0.98, 0.60 – 1.61<br>○ Cancer 0.93, 0.60 – 1.44<br>○ Diabetes 1.50, 0.99 – 2.28<br>○ Kidney disease 2.74, 1.76 – 4.26<br>○ Liver disease 0.95, 0.52 – 1.73
Guarneri et al., 2021 (Italy)	Median 70<br>IQR 25-92	170	R	○ Male 0.71, 0.37 – 1.36<br>○ Age (continuous) 1.03, 1.00 – 1.05<br>○ Smoking 0.75, 0.29 – 1.91<br>○ Hypertension 1.68, 0.88 – 3.21		
Guo et al., 2020 (China)	>65	14238	R	○ Male 1.92, 1.56 – 2.36		
Gupta et al., 2020 (USA)	Mean 60.5	2215	R	○ Male 1.50, 1.19 – 1.90<br>○ Hypertension 1.06, 0.83 – 1.36<br>○ Diabetes 1.14, 0.91 – 1.43<br>○ Chronic lung disease 1.39, 0.95 – 2.04<br>○ Smoking 1.21, 0.76 – 1.93<br>○ Cancer 2.15, 1.35 – 3.43		

				○ Age >=60 vs <60 = 2.91, 2.41 – 3.51<br>○ Asthma 0.65, 0.48 – 0.86<br>○ Chronic liver disease 1.45, 0.90 – 2.31<br>○ Chronic kidney disease 1.75, 1.36 – 2.26		
Haase et al., 2020 (Denmark)	Mean 68	323	R	○ Male 1.72, 0.99 – 2.96<br>○ Hypertension 1.53, 0.96 – 2.41<br>○ Chronic lung disease 2.15, 1.21 – 3.81<br>○ Chronic kidney disease 1.95, 0.98 – 3.86<br>○ Diabetes 1.13, 0.64 – 1.98<br>○ Cancer 3.42, 1.14 – 10.28		
Halvatsiotis et al., 2020 (Greece)	Median 65.5	90	R	○ Male 1.07, 0.34 – 3.38<br>○ Chronic pulmonary disease 0.81, 0.15 – 4.27<br>○ Cancer 0.38, 0.04 – 3.38<br>○ Chronic kidney disease 8.21, 0.81 – 83.03<br>○ Cardiovascular disease 0.59, 0.17 – 1.99<br>○ Diabetes 2.71, 0.91 – 8.08<br>○ Hypertension 0.81, 0.32 – 2.01<br>○ Smoking 1.87, 0.71 – 4.95<br>○ Obesity 2.03, 0.79 – 5.19		

				○ Age >65 vs ≤65 = 0.65, 0.25 – 1.62		
Hashemi et al., 2020 (USA)	MEAN 63.3	363	R	○ Age (continuous) 1.08, 1.05 – 1.12<br>○ Obesity 1.03, 0.51 – 2.09<br>○ Male 1.62, 0.80 – 3.26<br>○ Cardiovascular disease 0.98, 0.46 – 2.09<br>○ Hypertension 2.20, 0.88 – 5.52<br>○ Diabetes 1.38 , 0.68 – 2.79		○ Age (continuous) 1.01, 0.99 – 1.02<br>○ Obesity 1.26, 0.79 – 1.98<br>○ Male 1.51, 0.96 – 2.38<br>○ Cardiovascular disease 0.88, 0.49 – 1.58<br>○ Hypertension 0.93, 0.55 – 1.60<br>○ Diabetes 1.22, 0.74 – 2.00
Hatami et al., 2021 (Iran)	Mean 59.52	665	R	○ Male 1.48, 0.98 – 2.24<br>○ Age ≥60 vs <60 = 2.68, 1.74 – 4.14<br>○ Obesity 0.81, 0.44 – 1.48<br>○ Diabetes 1.39, 0.91 – 2.14<br>○ Hypertension 1.90, 1.26 – 2.85<br>○ Cardiovascular disease 1.70, 1.07 – 2.70<br>○ Chronic kidney disease 1.07, 0.51 – 2.28<br>○ Chronic lung disease 2.09, 0.63 – 6.92<br>○ Asthma 1.75, 0.72 – 4.27<br>○ Cancer 0.81, 0.37 – 1.77<br>○ Cerebrovascular disease 2.31, 1.06 – 5.06		○
He et al., 2020 (China)		1187	R		○ Age (continuous) 1.07, 1.05 – 1.08<br>○ Male 2.52, 1.80 – 3.54	

Hendra et al., 2021 (UK)	Mean 64.1	148	R	○ Age (continuous) 1.05, 1.00 – 1.10<br>○ Male 1.47, 0.51 – 4.34<br>○ Diabetes 2.20, 0.64 – 7.49<br>○ Hypertension 3.58, 0.57 – 22.69<br>○ Cardiovascular disease 0.77, 0.26 – 2.25<br>○ Lung disease 1.01, 0.21- 18.74		
Hoffmann et al., 2021 (Germany, Spain, Italy)	≥20	175	R		○ Male 1.21, 0.50 – 2.90<br>○ Age ≥60 vs <60 = 1.85, 0.79 – 4.31	
Hu F et al., 2021 (China)	Mean 47.53	91	P		○ Male 2.89, 1.07 – 7.83<br>○ Hypertension 2.75, 0.89 – 8.43<br>○ Diabetes 4.78, 1.16 – 19.75	
Hu J et al., 2020 (China)		252	R	○ Male 5.21, 2.41 – 11.27<br>○ Smoking 1.27, 0.36 – 4.41<br>○ Hypertension 1.05, 0.55 – 2.00<br>○ Cardiovascular disease 2.13, 0.93 – 4.91<br>○ Diabetes 0.85, 0.35 – 1.03		
Hu X et al., 2021 (China)	Mean 44	122	R		○ Male 4.41, 1.19 – 16.24	
Huang R et al., 2020 (China)	Median 44 IQR 33-54	202	R		○ Age >60 vs ≤60 = 2.09, 0.70 – 6.22<br>○ Male 2.29, 0.86 – 6.07<br>○ Obesity 6.90, 2.38 – 19.99<br>○ Hypertension 0.53, 0.12 – 2.42<br>○ Diabetes 8.14, 2.84 – 23.34<br>○ Smoking 1.11, 0.23 – 5.22	
Huang S et al., 2020 (China)	Median 62	310	R	○ Age (continuous) = 1.04, 1.02 – 1.06<br>○ Hypertension 1.26, 0.68 2.33	○ Age (continuous) 1.05, 0.68 – 2.33<br>○ Hypertension 1.56, 0.93 – 2.62<br>○ Male 1.91, 1.16 - 3.13	

	IQR 49-70			○ Male 2.10, 1.12 – 3.94		
Huang Y et al., 2020 (China)		151	R	○ Male 6.5, 1.41 – 29.90<br>○ Hypertension 3.73, 1.24 – 11.18<br>○ Chronic lung diseae 8.4, 2.48 – 28.37		
Huang Y et al., 2020 (China)	Median 64	1255	R			○ Age ≥65 vs <65 = 2.42, 1.80 – 3.24<br>○ Male 2.34, 1.73 – 3.17<br>○ Diabetes 1.48, 1.05 – 2.09<br>○ Hypertension 1.67, 1.25 – 2.22<br>○ Cerebrovascular disease 2.10, 1.21 – 3.64<br>○ Cancer 0.81, 0.43 – 1.52
Hussein et al., 2020 (USA)	Mean 60.6 SD 15.7	502	R	○ Asthma 0.69, 0.30 – 1.58		
Hwang et al., 2020 (South Korea)	Adult	103	R	○ Male 1.82, 0.73 – 4.15<br>○ Hypertension 1.74, 0.69 – 4.39<br>○ Diabetes 3.11, 1.24 – 7.80<br>○ Chronic kidney disease 0.58, 0.15 – 2.23<br>○ Chronic lung disease 8.92, 1.61 – 49.35<br>○ Cancer 2.62, 0.64 – 10.64<br>○ Cardiovascular disease 3.55, 1.03 – 12.21		
Iaccarino et al., 2020 (Italy)	Mean 68.2	2378	CS	○ Male 1.87, 1.41 – 2.30<br>○ Hypertension 1.31, 1.04 – 1.66<br>○ Diabetes 1.17, 0.89 – 1.55		

				○ Chronic kidney disease 1.58, 0.14 – 2.43<br>○ Cardiovascular disease 1.12, 0.80 – 1.55<br>○ Obesity 2.47, 1.72 – 3.55		
Iaccarino et al., 2020 (Italy)	Mean 66.5	1761	CS	○ Male 1.13, 0.82 – 1.56<br>○ Hypertension 2.42, 1.73 – 3.40<br>○ Obesity 0.99, 0.53 – 1.85<br>○ Diabetes 2.76, 1.96 – 3.87<br>○ Chronic lung disease 2.43, 1.54 – 3.83<br>○ Chronic kidney disease 4.74, 2.97 – 7.59		
Imam Z et al., 2020 (USA)	Mean 61 SD 16.3	1305	R	○ Age ≥60 vs <60 = 1.93, 1.26 – 2.94<br>○ Smoking 1.19, 0.55 – 2.60<br>○ Hypertension 1.43, 1.05 – 1.95<br>○ Diabetes 1.24, 0.90 – 1.70<br>○ Chronic kidney disease 1.86, 1.30 – 2.64<br>○ Chronic lung disease 2.23, 1.41 – 3.52<br>○ Cancer 1.84, 1.09 – 3.11<br>○ Asthma 1.18, 0.71 – 1.96<br>○ Cerebrovascular disease 2.11, 1.30 – 3.43<br>○ Chronic liver disease 2.78, 0.51 – 15.3		

				○ Immunosuppression 2.49, 0.76 – 8.15		
Incerti et al., 2021 (USA)	Median 62 IQR 49-75	17086	R	○ Male 1.28, 1.17 – 1.41<br>○ Smoking 1.28, 1.16 – 1.41<br>○ Cancer 1.78, 1.58 – 2.02<br>○ Cerebrovascular disease 2.44, 2.15 – 2.76<br>○ Chronic pulmonary disease 3.08, 2.78 – 3.42<br>○ Diabetes 1.65, 1.50 – 1.81<br>○ Hypertension 2.76, 2.48 – 3.07<br>○ Chronic kidney disease 3.09, 2.81 – 3.42		
Inciardi et al., 2020 (Italy)	Mean 67	99	R	○ Male 1.82, 0.33 – 10.07<br>○ Hypertension 0.56, 0.16 – 2.01<br>○ Diabetes 3.47, 1.02 – 11.82<br>○ Chronic lung disease 4.26, 0.70 – 25.93<br>○ Chronic kidney disease 6.44, 1.74 – 23.84<br>○ Cancer 2.72, 0.75 – 9.82		
Islam et al., 2020 (Bangladesh)	Median 37 IQR 28-49	1016	R	○ Age ≥60 vs <60 = 7.55, 3.19 – 17.92<br>○ Male 1.79, 0.71 – 4.54<br>○ Smoking 0.85, 0.28 – 2.51<br>○ Chronic lung disease 8.14, 3.53 – 18.74<br>○ Diabetes 2.71, 1.18 – 6.24		

				○ Cardiovascular disease 5.05, 1.64 – 15.48<br>○ Hypertension 3.53, 1.53 – 8.16		
Jain et al., 2021 (India)	Mean 74<br>SD 15.4	63	CS		○ Age (continuous)= 1.01, 0.98 – 1.04<br>○ Smoking 0.48, 0.08 – 2.84<br>○ Diabetes 1.29, 0.35 – 4.78<br>○ Hypertension 1.63, 0.50 – 5.29<br>○ Chronic lung disease 1.03, 0.06 – 17.28	
Jang et al., 2020 (Korea)	Mean 56.9<br>SD 17	110	R		○ Age ≥ 70 vs <70 = 3.37, 0.26 – 43.58<br>○ Male 2.98, 0.23 – 38.81<br>○ Hypertension 1.00, 0.09 – 10.78	
Jegede et al., 2020 (USA)	Adults	98	R	○ Male 0.82, 0.34 – 2.00<br>○ Age >60 vs ≤60 = 7.93, 2.91 – 21.59		
Ji XY et al., 2021 (China)	Mean 66.4	27	R		○ Male 0.78, 0.15 – 4.21<br>○ Smoking 0.57, 0.08 – 3.64<br>○ Diabetes 0.74, 0.11 – 4.86	
Jinag N et al., 2021 (China)	Medain 45	289	CS		○ Age≥65 vs <65 = 2.72, 1.31 – 5.63<br>○ Male 1.87, 1.00 – 3.52<br>○ Smoking 2.25, 0.36 – 14.09<br>○ Diabetes 3.31, 1.12 – 9.75<br>○ Cardiovascular disease 3.53, 0.98 – 12.64<br>○ Chronic lung disease 3.16, 0.97 – 10.25<br>○ Cancer 1.43, 0.23 – 8.75<br>○ Obesity 1.07, 0.09 – 11.97	
Jiang Y et al., 2020 (China)	Median 70	281	R	○ Hypertension 3.21, 1.21 – 8.51		

	IQR 65-77			○ Diabetes 3.63, 1.21 – 10.94		
Jimeno et al., 2020 (Spain)	Median 67.2	45	R	○ Male 0.6, 0.3 – 1.3<br>○ Hypertension 2.6, 1.1 – 2.5<br>○ Cardiovascular disease 2.3, 1.1 – 4.6<br>○ Diabetes 2.4, 1.05 – 5.8<br>○ Chronic lung disease 3.7, 1.4 – 9.8<br>○ Smoking 2.4, 0.6 – 9.6		
Jorgensen at al., 2020 (Norway)	Median 58	34	P			○ Diabetes 0.77, 0.07 – 8.52<br>○ Cardiovascular disease 3.33, 0.63 – 17.51<br>○ Chronic kidney disease 2.75, 0.33 – 22.92
Jose et al., 2021 (Saudi Arabia)		350	R	○ Diabetes 2.09, 1.18 – 3.72<br>○ Age (continuous) 1.04, 1.02 – 1.06<br>○ Male 0.59, 0.36 – 0.94<br>○ Immunosupression 1.24, 0.29 – 5.26		○
Jung et al., 2020 (Multinational)	>=70	324	P	○ Male 1.46, 0.90 – 2.35<br>○ Chronic kidney disease 2.35, 1.25 – 4.40<br>○ Chronic lung disease 1.30, 0.79 – 2.15<br>○ Hypertension 1.02, 0.64 – 1.61<br>○ Diabetes 1.28, 0.79 – 2.06<br>○ Cardiovascular disease 1.31, 0.75 – 2.27		

Kaeuffer et al., 2020 (France)	Mean 66	1045	P	○ Male 1.7, 1.1- 2.7<br>○ Hypertension 0.6, 0.3-0.9<br>○ Diabetes 1.7, 1.0 – 2.7<br>○ Chronic lung disease 0.9, 0.5 – 1.5<br>○ Immunosuppression 3.8, 1.6 – 7.7<br>○ Chronic kidney disease 2.3, 1.3 – 3.9	○ Male 2.52, 1.94 – 3.28<br>○ Hypertension 1.14, 0.89 – 1.47<br>○ Diabetes 1.20, 0.91 – 1.59<br>○ Smoking 0.63, 0.31 – 1.30<br>○ Cardiovascular disease 0.88, 0.60 – 1.31<br>○ Chronic lung disease 1.30, 0.93 – 1.81<br>○ Chronic kidney disease 1.06, 0.71 – 1.57<br>○ Immunosuppression 1.36, 0.76 – 2.44<br>○ Cancer 0.99, 0.66 – 1.48	
Kalligeros et al., 2020 (USA)	Median 60	103	R			○ Male 2.40, 0.87 – 6.64<br>○ Diabetes 1.91, 0.71 – 5.19<br>○ Hypertension 0.79, 0.27 – 2.28<br>○ Cardiovascular disease 1.52, 0.51 – 4.51<br>○ Chronic lung disease 1.50, 0.47 – 4.82<br>○ Chronic renal disease 0.75, 0.20 – 2.71<br>○ Cancer 2.94, 0.69 – 12.51
Karahan et al., 2020 (Turkey)	Mean 63.5	149	R	○ Age (continuous) 1.03, 1.01 – 0.06<br>○ Male 1.46, 0.76 – 2.81<br>○ Smoking 2.16, 0.198 – 4.30<br>○ Diabetes 2.40, 1.23-4.68<br>○ Chronic kidney disease 3.22, 1.35 – 7.66<br>○ Cardiovascular disease 4.83, 1.51 – 15.49	○ Male 1.37, 0.69 – 2.75<br>○ Smoking 2.48, 1.11 – 5.55<br>○ Hypertension 4.66, 2.22 – 9.80<br>○ Diabetes 2.69, 1.25 – 5.77<br>○ Chronic lung disease 1.29, 0.39 – 4.31<br>○ Cancer 1.36, 0.50 – 3.72<br>○ Chronic kidney disease 8.10, 1.83 – 35.69	○
Karimi et al., 2020 (Iran)	Mean 35.3	43	R	○ Male 1.76, 0.36 - 8.55		

Karlsson et al., 2020 (Denmark)	Mean 85	102	R	○ Male 4.0, 1.8 – 9.5		
Kaya et al., 2021 (Japan)		102	R	○ Male 1.53, 0.44 – 5.38		
Khan et a., 2020 (Saudi Arabia)	Median 34	648	R		○ Male 2.02, 1.22 – 3.34<br>○ Age ≥65 vs <65 = 3.15, 1.40 – 7.09<br>○ Smoking 0.96, 0.50 – 1.85<br>○ Diabetes 2.37, 1.28 – 4.37<br>○ Hypertension 2.28, 1.23 – 4.20<br>○ Chronic kidney disease 1.50, 0.32 – 6.69<br>○ Chronic respiratory disease 2.24, 1.23 – 4.08<br>○ Cardiovascular disease 4.30, 10.51 – 1.76<br>○ Cancer 2.98, 1.03 – 8.61	
Kim D et al., 2020 (Korea)		9148	R	○ Male 2.18, 1.50 – 3.17<br>○ Diabetes 1.82, 1.25 – 2.67<br>○ Hypertension 1.32, 0.88 – 1.99		
Kim E et al., 2021 (Korea)	Median 71	110	R	○ Age ≥65 vs <65 = 1.88, 0.81 - 4.39<br>○ Male 0.91, 0.42 - 1.97<br>○ Smoking 1.40, 0.61 – 3.17<br>○ Hypertension 1.08, 0.51 -2.20<br>○ Chronic kidney disease 4.10, 1.03 – 16.42<br>○ Cerebrovascular disease 0.88, 0.23 – 3.33<br>○ Cancer 2.16, 0.57 – 8.13<br>○ Cardiovascular disease 1.76, 6.93 – 0.44		

Kim S et al., 2020 (Korea)	MEDIAN 58	7057	R	○ Age >70 vs ≤70 = 4.77, 2.21 – 11.05<br>○ Male 2.16, 1.59 – 2.94<br>○ Asthma 1.87, 0.91 – 3.85<br>○ Chronic lung disease 2.26, 0.86 – 5.97<br>○ Chronic renal disease 8.30, 4.14 – 16.62<br>○ Smoking 1.58, 0.94 – 2.65<br>○ Diabets 2.59, 1.32 – 5.06<br>○ Cancer 4.06, 1.35 – 10.98<br>○ Cardiovascular disease 3.81, 0.99 – 13.06<br>○ Hypertension 2.16, 1.09 – 4.43		
Krause et al., 2020 (USA)		85	R	○ Age ≥60 vs <60 = 9.45, 1.43 – 62.31<br>○ Male 0.50, 0.18 – 1.41<br>○ Hypertension 1.97, 0.45 – 8.70<br>○ Diabetes 0.75, 0.19 – 2.86<br>○ Cancer 5.75, 1.16 – 28.44<br>○ Cardiovascular disease 2.25, 0.81 – 6.26		
Krishnamoorthy ey al., 2021 (USA)	≥15	799	R	○ Age (continuous) 1.06, 1.04 – 1.08<br>○ Hypertension 0.70, 0.40 – 1.20<br>○ Chronic lung disease 1.37, 0.76 – 2.47		

				○ Diabetes 1.77, 1.10 – 2.83<br>○ Cardiovascular disease 1.43, 0.75 – 2.74		
Krishnan et al., 2020 (USA)	Median 68	152	R	○ Age (continuous) 1.08, 1.04 – 1.12<br>○ Male 1.19, 0.61-2.33<br>○ Hypertension 2.56, 1.23 – 5.31<br>○ Diabetes 1.41, 0.73 – 2.74<br>○ Asthma 1.19, 0.49 – 2.91<br>○ Chronic lung disease 2.04, 0.75 – 5.51<br>○ Chronic renal disease 0.93, 0.37 – 2.34<br>○ Smoking 1.13, 0.56 – 2.28		
Kunal et al., 2020 (India)	Mean 51.2	108	R	○ Male 0.91, 0.38 – 2.20<br>○ Hypertension 2.94, 1.24 – 7.91<br>○ Diabetes 2.38, 0.99 – 5.69<br>○ Cardiovascular disease 1.53, 0.47 – 5.02<br>○		
Kute et al., 2021 (India)	Mean 43	250	R	○ Male 1.47, 0.42 – 5.13<br>○ Hypertension 1.16, 0.54 – 2.52<br>○ Dibetes 2.03, 0.91 – 4.51		
Laake et al., 2020 (Noraway)	Median 63	217	P	○ Male 0.67, 0.38 – 1.19<br>○ Age ≥65 vs <65 = 3.44, 1.99 – 5.96		

				o Cardiovascular disease 2.13, 1.26 – 3.61<br>o Diabetes 2.78, 1.53 – 5.06<br>o Asthma 1.20, 0.59 – 2.44<br>o Immunodeficiency 1.38, 0.57 – 3.33<br>o Chronic kidney disease 4.18, 1.82 – 9.58<br>o Chronic lung disease 2.26, 0.97 – 5.27		
Lara et al., 2020 (USA)	Median 64	121	R	o Smoking 5.71, 2.07 – 15.78<br>o Hypertension 1.95, 0.69 – 5.49<br>o Diabetes 1.66, 0.61 – 4.47<br>o Asthma 2.37, 0.56 – 10.08<br>o Chronic kidney disease 4.80, 1.16 – 19.81		
Lee J et al., 2021 (Korea)		38,919	R	o Male 2.12, 1.47 – 3.12<br>o Diabetes 2.24, 1.56 – 3.22		
Lee L et al., 2020 (UK)	Median 69	800	P	o Age (continuous) 1.67, 1.19 – 2.34<br>o Male 1.67. 1.19 – 2.34<br>o Cardiovascular disease 2.32, 1.47 – 3.64<br>o Chronic lung disease 1.80, 1.00 – 3.27<br>o Diabetes 1.61, 1.03 – 2.43<br>o Hypertension 1.95, 1.36 – 2.80		

Lee S et al., 2020 (Korea)		7272	R	○ Age 1.06, 1.05 – 1.07<br>○ Male 2.31, 1.58 – 3.37<br>○ Asthma 0.99, 0.58 – 1.70		
Lee Y et al., 2020 (Korea)	Median 61	1005	R	○ Age ≥65 vs <65 = 4.96, 2.65 – 9.30<br>○ Diabetes 2.29, 1.44 – 3.64<br>○ Chronic lung disease 4.52, 1.85 – 11.02	○ Age ≥65 vs <65 = 4.32, 2.73 – 6.84<br>○ Diabetes 2.26, 1.43 – 3.58<br>○ Chronic renal disease 4.20, 1.20 – 14.71	
Lei C et al., 2020 (China)	Median 48	297	R		○ Male 1.93, 1.05 – 3.56	
Lei F et al., 2020 (China)	Median 56	5771	R		○ Male 1.51, 1.32 – 1.71	
Li G et al., 2020 (Multinational)	Median 66	1075	R	○ Male 1.85, 1.23 – 2.80<br>○ Hypertension 3.74, 2.45 – 5.72<br>○ Diabetes 2.30, 1.46 – 3.63<br>○ Cardiovascular disease 1.36, 0.81 – 2.31<br>○ Cerebrovascular disease 0.37, 0.13 – 0.99<br>○ Cancer 1.28, 0.51 – 3.16		
Li G et al., 2021 (China)		145	R	○ Male 1.49, 0.66 – 3.36<br>○ Hypertension 1.60, 0.71 – 3.63<br>○ Diabetes 1.04, 0.39 – 2.73<br>○ Chronic lung disease 2.45, 0.64 – 9.35<br>○ Cerebrovascular disease 2.10, 0.44 – 9.93		

Li HY et al., 2020 (China)	Mean<br>58.8	132	R		○ Age ≥60 vs <60 = 1.26, 0.60 – 1.26<br>○ Male 5.31, 2.21 – 12.75<br>○ Cancer 3.05, 0.65 – 14.31	
Li K et al., 2020 (China)	Medain<br>57	102	R	○ Age ≥65 vs <65 = 4.17, 1.34 – 13.02<br>○ Male 2.22, 0.65 – 7.69<br>○ Diabetes 0.88, 0.18 – 4.34<br>○ Hypertension 2.30, 0.75 – 7.03<br>○ Smoking 0.96, 0.11 – 8.63	○	
Li T et al., 2020 (China)	Mean<br>69.2	312	R	○	○ Male 1.28, 0.79 – 2.07<br>○ Smoking 1.62, 0.77 – 3.39<br>○ Hypertension 6.16, 3.46 – 10.97<br>○ Diabetes 1.64, 1.02 – 2.65<br>○ Cardiovascular disease 2.65, 4.40 – 1.60<br>○ Chronic lung disease 1.40, 0.62 – 3.13<br>○ Cerebrovascular disease 1.67, 0.67 – 4.17<br>○ Chronic kidney disease 1.33, 0.37 – 4.81<br>○ Chronic liver disease 1.13, 0.32 – 3.96	
Lian et al., 2020 (China)	Mean<br>68.28	788	R		○ Age ≥60 vs <60 = 4.61, 2.81 – 7.55	
Lievre et al., 2020 (France)	Median<br>67	1289	P	○ Age ≥65 vs <65= 1.68, 1.30 – 2.16<br>○ Male 1.61, 1.25 – 2.08<br>○ Obesity 1.15, 0.82 – 1.61<br>○ Smoking 2.03, 1.59 – 2.59	○	

				o Chronic lung disease 2.15, 1.48 – 3.14<br>o Diabetes 1.40, 1.04 – 1.89<br>o Hypertension 1.78, 1.39 – 2.27		
Lim et al., 2021 (Singapore)	Median 59	275	R	o Male3.69, 1.50 – 9.08		
Ling et al., 2020 (UK)	Median 74 IQR 63-83	444	CS	o Age >74 vs ≤74 = 1.71, 0.80 – 3.70<br>o Asthma 0.18, 0.04 -0.94<br>o Diabetes 0.86, 0.41 – 1.81<br>o Male 0.68, 0.33 – 1.37<br>o Hypertension 1.25, 0.85 – 1.83		
Liu D et al., 2020 (China)	Median 63	599	P	o Male 1.51, 0.94 – 2.45<br>o Smoking 0.98, 0.55 – 1.75<br>o Hypertension 0.58, 0.27 – 1.26<br>o Diabetes 1.01, 0.46 – 2.21<br>o Cardiovascular disease 0.90, 0.43 – 1.88<br>o Chronic lung disease 0.83, 0.24 – 2.85<br>o Cerebrovascular disease 0.95, 0.21 – 4.27<br>o Chronic kidney disease 0.46, 0.11 – 1.98		
Liu J etal., 2021 (China)	Medain 62	122	R		o Male 1.04, 0.76 – 1.41<br>o Age ≥65 vs <65 = 1.46, 1.09 – 1.05<br>o Smoking 0.81, 0.13 – 5.04<br>o Hypertension 2.03, 0.93 – 4.46<br>o Diabetes 4.04, 0.87 – 18.81	

					○ Cerebrovascular disease 0.80, 0.21 – 3.01	
Liu S et al., 2020 (China)		721	R		○ Age ≥65 vs <65 = 6.56, 3.70 – 11.64<br>○ Hypertension 1.06, 0.47 – 2.40<br>○ Diabetes 1.64, 0.52 – 5.22<br>○ Male 1.69, 0.99 – 2.89	
Lohia et al., 2021 (USA)	Mean 64.11	1871	R	○ Chronic respiratory disease 1.36, 1.08 – 1.72<br>○ Smoking 1.14, 0.91 – 1.42	○	○ Chronic respiratory disease 1.34, 1.07 – 1.68<br>○ Smoking 1.25, 1.01 – 1.55
Long et al., 2020 (China)	Median 63.55	115	R	○ Age ≥60 vs<60 = 2.66, 0.83 – 8.47<br>○ Male 0.77, 0.31 – 1.92		
Lorente L et al, 2021a (Spain)		143	P	○ Male 0.48, 0.19 – 1.23<br>○ Diabetes 0.92, 0.35 – 2.40<br>○ Chronic lung disease 2.44, 1.02 – 11.58<br>○ Hypertension 1.21, 0.51 – 2.88<br>○ Smoking 1.96, 0.35 – 10.75		
Lorente L et al., 2021b (Spain)		3886	P	○ Male 0.52, 0.12 – 2.20<br>○ Dibetes 0.34, 0.04 – 2.99<br>○ Chronic lung disease 2.18, 0.20 – 23.36<br>○ Smoking 1.61, 0.16 – 16.08		
Louapre et al., 2020 (France)	Mean 44.6	348	R	○ Male 2.11, 1.23 – 3.63<br>○ Cardiovascular disease 5.72, 2.39 – 13.66<br>○ Diabetes 5.36, 1.93 – 14.95		

				o Smoking 0.65, 0.24 – 1.74<br>o Obesity 2.95, 1.25 – 6.94		
Lu Y et al., 2021 (China)	Medain<br>59	77	R	o Male 2.01, 0.77 – 5.19<br>o Smoking 0.45, 0.04 – 5.17<br>o Hypertension 1.12, 0.44 – 2.89<br>o Diabetes 0.71, 0.18 – 2.88		
Lucar et al., 2021 (USA)	Median<br>59	100	R	o Age ≥65 vs <65 = 0.59, 0.11 – 3.24<br>o Male 2.05, 0.55 – 7.52<br>o Smoking 5.85, 1.20 – 28.57<br>o Cardiovascular disease 0.93, 0.13 – 6.63<br>o Chronic kidney disease 3.11, 0.62 – 15.43<br>o Diabetes 5.94, 1.45 – 24.25<br>o Hypertension 0.42, 0.09 – 1.92		
Lunski et al., 2021 (USA)		5145	R	o Cancer 2.03, 1.44 – 2.87<br>o Age ≥ 65 vs <65 = 5.96, 4.71-7.54<br>o Male 2.15, 1.73 – 2.67<br>o Chronic kidney disease 3.84, 2.98 – 4.93<br>o Chronic lung disease 1.34, 0.96 – 1.88<br>o Diabetes 0.92, 0.70 – 1.21<br>o Hypertension 0.60, 0.47 – 0.78		

				○ Obesity 1.30, 1.03 – 1.63		
Luo H ey al., 2020 (China)	Median 46	625	R	○ Age ≥65 vs <65 = 6.56, 3.70 – 11.64		
Maguire et al., 2020 (UK)		224	R	○ Age ≥70 vs <70 = 4.49, 2.33 – 8.64<br>○ Male 1.55, 0.82 – 2.93<br>○ Hypertension 2.02, 1.08 – 3.78<br>○ Chronic kidney disease 2.04, 0.88 – 4.74<br>○ Chronic lung disease 1.48, 0.69 – 3.16<br>○ Smoking 1.06, 0.57 – 1.98<br>○ Alcohol 1.43, 0.61 – 3.33<br>○ Liver disease 0.87, 0.28 -2.75<br>○ Cancer 1.69, 0.41 - 7.02<br>○ Asthma 0.26, 0.09 - 0.76		
Mak et al., 2020 (Sweden)	Mean 69.2	2812	P	○ Age 1.11, 1.09 – 1.13<br>○ Male 1.48, 1.13 – 1.94<br>○ Smoking 1.29, 0.89 – 1.93		
Marengoni et al., 2020 (Italy)	≥70	91	R	○ Male 3.08, 0.87 – 12.80		
Marouf et al., 2021 (Qatar)	≥18	568	R	○ Male 0.82, 0.28 – 2.37<br>○ Smoking 5.07, 1.71 – 15.07<br>○ Diabetes 3.46, 1.18 – 10.13		○ Male 0.73, 0.37 – 1.44<br>○ Smoking 1.93, 0.85 – 4.41<br>○ Diabetes 3.24, 1.63 – 6.42
Martinez-Lopez et al., 2020 (Spain)	Median 71	167	R	○ Age >60 vs ≤60 = 3.0, 1.4 – 8.4<br>○ Male 3.8, 1.7 – 8.4		

				o Hypertension 1.7, 0.8 – 3.5<br>o Renal disease 4.6, 1.9 – 11.3<br>o Cardiovascular disease 1.22, 0.56 – 2.66<br>o Pulmonary disease 1.33, 0.54 – 3.29<br>o Diabetes 1.12, 0.48 – 2.63		
Martins et al., 2021 (Portugal)	Median<br>71	62	R	o Male 10.50, 1.24 – 88.59<br>o Hypertension 3.60, 0.70 – 18.46<br>o Diabetes 1.69, 0.46 – 6.13<br>o Cardiovascular disease 3.78, 0.98 – 14.56<br>o Cancer 7.60, 1.75 – 32.93<br>o Chronic lung disease 1.52, 0.26 – 9.03<br>o Chronic kidney disease 2.67, 0.39 – 18.17		
Mash et al., 2020 (South Africa)	Mean<br>46.3	1376	R	o Male 2.02, 1.37 – 2.98<br>o Diabetes 1.84, 1.24 – 2.73<br>o Obesity 1.58, 1.02 – 2.46<br>o Cardiovascular disease 2.85, 1.52 – 5.35<br>o Cancer 7.45, 1.87 – 29.89<br>o Chronic renal disease 5.16, 2.82 – 9.43<br>o Hypertension 3.95, 2.73 – 5.70		

				o Chronic lung disease 1.34, 0.59 – 3.03<br>o Asthma 1.11, 0.52 – 2.36<br>o Smoking 1.01, 0.46 – 2.21		
Mather et al., 2020 (USA)	Mean 67	878	R	o Obesity 5.87, 2.54 – 13.6<br>o Renal disease 2.50, 1.60 – 3.93<br>o Age (cont) 1.06, 1.04 – 1.08<br>o Cardiovascular disease 1.44, 0.80 – 2.62		
Mato et al., 2020 (international)	Median 63	198	R	o Age ≥65 vs <65 = 2.05, 1.05 – 4.02<br>o Male 0.77, 0.42 – 1.42<br>o Hypertension 1.35, 0.75 – 2.45<br>o Diabetes 2.89, 1.42 – 5.90<br>o Chronic lung disease 6.70, 2.47 – 18.18<br>o Asthma 3.41, 1.02 – 11.39<br>o Chronic renal disease 2.02, 0.95 – 4.30<br>o Smoking 1.07, 0.57 – 1.99		
Matsuzawa et al., 2020 (Japan)	Mean 60	151	R	o Hypetension 0.63, 0.15 – 2.34		o Hypertension 0.51, 0.15 – 1.48
Mayer et al., 2021 (Spain)	>15 years	348596	R	o Male 2.22, 1.90 – 2.60<br>o Hypertension 1.16, 0.98 – 1.37<br>o Diabetes 1.69, 1.43 – 1.99<br>o Obesity 1.08, 0.91 – 1.27		

				○ Chronic lung disease 1.18, 0.92 – 1.51<br>○ Cancer 1.19, 0.99 – 1.43<br>○ Smoking 0.98, 0.67 – 1.39<br>○ Cerebrovascular disease 0.84, 0.50 – 1.33		
Mehta et al., 2021 (USA)	Mean 82.7	482323	R	○ Male 1.45, 1.41 – 1.49<br>○ Cancer 1.20, 1.14 – 1.26<br>○ Cardiovascular disease 1.12, 1.08 – 1.16<br>○ Chronic renal disease 1.24, 1.20 – 1.29<br>○ Diabetes 1.07, 1.04 – 1.10<br>○ Malnutrition 1.02, 0.98 – 1.07<br>○ Chronic respiratory disease 1.02, 0.99 – 1.05		
Menager et al., 2021 (France)	82	Mean 88.8	P	○ Male 1.15, 0.29 – 4.63<br>○ Cancer 4.35, 1.00 – 18.93<br>○ Diabetes 0.38, 0.04 – 3.27<br>○ Hypertension 1.17, 0.27 – 5.08<br>○ Cerebrovascular disease 1.20, 0.23 – 6.44		
Mendes et al., 2020 (Switzerland)	235	Mean 86	R	○ Male 3.33, 1.88 – 5.89<br>○ Chronic lung disease 0.98, 0.40 – 2.39<br>○ Smoking 0.93, 0.52 – 1.65<br>○ Chronic kidney disease 1.10, 0.59 – 2.03		

				o Chronic liver disease 1.70, 0.71 – 4.08<br>o Diabetes 1.79, 0.96 – 3.36<br>o Stroke 1.02, 0.51 – 2.02<br>o Hypertension 1.18, 0.64 – 2.17		
Messika et al., 2021 (France)	35	Median 50.4	R	o Male 1.31, 0.19 – 9.02<br>o Age (continuous) 1.02, 0.94 – 1.10)		o Male 1.60, 0.40 – 6.46<br>o Age (continuous) 1.03, (0.98 – 1.10)
Moghaddam et al., 2020 (Germany)	33	Median (IQR) =77 (38, 94)	CS	o Male 0.63, 0.10 – 4.01<br>o Hypertension 1.00, 0.15 – 6.53<br>o Diabetes 2.88, 0.39 – 21.29<br>o Cardiovascular disease 0.93, 0.16 – 5.45<br>o Cerebrovascular disease 0.88, 0.08 – 9.29		
Morrison et al., 2020 (USA)	92	Median 64	R	o Male 1.98, 0.73 – 5.33<br>o Asthma 1.11, 0.31 – 3.99<br>o Chronic lung disease 0.31, 0.03 – 2.89<br>o Diabetes 0.67, 0.27 – 1.62<br>o Hypertension 0.79, 0.29 – 2.13		
Mostaza et al., 2020 (Spain)	404	Mean 85.2	R	o Male 2.31, 1.51 – 3.52<br>o Hypertension 0.69, 0.44 – 1.08<br>o Diabetes 1.20, 0.77 – 1.88<br>o Chronic lung disease 0.72, 0.42 – 1.22<br>o Chronic renal failure 0.80, 0.45 – 1.43		

				o Chronic liver disease 1.05, 0.38 – 2.96		
Murthy et al., 2021 (Canada)	811	Median 64	R	o Age ≥60 vs <60 = 3.69, 2.37 – 5.74<br>o Male 0.76, 0.54 – 1.08<br>o Hypertension 1.92, 1.36 – 2.72<br>o Diabetes 1.60, 1.10 – 2.32<br>o Chronic kidney disease 2.11, 1.34 – 3.32<br>o Asthma 1.34, 0.81 – 2.23<br>o Smoking 1.87, 0.92 – 3.81		o Age >70 vs ≤ 70 = 2.07, 1.09 – 3.93<br>o Male 1.63, 1.22 – 2.19<br>o Hypertension 1.06, 0.80 – 1.41<br>o Diabetes 1.24, 0.90 – 1.71<br>o Cardiovascular disease 0.94, 0.66 – 1.23<br>o Chronic kidney disease 3.29, 2.12 – 5.08<br>o Chronic liver disease 1.30, 0.63 – 2.70<br>o Asthma 1.09, 0.70 – 1.69<br>o Smoking 1.13, 0.58 – 2.20<br>o Obesity 2.85, 1.19 – 6.79<br>o Cancer 1.29, 0.60 – 2.74
Nachega et al., 2020 (Congo)	766	Median 46	R	o Male 1.50, 0.97 – 2.32<br>o Age ≥60 vs <60 = 6.86, 4.43 – 10.64<br>o Hypertension 3.15, 2.04 – 4.85<br>o Cardiovascular disease 4.78, 2.20 – 10.37<br>o Diabetes 3.53, 2.16 – 5.77<br>o Obesity 6.81, 3.48 – 13.33<br>o Chronic lung disease 1.39, 0.47 – 4.11<br>o Chronic kidney disease 10.43, 2.30 – 47.31<br>o Cancer 5.11, 0.84 – 30.97	o Age ≥60 vs <60 = 4.76, 3.31 – 6.85<br>o Male 1.39, 0.97 – 1.98<br>o Hypertension 3.66, 2.57 – 5.21<br>o Cardiovascular disease 7.77, 3.49 – 17.28<br>o Obesity 4.27, 2.22 – 8.23<br>o Diabetes 5.15, 3.36 – 7.89<br>o Chronic lung disease 2.69, 1.22 – 5.91<br>o Chronic kidney disease 2.28, 0.51 – 10.27<br>o Cancer 4.57, 0.76 – 27.57	o
Nachtigall et al., 2020 (Germany)	1904	Median 73	R	o Male 1.15, 0.90 – 1.47<br>o Diabetes 1.44, 1.04 – 1.98		o Male 1.98, 1.58 – 2.49<br>o Age ≥60 vs <60 = 1.79, 1.37 – 2.34<br>o Diabetes 2.05, 1.54 – 2.72

				○ Cancer 2.36, 1.49 – 3.74<br>○ Cardiovascular disease 2.25, 1.76 – 2.88<br>○ Lung disease 2.12, 1.52 – 2.97		○ Cancer 1.26, 0.78 – 2.05<br>○ Cardiovascular disease 1.68, 1.34 – 2.11<br>○ Chronic lung disease 1.78, 1.29 – 2.45
Nakamura et al., 2020 (Japan)	32	Median 74	R	○ Age >70 vs ≤70 = 2.00, 0.41 – 9.74<br>○ Male 7.50, 0.81 – 69.7<br>○ Smoking 0.60, 0.13 – 2.67<br>○ Diabetes 1.59, 0.29 – 8.87<br>○ Hypertension 2.40, 0.54 – 10.69<br>○ Chronic lung disease 2.11, 0.25 – 17.49<br>○ Asthma 2.00, 0.11 – 35.41		
Namendys-Silva, 2021 (Mexico)	164	Mean 57.3	R	○ Age (continuous) 1.04, 1.01 – 1.07<br>○ Hypertension 2.41, 1.26 – 4.62<br>○ Diabetes 3.03, 1.51 – 6.09<br>○ Smoking 0.75, 0.32 – 1.80<br>○ Chronic lung disease 2.85, 0.29 – 28.02<br>○ Chronic renal failure 0.45, 0.08 – 2.54		
Ng et al., 2020 (USA)	10,482		R	○ Male 1.39, 1.23 – 1.58<br>○ Diabetes 1.16, 1.02 – 1.32<br>○ Hypertension 0.84, 0.73 – 0.98<br>○ Cancer 1.30, 1.07 – 1.57		

Nie et al., 2020 (China)	45	Median 66	R	○ Male 6.19, 0.71 – 54.16<br>○ Smoking 1.22, 0.26 – 5.72<br>○ Hypertension 5.69, 1.32 – 24.54<br>○ Diabetes 3.88, 0.65 – 22.96		
Nie Y et al., 2020 (China)					○ Male 0.84, 0.51 – 1.38<br>○ Cardiovascular disease 0.81, 0.31 – 2.14<br>○ Hypertension 0.49, 0.16 – 1.45	
Nunez-Gil, 2021 (Spain)	2798	Median 67	R	○ Cardiovascular disease 3.58, 2.95 – 4.34		
O'Horo et al., 2021 (USA)	7217	Median 39	R			○ Male 0.84, 0.55 – 1.30<br>○ Smoking 1.17, 0.73 – 1.86<br>○ Age ≥65 vs <65 = 1.55, 0.31 – 7.80
Okoh et al., 2020 (USA)	416	Median 62	R	○ Age 1.04, 1.01 – 1.06<br>○ Male 1.99, 1.19 – 3.37<br>○ Hypertension 2.21, 1.23 – 4.04<br>○ Chronic kidney disease 3.43, 1.78 – 6.80<br>○ Diabetes 1.19, 0.78 – 1.83<br>○ Cerebrovascular disease 2.07, 0.95 – 4.51<br>○ Cancer 2.72, 1.11 – 6.65		
Oto et al., 2021 (Turkey)	109	Mean 48.4	R	○ Age >60 vs ≤60 = 2.74, 0.81 – 9.27<br>○ Male 0.50, 0.16 – 1.55<br>○ Diabetes 1.37, 0.39 – 4.82<br>○ Hypertension 1.15, 0.29 – 4.51		○ Age >60 vs ≤60 = 5.75, 1.33 – 24.88<br>○ Male 0.67, 0.26 – 1.72<br>○ Diabetes 1.73, 0.61 – 4.90<br>○ Hypertension 2.24, 0.61 – 8.34<br>○ Cardiovascular disease 2.33, 0.75 – 7.21

				○ Cardiovascular disease 3.70, 1.04 – 13.08		
Ouldali et al., 2021 (France)	397	Children	P		○ Male 0.5, 0.2 – 1.3<br>○ Asthma 1.4, 0.2 – 5.3<br>○ Chronic respiratory disease 5.3, 0.7 – 26.3<br>○ Cardiovascular disease 2.1, 0.1 – 13.1<br>○ Obesity 2.5, 0.1 – 16.6	
Ozdemir et al., 2021 (Turkey)	350	Median 55	R	○ Male 0.88, 0.49 – 1.57<br>○ Smoking 0.99, 0.54 – 1.80<br>○ Hypertension 3.02, 1.67 – 5.45<br>○ Diabetes 2.36, 1.23 – 4.53<br>○ Chronic kidney disease 6.39, 2.35 – 12.57<br>○ Chronic lung disease 2.47, 1.17 – 5.18		
Ozturk ey al., 2020 (Turkey)	1210	Median 61	R	○ Male 1.57, 1.12 – 2.19<br>○ Diabetes 1.94, 1.40 – 2.70<br>○ Hypertension 2.56, 1.75 – 3.76<br>○ Chronic lung disease 2.10, 1.38 – 3.21<br>○ Cancer 1.57, 0.81 – 3.04<br>○ Chronic liver disease 2.49, 0.64 – 9.27<br>○ Smoking 0.93, 0.65 – 1.33		
Park et al., 2020a (Korea)	954	Median 55	R	○ Male 2.03, 1.29 – 3.20<br>○ Asthma 2.13, 0.74 – 6.13<br>○ Chronic lung disease 1.05, 0.26 – 4.30		

				○ Chronic kidney disease 2.19, 0.85 – 5.63<br>○ Cancer 2.12, 0.94 – 4.76<br>○ Diabetes 2.43, 1.51 – 3.90<br>○ Hypertension 1.48, 0.91 – 2.40		
Park et al., 2020b (South Korea)	1005	Median 72	R	○ Male 2.11, 1.22 – 3.65<br>○ Hypertension 1.36, 0.79 – 2.33<br>○ Diabetes 2.56, 1.47 – 4.46<br>○ Cardiovascular disease 1.37, 0.51 – 3.71<br>○ Chronic lung disease 4.13, 1.08 – 15.85<br>○ Chronic kidney disease 0.69, 0.14 – 3.25<br>○ Chronic liver disease 2.19, 0.75 – 6.38		
Parlak et al., 2021 (Turkey)	343	Mean 48.4	R	○ Male 1.06, 0.42 – 2.67<br>○ Diabetes 0.43, 0.08 – 2.25<br>○ Hypertension 0.82, 0.23 – 2.98<br>○ Chronic lung disease 1.17, 0.22 – 6.16<br>○ Chronic kidney disease 34.84, 4.11 – 295.58		○ Male 1.24, 0.68 – 2.26<br>○ Diabetes 0.53, 0.18 – 1.52<br>○ Hypertension 1.05, 0.45 – 2.44<br>○ Chronic lung disease 1.77, 0.51 – 6.14<br>○ Chronic kidney disease 5.39, 0.87 – 33.22
Pascual et al., 2020 (Spain)	24	Median 66.5	R	○ Male 0.97, 0.19 – 4.87<br>○ Age ≥ 65 vs <65 = 6.00, 1.02 – 35.38<br>○ Hypertension 0.83, 0.05 – 15.09<br>○ Diabetes 0.36, 0.07 – 1.88		

Passamonti et al., 2020 (Italy)	536	adults	R	○ Age ≥60 vs <60 = 3.17, 2.01 – 4.98<br>○ Male 1.39, 0.96 – 2.01<br>○ Lung disease 1.39, 0.74 – 2.61<br>○ Chronic liver disease 2.01, 1.00 – 4.04<br>○ Chronic kidney disease 2.45, 1.30 – 4.64<br>○ Diabetes 1.26, 0.76 – 2.09		
Patel et al., 2020 (UK)	645	Mean 62	R	○ Hypertesnsion 1.54, 1.02 – 2.32<br>○ Cardiovascular disease 2.11, 1.34 – 3.36<br>○ Chronic lung disease 1.22, 0.77 – 1.91<br>○ Diabetes 1.29, 0.85 – 1.97<br>○ Chronic kidney disease 2.29, 1.30 – 4.01<br>○ Obesity 4.05, 1.71 – 9.61<br>○ Chronic liver disease 2.88, 0.60 – 13.75<br>○ Cancer 1.36, 0.71 – 2.58		○ Hypertension 1.26, 0.68 – 2.29<br>○ Cardiovascular disease 0.66, 0.27 – 1.58<br>○ Chronic lung disease 1.33, 0.69 – 2.54<br>○ Diabetes 0.58, 0.31 – 1.09<br>○ Chronic liver disease 0.89, 0.09 – 8.38<br>○ Chronic kidney disease 0.11, 0.01 – 0.83<br>○ Obesity 2.77, 1.18 – 6.49<br>○ Cancer 0.39, 0.08 – 1.79
Peiro et al., 2021 (Spain)	196	Median 67.5	R	○ Male 1.14, 0.54 – 2.37<br>○ Smoking 2.43, 1.11 – 5.33<br>○ Hypertension 2.81, 1.33 – 5.93<br>○ Diabetes 2.07, 0.95 – 4.49<br>○ Cerebrovascular disease 2.95, 0.91 – 9.60<br>○ Chronic kidney disease 8.07, 3.18 – 20.46		

				○ Chronic lung disease 3.76, 1.66 – 8.56		
Pepe et al., 2021 (Italy)	5746		R	○ Chronic kidney disease 2.47, 1.78 – 3.42		
Pinato et al., 2020 (UK, Italy, Spain)	204	Mean 69	R	○ Age ≥65 vs <65 = 3.07, 1.50 – 6.25<br>○ Smoking 1.64, 0.88 – 3.04		
Pineiro et al., 2021 (Spain)	237	Median 35.5	P	○ Male 1.1, 0.3 – 4.02<br>○ Age >60 vs ≤60 = 4.16, 0.65 – 26.6<br>○ Hypertension 1.85, 0.6 – 5.66<br>○ Diabetes 3.09, 0.72 – 13.32<br>○ Chronic lung disease 3.08, 0.72 – 13.3<br>○ Chronic kidney disease 6.86, 0.76 – 61.67		
Pizzini et al., 2020 (Austria)	109	Median 58	P	○ Male 6.65, 2.78 – 15.90		
Plotnikow et al., 2020 (Argentina)	47	Median 61	P	○ Male 0.55, 0.06 – 5.35<br>○ Hypertension 1.58, 0.27 – 9.31<br>○ Diabetes 1.35, 0.23 – 8.03<br>○ Chronic lung disease 0.81, 0.07 – 9.01		
Pongpirul et al., 2020 (Thailand)	195	Median 37	R	○ Age >60 vs ≤60 = 3.24, 1.24 – 8.48<br>○ Male 2.02, 0.88 – 4.63<br>○ Smoking 0.78, 0.25 – 2.41<br>○ Alcohol 0.58, 0.22 – 1.49<br>○ Diabetes 11.74, 3.88 – 35.50		

				○ Hypertension 5.44, 2.30 – 12.83		
Prado-Galbarro et al., 2020 (Mexico)	15529		R	○ Male (HR) 1.46, 1.03 – 1.04<br>○ Chronic lung disease (HR) 1.28, 1.04 – 1.59<br>○ Cardiovascular disease (HR) 0.85, 0.67 – 1.06<br>○ Hypertension (HR) 1.27, 1.06 – 1.52<br>○ Diabetes (HR) 1.43, 1.18 – 1.74<br>○ Obesity (HR) 1.91, 1.59 – 2.29<br>○ Chronic kidney disease (HR) 1.60, 1.29 – 2.00		
Qian et al., 2020 (China)	82858	Meidan 51	R		○ Male 1.25, 1.20 – 1.29	
Qin L et al., 2020 (china)	548		R	○ Male 2.44, 1.50 – 3.95		
Qin W et al., 2021a (china)	262	Mean 63.5	R	○ Male 0.86, 0.36 – 2.03<br>○ Hypertension 2.13, 0.90 – 5.03<br>○ Diabetes 1.08, 0.35 – 3.35<br>○ Stroke 4.46, 0.81 – 24.39		
Qin W et al., 2021b (China)	749	Mean 60	R	○ Male 3.15, 1.88 – 5.27<br>○ Hypertension 2.03, 1.26 – 3.27<br>○ Diabetes 1.49, 0.77 – 2.89<br>○ Chronic lung disease 2.52, 1.20 – 5.29<br>○ Chronic kidney disease 5.33, 1.25 – 22.74		

Quisi et al., 2020 (Turkey))	349	Median 56	R	○ Smoking 4.28, 2.14 – 8.58<br>○ Chronic lung disease 5.05, 2.08 – 12.25<br>○ Male 0.72, 0.36 – 1.45		
Raimondi et al., 2021 (Italy)	431	Mean 67.6	R	○ Male 1.05, 0.47 – 2.37<br>○ Smoking 2.17, 1.31 – 3.58<br>○ Hypertension 3.09, 2.00 – 4.78<br>○ Chronic kidney disease 2.56, 1.22 – 5.36<br>○ Chronic lung disease 1.98, 1.04 – 3.97		
Ramaswamy et al., 2020 (India)	230	Median 42	P	○ Male 1.12, 0.47 – 2.68<br>○ Age >60 vs ≤60 = 1.90, 0.66 – 5.65<br>○ Smoking 1.12, 1.07 – 1.18<br>○ Diabetes 2.02, 0.69 – 5.91<br>○ Hypertension 1.26, 0.35 – 4.59		
Ramos-Rincon et al., 2020 (Spain)	2772	Median 86.3	R	○ Male 1.27, 1.01 – 1.59<br>○ Obesity 1.24, 1.00 – 1.54<br>○ Hypertension 0.96, 0.81 – 1.14<br>○ Dibetes 1.09, 0.92 – 1.30<br>○ Cancer 1.21, 0.97 – 1.51		
Rastad et al., 2021 (Iran)	520		R	○ Age ≥65 vs <65 = 1.62, 0.60 – 4.41<br>○ Male 2.94, 0.95 – 9.15<br>○ Hypertension 1.12, 0.43 – 2.89		

				o Diabetes 1.89, 0.73 – 4.90		
				o		
Recinella et al., 2020 (Bologna)	76–91) Median 83 years	109	P	o Age continuous<br>o nonsurvivors mean 85.5 [79–86.7]<br>o survivors mean 79<br>o Male 1.11 (.52-2.4)<br>o Charleston Comorbidity Index nonsurvivors mean 4.5, 2.2-8.7<br>o Cognitive Impairment 3.62, 1.6-8.1<br>o Coronary Heart Disease 1.03, .34-3.13<br>o Atrial Hypertension ∞<br>o Diabetes .57, .22-1.5<br>o Previous stroke 2.55, .89-7.34<br>o Chronic Kidney Disease .78, .3-2.03<br>o Chronic Heart Failure 1.45, .51-4.1		
Ren et al., 2020 (Wuhan, China)	34-61 Median 50.0	129	R	o Age ≥ 65 vs <65 20.96, 3.69-119.16	o Age 65 vs <65 5.81, 2.14-15.78<br>o Male .54, .25-1.15<br>o Hypertension 3.29, 1.42-7.63<br>o Diabetes 1.89, .65- 5.48<br>o Cardiovascular disease 4.63 .41-52.63	
Rieg et al., 2020 (Germany)	54-79 Median 65 years	213	R	o Age continuous<br>o Nonsurvivors median 70<br>o Survivors median 61		o Age continuous<br>o Admission in ICU median 65<br>o Non ICU admitted median 65<br>o Male 2.48, 1.32-4.64

Rivera-Izquierdo et al., 2020 (Spain)	24-97<br>Mean 64.7<br>Median 67	238	R	○ Age ≥60 vs<60 6.53, 1.88-22.7<br>○ Male 1.49, .82-2.71<br>○ Smoking .57, .16-2.07<br>○ Hypertension 5.25, 2.69-10.26<br>○ Diabetes 4.83, 2.5-9.3<br>○ Cardiovascular Disease 12.72, 6.28-25.76		
Rivinius et all., 2020 (Germany)	Mean 58.6	21	CS		○ Age >60 vs ≤60 15.75, 1.42-174.25	
Roberts et al., 2020 (Massachusetts, USA)	Median 58 IQR 50-66	52	R			○ Age continuous<br>○ Admission in ICU median 61<br>○ Non ICU admitted median 50<br>○ Male 2.33, .62- 8.72
Gostner et al., 2020 (Sweden)	Mean 51.3	34	CS		○ Age continuous<br>○ Severe mean 61.3<br>○ Mild mean 51.3<br>○ Any comorbidity 8.94, 1.56-51.18<br>○ Diabetes 6.46, .68-61.16<br>○ Coronary Heart Disease 2.63, .24-28.2	○
Rodilla et al., 2021 (Spain)	Median 67.5	12170	P	○ Age<br>○ <60.9 1<br>○ ≥60.9 and <76.3 2.0, 1.56-2.44<br>○ ≥76.3 4.7, 3.77-5.97<br>○ Male 1.6, 1.43-1.75<br>○ Charlson Comorbidity Index<br>○ <2 1<br>○ ≥2 and <5. 4.8, 3.82-6.02<br>○ ≥5 8.6, 6.72-10.9	○	○

Rodriguez et al., 2020<br>(Spain)	Median 65.5	43	P	o Age continuous<br>o Survivors median 60.5<br>o Nonsurvivors median 67.5<br>o Male .56, .13-2.39<br>o Chronic Renal Failure 3, .17-53.05<br>o Obesity 2.44, .52-11.57<br>o Diabetes 1.5, .23-9.8<br>o Arterial Hypertension 3, .67-13.53<br>o Autoimmune Disease 6.75, .54-84.46<br>o Ischemic Heart Disease 3.25, .39-26.92		
Diaz et al., 2020<br>(United States)	n/a	3144	R	o Latino RR 1.05, 1.0-1.1<br>o Black RR 1.2, 1.08-1.3<br>o White RR .853, 1.08-1.04<br>o Unemployed RR .88, .80-.97<br>o Diabetes RR 1.0, .92-1.09<br>o Heart Disease RR 1.02, .92-1.1<br>o Hypertension RR 1.04, .99-1.0<br>o HIV RR .99, .92-1.05		
Molinero et al., 2020<br>(Spain)	Mean 65.4	418	CS	o Age continuous 1.08, 1.05-1.12<br>o Male .99. .61-1.64	o Age continuous 1.01, .10-1.02<br>o Male 1.73, 1.17-2.57<br>o Chronic Kidney Disease 1.83, 1.04- 3.22<br>o Hypertension 1.48, 1.0-2.18	

				- Chronic Kidney Disease 4.64, 2.57-8.34 - Hypertension 5.64, 3.13-1087 - Diabetes 2.96, 1.75-4.99 - Obesity 1.12, .58-2.06 - Smoking .38, .09-.228 - Alcoholism .48, .02-.84 - Stroke 3.63, 1.48-8.67 - Asthma .42, .06-1.49 - Ischemic Heart Disease 1.02, .39-2.30 - Autoimmune Disease 3.7, 1.35-9.85 - Dementia 7.28, 3.74-14.40 - Lung Cancer 4.36, .45-42. 37 - Other Cancer 1.41, .49-3.49 - HIV 2.29, .07-28.64 - Anemia 2.71, 1.23-5.75	- Diabetes 1.70, 1.07-2.74 - Obesity 2.06, 1.22-3.58 - Smoking 1.03, .52-2.10 - Alcoholism 7.59, 1.42-188.85 - Stroke 2.4, .97-6.88 - Asthma .89, .38-2.13 - Ischemic Heart Disease .86, .43-1.71 - Autoimmune Disease 2.92, 1.02-10.77 - Dementia 1.05, .55-2.0 - Other Cancer 1.05, .48-2.4 - HIV 1.5, .71-3.2	
Roedl et al., 2020 (Germany)	Median 69	223	R	- Age continuous - Nonsurvivors median 72 - Survivors median 65 - Male 1.78, .87-3.65 - BMI continuous - Nonsurvivors median 26.2 - Survivors median 26.9		

Rokni et al., 2020<br>(Iran)	Mean<br>49.8	233	CS	o Age continuous<br>o Nonsurvivors mean 65.3<br>o Survivors mean 47.6<br>o Male 1.47, .62-3.51<br>o Diabetes 1.47, .55-3.92<br>o Hypertension 1.94, .76-4.97<br>o Cardiovascular Disease 1, .28-3.58<br>o Pulmonary Disease 3.5, 1.13-10.82<br>o Renal Disease 2.96, .74-11.87<br>o Autoimmune Disease 7.81, 1.05-57.81		
Botero et al., 2020	Mean<br>52.6<br>Sd 17	157	R	o Age ≤ 55 1.08<br>o Age >55 1.014<br>o Male .837, p-value=.473<br>o BMI 1.034, p-value= .59		
Rosenthal et al., 2020<br>(United States)	(41-71)<br>Median<br>57<br>IQR<br>(41-71)	64,781	R	o Age ≥65 vs < 65 8.07, 7.62-8.55<br>o Male 1.18, 1.1-1.26<br>o Black vs White .75, .69-.82<br>o Congestive Heart Failure 1.37, 1.26-1.49<br>o Cerebrovascular Disease 1.39, 1.26-1.56<br>o Chronic Pulmonary Disease 1.16, 1.08-1.26		

				- Dementia 1.21, 1.11-1.32 - Diabetes 1.20, 1.12-1.28 - Malignancy 1.27, 1.09-1.47 - AIDS .68, .44-1.04 - Hypertension 1.08, .99-1.18 - Hyperlipidemia 1.11, 1.03- 1.19		
Rossi et al., 2020<br>(Italy)	(33.8-98.5) Median 76.2 IQR (68.2-82.6)	590	R	- Age continuous - Median nonsurvivors 72.9 - Median survivors 72.9 - Male 1.56, 1.09-2.22 - Cardiovascular Disease 1.21, .78-1.88 - Hyperlipidemia 1.1, .78-1.54 - Diabetes 1.34, .91-1.96 - Chronic Kidney Disease 2.76, 1.73-4.41 - Stroke 2.18, .89-5.34 - Malignancy 1.4, .77-2.54		

Rottoli et al., 2020<br>(Italy)	Mean 66.2	482	R	o Age <60 vs 60-69.9 2.55, 0.67–9.67<br>o Age <60 vs 70-79.9 13.7, 4.35–43.5<br>o Age <60 vs ≥ 80 35.0, 10.8–113<br>o Male 2.36, 1.26–4.43<br>o Hypertension 1.09, 0.56–2.15<br>o Diabetes 1.0, 0.48–2.05<br>o Cerebrovascular Disease 3.41, 1.61–7.26<br>o Moderate/Severe Renal Disease 2.35, 0.90–6.17<br>o Obesity 2.35, 1.17–4.75<br>o BMI continuous 1.09, 1.02–1.17		o Male 1.54, 0.81–2.91<br>o Hypertension 1.61, 0.80–3.25<br>o Diabetes 1.55, 0.73–3.31<br>o Cerebrovascular Disease 0.52 0.13–2.05<br>o Moderate/severe Renal Disease 4.80, 1.83–12.6<br>o Obesity 5.28, 2.81–9.91<br>o BMI continuous 1.20, 1.12–1.28
Russo et al., 2020<br>(Italy)	Mean 66.9 Sd 15	414	R	o Age continuous<br>o nonsurvivors mean 71.75<br>o survivors mean 65.26<br>o Male 1.8, 1.12-2.9<br>o Smoker 1.02, .59-1.76<br>o Hypertension 1.89, 1.16-3.07<br>o Diabetes 1.34, .82-2.19<br>o Obesity 1.32, .58-2.99<br>o Stroke .99, .45-2.19<br>o Chronic Kidney Disease 2.46, 1.41-4.29		
Ruthrich et al., 2021<br>(Europe)	IQR 76–85	3071	R	o Male 1.73, .55-5.36<br>o Cancer .56, .44-.72		

Rutten et al., 2020<br>(Netherlands)	Mean 84<br>Sd 9.8	1538	P	Hazard Ratio (HR)<br>o Male 1.82, 1.54-2.15<br>o Dementia 1.26, 1.06-1.5<br>o Cardiovascular Disease 1.15, .97-1.35<br>o Cerebrovascular Disease 1.06, .89-1.25<br>o Diabetes .99, .82-1.19<br>o Chronic Respiratory Disease .92, .75-1.14<br>o Parkinson's 1.49, 1.11-2.0		
Saand et al., 2020<br>(USA)	Median 68<br>IQR 58-77	495	R	o Hazard Ratio (HR)<br>o Age ≥ 60 vs <60 3.2, 1.79-5.79<br>o Male .73, .50-1.05<br>o BMI ≥30 vs <30 .79, .54-1.14<br>o Diabetes .82, .5-1.27		
Sadeghi et al., 2020<br>(Iran)	Mean 61.78<br>Sd 16.4	214	R		o Age mild mean 54.4<br>o Age severe mean 70.3<br>o Male mild vs severe .98, .27-2.5	
Saeed et al., 2020<br>(NY, United States)	Mean 68<br>Sd 12	2266	R	o Age survivors mean 67<br>o Age nonsurvivors mean 74<br>o Male 1.34, 1.12-1.59<br>o Nonhispanic Black vs Nonhispanic White .78, .55-1.1<br>o Hispanic vs Nonhispanic White .81, .57-1.14<br>o Other vs White .89, .61-1.3<br>o BMI mean survivors 28.9		

				○ BMI mean nonsurvivors 28.2<br>○ Hypertension .89, .68-1.16<br>○ Charlson Comorbidity Index survivors 4 (2-8) Nonsurvivors 7 (4-10)<br>○ Lung Disease 1.12, .93- 1.34		
Saez-Giménez et al., 2020 (Spain)	Median 62.8 IQR 56.4-67.8	44	R	○ Age nonsurvivors median 62.9<br>○ Male 2.23, .61-8.08		
Saggi et al., 2020 (United States)	N/A	75	R	○ Age 30-70 vs 71-76. 1.12, .45-2.70<br>○ Age 30-70 vs 77-99. 2.17, .75-6.25		
Saha et al., 2021 (Bangladesh)	Median 48	168	R	○ Diabetes 1.37, .74-2.53		
Sai et al., 2021 (Wuhan, China)	(38-93) Mean 70.6 Sd 12.5	47	R	○ Age survivors mean 69.67<br>○ Age nonsurvivors mean 70.64<br>○ Male .34, .1-1.2<br>○ Comorbidity 3.23, .35-29.6<br>○ Hypertension .68, .2-2.33<br>○ Diabetes 1.11, .32- 3.9<br>○ Chronic Kidney Disease .09, .01-.78<br>○ Chronic Heart Disease 1.4, .29-6.82		

				○ Chronic Liver Disease .41, .07-2.35<br>○ Malignancy 1.49, .22-9.99		
Saifi et al., 2021<br>(Italy)	(90-101) Median 93	34	R	○ Age survivors median 93<br>nonsurvivors median 93<br>○ Male 1, .26-3.85<br>○ BMI survivors median 25.2<br>BMI nonsurvivors median 23.2<br>○ Hypertension .87, .15-5.06<br>○ Chronic Kidney Disease .45, .11-1.85<br>○ Chronic Heart Failure .45, .11-1.85<br>○ Arrhythmias 1.18, .27-5.18<br>○ Dementia 1.18, .27-5.18<br>○ Diabetes 1.85, .27-12.76<br>○ Cerebrovascular Disease 1.85, .27-12.76<br>○ Charlson Comorbidity Index survivors 5 (4.7-6)<br>Nonsurvivors 5.5 (4-6.7)		
Salazer et al., 2021<br>(Argentina)	Mean 62<br>Sd 17	3529	R	○ Age mean survivors 58<br>Age mean nonsurvivors 69<br>○ Male .97, .84-1.1		

				○ Hypertension 1.69, 1.46-1.95<br>○ Diabetes 1.32, 1.12-1.56<br>○ Cardiovascular Disease 2.09, 1.68-2.6<br>○ Chronic Pulmonary Disease 1.26, .98-1.63<br>○ Immunodeficiency 1.35, .86-2.12		
Saleh et al., 2021 (Egypt)	(0-18)	398	P		○ Age continuous 1.07, .58-1.99<br>○ Male 1.14, .68-2.3	
Sands et al., 2020 (United States)	Median 63	6180	R	○ Age continouous 1.07, 1.06-1.08<br>○ Diabetes 1.57, 1.21-2.03<br>○ Hypertension .63, .48-.82<br>○ Obesity 1.41, 1.08-1.85<br>○ Renal Failure .96, .71-1.29<br>○ Congestive Heart Failure 1.19, .9-1.58		
Sano et al., 2020 (Japan)	(76-98) Mean 87.8	26	R	○ Age ≥ 75 vs <75 1.65, .23- 11.99		
Santos et al., 2020 (Spain)	Mean 75.3 IQR 68-83	38	P	○ Age >65 vs ≤ 65 1.08, .1-11.76<br>○ Male 3.6, .77-7.0<br>○ Hypertension 9, 1-80.8<br>○ Diabetes 33, 3.46-314.55<br>○ Dyslipidaemia 12, 1.33-108.03		

M. Santos et al., 2020 (Brazil)	N/a	46285	R	Hazard Ratio (HR)<br>○ Diabetes 1.17, 1.11-1.24<br>○ Chronic Kidney Disease 1.11, 1.43-1.58<br>○ Asthma .71, .61-.81<br>○ Cardiopathy 1.14, 1.08-1.2<br>○ Race: Black or mixed vs White or yellow 1.5, 1.43- 1.58		
Santus et al., 2020 (Italy)	Median 66 IQR (55-76)	412	P	(HR)<br>○ Age >65 vs ≤ 65 3.41, 2.0-5.78<br>○ Male 1.17, .73-1.86	○ Age continuous Severe median 75 Mild median 67<br><br>Odds Ratio (severe vs mild)<br>○ Male 2.22, 1.05-4.72	
Sapey et al., 2020 (United Kingdom)	Median 73 IQR (58-84)	2217	R	(HR)<br>○ Age continuous 2.4, 1.8-3.2<br>○ Male 1.3, 1.1-1.5<br>○ Comorbidity (≥3) 2.3, 1.7-3.0<br>○ Asian vs White. 1.4, 1.2- 1.8<br>○ Black vs White 1.1, .8- 1.5<br>○ Mixed vs White 1.9, .9-3.9		
Scarfo et al., 2020 (Europe)	(58-84) Median 72	121	R		○ Age ≥65 vs <65 3.72, 1.79-7.71<br>○ Male .73, .34-1.57<br>○ Comorbidities >2 vs ≤2 .99, .46-2.11	
Schultze et al., 2020 (England)	(18-106) Median 53	33,356,521	R	(HR)<br>○ Asthma 1.55, 1.13-2.21		

Wang F et al., 2021<br>(China)	Median<br>46<br>IQR<br>32-57	625	R		○ Age continuous 1.07, 1.03-1.10<br>○ Hypertension .80, .30-2.13<br>○ Diabetes 2.13, .59-7.68<br>○ Coronary Heart Disease 4.6, .78-27.20	
Wang Y et al., 2020<br>(Wuhan, China)	Median<br>68<br>IQR<br>61-75	242	R	○ Age continuous Survivor median 66 Nonsurvivor median 69<br>○ Male 1.71, .96-3.04<br>○ Cardiovascular Disease 1.14, .48-2.71<br>○ Chronic Kidney Disease .49, .11-2.24<br>○ Cerebrovascular Disease 2.77, .61-12.51		
Yao et al., 2021<br>(Wuhan, China)	Mean<br>62.7<br>Sd<br>11.09	97	R	○ Age continuous Survivor mean 60.8 Nonsurvivor mean 69.8<br>○ BMI continuous Survivor mean 23.09 Nonsurvivor mean 24.95<br>○ Smoker 1.22, .12-12.34<br>○ Diabetes 2.98, 1.03-8.64<br>○ Hypertension 2, .75-5.3<br>○ Cardiovascular Disease 2.67, .89-7.99<br>○ Lung Disease .69, .14-3.45		

Zhou et al., 2020<br>(Wuhan, China)	n/a	110	R		o Age 40-60 vs ≤40 12.28, 1.62-92.6<br>o Age ≥ 60 vs ≤40 25.3, 3.68-173.783	
Ye et al., 2020<br>(Wuhan, China)	Median 59.2<br>IQR 42.8-73.1	293	R	o Age ≥ 65 vs <65. 9.95, 5.76-17.19<br>o Male 1.82, 1.13-2.91<br><br>(HR)<br>o Hypertension 2.88, 2-4.17<br>o Diabetes 1.83, 1.12-2.84<br>o Cerebrovascular Disease 2.41, 1.47-3.94<br>o Coronary Heart Disease 1.77, 1.01-3.1<br>o Chronic Renal Disease 2.16, .88-5.28<br>o Chronic Liver Disease 1.06, .43-2.58<br>o Malignancy 1.4, .6-3.18		
Wang Z et al, 2020<br>(NY, United States)	n/a	3273	R	o Age continuous 1.08, p<.0001<br>o Male 1.06, p=.698<br>o Race<br>African American .95, p=.815<br>Asian 1.04, p=.914<br>Hispanic .93, p=.723<br>Other .99, p= .975<br>o Smoker 1.71, p=.172<br>o BMI 1, p=.791		
Wargny et al., 2021<br>(France)	Mean 69.7<br>Sd 13.2	2796	R	o Age continuous 1.84, 1.49-2.27<br>o Male 1.01, .7-1.45		

				○ BMI continuous 1.03, .86-1.25<br>○ Hypertension .84, .51-1.39		
Chen et al., 2020<br>(USA, UK, Italy, Middle East)	Median 60<br>IQR 47-66	151	R	○ Age continuous 1.06, 1.01-1.11<br>○ Male .79, .24-2.62<br>○ Smoker .95, .03-29.82<br>○ White vs Other Race 1.56, .45-5.42<br>○ Obesity 1.10, .37-3.31<br>○ Cardiovascular Disease .65, .15-2.71<br>○ Diabetes 1.83, .57-5.87<br>○ Hypertension 1.03, .34-3.09		
Wei et al.,2020a<br>(Wuhan, China)	Median 61.5	566	R	○ Age continuous 1.09, 1.02-1.16<br>○ Cardiovascular Disease 3.29, 1.66-6.54<br>○ Heart Failure 4.78, 1.54-14.82<br>○ Arrhythmia 1.38, .72-2.65		
Wei X et al., 2020b<br>(Wuhan, China)	Median 66<br>IQR 59-72	597	R		○ Age continuous<br>○ Mild median 64<br>○ Severe median 69<br>○ Male 1.6 1.12- 2.3<br>○ Hypertension 2.37, 1.62-3.46	
Wei Z et al, 2021<br>(China)	Median 49<br>IQR 37-59	498	R	○	○ Age <65 vs ≥ 65 1.68, .55-5.07<br>○ Male .86, .31-2.21<br>○ Diabetes 2.85, 1.35-6.01<br>○ Coronary Heart Disease 1.08, .25-4.62	

					○ Chronic Kidney Disease 3.74, .67-20.88	
Wen et al., 2021 (Hubei, China)	Mean 53 Sd 15.7	476	R	(HR)<br>○ Age continuous 1.09, 1.05-1.14<br>○ Male 1.38, .56-3.4<br>○ Hypertension 5.82, 2.60-13.07<br>○ Diabetes .82, .32-2.11		
Williams et al., 2021 (China, Italy, Spain, Netherlands	n/a	876,959	R	○ Male 1.77, 1.73-1.8		
Betech et al., 2020 (Brazil)	(0-100)	113,214	R	○ Age <65 vs ≥ 65 1.28, .84-1.94<br>○ Male 1.24, 1.2-1.29<br>○ Diabetes 1.22, 1.17-1.27<br>○ Obesity 1.67, 1.52-1.82<br>○ Asthma .80, .71-.89<br>○ Cardiovascular Disease 1.12, 1.08-1.16<br>○ Hematologic Disease 1.19, 1.0-1.42<br>○ Chronic Liver Disease 1.72, 1.44-2.04<br>○ Chronic Renal Disease 2.0, 1.84-2.17<br>○ Immunosuppresson 1.67, 1.52-1.82<br>○ Neurological Disease 1.64, 1.51-1.79<br>○ Geographic Location<br>○ N Brazil 2.06, 1.35-3.14<br>○ S Brazil .62, .40-.95		

				○ NE Brazil 2.19, 1.4-3.3<br>○ SE Brazil 1.03, .68-1.57<br>○ Midwest Brazil .84, .55-1.29		
Wu et al., 2020<br>(Wuhan, China)	(21-83)<br>Median 51<br>IQR 43-60	201	R	(HR)<br>○ Age <65 vs ≥ 65 6.17, 3.26-11.67<br>○ Male .56, .30-1.05<br>○ Hypertension 1.82, 1.13-2.95<br>○ Diabetes 2.34, 1.35-1.05	(HR)<br>○ Age <65 vs ≥ 65 3.26, 2.08-5.11<br>○ Male 1.47, .92-2.36<br>○ Hypertension 1.82, 1.13-2.95<br>○ Diabetes 2.34, 1.35-4.05	
Wu, F et al., 2020<br>(China)	>40	1048	R	○ Chronic Obstructive Pulmonary Disease 7.56, 3.67-15.57	○ Chronic Obstructive Pulmonary Disease 2.69, 1.37-5.29	○ Chronic Obstructive Pulmonary Disease 2.78, 1.35-5.73
Wu, M et al., 2020<br>(China)	Mean 57<br>IQR 45-67	334	R		○ Age <50 vs 51-63 1.8, .2-20.9	○ Age <50 vs 51-63 .7 (.1-4.8)<br>Age <50 vs >64 .1, 0-2
Xie et al., 2020<br>(Wuhan, China)	>18<br>Median 65<br>IQR 56-73	733	R	(HR)<br>○ Age continuous 1.015, 1.02-1.03<br>○ Male 1.23, 1.03-1.59<br>○ Hypertension 1.38, 1.13-1.68<br>○ Malignancy 1.91, 1.17-3.12<br>○ Smoking 1.55, 1.08-2.23		
Xie,Y et al., 2020<br>(Wuhan, China)	Median 66<br>IQR 53-73	62	R		○ Cardiovascular Disease 3.34, 1.12-9.94<br>○ Male 2.03, .72-5.73<br>○ Diabetes 8.33, 1.99-34.87<br>○ Hypertension 5.37 1.76-16.37	
Xiong et al., 2020	(31-92)	131	R		○ Age continuous<br>○ Mild mean 63.1	

(Wuhan, China)	Mean 63.2 Sd 13.1				o Severe mean 64.3<br>o BMI continuous<br>o Mild mean 23.6<br>o Severe mean 22.6<br>o Male .61, .21-1.76<br>o Smoker .66, .26-1.69<br>o Cardiovascular Disease 2.77, .98-7.86<br>o Diabetes .61, .21-1.76<br>o Chronic Obstructive Pulmonary Disease .84, .09-7.78<br>o Cancer 3.45, .21-56.85	
Xiong, S et al., 2020 (Wuhan, China)	Median 58.5 IQR 47-69	116	R		o Age <65 vs ≥ 65 3.48, 1.57-7.73<br>o Male 1.01, .46-2.22<br>o Hypertension 1.98, .93-4.23<br>o Diabetes 1.66, .61-4.48<br>o Coronary Heart Disease 4.41, 1.34-14.49<br>o Chronic Liver Disease 1.11, .07-18.2	
Xu B. et al., 2020 (Wuhan, China)	Median 62 IQR 48.5-71	187	R	o Age continuous<br>o Nonsurvivors median 73<br>o Male 1.31, .58-2.98<br>o Chronic Kidney Disease 2.98, .52-17.11<br>o Metabolic Disease 1.39, .43-4.49	o Age continuous<br>o Mild median 56<br>o Severe median 60<br>o Male 1.55, .64-3.73<br>o Metabolic Disease 1.6, .5-5.11	o Age continuous<br>o ICU median 70<br>o Male 2.2, 1.16-4.17<br>o Metabolic Disease 1.11, .44-2.77
Xu Jingyuan. et al., 2020 (Wuhan, China)	Median 65 IQR 56-73	671	R	o Age continuous<br>o Survivors median 61<br>o Nonsurvivors median 68<br>o Male 2.37, 1.36-4.12		

				○ Hypertension 2.20, 1.16-3.54<br>○ Coronary Heart Disease .35, .18-.68<br>○ Tumor .38, .09-1.55<br>○ Stroke 1.32, .35-4.95<br>○ Diabetes 1.05, .55-1.98<br><br>(HR)<br>○ Acute Kidney Injury 1.62, 1.15-2.3		
Xu Jiqian et al., 2020 (Wuhan, China)	Mean 62.5<br>Sd 13.3	239	R	○ Age <65 vs ≥ 65 2.61, 1.51-4.49<br>○ Male 1.16, .68-1.97<br>○ Hypertension .96, .57-1.67<br>○ Diabetes .8, .41-1.58<br>○ Malignancy 3.64, .79-16.81<br>○ Chronic Cardiac Disease .93, .45-1.93<br>○ Chronic Pulmonary Disease 1.93, .51-7.34<br>○ Cerebrovascular Disease 1.43, .43-4.8<br>○ Chronic Liver Disease 1.18, .45-3.07		○ Age continuous<br>○ ICU mean 62.5 sd 13.3
Xu Peng et al., 2020 (China)	(2 months-86 yrs)<br><br>Mean 46.1<br>Sd 15.2	703	R	○ Male 2.37, 1.08-5.17<br>○ Cardiovascular Disease 18.26, 7.9-42.18<br>○ Diabetes 7.11, 3.3-15.31<br>○ Hypertension 6.4, 3.12-13.11<br>○ Chronic Liver Disease .73, .1-5.55		

				o Chronic Kidney Disease 10.88, 2.6-45.63<br>o Malignancy 2.54, .31-20.95<br><br>(HR)<br>o Age ≤ 60 vs >60 ≤ 60 vs >60 2.23, 1.12-4.43		
Yan Chengxi et al., 2021 (China)	Mean 57.03<br>Sd 17.2	310	R			o Age ≤ 60 vs >60 12.72, 2.42-24.61<br>o Cardiovascular Disease 6.23, 2.88-13.48<br>o Male .57, .28-1.13<br>o Chronic Obstructive Pulmonary Disease 12.79, 2.38-68.56<br>o Diabetes 15.79, 3.06-81.57
Yan Q. et al., 2020 (Wuhan, China)	>65<br><br>Median 71<br>IQR 68-77	882	R	(HR)<br>o Age continuous 1.01, .98-1.05<br>o Male 1.36, .83-2.24<br>o Cardiovascular Disease 1.51, .91-2.51<br>o Chronic Kidney Disease 1.09, .60-1.97<br>o Cerebrovascular Disease .70, .28-1.8<br>o Diabetes 1.17, .73-1.86<br>o Acute Kidney Injury 4.78, 2.97-7.70<br>o Malignancy 1.45, 1.01-2.44		
Yang C. et all., 2021 (Wuhan, China)	Median 62<br>IQR 49-69	203	R	o Age continuous 1.03, .99-1.06<br>o Male 1.68, .89-3.15		

				o Smoking History 1.89, .74-4.87<br>o Chronic Obstructive Pulmonary Disease 5.5, .98-30.92<br>o Hypertension 3.8, 2.09-6.93<br>o Diabetes 1.65, .73-3.75<br>o Cardiovascular Disease 3.33, .86-12.86<br>o Cerebrovascular Disease 1.15, .38-3.47		
Yang D. et al., 2020 (Wuhan, China)	(20-99)	836	R	o Age <65 vs ≥ 65 3.26, 1.79-5.94<br>o Male 2.77, 1.54-5.0		
Yang G. et al., 2020 (China)	Median 66 IQR 60-73	462	R	o Hypertension 1.68, .67-4.21	o Hypertension 1.44, .76-2.73	o Hypertension 1.77, .86-3.62
Yang K. et al., 2020 (Hubei, China)	(14-96)<br><br>Median 63 IQR 56-70	205	R	o Age ≤ 60 vs >60 .97, .48-1.96<br>o Male 3.86, 1.80-8.25<br>o Hypertension .74, .34-1.59<br>o Diabetes .41, .34-1.59<br>o Coronary Heart Disease 2, .65-6.12		
Yang Q. et al., 2020 (Wuhan, China)	(24-85)<br><br>Median 56 IQR 44-64	136	R		o Age continuous<br>o Mild median 53.5<br>o Severe median 64<br>o Male 1.38, .63-3.02<br>o Hypertension 4.7, 2.02-10.94<br>o Cardiovascular Disease 7.41, 1.74-31.57<br>o Diabetes 4.04, 1.51-10.86<br>o Malignancy 1.04, .1-.17	

					○ Chronic Kidney Disease .05, .01-.17<br>○ Chronic Liver Disease 1.04, .2-5.43	
Yang X. et al., 2020<br>(Wuhan, China)	Median 57<br>IQR 47-67			○ Age continuous<br>○ Median survivors 56<br>○ Median nonsurvivors 67<br>○ Male 1.9, 1.42- 2.53<br><br>(Rate Ratio)<br>○ Age 1.03, 1.02-1.04		
Yarza et al., 2020	Mean 66<br>IQR 63-68	63	P	○ Age >65 vs ≤ 65 1.59, .5-4.99<br>○ Male 1.17, .37-3.57<br>○ Current Smoker 2.48, .5-12.54<br>○ Hypertension 2, .68-5.88<br>○ Diabetes 1.18, .34-3.9<br>○ Chronic Kidney Disease 2.39, .29-19.85<br>○ Chronic Obstructive Pulmonary Disease .35, .05-2.23<br>○ Lung Cancer, 2.35, .58-9.51		
Yayla et al., 2020<br>(Turkey)	(0-17)<br><br>Median 8<br>IQR 2-13	77	R	○	○ Age continuous<br>○ Mild median 6.5<br>○ Severe median 1<br>○ Male 1.15, .1-13.25	
Yehia et al., 2020<br>(United States)	Median 61<br>IQR 46-74)	11210	R	(HR)<br>○ Age 18-49 1 (reference)<br>○ 50-64. 1.36, 1.11-1.67	○	

				○ 65-84, 2.38, 1.73-3.26<br>○ ≥ 85 3.96, 2.82-5.55<br>○ Male 1.23, 1.11-1.36<br>○ Asthma .91, .74-1.12<br>○ Cancer 1.15, .91-1.47<br>○ Chronic Kidney Disease 1.15, 1.03-1.30<br>○ Chronic Obstructive Pulmonary Disease 1.22, .94-1.57<br>○ Congestive Heart Failure 1.05, .91-1.22<br>○ Diabetes 1.08, .85-1.36<br>○ Obesity .97, .81-1.16		
Yu Caizheng et al., (2020)<br>(Wuhan, China)	Median 64<br>IQR 51-71	1663	R	○ Age <65 vs ≥65. 2.11, 1.39-3.21<br>○ Male 2.02 1.37-2.99<br>○ Diabetes 2.52, 1.62-3.94		
Yu Y. et al., 2020<br>(Wuhan, China)	(8-97)<br><br>Median 58<br>IQR 46-67	3265	R		○ Age continuous 1.03, 1.02-1.05<br>○ Diabetes 1.95, .71-5.36<br>○ Coronary Heart Disease .91, .24-3.37<br>○ Hypertension .7, .13-3.86	
Yue et al., 2021<br>(Hubei, China)	(18-90)<br><br>Mean 47<br>Sd 15	180	R			○ Age continuous 1.05, 1.02-1.09<br>○ Male 1.08, .43-2.73<br>○ Diabetes 5, 1.51-16.56<br>○ Cardiovascular Disease 8.5, 3.25-22.97<br>○ Chronic Kidney Disease 52.67, 10.05-276.14
Zachariah et al., 2020<br>(NYC, United States)	<21	50	R		○ Age continuous<br>○ Severe median 14y	

					○ Nonsevere median 9y<br>○ Male .63, .15-2.68<br>○ White 1.08, .25-4.61<br>○ Hispanic .76, .18-3.25<br>○ Obesity 14.4, 2.71-76.65<br>○ Asthma 2.64, .4-17.32<br>○ Immunosuppression .61, .07-5.66<br>○ Sickle Cell Disease 1.58, .15-17.25<br>○ Diabetes 11.43, .91-143.64	
Ziener et al., 2020<br>(Michigan, United States)	n/a	49701	R	(RR)<br>○ White 1 (Reference)<br>○ Black 5.5, 5.4-5.6<br>○ Latino 3.1, 3.-3.2<br>○ Asian/Pacific Islander 1.7, 1.6-1.8<br>○ Native American 1, .8-1.2		
Zeng H. et al., 2020<br>(Wuhan, China)	Median 63.5<br>IQR 52-70	642	R	○ Age continuous<br>○ Median survivors 60<br>○ Median nonsurvivors 70<br>○ Age <65 vs ≥65 2.62, 1.57-4.38<br>○ Male 2.08, 1.26-3.44	○	
Zeng Z. et al., 2020<br>(Hunan, China)	Median 45<br>IQR 34.5-57	461	R			(HR)<br>○ Age <65 vs ≥65 4.02, 2.32-6.95<br>○ Male .68, 1.39-1.18<br>○ Hypertension 2.65, 1.52-4.64<br>○ Diabetes 1.55, .73-3.29<br>○ Cardiovascular Disease 2.30, .99-5.38
Zhang Hongmei et al., 2020<br>(Wuhan, China)	(22-89)<br><br>Median 55	88	R		○ Age continouous<br>○ Moderate median 37<br>○ Severe median 58<br>○ Male 1.46, .54-3.96	○ Age continouous<br>○ Critical Median 66<br>○ Male 3.74, 1.31-10.72<br>○ Hypertension 1.34, .47-3.84

					o Hypertension 3.3, .96-11.31<br>o Diabetes 1.68, .26-10.69<br>o Cardiovascular Disease 2.55, .25-25.86<br>o Chronic Kidney Disease .8, .05-13.37<br>o Cancer .8, .05-13.37	o Diabetes 4.24, 1.15-15.59<br>o Cardiovascular Disease 3.21, .73-14.08<br>o Cerebrovascular Disease 13.47, 1.42-127.88<br>o Chronic Kidney Disease 3, .4-22.64<br>Cancer 1.43, .12-16.58
Zhang Hongyan et al., 2020<br>(Wuhan, China)	(36-98)<br><br>Median 66	107	R	(HR)<br>o Age 1.05, 1.01-1.1	o Age continuous<br>o Mild median 64<br>o Severe median 69<br>o Male 2.37, 1.09-5.18<br>o Smoke 1.44, .35-5.89<br>o Cancer 1, .39-2.6<br>o Hypertension 1.66, .74-3.74<br>o Diabetes 4.01, 1.36-11.86<br>o Cardiovascular Disease 2.55, .75-8.73<br>o Autoimmune Disease .91, .12-6.69	
Zhang Jing et al., 2020<br>(Wuhan, China)	Median 60<br>IQR 49-69	901	R		o Age continuous<br>o Moderate median 54<br>o Severe median 64<br>o Male 1.22, .92-1.62<br>o Hypertension 1.97, 1.43-2.71<br>o Diabetes 1.39, .9-2.15<br>o Malignancy 1.46, .6-3.57	o Age continuous<br>o ICU median 68
Zhang Lin et al., 2020<br>(Wuhan, China)	(21-97)<br><br>Median 51.5<br>IQR 34-65.3	222	R	o Age <70 vs ≥70 12.81, 5.7-28.8<br><br>(HR)<br>o Smoke 2.59, .81-8.29<br>o Male .97, .41-2.28<br>o Chronic Liver Disease 1.94, .62-6.05	o Age <70 vs ≥70 16.19, 3.28-79.8<br>o	o Age <70 vs ≥70 3.58, 1.69-7.59

				○ Chronic Kidney Disease .42, .06-3.18<br>○ Chronic Obstructive Pulmonary Disease 2.31, .77-6.9<br>○ Diabetes .76, .22-2.58		
Zhang L. el al., 2020<br>(Wuhan, China)	Median 65<br>IQR 56-70	28	R		(HR)<br>○ Age continuous 1.46, .48-4.43<br>Male .57, .16-2.04	
Zhang Lijuan et al., 2020<br>(Wuhan, China)	Median 56<br>IQR 39-68	651	R	○ Age continuous<br>○ Survivors median 69<br>○ Nonsurvivors median 84<br>○ Male 3.6, .96-13.52<br>○ Stroke 7.79, 4.17-14.56<br>○ Hypertension 2.17, .38-12.46<br>○ Diabetes 1.18, .37-3.73<br>○ Cardiovascular Disease 1.64, .5-5.1<br>○ Chronic Kidney Disease 2.05, .55-7.7<br>○ Chronic Pulmonary Disease 2.68, .67-10.77<br><br>(HR)<br>○ Age 1.08, 1.01-1.15	○ Stroke 1.12, .41-3.09	○ Stroke 9.12, 4.88-17.04
Zhang Nan et al., 2020<br>(Wuhan, China)	Median 65<br>IQR 61-72	52	R		○ Male 1.26, .4-4.03<br>○ BMI continuous<br>○ Severe median 24.34	

					o Non-severe median 24.67<br>o Smoke .66, .19-2.3<br>o Chronic Kidney Disease 3.16, .27-37.27<br><br>(HR)<br>o Age 1.02, .96-1.07	
Xu Xunhua et al., 2020<br>(Wuhan, China)	Median 64.4<br><br>Sd 11.0	60	R	o Age contiuous<br>o Mean Survivors 62.6<br>o Mean Nonsurvivors 70.6<br>o Male .91, .21-4.01		
Zhang Nannan et al., 2021<br>(China)	Median 43.8<br>Sd 15.9	78	P		o Age continuous<br>o Moderate median 43.5 sd 16<br>o Severe median 48 sd 15.5<br>o Male 3, .33-27.06<br>o Hypertension 1.24, .13-11.75<br>o Diabetes 1.24, .13-11.75<br>o Smoke 1.87, .19-18.73	
Zhang Q. et al., 2020<br>(Wuhan, China)	Median 62<br>IQR 58-81	74	R		o Age continuous<br>o Nonsevere median 61<br>o Severe median 72<br>o Male 3.22, 1.19-8.7<br>o BMI continuous<br>o Nonsevere median 24.7<br>o Severe median 24.5<br>o Hypertension 1.69, .65-4.38<br>o Coronary Heart Disease 3.54, 1.02-12.24	
Zhang S. et al.,2020<br>(Wuhan, China)	Median 62<br>IQR 51-69	828	R	o Age >60 vs ≤60 4.63, 2.99-7.18<br><br>(HR)<br>o Male 1.41, .93-2.15<br>o Comorbidity (yes vs no) 1.41, .93-2.15		
Zhang W. et al., 2021<br>(Wuhan, China)	Median 63<br>IQR	461	R		o Diabetes 2.04, 1.28-3.28	

	54-69					
Zhang Y. et al., 2021 (Wuhan, China)	>18<br><br>Mean 60<br>Sd 15	274	R		○ Age 1.03, 1.01-1.05<br>○ Male 2.61, .84-7.45<br>○ Smoke 1.55, .70-3.45<br>○ Chronic Pulmonary Disease 2.16, .68-6.93<br>○ Cardiovascular Disease 1.13, .50-2.52<br>○ Diabetes 1.74, .85-3.58<br>○ Hypertension .86, .5-1.47	
Zhao J. et al., 2021 (Jiangsu, China)	>18	583	R		○ Age <65 vs ≥ 65 2.75, 1.57-4.81<br>○ Male 1.28, .8-2.04<br>○ Smoke .9, .41-1.98<br>○ Hypertension 2.18, 1.31-3.64<br>○ Diabetes 4.26, 2.44-7.48<br>○ Chronic Obstructive Pulmonary Disease 4.45, 1.64-12.03<br>○ Coronary Heart Disease 3.12, 1.14-8.56<br>○ Cardiovascular Disease 6.25, 1.77-22.09<br>○ Cancer 9.52, 2.63-34.49	
Zhao M., et al., 2020 (China)	(21-101)<br><br>Median 61<br>IQR 46-70	1000	R	○ Age <60 vs ≥ 60. 4.11, 2.58-6.56<br><br>(HR)<br>○ Hypertension 1.97, 1.3-3.0<br>○ Diabetes .96, .58-1.61<br>○ Coronary Heart Disease .97, .55-1.73<br>○ Chronic Obstructive Pulmonary Disease 1.48, .63-3.48<br>○ Cerebrovascular Disease 2.1, 1.16-3.81	○ Age <60 vs ≥ 60 2.8, 2.08-3.79	○ Age <60 vs ≥ 60 2.01, 1.17-3.47

				o Chronic Renal Disease 1.37, .63-2.94<br>o Chronic Liver Disease 1.22, .46-3.21<br>o Malignancy .74, .30-1.84		
Zhao M. et al., 2020<br>(Wuhan, China)	Mean 60.3<br>Sd 12.7	413	R			o Age continuous<br>o ICU mean 64.6<br>o Non-ICU mean 59.5<br>o Male 1.21, .71-2.04<br>o Diabetes 1.26, .58-2.74<br>o Hypertension 1.22, .69-2.16<br>o Cardiovascular Disease 1.38, .63-3.02<br>o Pulmonary Disease 2.46, .82-7.31<br>o Cancer 2.33, .98-5.58<br>o Chronic Renal Disease 4.33, 1.13-16.57
Zhao Y. et al., 2020<br>(Wuhan, China)	Median 58<br>IQR 37-65	539	R	o Age continuous .91, .88-.94<br>o Male 1.64, 1.1-2.46<br>o Diabetes 2.76, 1.59-4.9<br>o Chronic Kidney Disease 10.39, 2.07-52.13<br>o Hypertension 4.24, 2.76-6.51<br>o Coronary Heart Disease 4.45, 2.25-8.79<br>o Cerebrovascular Disease 13.98, 4.55-42.96<br>o Malignant Tumor 3.23, 1.39-7.52		

Zheng B. et al., 2021<br>(Beijing, China)	(17-86)	198	R		○ Age continuous<br>○ Mild mean 46<br>○ Severe mean 64.5<br>○ Male .8, .38-1.69<br>○ Hypertension 3.92, 1.45-10.62<br>○ Diabetes 3.25, .87-12.18	
Zheng T. et al., 2020<br>(China)	(18-65)<br><br>Median 50<br>IQR 40-57	1320	R		○ Age <60 vs ≥ 60 1.11, .72-1.70<br>○ Male 2.34, 1.64-3.35	
Zhong Y. et al., 2020<br>(Wuhan, China)	Mean 66.3<br>Sd 10.6	650	R	○ Age >70 vs ≤70. 1.83, .66-5.07<br>○ Male 5.49, 1.78-16.94<br>○ Coronary Artery Disease 2.03, .45-9.08		
Zhou Jun et al., 2021<br>(Jiangsu, China)	Median 48	283	R		○ Age continuous<br>○ Mild median 45<br>○ Severe/Critical median 61<br>○ Male 2.39, .74-7.77<br>○ Diabetes 4.81, 1.03-14.21<br>○ Cerebrovascular Disease 9.85, 2.1-46.7<br>○ Cancer .83, .1-6.73	
Zhou J. et al., 2020<br>(Hubei, China)	(19-92)<br><br>Mean 58.3<br>Sd 15.9	429	R			○ Age <61 vs ≥61. 4.45, 1.68-11.75<br>○ Male 2.99, 1.27-6.99<br>○ Hypertension 2.65, 1.16-6.04
Zhou S. et al., 2020<br>(Hubei, China)	Median 59.5<br>IQR 47-69	220	R	○ Age continuous 1.09, 1.03-1.15<br>○ Male 3.15, 1.62-6.12<br>○ Smoke 3.76, 1.47-9.63<br>○ Hypertension 2.96, 1.56-5.61<br>○ Diabetes 3.18, 1.48-6.84		

				○ Cardiovascular Disease 3.58, 1.52-8.44<br>○ Cerebrovascular Disease 10.83, 2.11-55.44<br>○ Chronic Obstructive Pulmonary Disease 10.22, 1.04-100.51<br>○ Chronic Renal Failure 10.22, 1.04-100.51		
Zhou Wei et al., 2021 (Wuhan, China)	Median 68 IQR 62-74	186	R	○ Age continuous<br>○ Survivor median 67<br>○ Nonsurvivor median 68<br>○ Male 2.19, 1.21-3.95<br>○ Hypertension 2.36, 1.17-4.78<br>○ Smoke 2.15, .77-5.99<br>○ Diabetes 1.21, .6-2.47<br>○ Chronic Obstructive Pulmonary Disease 1.35, .29-6.2<br>○ Coronary Heart Disease 1.85, .85-4.04<br>○ Chronic Renal Disease .66, .11-4.04<br>○ Malignancy .82, .24-2.8		
Zhou Yiwu et al., 2020	Median 43 IQR 31.8-51	366	R		○ Age <65 vs ≥ 65 2.41, 1.02-5.69<br>○ Male 1.2, .63-2.3<br>○ Diabetes 1.73, .28-8.62<br>○ Hypertension 6.75, 2.03-23.2<br>○ Cardiovascular Disease 123.63, 7.99-7441.61	

					○ Chronic Liver Disease 16.72, 1.22-258.17<br>○ Chronic Kidney Disease 39.49, 1.88-1107.01	
He Y. et al., 2020<br>(China)	Median 65	10871	R			○ Age <65 vs ≥ 65 5.21, 2.81-9.8<br>○ Male 1.73, .95-3.21<br>○ Smoke .37, .19-.76<br>○ Chronic Kidney Disease 7.69, 1.72-32.26<br>○ Stroke 7.18, .51-7.69<br>○ Cardiovascular Disease 1.59, .66-3.64<br>○ Chronic Liver Disease .96, .26-2.77<br>○ Autoimmune Disease 3.10, .65-13.33
Zhu B. et al., 2020<br>(China)	Median 47<br>IQR 37-55.5	345	R		○ Age continuous<br>○ Mild median 44<br>○ Severe median 54<br>○ Male 1.41, .83-2.4<br>○ BMI continuous<br>○ Mild mean 23.4<br>○ Severe mean 25.11	
Zhu Y. et al., 2021<br>(Wuhan, China)	(29-93)<br><br>Median 66<br>IQR 58-77	167	R	○ Age continuous 1.04, 1.02-1.06<br>○ Male .94, .6-1.47<br>○ Diabetes .93, .58-1.49<br>○ Hypertension 1.41, .92-2.16<br>○ Coronary Heart Disease 1.51, .96-2.40<br>○ Chronic Lung Disease .82, .36-1.87<br>○ Chonic Renal Failure 1.42, .77-2.61<br>○ Malignant Tumor 1.51, .66-3.46		

				○ Cerebrovascular Disease 1.10, .67-1.79		
Zieleskiewicz et al., 2020<br>(France)	Median 61<br>IQR 54–77	100	R			○ Age continuous<br>○ ICU median 69<br>○ Non-ICU median 60<br>○ Male 4.74, 1.3-17.31<br>○ BMI >30 vs ≤30 2.12, .69-6.55<br>○ Hypertension 5.91, 2.12-16.45<br>○ Coronary Disease 5.08, 1.39-18.63<br>○ Chronic Obstructive Pulmonary Disease .82, .16-4.17<br>○ Chronic Kidney Disease 3.45, .21-57.5<br>○ Diabetes 6.43, 2.05-20.15<br>○ Cancer 2.74, .57-13.24
Zou X. et al., 2020<br>(Wuhan, China)	Mean 60.7<br>Sd 13	154	R	○ Age <60 vs ≥ 60 6.44, 2.76- 15.03<br>○ Male .73, .37-1.45<br>○ Smoking 1.77, .56-5.57<br>○ Hypertension 1.98, .97-4.03<br>○ Diabetes 1.44, .69-3.04<br>○ Coronary Heart Disease 10.27, 3.54-29.79<br>○ Chronic Obstructive Pulmonary Disease 4.3, 1.03-17.97<br>○ Malignancy .48, .05-4.41		
Zuniga et al., 2021<br>(Spain)	(19-96)<br>Mean 64.9<br>Sd 14.1	318	P	○ Age<br>○ Survivor mean 63.3		

				o Nonsurvivor mean 73.9<br><br>(HR)<br>o Age continuous 1.05, 1.02-1.09<br>o Male .43, .17- 1.05<br>o Obesity 1.24, .39-3.90<br>o Asthma 2.24, .55-9.02<br>o Chronic Obstructive Pulmonary Disease 1.73, .55-5.44<br>o Hypertension .56, .19-1.64<br>o Chronic Heart Disease 1.06, .31-3.59<br>o Atrial Fibrillation 1.67, .52-5.38<br>o Immunosuppression 1.74, .23-12.89<br>o Tumour 2.45, .88-6.79<br>o Diabetes 1.22, .50-2.96		
Zuo P. et al., 2020<br>(Wuhan, China)	(65-95)<br><br>Mean 72.95<br>Sd 6.39	446	R	o Male .98, .96–1.0<br>o Age >70 vs ≤70 .98, .97–.99<br>o Hypertension .98, .97–.99<br>o Diabetes .98, .96–1.0		o Male .97, .96–.99<br>o Age >70 vs ≤70 .97, .96–.99<br>o Hypertension .92, .86–.99<br>o Diabetes .94, .90–.99
Thompson et al., 2020 (London)	Median 62<br>IQR (54-70)	156	P			o Age continuous 1.13, 1.07-1.21<br>o Male 1.27, .54-3.17<br>o Black 2.11, .59-7.6<br>o Asian 2.94, .94-9.78<br>o Other .41, .02-3.18<br>o Overweight/Obese 3.06, 1.1-8.74<br>o Smoke .67, .40-1.08

						○ Any Comorbidity 1.29, .46-4.21
Tian et al., 2020 (Wuhan, China)	≥ 18<br><br>Median 64<br>IQR (57-69)	751	R	○ Cancer 2.04, 1.34-3.13	○ Cancer 3.75, 2.71-5.19<br>○ Age continuous 1.04, .85-2.51<br>○ Male 1.46, .85-2.51<br>○ Hypertension 1.7, .97-2.97<br>○ Diabetes .81, .43-1.51<br>○ Coronary Heart Disease 2.05, .73-5.77<br>○ Chronic Kidney Disease 1.14, .2-6.35<br>○ Cardiovascular Disease 1.14, .28-4.68<br>○ Chronic Obstructive Pulmonary Disease 1.14, .1-12.73	
Tobolowsky et al., 2021 (United States)	(51-100)<br><br>Median 83	130	R	Rate Ratio<br>○ Age 51-70 (reference)<br>71-80 1.38, .6-4.9<br>81-90 1.8, .7-4.7<br>91-100 2.3, .9-6<br>○ Female 1.1, .6-1.9<br>○ Hypertension .9, .5-1.6<br>○ Renal Disease 1.7, 1-2.8<br>○ Pulmonary Disease 1.1, .6-1.9<br>○ Diabetes 1, .6-1.7<br>○ Malignancy 1.4, .8-2.4<br>○ Obesity 1, .6-1.7<br>○ Neurologic Disease 1, .6-1.7<br>○ Dementia 1.6, .9-2.6		
Toh et al., 2021 (Australia)	(19-85)<br><br>Median 60<br>IQR (51-69)	103	R	○ Age continuous<br>○ Median nonsurvivors 74.5<br>○ Median survivors 60		○ Age continuous<br>○ ICU median 66.5<br>○ Non-ICU median 60<br><br>Relative Risk

				Relative Risk<br>○ Male 2.6 (p=.62)<br>○ Cardiovascular Disease 16.3 (p=.01)<br>○ Respiratory Disease 1.5 (p=.56)<br>○ Diabetes 19 (p=.01)		○ Male 3.1 (p=.04)<br>○ Hypertension 2.8 (p=.03)<br>○ Cardiovascular Disease 2.7 (p=.03)<br>○ Respiratory Disease 1.7 (p=.32)<br>○ Diabetes 3.2 (p=.02)<br>○ Immunosuppression .95 (p=1)
Tomasoni et al., 2020 (Italy)	Mean 67.4<br>Sd 13.2	692	R	(HR)<br>○ Age (per 5 years) 1.28, 1.13-1.45<br>○ Male 2.14, 1.16-3.94<br>○ Smoker 1.44, 1.01-2.06<br>○ Heart Failure 2.25, 1.26-4.02<br>○ Diabetes 1.34, .95-1.89<br>○ Atrial Fibrillation 1.27, .67-2.14<br>○ Coronary Artery Disease 1.2, .67-2.14<br>○ Chronic Obstructive Pulmonary Disease 1.41, .72-2.75<br>○ Chronic Kidney Disease 1.09, .56-2.10		
Tong et al., 2020 (China)	Median 50<br>IQR (42-57)	39	R		○ Age continuous<br>○ Mild median 49<br>○ Severe median 54<br>○ Male .7, .16-3.13<br>○ Current smoker 4, .48-33.58<br>○ Cardiovascular Disease 8.29, .65-104. 89	

Albalawi et al., 2021 (Saudi Arabia)	Mean 54.2<br>Sd 15.7	119	R	○ Age ≥ 65 vs < 65 5.2, 1.74-15.49<br>○ Male .48, .2-1.16<br>○ Non Saudi vs Saudi. .33, .13-.81<br>○ Kidney Disease .8, .21-3.06<br>○ Diabetes .36, .12-1.15		
Trifan et al., 2020 (Chicago, United States)	Median 64	83	R	○ Male 4.02, 1.14-14.18		
Argirios et al., 2020 (Spain)	n/a	32	R			○ Age continuous<br>○ ICU mean 73.5 Sd 12.9<br>○ Non-ICU mean 68.2 Sd 20.4<br>○ Male 9.09, .98-84.28
Cao et al., 2021 (Wuhan, China)	Median 68<br><br>IQR 61.5-74	74	R	(HR)<br>○ Age ≥ 70 vs < 70 1.8, 1.08-2.99<br>○ Male .99, .57-1.73<br>○ Hypertension 1.38, .83-2.32<br>○ Diabetes 1.22, .65-2.31		
Yildirim et al., 2021 (Turkey)	Mean 49.2<br>Sd 20.8	139	R	○ Age continuous 1.13 .03-1.25<br>○ Gender 1.2 .13-11.46<br>○ Hypertension 1.15, 0-80.73<br>○ Diabetes .34, .04-2.84<br>○ Cardiovascular Disease .29, 0-164.88<br>○ Chronic Obstructive Pulmonary Disease/Asthma 1.72, .05-54.94		

				○ Chronic Renal Insufficiency .14, 0-80.73<br>○ Malignancy .14, 0-4.46		
Turgutalp et al., 2021 (Turkey)	Median 63<br>IQR 53-71	567	R	○ Age continuous<br>○ Median survivor 63<br>○ Median nonsurvivor 66<br>○ Male 1.33, .85-2.08<br>○ Diabetes 1.01, .65-1.58<br>○ Hypertension .81, .48-1.37<br>○ Ischemic Heart Disease 1.35, .86-2.11<br>○ Cardiovascular Disease 1.36, .87-2.13<br>○ Congestive Heart Failure 2.03, 1.24-3.32<br>○ Chronic Obstructive Pumonary Disease 2.17, 1.24-3.79<br>○ Malignancy 1.29, .51-3.26<br><br>(HR)<br>○ Age continuous 1.02, 1.00-1.04	○ Age continuous mild-moderate vs severe-critical<br>○ Median mild 63<br>○ Median severe 64	
Haq et al., 2020 (Pakistan)	≥18<br>Mean 48.7<br>Sd 17.74	179	R	○ Age continuous<br>○ Mean survivor 44.1<br>○ Mean nonsurvivor 60.3<br>○ Male .36, .16-.82		

				○ Age >60 vs ≤ 60 2.7, .5-14<br>○ Unemployed vs Employed 2.13, .97-4.68<br>○ Hypertension 7.38, 3.44-15.85<br>○ Coronary Heart Disease 6.74, 2.86-15.88<br>○ Diabetes Mellitus 3.68, 1.82-7.43<br>○ Asthma 1.24, .36-4.31<br>○ Chronic Obstructive Pulmonary Disease 4.22, 1.42-12.55<br>○ Chronic Kidney Disease 5.04, .45-56.85<br>○ Smoking History 1.79, .88-3.62<br>○ Multi-morbid condition 3.2, 1.1-9.1		
Vanhems et al., 2021 (France)	Median 72<br>IQR 57-83	412	P			○ Age ≥ 70 vs < 70 .75, .47-1.2<br>○ Male 1.66, .76-3.62<br>○ Cardiovascular Disease .61, .38-.97<br>○ Diabetes .97, .54-1.74<br>○ Chronic Lung Disease 1.02, .53-1.94<br>○ Renal Disease 1.63, .88-3.05<br>○ Malignancy 1.09, .56-2.13<br>○ Chronic Nuerological Disease .49, .21-1.13<br>○ Liver Disease .99, .39-2.54<br>○ Immunodeficiency 1.05, .41-2.69

Venturini et al., 2021 (Italy)	≥18<br>Median 75<br>IQR 72-77	176	R	o Age continuous<br>o Median discharged 72<br>o Median deceased 86<br>o Male 1.06, .5-2.25<br>o BMI continuous<br>o Median discharged 25.9<br>o Median deceased 25.2<br>o Hypertension 1.66, .77-3.61<br>o Coronary Artery Disease 3, 1.19-7.54<br>o Diabetes 1.33, .48-3.71<br>o Obesity .84, .22-3.15<br>o Atrial Fibrillation 2.24, .92-5.46<br>o Dementia 6.82, 2.92-15.89<br>o Respiratory Disease .84, .17-4.15<br>o Liver Failure 2.1, .48-9.26		o Age continuous<br>o Median discharged 72<br>o Median ICU 68<br>o Male 2.37, .87-6.48<br>o Hypertension 1.18, .47-2.94<br>o Coronary Artery Disease 2.21, .7-6.91<br>o Diabetes 1.89, .61-5.85<br>o Obesity 2.62, .82-8.38<br>o Atrial Fibrillation .89, .24-3.31<br>o Dementia .31, .04-2.44<br>o Respiratory Disease .66, .08-5.56
Zertuche et al., 2021 (Mexico)	Mean 46.6<br>Sd 15.5	15,529	R	(HR)<br>o Obesity 2.47, .04-2.98<br>o Chronic Obstructive Pulmonary Disease 1.72, 1.05-2.84<br>o Asthma .63, .24-1.7<br>o Immunosuppression 2.54, 1.46-4.43<br>o Hypertension 1.33, 1.08-1.64<br>o Cardiovascular Disease 2.0, 1.15-3.48		(OR)<br>o Diabetes 1.46, 1.06-1.99<br>o Obesity 1.9, 1.46-2.47<br>o Chronic Obstructive Pulmonary Disease 1.49, .65-3.4<br>o Asthma .81, .29-2.2<br>o Immunosuppression 2.17, .92-5.13<br>o Hypertension 1.17, .86-1.59<br>o Cardiovascular Disease 2.03, .95-4.43

				○ Chronic Kidney Disease 1.24, .46-3.34		○ Chronic Kidney Disease 1.53, 1.45-5.23
Verma et al., 2021 (NYC, United States)	(0-21)<br><br>Median 5y IQR 2.5m-15.2y	82	R			○ Age continuous<br>○ Median acute 6.7 months<br>○ Median critical 10 years<br>○ Asthma 2.06, .58-7.32<br>○ Obesity 4.44, 1.5-13.17<br>○ Chronic Lung Disease 8.7, .86-88.49<br>○ Malignancy 3.82, .92-15.78
Llamas et al., 2021 (Spain)	Median 71 IQR 58-82	609	R	○ Age continuous 1.01, 1.06-1.1<br>○ Male 1.61, 1.04-2.51<br>○ BMI continuous .98, .91-1.07<br>○ Charlson Age Index 1.48, 1.34-1.63<br>○ Smoking 1.47, .97-2.24<br>○ Hypertension 2.89, 1.87-4.46<br>○ Diabetes 1.94, 1.27-2.95<br>○ Dyslipidemia 1.63, 1.1-2.42<br>○ Chronic Heart Disease 3.01, 2.01-4.5<br>○ Atrial Fibrillation 3.68, 2.39-6.23<br>○ Chronic Lung Disease 1.42, .92-2.2<br>○ Cerebrovascular Disease 1.7, .9-3.2<br>○ Immunosuppression 2.37, 1.3-4.32		

				○ Chronic Kidney Disease 4.13, 2.22-7.69<br>○ Dementia 2.34, 1.18-4.61<br>○ Chronic Liver Disease .77, .29-2.07		
Villa et al., 2021 (Germany)	Median 62<br>IQR (55-68)	21	R	○ Kidney Transplant w/ Covid 2.75, .38-19.67	○ Age continuous<br>○ Median ARDS 23.8<br>○ Median non-ARDS 67<br>○ BMI continuous<br>○ Median ARDS 23.8<br>○ Median non-ARDS 28.3<br>○ Hypertension 1, .07-13.37<br>○ Diabetes Mellitus .75, .12-4.66	
Vizcarra et al., 2020 (Spain)	≥18<br><br>Mean 53.3<br>Sd 9.5	51	P		○ Age continuous<br>○ Median mild/moderatre 55.4<br>○ Median severe 55.5<br>○ BMI continuous<br>○ Median mild/moderate 25.5<br>○ Median severe 24.3<br>○ Male 2.71, .3-24.42<br>○ Smoker 8.73, 1.03-74.12<br>○ Hypertension 1.2, .33-4.42<br>○ Diabetes .44, .05-4.09	
Vrillon et al., 2021 (France)	≥ 65<br><br>Median 86<br>IQR 82-90	86	P	○ Age continuous<br>○ Median survivors 87<br>○ Median nonsurvivors 86<br>○ Charlson Comorbidity Index<br>○ Median survivors 6<br>○ Median nonsurvivors 6		

				○ Male 1.12, .38-3.31<br>○ Dementia .96, .41-2.27<br>○ Cardiovascular Disease 1.62, .7-3.78<br>○ Hypertension 1.19, .4-3.54<br>○ Atrial Fibrillation 1.01, .38-2.68<br>○ Chronic obstructive Pulmonary Disease 3.3, .92-11.76<br>○ Respiratory Insufficiency 1.16, .12-11.61<br>○ Diabetes 1.49, .52-4.29<br>○ Malignancy .29, .04-2.35		
Vrotsou et al., 2021 (Spain)	≥ 14<br><br>Mean 53.7<br><br>Sd 17.4	14197	R	○ Age continuous<br>○ Mean hospital/ICU 62.8<br>○ Mean nonsurvivors 82.3<br>○ Male 1.94, 1.68-2.25<br>○ Income <18k∈ vs ≥ 18k∈ 2.34, 1.82-3.01		
Vu et al., 2020 (United States)	(26-87)<br><br>Median 54	63	R	○ Age continuous<br>○ Median nonsurvivors 58<br>○ Male 4.75, .55-40.98<br>○ Hispanic 1.19, .27-5.31<br>○ Black .93, .17-5.08<br>○ White .94, .1-8.86<br>○ Obese 1.52, .34-6.75<br>○ Hypertension 9, 1.05-77.29		

				○ Diabetes 1.63, .35-7.5		
Wang F et al., 2020a (Wuhan, China)	Median 64<br>IQR 53-71	7283	R			○ Age ≥ 60 vs < 60 1.76, 1.52-2.03<br>○ Male 1.57, 1.33-1.86<br>○ Other areas vs central Wuhan. 1.62, 1.30-2.04<br>○ Occupation<br>○ Reference: Others<br>○ Medical Workers 1.25, .73-2.13<br>○ Retirees .82, .68-1<br>○ Diabetes 2.11, 1.32-3.38<br>○ Hypertension 2.72, 1.97-3.76<br>○ Cardiovascular Disease 2.15, 1.38-3.33<br>○ Respiratory Disease 3.5, 1.67-7.34
Wang F et al., 2020b (Shenzen, China)	(8 months-86 y)<br><br>Median 46<br>IQR 33-59	333	R		○ Age > 65 vs ≤ 65 3.22, 1.51-6.87<br>○ Male 2.21, 1.2-4.07<br>○ Cormorbidities (yes vs no) 1.78, 1.13-2.81	
Wang Gang et al., 2021 (Wuhan, China)	Median 50.5<br>IQR 37-64	1184	R	○ Wuhan vs Sichuan. 7.64, 2.3-25.27		○ Wuhan vs Sichuan. 1.66, 1.05-2.63
Wang Guyi et al., 2020 (Changsha, China)	Median 45.5<br>IQR 36-60	228	R		(HR)<br>○ Age continuous 1.03, 1.01-1.06<br>○ Gender (unspecified) .62, .27-1.45<br>○ Hypertension 1.58, .59-4.19<br>○ Cardiovascular Disease .89, .12-7.36	
Wang Juan et al., 2020 (China)	(5-87)	562	R		○ Age > 60 vs ≤ 60 3.75, 2.07-6.8	

	Median 47<br>IQR 35-57				○ Male .66, .37-1.2	
Wang Junhong et al., 2020<br>(Wuhan, China)	Median 62.3<br>Sd 14	243	R	○ Age continuous<br>○ Median survivor 64<br>○ Median nonsurvivor 69.5<br>○ Male 2.07, .84-5.09<br>○ Hypertension 1.65, .7-3.89<br>○ Diabetes 2.58, 1.04-6.41<br>○ Coronary Heart Disease .67, .15-3.04<br>○ Chronic Obstructive Pulmonary Disease 4.34, 1.34-14.04<br>○ Stroke 12.36, 1.95-78.25	○ Age continuous<br>○ Median moderate 58.1<br>○ Median severe 64.6<br>○ Male 1.68, .93-3.02<br>○ Hypertension 2.02, 1.11-3.69<br>○ Diabetes 1.22, .58-2.53<br>○ Coronary Heart Disease 1.1, .44-2.71<br>○ Chronic Obstructive Pulmonary Disease 1.95, .39-9.65<br>○ Stroke .81, .13-4.96<br>○ Other Comorbidities .63, .19-2.16	
Wang Lang et al., 2020<br>(Wuhan, China)	(≥ 60)<br><br>Median 69<br>IQR 65-76	339	R	(HR)<br>○ Age continuous 1.06, 1.03-1.1<br>○ Female .61, .27-1.01<br>○ Hypertension 1.49, .92-2.44<br>○ Diabetes 1.09, .57-2.08<br>○ Chronic Liver Disease 2.9, .4-20.9<br>○ Cardiovascular Disease 1.85, 1.06-3.26<br>○ Cerebrovascular Disease 1.38, .65-2.93<br>○ Chronic Obstructive Pulmonary Disease 2.24, 1.12-4.50		

				○ Arrhythmia, .75, .37-1.53<br>○ Acute Kidney Injury 1.16, .56-2.41<br>○ Acute Respiratory Distress Syndrome 29.33, 12.37-69.58<br>○ Cardiac Insufficiency 1.11, .59-2.06<br>○ Malignancy .98, .31-3.13<br>○ Autoimmune Disease 1.1, .15-7.91<br>○ Shock 2.42, .76-7.7<br>○ Acute Cardiac Injury 9.15, 5.3-15.57		
Wang Peng et al., 2020 (China)	(40-59)<br><br>Median 50<br>IQR 45-54	119	R		○ Age continuous<br>○ Median non-severe 50<br>○ Median severe 51<br>○ Male 1.5, .5-4.55	
Wang Sijiao et al., 2020 (Fujian, China)	(16-93)<br><br>Median 46.3<br>Sd 16.4	199	R		○ Age ≥ 75 vs < 75 5.61, 1.16-27.12<br>○ Male 2.23, .56-8.77<br>○ Hypertension 3.44, 1.06-11.16<br>○ Diabetes 6.94, 1.65-29.22<br>○ Cardiovascular Disease 2.51, .45-13.85	
Wang Sufei et al., 2020 (Wuhan, China)	Median 59<br>IQR 47-68	605	R	(OR)<br>○ Age ≥ 65 vs < 65 3.61, 2.37-5.5<br><br>(HR)<br>○ Age continuous 1.02, 1.00-1.04<br>○ Male 1.75, 1.17-2.60		

				○ Hypertension 1.27, .85-1.89<br>○ Chronic Lung Disease 1.12, .41-3.03<br>○ Chronic Heart Disease 1.34, .75-2.39<br>○ Chronic Liver Disease .95, .30-2.99<br>○ Chronic Kidney Disease 2.28, 1.0-5.19<br>○ Cerebrovascular Disease 2.82, 1.31-6.05<br>○ Carcinoma 1.81, .92-3.58		
Wang Tao et al., 2020 (China)	Median 64 IQR 55-70	141	P	(HR)<br>○ Chronic Obstructive Pulmonary Disease 4.57, 1.65-12.69<br>○ Chronic Renal Disease 5.45, 1.85-16.12		(OR)<br>Invasive mechanical ventilation vs noninvasive mechanical ventilation<br><br>○ Male 1.67, .49-5.62<br>○ Age > 65 vs ≤ 65 1.03, .98-1.07
Wang Wen et al., 2021 (Sichuan, China)	Median 44 IQR 33-50	169	R		○ Age > 65 vs ≤ 65 1.92, .5-6.24<br>○ Male 2.27, .85-6.1<br>○ Pulmonary Disease 7.80, 2.14-29.72<br>○ Hypertension 5.05, 1.73-14.48<br>○ Diabetes 3.87, 1.0-14.93<br>○ Cardiovascular Disease 4.68, 1.16-18.93<br>○ Chronic Kidney Disease 5.45, .73-40.79<br>○ Chronic Liver Disease 1.28, .14-12.0)	
Wang Wenjun et al., 2021 (Wuhan, China)	≥ 18	2433	R	(HR)<br>○ Age <60 vs 60-74 1.26, 1.02-1.56	○ Age continuous<br>○ Median mild 36<br>○ Median moderate 58	○ Age continuous<br>○ Median critical 67

	Median<br>60<br>IQR<br>50-68			○ Age <60 vs >74 3.41, 1.07-10.89	○ Median severe 65<br><br>(HR)<br>○ Age <60 vs 60-74 1.26, 1.02-1.56<br>○ Age <60 vs >74 1.44, 1.02-2.03<br><br>(OR)<br>○ Male .85, .71-1.02<br>○ Smoking .98, .7-1.36<br>○ Drinking .74, .49-1.1<br>○ Hypertension 1.64, 1.36-1.99<br>○ Diabetes 1.33, 1.04-1.71<br>○ Coronary Heart Disease 1.96, 1.41-2.73<br>○ Cancer 1.15, .58-2.26<br>○ Cerebrovascular Disease 2.2, 1.42-3.42<br>○ Chronic Kidney Disease 2.08, 1.1-3.91<br>○ Chronic Obstructive Pulmonary Disease 2.79, 1.1-7.07	(OR)<br>○ Male .66, .35-1.24<br>○ Smoking 1.57, .61-4.04<br>○ Drinking 1.66, .58-4.73<br>○ Hypertension .93, .51-1.72<br>○ Diabetes 1.07, .47-2.44<br>○ Coronary Heart Disease 1.56, .55-4.43<br>○ Cancer 1.98, .26-14.87<br>○ Cerebrovascular Disease 3.02, 1.05-8.67<br>○ Chronic Kidney Disease 4.77, 1.41-16.11<br>○ Chronic Obstructive Pulmonary Disease 3.38, .44-25.98
Wang X. et al., 2020 (Wuhan, China)	(16-89)<br><br>Median<br>55.5<br>IQR<br>47-62	1012	R		○ Age ≥ 60 vs < 60 2.1, 1.36-3.25<br>○ Male 1.59, 1.04-2.43<br>○ Hypertension 1.39, .57-3.37<br>○ Diabetes 3.36, 1.38-8.15<br>○ Cardiovascular Disease 4.75, 1.59-14.18<br>○ Respiratory Disease 1.02, .23-4.44<br>○ Other Diseases .88, .26-2.93	
Seo et al., 2020 (Korea)	Mean<br>65years	4932	CS	○ Exposure to RAAS within 1year 0.88 (0.55-1.40)<br>○ Exposure to ACEs inhibitors within 1year 0.71 (0.32-1.58)		

				o Exposure to ARBs within 1 year 01.02 (0.65-1.62)<br>o Exposure to RAAS within 6 months 0.94 (0.81-1.09)<br>o Exposure to ACEs inhibitors within last 6months 0.91 (0.67-1.25)<br>o Exposure to ARBs within last 6months 0.96 (0.83-1.09)<br>o Exposure to RAAS within 3 months 0.95 (0.59-1.52)<br>o Exposure to ACEs inhibitors within last 3months 0.59(0.24-1.46)<br>o Exposure to ARBs within last 3months 1.10 (0.69-1.76)		
Shah et al., 2020(USA)	Median 62 years	77	CS		o Myelofibrosis, AML, ALL, MDS, MPN, CML, CLL HR:1.16 (0.47-2.87)having 1 comorbidity: HR 3.3[illegible] (1.15-9.85)<br>o 2+ comorbidities: HR 5.41 (1.84-15.9)<br>o Time after cellurar therapy:<br>o 101-180 days: HR 0.29(0.0[illegible] 2.75)<br>o 181-365 days: HR 1.08 (0.26-4.51)<br>o 366-1095 days: HR 0.61 (0.17-2.28)<br>o 1096+ days: HR 0.49 (0.13[illegible] 1.90)	

					o Race white vs non-white HR 0.62 (0.27-1.43)<br>o Male vs female: HR: 1.04 (0.46-2.36)<br>o Hematologic malignancy active at the timeof COVID-19 diagnosis: HR 1.96 (0.88-4.38)<br>o Age: HR 1.03 (0.99-1.06)<br>o Absolute neutrophil count: HR 1.15 (1.02-1.29)<br>o Absolute lymphocyte count: HR 1.[illegible] (0.62-1.74)<br>o Neutrophil to lymphocyte ratio: HR 1.03 (1.00-1.07)<br>o Absolute CD4* : HR 1.00 (1.00-1.00)<br>o Absoulte CD8* HR 1.00 (1.00-1.00)<br>o Absolute CD19*: HR 1.00 (1.00-1.00)<br>o Absolute CD16*CD56*NK: HR 1.[illegible] (1.00-1.00)<br>o CD4/CD8 Ratio: HR 0.97 (0.79-1.18)<br>o Never smoker Vs Current or former smokers: HR 0.72 (0.32-1.60)	
Shahriarirad et al., 2020 (Iran)		103	CS	o >=65 years vs < 65 years: OR 1.58 (0.37-6.78)<br>o Male vs Female: OR 0.7[illegible] (0.18-2.84)<br>o Hypertension : OR 1.2 (0.23-6.22)<br>o Diabetes: OR 1.84 (0.35-9.75)<br>o Cardiovascular disease:OR 1.84 (0.35-9.75)<br>o Asthma: OR 2.04 (0.22-19.10)	o >=65 years vs < 65 years: OR 0.87 (0.21-3.51)<br>o Male vs Female: OR 1.04 (0.29-3.78)<br>o Hypertension : OR 4.17 (1.14-15.22)<br>o Diabetes: OR 2.57 (0.60-10.93)<br>o Cardiovascular disease: OR 4.29 (1.09-16.84)<br>o Asthma: OR 1.6 (0.17-14.66)<br>o COPD: OR 1.17 (0.13-10.38)	

				o COPD: OR 1.5 (0.17-13.54)		
Shao et al., 2020 (China)	Common group46.66±14.81 Severe group 58.14±13.96	126	CS		o Age continuous OR: 1.06 1.024-1.086<br>o Co-infection: OR 3.63 (1.60-8.20<br>o Hypertension: OR 3.25 (1.29-8.17)<br>o Male: OR 0.94 (0.43-2.02)<br>o Diabetes: OR 2.37 (0.74-7.62)<br>o Malignancy: OR 1.26 (0.11-14.31)<br>o Cardiovascular diseases: OR 1.26 (0.22-7.23)	
Sadeghi-Haddad-Zavareh et al.,2020 (Iran)	Mean 57.21 ± 16.18	429	CS		o Male: OR 1.35 (0.91-2.00)<br>o Hypertension: OR 0.64 (0.40-1.02)<br>o Cardiovascular disease: OR 0.74 (0.47-1.17)<br>o Diabetes: OR 1.02 (0.67-1.57)<br>o Cancer: OR 0.29 (0.08-1.13)<br>o Chronic liver disease: OR 0.46 (0.0 2.75)<br>o Chronic kidney disease: OR 0.18 (0.04-0.82)<br>o COPD: OR 1.47 (0.51-4.27)	
Sharma.,2020 (USA)	Mean 71	34,881	R	o Age: OR 1.22 (1.05-1.42)<br>o Male: OR 1.02 (0.92-1.12)<br>o BMI: OR 1.04 (1.02-1.04)<br>o Diabetes: OR 1.19 (1.08-1.32)<br>o Hypertension: OR 1.36 (1.09-1.66)<br>o Coronary heart disease: OR 1.12 (0.99-1.27)		
Shi et al., 2020 (China)		85	CS		o Age: OR 1.09 (1.02-1.17)<br>o Male: OR 6.8 (1.44-32.20)	

					o Hypertension: OR 0.21 (0.52-18.88<br>o Dsypnea: OR 2.32 (0.27-20.07)	
Shi et al.,2020 (China)	Mean 59.38 ± 16.54	161	R	o Age >=70 years: OR 5.28 (1.83-15.21)<br>o Male: OR 0.65 (0.24-1.79)<br>o Hypertension: OR 1.58 (0.57-4.37)<br>o Cardiovascular disease: OR 1.83 (0.52-6.42)<br>o Chronic pulmonary disease: OR 2.40 (0.68-8.51)<br>o Diabetes: OR 2.18 (1.13-4.17)		
Shields et al.,2020 (UK)		100	CS	o Female: OR 1.08 (0.30-3.65)<br>o Chronic lung disease: OR 3.33 (0.92-10.87)<br>o Cardiovascular disease: OR 2.20 (0.37-10.86)<br>o Chronic liver disease: 1.72 (0.30-10.74)<br>o Diabetes: OR 7.50 (1.67-32.66)<br>o Chronic GIT disease: OR 0.87 (0.17-4.12)		
Siepmann et al., 2020	Merdian 66	101	CS		o Male: OR 2.77 (1.10-6.99)<br>o Hypertension: OR 5.99 (2.28-15.79<br>o Hyperlipidemia: OR 1.39 (0.49-3.9<br>o Diabetes: OR 3.68 (1.005-13.47)<br>o Atrial fibrillation: OR 2.03 (0.54-7.67)<br>o Smoking: 0.37 (0.11-1.21)<br>o Coronary heart disease: OR 1.40 (0.36-5.44)	

					o Cerebrovascular disease: OR 6.61 (0.83-52.71)<br>o Ischemic stroke: OR 6 (0.74-48.67)	
Singh et al.,2021 (USA)	Mean 50 years	5558	P	o Age: OR 1.05 (1.03-1.07)<br>o Male: OR 1.91 (1.3-2.83)<br>o Smoking (former&current): OR 0.01 (0-0.04)<br>o BMI: OR 0.92 (0.86-0.98)<br>o >= 3 comorbidities: OR 1.64 (1-2.67)<br>o Cardiac failure: OR 1.82 (1.14-2.92)<br>o Chronic kidney disease: OR 1.69 (1.09-2.63)<br>o Peripheral vascular disease: OR 2.27 (1.12-4.59)		
Singh et al.,2020 (USA)	Mean 49 years	41513	CS	o Obesity(>=30kg/ m^2): RR 1.56 (1.41-1.73)		
Singh et al.,2020 (USA)	Mean 55 years	2780	CS	o Liver disease: RR 3.0 (1.5-6.0)		
Singh et al., 2020 (USA)	Mean 51 years	196403	CS		o Inflamatory bowel disease: RR 0.93 (0.68-1.27)	
Smati et al., 2020		1965	CS	BMI:<br>o 25-29.9 vs 18.5-24.9 : OR 1.65 (1.05-2.59)		

				o 30-34.9 Vs 18.5-24.9: OR 1.93 (1.19-3.14)<br>o >=35 vs 18.5-24.9: OR 1.98 (1.11-3.52)		
Smith et al.,2020 (UK)	Mean 67	346	CS	o >=60 years: RR 4.70 (2.40-9.12)<br>o Mean age deacesed: 73.31<br>o Mean age Discharged: 63.56<br>o Male: OR 1.13 (0.72-1.78)<br>o Smoking: OR 1.22 (0.77-1.90)<br>o Hypertension: OR 1.72 (1.03-2.87)<br>o Diabetes: OR 1.96 (1.25-3.08)<br>o Cardiovascular disease: OR 1.79 (1.14-2.8)<br>o Neurological Disease: OR 1.28 (0.78-2.09)<br>o Chronic Kidney Disease: 1.89 (1.14-3.14)<br>o COPD: OR 2.70 (1.52-4.79)<br>o Cancer: OR 1.93 (1.06-3.53)<br>o Asthma: 0.63 (0.32-1.27)<br>o Hypothyroid: OR 1.68 (0.89-3.18)		

				o Hyperlipidemia: RR 0.75 (0.57-0.98)		
Smith et al.,2020(USA)	68 years	86	R		o Non Caucasian vs Caucasian: OR 1.58 (0.64-3.90)<br>o Former/current smokers vs nev smokers: OR 2.87 (1.11-7.38)<br>o Current cancer treatment: 0.78 (0.32-1.94)	
Snipelisky et al.,2020	61 years	156	CS			o Male: OR 2 (0.96-4.19)<br>o CKD: OR 1.19 (0.43-3.33)<br>o COPD: OR 2.07 (0.62-6.91)
Vee et al.,2020 (Malaysia)	28 Years	247	R		o >=65 years vs <65 years: OR :3.82 (0.74-19.58)<br>o COPD: OR 11.74 (1.80-76.54)<br>o Diabetes: OR 56 (15.50-202.31<br>o Hypertension: OR 8.79 (2.90-26.65)<br>o Cardiovascular disease: OR 20.73 (4.57-94.04)<br>o CKD: OR 50.89 (10.94-236.82)	
Soleimani et al.,2020 (Iran)		681	R	o Male: OR 0.86 (0.49-1.50)<br>o Cardiac disease: OR 1.70 (0.95-3.04)<br>o Cerebro vascular disease: OR 21.96 (6.22-77.55)<br>o Chronic lung disease: OR 2.5 (1.03-6.09)<br>o Chronic kidney disease: OR 1.32 (0.55-3.19)<br>o Malignancy: OR 2.84 (0.69-11.71)	o Male: OR 0.4 (0.22-0.71)<br>o Cardiac disease: OR 1.02 (0.58 1.81)<br>o Chronic Lung disease: OR 2.68 (0.77-9.36)<br>o Chronic kidney disease: OR 8.9 (3.57-22.48)<br>o Malignancy: 0.65 (0.15-2.79)<br>o Cerebrovascular disease: OR 2.52 (0.72-8.85)	

Song et al., 2020 (China)	Median 52 years	69	R			o >=60 years vs <60 years: OR6.63 (1.22-35.93)
Song et al, 2020 (China)	Median 39 yaers	41	CS		o Age continuous:<br>o Mild: 37 years (29-44)<br>o Severe: 50 (38-85)<br>o Male: OR 1.41 (0.34-5.78)<br>o Any comorbidity: OR 12.13 (2.31-63.62)<br>o Hypertension: OR 14 (1.36-143.60)<br>o Diabetes: OR 2.7 (0.33-21.83)<br>o Chronic liver disease: OR 5.6 (0.46-68.68)	
Stachura et al., 2021 (Poland)	Mean 59	100	CS		o Age: OR 1.1 (1.01-1.15)<br>o BMI: OR 1.24 (1.01-1.53)<br>o Male: 1.07 (0.47-2.41)<br>o Dyspnea: Ot 7.29 (3.00-17.73)<br>o Hypertension: OR 2.66 (1.18-5.97)<br>o Chronic heart failure: OR 14.0[illegible] (1.72-114.59)<br>o Lactate deshydrogenase: OR 1.16 (1.03-1.3)<br>o Higher D-Dimer: OR 1.73 (1.0[illegible] 2.9)	
Rodrigues et al.,2020 (Canada)		233,566	CS	o Male: OR 1.70 (1.54-1.88)		o Male: OR 2.24 (1.91-2.62)
Stefan et al., 2020 (Romania)	Median 64 years	37	R	o Age: 0.93 (0.83-1.04)<br>o Obesity: OR 1.38 (0.25-7.58)<br>o Smoking: OR 4.48 (0.54-36.92)<br>o Coronary heart disease: OR 0.98 (0.05-17.54)	o Male: OR 0.29 (0.07-1.14)<br>o Smoking:OR 7.64 (0.76-76.90)<br>o Hypertension: 1.91 (0.32-11.47[illegible]<br>o Diabetes: OR 1.43 (0.36-5.61)<br>o Atrial fibrillation: OR 3 (0.67-13.40)<br>o Cancer: OR 1.5 (0.09-26.01)<br>o Obesity:OR 2.27 (0.54-9.53)	

				○ Gender: OR 0.66 (0.12-3.45)<br>○ Hypertension: OR 0.5 (0.07-3.34)<br>○ Diabetes: OR 0.69 (0.11-4.18)<br>○ COPD: OR 11.6 (0.88-158.28)<br>○ Atril fibrillation: OR 5.33 (0.93-30.51)<br>○ Cancer: OR 4.14 (0.22-74.70)		
Steinberg et al., 2020 (USA)		210	R	BMI >30: OR 6.01 (2.5-14.48)		
Suleyman et al., 2020(USA)	Mean 57 Years (SD: 16.8)	463	CS			○ Male: OR 2.0 (1.3-3.2)<br>○ Age>60 years: 1.6 (1.0-2.7)<br>○ African American race: OR 0.9 (0.5-1.6)<br>○ Obesity: OR 2.0 (1.4-3.6)<br>○ CKD: OR 2.0 (1.3-3.3)<br>○ Cancer: OR 1.9 (1.0-3.9)<br>○ Diabetes: 1.3 (0.8-2.2)<br>○ Hypertension: OR 1.0 (0.5-1.8)<br>○ Coronary artery disease: 1.1 (0.6-2.0)
Sun et al.,2020b (China)		244	R	○ Age: OR 1.12 (1.01-1.25)<br>○ Female: 0.32 (0.04-2.41)<br>○ Hypertension: 0.82 (0.24-2.75)<br>○ Diabetes: OR 1.18 (0.64-2.20)		

				○ Coronary heart disease: 1.43 (0.69-2.94)<br>○ Previous respiratory diseases: 3.24 (0.45-23.57		
Suresh et al., 2020	Median 64 years (SD: 17)	1983	R	○ Obesity: OR 1.10 (0.83-1.44		○ Obesity: OR 1.37 (1.07-1.76)
Swann et al.,2020 (UK)	Median (4.6 (0.3-13.7) years	651	P			○ Female vs Male: OR 0.82 (0.51-1.31)<br>○ Age group: ref group (15-19 years)<br>○ <1 months OR 3.21 (1.36-7.66)<br>○ 1 month-<1 year: OR 0.53 (0.22-1.25)<br>○ 1-4 years: 1.28 (0.57-2.89)<br>○ 5-9 years: 1.33 (0.58-3.05)<br>○ 10-14 years: 3.23 (1.55-6.99)<br>○ Ethnicity: Ref(White)<br>○ Black:OR 2.82 (1.41-5.57)<br>○ South Asian: 1.86 (0.87-3.77)<br>○ Other: OR 1.91 (1.07-3.34)<br>○ Any comorbidity:OR 1.42 (0.89-2.28)
Szymanski et al.,2021 (USA)		4968	CS	○ Blood Group A vs O: HR 1.17 (1.02-1.33)		

				○ Blood group B vs O: HR 1.32 (1.10-1.58)		
Talavera et al., 2020 (Spain)	Mean: 67 (SD: 14.7)	576	R	○ Anosmia: OR 0.180 (0.07–0.47)<br>○ Time from clinical onset<br>○ Age: OR 1.056 (1.03–1.08)<br>○ Female: OR 0.67 (0.391–1.148)<br>○ Hypertension: OR 1.31 (0.76–2.28)<br>○ Diabetes: OR 1.33 (0.76–2.34)<br>○ Smoking: OR 1.71 (0.93–3.12)<br>○ Cardiological disorders: OR 1.20 (0.72–2.02)<br>○ Pulmonary disorders: OR 0.83 (0.48 –1.45)<br>○ Cancer: OR 1.40 (0.76–2.56)<br>○ Immunosuppression: OR 1.30 (0.41–4.14)<br>○ Chronic neurological disorders: OR 1.54 (0.87–2.72)		
Tang et al., 2020 (USA)	Mean: 74 years	1970	R	○ Asthma: OR 0.64 (0.30-1.40)<br>○ COPD/emphysema:OR 1.55 (1.08-2.24)<br>○ Cancer: OR 1.22(0.74, 2.01)		

				o Hypertension:HR 0.78 (0.47-1.27)<br>o Atrial fibrillation:HR 0.86 (0.57-1.30)<br>o Coronary heart disease:HR 0.83 (0.58-1.19)<br>o Heart failure: HR 1.13 (0.79-1.62)<br>o Diabates: HR 0.87 (0.63-1.21)<br>o Hypothyroidism: HR 1.06 (0.70-1.61)<br>o HIV: HR 0.59 (0.08-4.36)<br>o Chronic hepatitis C: HR 0.94 (0.29-3.02)<br>o Cerebrovascular disease: HR 0.73 (0.51-1.05)<br>o Dementia: HR 0.99 (0.69-1.42)<br>o CKD (stage1-5): HR 1.02 (0.71-1.46)<br>o End stage kidney disease: HR 1.56 (0.95-2.55)<br>o Depression: HR 1.08 (0.77-1.50)		
				o		

Tao et al., 2020 (China)	Median 55 years	222	R		Anamia 3.47, 1.02-11.75 (aOR)<br>Male 0.83, 0.33-2.13<br>Age ≥60 vs <60 2.66, 1.02-6.96	

	(IQR, 42-66 years)				Hypertension 4.44, 1.72-11.48<br>COPD 6.95, 1.53-31.62<br>Cardiovacular diseases 7.44, 2.40-23.1<br>Any comorbidities 12.22, 3.46-43.19	
Tatum et al., 2020 (USA)	Mean ± SD (58.7 ± 14.8 years)	188	RC	Male 1.41, 0.57-3.57<br>Coormibidities 0.89, 0.1-8.43		
Tehrani et al., 2021 (Swden)	20-103 years (mean 66 ± 17 years)	255	P	Kidney 0.92, 0.45–1.92 (aOR)<br>Heart failure, 3.30,1.54–7.10 (aOR)<br>Ischematic heart disease, 2.82, 1.06–7.51(aOR)<br>Obesity 1.07 0.61–1.87<br>Diabetes 1.35, 0.68–2.66<br>Hypertension 1.37,0.77–2.46		
Tejpal et al., 2021(??)	Adults	10,630	R	Male 1.37, 1.23–1.52		Male 1.32, 1.19-1.45
Telle et al., 2021 (Norwey)	Adult	8569	R	Male 1.7, 1.4–2.1 (aRR)<br>Comorbidity 1.3, 1.1–1.7 (aRR)<br>Risk increases with age		Male 2.8 1.9–4.1 (aRR)<br>Comorbidity 1.1 0.7–1.6 (aRR)<br>Risk increases with age<br>Hospitalization:<br>Male 1.3 1.2–1.5 (aRR)<br>Comorbidity 1.6 1.4–1.8 (aRR)<br>Risk increases with age
Temkin-Greener et al., 2020 (USA)	All	556,792	R	The odds having at least one death in states didn't differ by the percent of residents who wrere male, or with asthma, hypertension, COPD, diabetes, obesity, dementia.		
Tesoriero et al., 2020 (USA)	Mean ± SD, 54.0 ±13.3 years	2988	RC			Female 1.12, 0.84-1.50 (rate ratio)<br>Age ≥60 vs 40 years 4.41, 1.63-11.92, (rate ratio)<br>Hospitalization:<br>Female 1.01, 0.87-1.16 (adjusted rate ratio)<br>Age ≥60 vs 40 years 3.46, 2.66-4.51 (adjusted rate ratio)

Tessitore et al., 2021 (Switherland)	Median (Q1–Q3)= 67 (54–81) years	839	R	CVD 2.9, 1.7 -5.0<br>Male 1.6, 0.98 - 2.6<br>Hypertension 0.80, 0.48 - 1.3<br>Obesity 0.83, 0.54-1.3<br>Diabetes 1.6, 0.91-2.6<br>Smoking 1.1, 0.31-3.1<br>COPD 2.0, 0.94 - 4.3<br>Anaemia 1.5, 0.90-2.3		

CS= Cross-sectional
ICU=intensive care unit
R=Retrospective
P: Prospective
HR= hazards ratio
