# Supplementary material for: Heterogeneity and Risk of Bias in Studies Examining Risk Factors for Severe Illness and Death in COVID-19: A Systematic Review and Meta-Analysis
Source: Pathogens. 2022 May 10;11(5):563. doi: 10.3390/pathogens11050563 (PMC9147100; doi:10.3390/pathogens11050563)
Supplement: Supplementary file 1 [file pathogens-11-00563-s001.zip › Table S4.pdf]

Heterogeneity and risk of bias in studies examining risk factors for severe illness and death in COVID-19: a systematic review and meta-analysis

Abraham Degarege *, Zaeema Naveed, Josiane Kabayundo, David Brett-Major

Table S4: Forest plot showing the relationship of hypertension with the odds of death among COVID-19 patients

Author, year	OR	LCI	UCI
Acar et al., 2020	2.16	1.31	3.57
Aishwarya et al., 2020	0.61	0.09	3.71
Akhtar et al., 2021	1.2	0.75	1.93
Albani et al., 2020	0.89	0.69	1.15
Alberici et al., 2020	0.27	0.05	1.31
Alguwaihes et al., 2021	1	0.4	2.6
Allameh et al., 2020	1.26	0.77	2.06
Almaghlouth et al., 2020	0.68	0.14	3.32
Alser et al., 2021	2.25	1.22	4.17
Anaklı etal., 2021	1.77	0.84	3.73
Anantharamna et al., 2020	1.71	1.13	2.6
Angelidi et al., 2021	0.84	0.15	1.04
Aoun et al., 2021	1.65	0.6	4.55
Atkins et al., 2020	1.42	0.96	2.11
Attauabi et al., 2021	0.75	0.22	2.37
Auld et al., 2020	1.15	0.62	2.13
Azar et al., 2020	3.26	1.83	5.78
Baggio et al., 2021	1.75	1.43	2.14
Barbui et al., 2021	1.67	0.83	3.33
Bartoletti et al., 2020	1.66	0.93	2.96
Bhatt et al., 2021	0.99	0.86	1.14
Bielza et al., 2021	1.1	0.79	1.53
Bossini et al., 2020	0.38	0.08	1.3
Botta et al., 2021	1.16	0.72	1.88
Bottio et al., 2021	0.67	0.18	2.4
Brandão et al., 2021	1.13	0.77	1.66
Brouns et al., 2020	0.54	0.22	1.37
Cabezudo-García et al., 2020	2.96	1.56	5.62
Caillard et al., 2020	1.66	0.99	2.79
Caliskan et al., 2020	1.45	0.69	3.07
Capdevila-Reniu et al., 2021	0.86	0.4	1.83
Cariou et al., 2020	1.82	1.11	2.8
Castelnuovo et al., 2020	2.38	2	2.84
Cen et al., 2020	2.48	1.34	4.59
Chand et al., 2020	1.86	1.15	3.03
Chang et al., 2020	1.89	0.78	4.58
Chen et al., 2021	1.98	1.43	2.75
Chen et al., 2021	1.14	0.87	1.5

Chen L et al., 2020	3	1.81	4.96
Chen T et al., 2020	1.8	0.57	5.61
Chen Y et al., 2020	3.48	2.24	5.4
Chilimuri et al.,2020	1.46	0.82	2.62
Choron et al., 2021	1.04	0.45	2.36
Choudhuri et al., 2020	1.03	0.72	1.47
Ciceri et al., 2020	2.77	1.71	4.52
COVID-ICU group, 2020	1.49	1.31	1.71
Covino et al., 2020	0.37	0.13	1.04
Covino et al., 2020	2.84	1.23	6.57
Covino et al., 2021	1.65	0.81	3.36
Dagher et al., 2021	1.36	0.55	3.37
De Souza et al., 2020	1.53	1.2	1.94
De Souza et al., 2021	4.06	2.41	6.84
Deeb et al., 2021	2.42	1.58	3.71
Deng et al., 2020	3.04	2.56	3.62
Du et al., 2020	4.08	1.58	10.5
Du H et al., 2020	2	0.88	4.53
Duclos et al, 2021	2.5	0.66	9.51
Elhadi et al., 2021	1.02	0.7	1.48
Falcone etal., 2020	2.41	1.39	4.17
Ferrando et al., 2020	1.5	1.07	2.09
Ferrari et al., 2020	1.79	0.84	3.83
Fox et al., 2020	0.52	0.13	2.02
Foy et al., 2020	8.75	5.09	15.03
Frontera et al., 2020	0.7	0.47	1.04
Galiero et al., 2020	1.3	0.89	1.89
Gao et al., 2020	3.79	2.21	6.53
Genet et al., 2020	0.91	0.49	1.65
Giannoglou et al., 2020	3.6	2.21	5.88
Goodall et al., 2020	1.88	1.45	2.46
Grasselli et al., 2020	2.24	1.7	2.95
Grasselli et al., 2020	2.02	1.78	2.3
Guan et al., 2020	6.86	3.85	12.19
Guarneri et al., 2021	1.68	0.88	3.21
Gupta et al., 2020	1.06	0.81	1.36
Haase et al., 2020	1.53	0.96	2.41
Halvatsiotis et al., 2020	0.81	0.32	2.01
Haq et al., 2020	7.38	3.44	15.85
Hashemi et al., 2020	2.2	0.88	5.52
Hatami et al., 2021	1.9	1.26	2.85
Hendra et al., 2021	3.58	0.57	22.69
Hu et al.,2020	1.34	0.45	3.96
Hu J et al., 2020	1.05	0.55	2
Huang S et al., 2020	1.26	0.68	2.33

Huang Y et al., 2020	3.73	1.24	11.18
Hwang et al., 2020	1.74	0.69	4.39
Iaccarino et al., 2020	1.31	1.04	1.66
Iaccarino et al., 2020	2.42	1.73	3.4
Imam Z et al., 2020	1.43	1.05	1.95
Incerti et al., 2021	2.76	2.48	3.07
Inciardi et al., 2020	0.56	0.16	2.01
Islam et al., 2020	3.53	1.53	8.16
Jiang Y et al., 2020	3.21	1.21	8.51
Jimeno et al., 2020	1.9	1.1	2.5
Jung et al., 2020	1.02	0.64	1.61
Kaeuffer et al., 2020	0.6	0.3	0.9
Kim E et al., 2021	1.08	0.51	2.2
Kim et al., 2020	1.32	0.88	1.99
Kim S et al., 2020	2.16	1.09	4.43
Krause et al., 2020	1.97	0.45	8.7
Krishnamoorthy et al., 2021	0.7	0.4	1.2
Krishnan et al., 2020	2.56	1.23	5.31
Kunal et al., 2020	2.94	1.24	7.91
Kute et al., 2021	1.16	0.54	2.52
Lee L et al., 2020	1.95	1.36	2.8
Li et al., 2021	3.74	2.45	5.72
Li G et al., 2021	1.6	0.71	3.63
Li K et al., 2020	2.3	0.75	7.03
Lievre et al., 2020	1.78	1.39	2.27
Lim et al., 2021	3.69	1.5	9.08
Ling et al., 2020	1.25	0.85	1.83
Liu et al., 2020	3.94	1.82	8.53
Liu et al., 2020	0.58	0.27	1.26
Llamas et al., 2021	2.89	1.87	4.46
Lorente et al., 2021	1.21	0.91	2.88
Lu Y et al., 2021	1.12	0.44	2.89
Lucar et al., 2021	0.42	0.09	1.92
Lunski et al., 2021	0.6	0.47	0.78
Maguire et al., 2020	2.02	1.08	3.78
Martinez-Lopez et al., 2020	1.7	0.8	3.5
Martins et al., 2021	3.6	0.7	18.46
Mash et al., 2020	3.95	2.73	5.7
Mato et al., 2020	1.35	0.75	2.45
Matsuzawa et al., 2020	0.63	0.15	2.34
Mayer et al., 2021	1.16	0.98	1.37
Menager et al., 2021	1.17	0.27	5.08
Mendes et al., 2020	1.18	0.64	2.17
Moghaddam et al., 2020	1.0	0.15	6.53
Molinero et al., 2020	5.64	3.13	10.87

Morrison et al., 2020	0.79	0.29	2.13
Mostaza et al., 2020	0.69	0.44	1.08
Murthy et al., 2021	1.92	1.36	2.72
Nachega et al., 2020	3.15	2.04	4.85
Nakamura et al., 2020	2.4	0.54	10.69
Namendys-Silva., 2021	2.41	1.26	4.62
Nepogodiev et al., 2020	1.73	1.31	2.29
Ng et al., 2020	0.84	0.73	0.98
Nie et al., 2020	5.69	1.32	24.54
Nikpouraghdam et al., 2020	1.81	0.85	3.87
Okoh et al., 2020	2.21	1.23	4.04
Otero et al., 2021	4.82	1.02	22.75
Oto et al., 2021	1.15	0.29	4.51
Ozdemir et al., 2021	3.02	1.67	5.45
Ozturk ey al., 2020	2.56	1.75	3.76
Pascual et al., 2020	0.83	0.05	15.09
Park et al., 2020	1.48	0.91	2.4
Park et al., 2020b	1.36	0.79	2.33
Parlak et al., 2021	0.82	0.23	2.98
Patel et al., 2020	1.54	1.02	2.32
Peiro et al., 2021	2.81	1.33	5.93
Pineiro et al., 2021	1.85	0.6	5.66
Plotnikow et al., 2020	1.58	0.27	9.31
Pongpirul et al., 2020	5.44	2.3	12.83
Qin W et al., 2021	2.13	0.9	5.03
Qin W et al., 2021	2.03	1.26	3.27
Raimondi et al., 2021	3.09	2	4.78
Ramaswamy et al., 2020	1.26	0.35	4.59
Ramos-Rincon et al., 2020	0.96	0.81	1.14
Rastad et al., 2021	1.12	0.43	2.89
Rajter et al., 2021	0.72	0.17	3.08
Rivera-Izquierdo et al.,2020	5.25	2.69	10.26
Rodriguez et al., 2020	3.0	0.67	13.53
Rokni et al., 2020	1.94	0.76	4.97
Rosenthal et al., 2020	1.08	0.99	1.18
Rottoli et al., 2020	1.09	0.56	2.15
Russo et al., 2020	1.34	0.82	2.19
Saeed et al., 2020	0.89	0.68	1.16
Sai et al., 2021	0.68	0.2	2.33
Saifi et al., 2021	0.87	0.15	5.06
Salazer et al., 2021	1.69	1.46	1.95
Sands et al., 2020	0.63	0.48	0.82
Santos et al., 2020	9	1	80.8
Shahriarirad et al., 2020	1.2	0.23	6.22
Shi et al.,2020	1.58	0.57	4.37

Smith et al.,2020	1.72	1.03	2.87
Stefan et al., 2020	0.5	0.07	3.34
Sun et al.,2020	0.82	0.24	2.75
Talavera et al., 2020	1.31	0.76	2.28
Tedeschi et al., 2020	4.31	2.91	6.39
Tehrani et al., 2021	1.37	0.77	2.46
Tessitore et al., 2021	0.8	0.48	1.3
Cao et al., 2020	7.33	2.37	22.65
Turgutalp et al., 2021	0.81	0.48	1.37
Venturini et al., 2021	1.66	0.77	3.61
Vrillon et al., 2021	1.19	0.4	3.54
Vu et al., 2020	9	1.05	77.29
Wang et al., 2020	5	1.74	14.3
Wang et al.,2020	1.53	0.89	2.64
Wang J et al., 2020	1.65	0.7	3.89
Wargny et al., 2021	0.84	0.51	1.39
Webb et al., 2020	1.03	0.34	3.09
Xu Jingyuan et al., 2020	2.02	1.16	3.54
Xu Jiqian et al., 2020	0.96	0.57	1.67
Xu Peng et al., 2020	6.4	3.12	13.11
Yan et al., 2020	4.81	2.48	9.35
Yang C. et al., 2021	3.8	2.09	6.93
Yang etal., 2020	1.68	0.67	4.21
Yang G. et al., 2020	1.68	0.67	4.21
Yang K. et al., 2020	0.74	0.34	1.59
Yao et al., 2020	13.53	3.55	51.5
Yao et al., 2021	2	0.75	5.3
Yarza et al., 2020	2	0.68	5.88
Yildirim et al., 2021	1.15	0	80.73
Zhang et al., 2020	2.17	0.38	12.46
Zhao Y. et al., 2020	4.24	2.76	6.51
Zhou S. et al., 2020	2.96	1.56	5.61
Zhou W et al., 2021	2.36	1.17	4.78
Zhu Y. et al., 2021	1.41	0.92	2.16
Zou X. et al., 2020	1.98	0.97	4.03
Zuo P. et al., 2020	0.98	0.97	0.99
**Total (I squared=77.3%, p<0.01)**	**1.38**	**1.30**	**1.46**
