# Supplementary material for: Heterogeneity and Risk of Bias in Studies Examining Risk Factors for Severe Illness and Death in COVID-19: A Systematic Review and Meta-Analysis
Source: Pathogens. 2022 May 10;11(5):563. doi: 10.3390/pathogens11050563 (PMC9147100; doi:10.3390/pathogens11050563)
Supplement: Supplementary file 1 [file pathogens-11-00563-s001.zip › Table S5.pdf]

Heterogeneity and risk of bias in studies examining risk factors for severe illness and death in COVID-19: a systematic review and meta-analysis

Abraham Degarege *, Zaeema Naveed, Josiane Kabayundo, David Brett-Major

Table S5: Forest plot showing the relationship of hypertension with the odds of severe illness among COVID-19 patients

Author, year	OR	LCI	UCI
Aiswarya et al., 2020	0.95	0.18	4.83
Algado-Sellés et al., 2020	3.2	0.7	13.5
Allard et al., 2020	1.44	0.63	3.32
Allenbach et al., 2020	1.05	0.52	2.1
Alqahtani et al., 2020	92.8	39.26	219.36
Alsharidah etal., 2021	1.34	0.86	2.09
Annweiler et al., 2020	0.51	0.11	2.33
Basaran et al., 2021	2.47	1.03	5.94
Basaran et al., 2021	2.47	1.03	5.94
Bauer et al., 2020	1.33	0.95	1.84
Bhargava et al., 2020	1.55	0.81	2.98
Bolouri et al., 2021	0.84	0.29	2.38
Bravi et al., 2020	1.1	0.8	1.47
Cabello et al., 2021	5.09	1.34	19.02
Cai et al., 2020	2.28	1.25	4.14
Cekerevac et al., 2021	3.78	1.81	7.91
Cen et al., 2020	2.9	2.12	3.96
Chaudhry et al., 2020	1.61	0.28	9.23
Chen et al., 2020	1.81	0.71	4.62
Chen Q et al., 2020	1.81	0.71	4.62
Chen Z et al., 2020	2.41	1.68	3.43
Cheng et al., 2020	1.45	0.64	3.19
Cizmecioglu et al., 2020	1	0.22	4.5
Colaneri et al., 2020	0.7	0.19	2.61
Deng et al., 2020	1.53	0.67	3.51
Du et al., 2020	0.8	0.37	1.73
Du et al., 2020	0.8	0.37	1.73
Duan et al., 2020	3.31	1.02	10.73
Feng et al., 2020	1.82	1.15	2.87
Gao et al., 2020	1.86	1.56	2.23
Gao et al., 2020	3.36	1.41	9.52
Ghaffari et al., 2021	1.75	1.07	2.87
Guan et al., 2020	3.37	2.5	4.57
Hu et al., 2020	1.78	1.11	2.88
Hu et al., 2021	2.75	0.89	8.43
Huang et al., 2020	0.53	0.12	2.42
Huang S et al., 2020	1.56	0.93	2.62
Jain et al., 2021	1.63	0.5	5.29
Jang et al., 2020	1	0.09	10.78
Kaeuffer et al., 2020	1.14	0.89	1.47
Karahan etal., 2020	4.66	2.22	9.8

Khan et a., 2020	2.28	1.23	4.2
Lara et al., 2020	1.95	0.69	5.49
Li et al., 2020	2.2	1.51	3.2
Li et al.,2020	1.59	0.24	10.18
Li T et al., 2020	6.16	3.46	10.97
Liu J etal., 2021	2.03	0.93	4.46
Liu S et al., 2020	1.06	0.47	2.4
Molinero et al., 2020	1.48	1	3.22
Nachega et al., 2020	3.66	2.57	5.21
Nguyen et al., 2020	1.19	0.71	1.99
Nie Y et al., 2020	0.49	0.16	1.45
Pei et al., 2020	1.71	1.06	2.76
Pereira et al., 2020	1.66	0.64	4.38
Qin et al., 2020	2.62	1.65	4.17
Ren et al., 2020	3.29	1.42	7.63
Sadeghi-Haddad-Zavareh et al.,2020	0.64	0.4	1.02
Shahriarirad et al., 2020	4.17	1.14	15.22
Shao et al., 2020	3.25	1.29	8.17
Shi et al., 2020	0.52	0.21	18.88
Shi et al.,2020	5.65	3.05	10.45
Siepmann et al., 2020	5.99	2.28	15.79
Song et al, 2020	14	1.36	21.83
Stachura et al., 2021	2.66	1.18	5.97
Stefan et al., 2020	1.91	0.32	11.47
Sun et al., 2020	12.6	2.18	73.4
Tao et al., 2020	4.44	1.72	11.48
Tian et al., 2020	1.7	0.97	2.97
To et al., 2020	3.66	0.51	26.2
Vee et al., 2020	8.79	2.9	26.65
Villa et al., 2021	1	0.07	13.37
Vizcarra et al., 2020	1.2	0.33	4.42
Wan et al., 2020	1.06	0.3	3.67
Wang et al., 2021	0.8	0.3	2.13
Wang Junhong et al., 2020	2.02	1.11	3.69
Wang Sijiao et al., 2020	3.44	1.06	11.16
Wang Wen et al., 2021	5.05	1.73	14.48
Wang Wenjun et al., 2021	1.64	1.36	1.99
Wang X. et al., 2020	1.39	0.57	3.37
Wei et al., 2020	2.1	1.22	3.6
Wei X et al., 2020	2.37	1.62	3.46
Xie Y et all., 2020	5.37	1.76	16.87
Xiong S et al., 2020	1.98	0.93	4.23
Yang et al., 2020	1.13	0.65	1.98
Yang G. et al., 2020	1.44	0.76	2.73
Yang Q. et al., 2020	4.7	2.02	10.94

Yu et al., 2020	0.7	0.2	2
Yu Y. et al., 2020	0.7	0.13	3.86
Zhang et al., 2020	10.7	3.56	32.12
Zhang et al., 2020	22	6.03	80.3
Zhang Hongmei et al., 2020	3.3	0.96	11.31
Zhang Hongyan et al., 2020	1.66	0.74	3.74
Zhang Jing et al., 2020	1.97	1.43	2.71
Zhang Nannan et al., 2021	1.24	0.13	11.75
Zhang Q et al., 2020	1.69	0.65	4.38
Zhang Y et al., 2021	0.86	0.5	1.47
Zhao J et al., 2021	2.18	1.31	3.64
Zheng B et al., 2021	3.92	1.45	10.62
Zhou Jun et al., 2021	4.81	1.03	14.21
Zhou Yiwu et al., 2020	6.75	2.03	23.2
**Total (I squared= 47.9%, p<0.01)**	**1.59**	**1.41**	**1.76**
