# Supplementary material for: Heterogeneity and Risk of Bias in Studies Examining Risk Factors for Severe Illness and Death in COVID-19: A Systematic Review and Meta-Analysis
Source: Pathogens. 2022 May 10;11(5):563. doi: 10.3390/pathogens11050563 (PMC9147100; doi:10.3390/pathogens11050563)
Supplement: Supplementary file 1 [file pathogens-11-00563-s001.zip › Table S6.pdf]

# Heterogeneity and risk of bias in studies examining risk factors for severe illness and death in COVID-19: a systematic review and meta-analysis

Abraham Degarege *, Zaeema Naveed, Josiane Kabayundo, David Brett-Major

**Table S6: Forest plot showing the relationship of cardiovascular disease with the odds of death among COVID-19 patients**

| Author, year | OR | LCI | UCI | % Weight |
|---|---|---|---|---|
| Acar et al., 2020 | 3.29 | 1.85 | 5.85 | 0.34 |
| Agarwal et al., 2020 | 2.00 | 1.20 | 3.40 | 0.89 |
| Alberici et al., 2020 | 3.11 | 1.02 | 9.60 | 0.08 |
| Alguwaihes et al., 2021 | 1.40 | 0.40 | 4.30 | 0.35 |
| Allameh et al., 2020 | 1.51 | 0.88 | 2.62 | 1.20 |
| Anaklı etal., 2021 | 1.84 | 0.81 | 4.18 | 0.45 |
| Angelidi et al., 2021 | 2.47 | 0.43 | 14.39 | 0.03 |
| Aoun et al., 2021 | 4.42 | 2.06 | 9.49 | 0.11 |
| Attauabi et al., 2021 | 4.28 | 1.15 | 16.50 | 0.03 |
| Auld et al., 2020 | 1.13 | 0.54 | 2.35 | 1.15 |
| Azar et al., 2020 | 7.95 | 4.35 | 14.51 | 0.06 |
| Baggio et al., 2021 | 1.26 | 1.07 | 1.49 | 2.77 |
| Baqui et al., 2020 | 2.32 | 2.09 | 2.55 | 2.73 |
| Barbui et al., 2021 | 2.18 | 0.64 | 7.36 | 0.13 |
| Berenguer et al., 2020 | 3.21 | 2.75 | 3.75 | 2.02 |
| Bertsimas et al., 2020 | 1.74 | 1.2 | 2.54 | 1.59 |
| Betech et al., 2020 | 1.12 | 1.08 | 1.16 | 3.01 |
| Bossini et al., 2020 | 1.94 | 0.43 | 8.15 | 0.10 |
| Brandão et al., 2021 | 1.32 | 0.67 | 2.62 | 1.04 |
| Cabezudo-García et al., 2020 | 2.48 | 1.38 | 4.46 | 0.53 |
| Caillard et al., 2020 | 2.38 | 1.51 | 3.76 | 0.86 |
| Catteau et al., 2020 | 2.46 | 2.23 | 2.73 | 2.68 |
| Cen et al., 2020 | 5.01 | 2.35 | 10.68 | 0.09 |
| Chand et al., 2020 | 1.72 | 0.62 | 4.78 | 0.31 |
| Chen et al., 2021 | 1.41 | 0.94 | 2.13 | 1.77 |
| Chen T et al., 2020 | 2.86 | 0.74 | 11.06 | 0.06 |
| Chen Y et al., 2020 | 8.29 | 5.02 | 13.68 | 0.08 |
| Chilimuri et al.,2020 | 1.56 | 0.78 | 3.11 | 0.82 |
| Covino et al., 2020 | 1.00 | 0.33 | 2.69 | 0.8 |
| Covino et al., 2020 | 2.49 | 1.09 | 5.67 | 0.26 |
| Covino et al., 2020 | 1.35 | 0.6 | 3.01 | 0.78 |
| Dagher et al., 2021 | 1.16 | 0.59 | 2.28 | 1.25 |
| De Souza et al., 2020 | 1.15 | 0.95 | 1.39 | 2.75 |
| Deeb et al., 2021 | 2.84 | 1.61 | 5.01 | 0.45 |
| Deng et al., 2020 | 5.42 | 4.33 | 6.79 | 0.75 |
| Du et al., 2020 | 11.06 | 4.06 | 30.06 | 0.01 |
| Duarte et al., 2020 | 1.39 | 0.93 | 2.07 | 1.83 |
| Elhadi et al., 2021 | 0.92 | 0.58 | 1.44 | 2.21 |
| Escosteguy et al., 2021 | 1.31 | 0.85 | 2.04 | 1.77 |
| Falcone etal., 2020 | 2.86 | 1.65 | 4.93 | 0.48 |
| Fava et al., 2020 | 1.81 | 0.71 | 4.52 | 0.37 |
| Ferrando et al., 2020 | 4.64 | 1.15 | 18.74 | 0.02 |

| | | | | |
|---|---|---|---|---|
| Ferrari et al., 2020 | 2.51 | 0.94 | 6.68 | 0.17 |
| Genet et al., 2020 | 1.11 | 0.59 | 2.04 | 1.48 |
| Giannoglou et al., 2020 | 2.56 | 1.27 | 5.15 | 0.36 |
| Goodall et al., 2020 | 2.12 | 1.57 | 2.85 | 1.66 |
| Grasselli et al., 2020 | 2.11 | 1.75 | 2.55 | 2.29 |
| Guan et al., 2020 | 5.56 | 2.48 | 12.45 | 0.06 |
| Halvatsiotis et al., 2020 | 0.59 | 0.17 | 1.99 | 1.14 |
| Haq et al., 2020 | 6.74 | 2.86 | 15.88 | 0.04 |
| Hashemi et al., 2020 | 0.98 | 0.46 | 2.09 | 1.30 |
| Hatami et al., 2021 | 1.70 | 1.07 | 2.7 | 1.30 |
| Hendra et al., 2021 | 0.77 | 0.26 | 2.25 | 1.02 |
| Hu J et al., 2020 | 2.13 | 0.93 | 4.91 | 0.34 |
| Hwang et al., 2020 | 3.5 | 1.03 | 12.21 | 0.05 |
| Iaccarino et al., 2020 | 1.12 | 0.8 | 1.55 | 2.36 |
| Islam et al., 2020 | 5.05 | 1.64 | 15.48 | 0.03 |
| Jimeno et al., 2020 | 2.30 | 1.10 | 4.60 | 0.43 |
| Jung et al., 2020 | 1.31 | 0.70 | 2.27 | 1.36 |
| Kim E et al., 2021 | 1.76 | 0.44 | 6.93 | 0.14 |
| Kim S et al., 2020 | 3.81 | 0.99 | 13.06 | 0.04 |
| Krause et al., 2020 | 2.25 | 0.81 | 6.26 | 0.19 |
| Krishnamoorthy et al., 2021 | 1.43 | 0.75 | 2.74 | 1.02 |
| Kunal et al., 2020 | 1.53 | 0.47 | 5.02 | 0.27 |
| Laake et al., 2020 | 2.13 | 1.26 | 3.61 | 0.81 |
| Lee L et al., 2020 | 2.32 | 1.47 | 3.64 | 0.90 |
| Li et al., 2021 | 1.36 | 0.81 | 2.31 | 1.42 |
| Liu et al., 2020 | 0.9 | 0.43 | 1.88 | 1.48 |
| Llamas et al., 2021 | 3.01 | 2.01 | 4.50 | 0.74 |
| Louapre et al., 2020 | 5.27 | 2.39 | 13.66 | 0.05 |
| Lucar et al., 2021 | 0.93 | 0.13 | 6.63 | 0.14 |
| Martinez-Lopez et al., 2020 | 1.22 | 0.56 | 2.66 | 0.95 |
| Martins et al., 2021 | 3.78 | 0.98 | 14.56 | 0.03 |
| Mash et al., 2020 | 2.85 | 1.52 | 5.35 | 0.36 |
| Mather et al., 2020 | 1.44 | 0.8 | 2.62 | 1.14 |
| Mehta et al., 2021 | 1.12 | 1.08 | 1.16 | 3.01 |
| Moghaddam et al., 2020 | 0.93 | 0.16 | 5.45 | 0.2 |
| Molinero et al., 2020 | 1.02 | 0.39 | 2.30 | 1.07 |
| Nachega et al., 2020 | 4.78 | 2.20 | 10.37 | 0.09 |
| Nachtigall et al., 2020 | 2.25 | 1.76 | 2.88 | 1.86 |
| Nepogodiev et al., 2020 | 1.81 | 1.13 | 2.89 | 1.19 |
| Nikpouraghdam et al., 2020 | 1.38 | 0.48 | 3.95 | 0.43 |
| Nunez-Gil, 2021 | 3.58 | 2.95 | 4.34 | 1.54 |
| Oto et al., 2021 | 3.70 | 1.04 | 13.08 | 0.04 |
| Park et al., 2020 | 1.37 | 0.51 | 3.71 | 0.50 |
| Patel et al., 2020 | 2.11 | 1.34 | 3.36 | 1.00 |
| Rivera-Izquierdo et al., 2020 | 12.72 | 6.28 | 25.76 | 0.02 |
| Rodriguez et al., 2020 | 3.25 | 0.39 | 26.92 | 0.01 |

| | | | | |
|---|---|---|---|---|
| Rokni et al., 2020 | 1 | 0.28 | 3.58 | 0.47 |
| Rossi et al., 2020 | 1.21 | 0.78 | 1.88 | 1.88 |
| Sai et al., 2021 | 1.4 | 0.29 | 6.82 | 0.14 |
| Saifi et al., 2021 | 0.45 | 0.11 | 1.85 | 1.20 |
| Salazer et al., 2021 | 2.09 | 1.68 | 2.60 | 2.12 |
| Sands et al., 2020 | 1.19 | 0.9 | 1.58 | 2.45 |
| Shahriarirad et al., 2020 | 1.84 | 0.35 | 9.75 | 0.07 |
| Sharma,2020 | 1.12 | 0.99 | 1.27 | 2.90 |
| Shi et al., 2020 | 1.83 | 0.52 | 6.42 | 0.16 |
| Shields et al., 2020 | 2.20 | 0.37 | 10.86 | 0.05 |
| Smith et al., 2020 | 1.79 | 1.14 | 2.80 | 1.27 |
| Soleimani et al., 2020 | 1.7 | 0.95 | 3.04 | 0.95 |
| Tu et al., 2020 | 8.89 | 1.36 | 58.08 | 0.00 |
| Turgutalp et al., 2021 | 1.36 | 0.87 | 2.13 | 1.69 |
| Venturini et al., 2021 | 3.00 | 1.19 | 7.54 | 0.14 |
| Vrillon et al., 2021 | 1.62 | 0.7 | 3.78 | 0.53 |
| Wang et al., 2020 | 7.97 | 2.29 | 27.75 | 0.01 |
| Wang et al., 2020 | 3.60 | 1.9 | 6.82 | 0.23 |
| Wang Junhong et al., 2020 | 0.67 | 0.15 | 3.04 | 0.59 |
| Webb et al., 2020 | 0.65 | 0.15 | 2.71 | 0.71 |
| Wei et al.,2020 | 3.29 | 1.66 | 6.54 | 0.24 |
| Xu Jingyuan et al., 2020 | 0.35 | 0.18 | 0.68 | 2.68 |
| Xu Jiqian et al., 2020 | 0.93 | 0.45 | 1.93 | 1.44 |
| Xu Peng et al., 2020 | 18.26 | 7.90 | 42.18 | 0.01 |
| Y.Wang et al., 2020 | 1.14 | 0.48 | 2.71 | 0.87 |
| Yan et al., 2020 | 6.75 | 2.25 | 20.16 | 0.02 |
| Yang C. et al., 2021 | 3.33 | 0.86 | 12.86 | 0.04 |
| Yang K. et al., 2020 | 2.00 | 0.65 | 6.12 | 0.19 |
| Yao et al., 2021 | 2.67 | 0.89 | 7.99 | 0.12 |
| Yildirim et al., 2021 | 0.29 | 0.00 | 164.88 | 0.00 |
| Zhao Y. et al., 2020 | 4.45 | 2.25 | 8.79 | 0.14 |
| Zhong Y. et al., 2020 | 2.03 | 0.45 | 9.08 | 0.08 |
| Zhou S. et al., 2020 | 3.58 | 1.52 | 8.44 | 0.12 |
| Zhu Y. et al., 2021 | 1.51 | 0.96 | 2.40 | 1.49 |
| Zou X. et al., 2020 | 10.27 | 3.54 | 29.79 | 0.01 |
| **Total (I squared=80.2%, p<0.01)** | **1.63** | **1.51** | **1.75** | 100.00 |
