# Supplementary material for: Heterogeneity and Risk of Bias in Studies Examining Risk Factors for Severe Illness and Death in COVID-19: A Systematic Review and Meta-Analysis
Source: Pathogens. 2022 May 10;11(5):563. doi: 10.3390/pathogens11050563 (PMC9147100; doi:10.3390/pathogens11050563)
Supplement: Supplementary file 1 [file pathogens-11-00563-s001.zip › Table S7.pdf]

Heterogeneity and risk of bias in studies examining risk factors for severe illness and death in COVID-19: a systematic review and meta-analysis

Abraham Degarege *, Zaeema Naveed, Josiane Kabayundo, David Brett-Major

Table S7: Forest plot showing the relationship of cardiovascular disease with the odds of severe illness among COVID-19 patients

Author, year	OR	LCI	UCI	% Weight
Algado-Sellés et al., 2020	2.30	0.40	12.10	0.12
Allenbach et al., 2020	1.44	0.65	3.20	2.09
Alqahtani et al., 2020	4.51	1.09	18.67	0.05
Al-Samkari et al., 2020	0.90	0.58	1.41	8.02
Bauer et al., 2020	1.72	1.09	2.70	4.16
Betti et al., 2021	0.38	0.09	1.50	4.91
Bhargava et al., 2020	1.68	0.76	3.71	1.63
Bravi et al., 2020	1.88	1.32	2.70	5.03
Cai et al., 2020	3.49	1.71	7.12	0.54
Cen et al., 2020	2.36	1.35	4.11	1.83
Chen Z et al., 2020	3.15	1.9	5.23	1.32
Cheng et al., 2020	1.33	0.32	6.00	0.49
Cizmecioglu et al., 2020	1.90	0.23	15.58	0.07
Colaneri et al., 2020	2.40	0.59	9.63	0.2
Deng et al., 2020	2.01	0.59	6.77	0.42
Du et al., 2020	0.68	0.30	1.52	5.77
Du et al., 2020	0.68	0.30	1.52	5.77
Feng et al., 2020	2.50	1.27	4.92	1.12
Ghaffari et al., 2021	1.42	0.78	2.58	3.57
Guan et al., 2020	2.84	1.62	4.96	1.31
Jinag N et al., 2021	3.53	0.98	12.64	0.12
Kaeuffer et al., 2020	0.88	0.6	1.31	8.81
Khan et a., 2020	4.30	1.76	10.51	0.21
Li T et al., 2020	2.65	1.60	4.40	1.79
Molinero et al., 2020	0.86	0.43	1.71	5.48
Nachega et al., 2020	7.77	3.49	17.28	0.09
Nie Y et al., 2020	0.81	0.31	2.14	3.49
Ouldali et al., 2021	2.10	0.10	13.10	0.10
Qin et al., 2020	4.97	1.47	19.79	0.05
Ren et al., 2020	4.63	0.41	52.63	0.01
Sadeghi-Haddad-Zavareh et al., 2020	0.74	0.47	1.17	8.88
Shahriarirad et al., 2020	4.29	1.09	16.84	0.07
Shao et al., 2020	1.26	0.22	7.23	0.33
Shi et al., 2020	5.47	1.54	19.41	0.05
Soleimani et al., 2020	1.02	0.58	1.81	5.72
Tian et al., 2020	1.14	0.28	4.68	0.79
Tong et al., 2020	8.29	0.65	104.89	0.00
Vee et al., 2020	20.7	4.57	94.04	0.00
Wang et al., 2021	4.60	0.78	27.20	0.02
Wang Junhong et al., 2020	1.10	0.44	2.71	2.52
Wang Sijiao et al., 2020	2.51	0.45	13.85	0.09
Wang Wen et al., 2021	4.68	1.16	18.93	0.05

Wang Wenjun et al., 2021	1.96	1.41	2.73	5.30
Wang X et al., 2020	4.75	1.59	14.18	0.10
Wei et al., 2020	3.56	1.25	10.13	0.20
Wei Z et al, 2021	1.08	0.25	4.62	0.80
Xie Y et al., 2020	3.34	1.12	9.94	0.21
Xiong et al., 2020	2.77	0.98	7.86	0.34
Xiong S et al., 2020	4.41	1.34	14.49	0.09
Yang Q. et al., 2020	7.41	1.74	31.57	0.02
Yu et al., 2020	4.20	1.20	14.20	0.10
Yu Y. et al., 2020	0.91	0.24	3.37	1.47
Zhang et al., 2020	4.30	1.07	17.20	0.06
Zhang et al., 2020	11.5	0.98	134.40	0.00
Zhang Hongmei et al., 2020	2.55	0.25	25.86	0.03
Zhang Hongyan et al., 2020	2.55	0.75	8.73	0.25
Zhang L et al., 2020	1.64	0.50	5.10	0.73
Zhang Q et al., 2020	3.54	1.02	12.24	0.13
Zhang Y et al., 2021	1.13	0.50	2.52	3.02
Zhao J et al., 2021	6.25	1.77	22.09	0.04
Zhou Y et al., 2020	123.63	7.99	7441.61	0.00
**Total (I squared=20.5%, p=0.086)**	**1.27**	**1.07**	**1.47**	**100.00**
