# Supplementary material for: Heterogeneity and Risk of Bias in Studies Examining Risk Factors for Severe Illness and Death in COVID-19: A Systematic Review and Meta-Analysis
Source: Pathogens. 2022 May 10;11(5):563. doi: 10.3390/pathogens11050563 (PMC9147100; doi:10.3390/pathogens11050563)
Supplement: Supplementary file 1 [file pathogens-11-00563-s001.zip › Table S8.pdf]

Heterogeneity and risk of bias in studies examining risk factors for severe illness and death in COVID-19: a systematic review and meta-analysis

Abraham Degarege *, Zaeema Naveed, Josiane Kabayundo, David Brett-Major

Table S8: Forest plot showing the relationship of diabetes with the odds of death among COVID-19 patients

Author, year	OR	LCI	UCI	% Weight
Acar et al., 2020	2.61	1.46	4.63	0.32
Aiswarya et al., 2020	2.57	0.61	10.71	0.05
Akbariqomi et al., 2020	2.23	1.3	3.81	0.42
Akhtar et al., 2021	0.90	0.53	1.52	0.75
Albalawi et al., 2021	0.36	0.12	1.15	0.74
Albani et al., 2020	1.26	0.95	1.69	0.80
Alberici et al., 2020	1.70	0.69	4.20	0.28
Alguwaihes et al., 2021	1.70	0.7	4.00	0.30
Allameh et al., 2020	0.91	0.54	1.55	0.74
Almaghlouth et al., 2020	1.29	0.27	6.24	0.12
Al-Salameh et al.,2020	0.77	0.44	1.34	0.77
Altschul et al., 2020	0.95	0.78	1.16	0.85
Anaklı etal., 2021	1.79	0.76	4.22	0.28
Anantharamna et al., 2020	1.27	0.81	2.00	0.70
Angelidi et al., 2021	0.49	0.11	2.09	0.52
Aoun et al., 2021	1.34	0.71	2.54	0.55
Atkins et al., 2020	3.11	2.06	4.71	0.39
Attauabi et al., 2021	1.50	0.39	5.29	0.17
Auld et al., 2020	1.08	0.59	1.95	0.66
Azar et al., 2020	2.41	1.26	4.56	0.30
Baggio et al., 2021	2.82	2.46	3.24	0.79
Baqui et al., 2020	2.54	2.28	2.83	0.83
Barbui et al., 2021	2.65	1.08	6.50	0.14
Barron et al., 2020	9.15	8.91	9.41	0.84
Bartoletti et al., 2020	1.20	0.72	2.83	0.49
Berenguer et al., 2020	2.16	1.84	2.53	0.81
Bertsimas et al., 2020	1.47	1.16	1.85	0.81
Betech et al., 2020	1.22	1.17	1.27	0.87
Bhatt et al., 2021	1.13	1.01	1.26	0.86
Bielza et al., 2021	1.18	0.78	1.78	0.74
Bode et al., 2020	6.10	3.62	20.28	0.02
Bossini et al., 2020	1.61	0.36	6.48	0.12
Botta et al., 2021	1.58	0.93	2.67	0.57
Bottio et al., 2021	5.56	1.11	27.89	0.01
Brandão et al., 2021	1.34	0.91	1.98	0.73
Cabezudo-García et al., 2020	1.54	0.85	2.81	0.52
Caillard et al., 2020	1.86	1.18	2.93	0.57
Caliskan et al., 2020	1.04	0.44	2.42	0.52
Capdevila-Reniu et al., 2021	1.25	0.61	2.67	0.50
Castelnuovo et al., 2020	2.06	1.71	2.49	0.79
Catteau et al., 2020	1.50	1.34	1.68	0.86

Cen et al., 2020	3.10	1.54	6.21	0.18
Chand et al., 2020	1.16	0.74	1.84	0.72
Chang et al., 2020	2.66	1.12	6.34	0.15
Chen et al., 2021	0.97	0.7	1.36	0.81
Chen L et al., 2020	3.24	1.91	5.50	0.27
Chen T et al., 2020	1.47	0.39	5.50	0.16
Chen Y et al., 2020	2.51	1.53	4.13	0.40
Chilimuri et al.,2020	1.58	0.94	2.65	0.58
Choron et al., 2021	0.78	0.35	1.72	0.66
Choudhuri et al., 2020	1.38	1	1.90	0.77
Ciceri et al., 2020	1.71	0.97	3.03	0.50
Cinar et al., 2021	4.24	1.95	9.22	0.09
COVID-ICU group, 2020	1.76	1.53	2.03	0.84
Covino et al., 2020	0.53	0.1	2.78	0.39
Covino et al., 2020	0.67	0.15	2.96	0.37
Covino et al., 2020	0.71	0.19	2.60	0.44
Covino et al., 2021	0.66	0.21	2.03	0.56
Dagher et al., 2021	1.73	0.9	3.32	0.43
Deng et al., 2020	3.54	2.71	4.48	0.57
De Smet et al., 2020	1.16	0.32	4.15	0.25
De Souza et al., 2020	2.33	1.99	2.74	0.80
De Souza et al., 2021	3.93	2.34	6.61	0.21
Deeb et al., 2021	2.38	1.57	3.60	0.51
Du et al., 2020	1.94	0.69	5.45	0.18
Du et al., 2020	1.47	0.56	3.83	0.31
Duarte et al., 2020	1.14	0.73	1.77	0.74
Duclos et al., 2021	0.45	0.11	1.91	0.56
Elamari et al., 2020	1.86	0.53	6.53	0.12
Elhadi et al., 2021	1.31	0.9	1.91	0.74
Escosteguy et al., 2021	1.41	0.87	2.29	0.65
Fadini et al., 2020	1.35	0.71	2.59	0.54
Falcone etal., 2020	1.42	0.76	2.69	0.53
Fava et al., 2020	1.69	0.68	4.21	0.28
Ferrando et al., 2020	1.83	0.42	7.83	0.08
Ferrari et al., 2020	3.32	1.49	7.35	0.13
Foy et al., 2020	6.86	3.59	13.11	0.05
Galiero et al., 2020	1.48	0.94	2.34	0.65
Genet et al., 2020	1.78	0.87	3.64	0.38
Giannoglou et al., 2020	2.11	1.13	3.96	0.37
Goodall et al., 2020	1.51	1.15	1.97	0.78
Grasselli et al., 2020	2.07	1.71	2.51	0.79
Guan et al., 2020	4.27	2.2	8.26	0.12
Guo et al., 2020	3.20	0.81	12.58	0.04
Gupta et al., 2020	1.14	0.91	1.43	0.83
Haase et al., 2020	1.13	0.64	1.98	0.67

Halvatsiotis et al., 2020	2.71	0.91	8.08	0.09
Haq et al., 2020	3.68	1.82	7.43	0.14
Hashemi et al., 2020	1.38	0.68	2.79	0.49
Hatami et al., 2021	1.39	0.91	2.14	0.69
Hendra et al., 2021	2.20	0.64	7.49	0.10
Hu J et al., 2020	0.50	0.35	1.03	0.81
Hwang et al., 2020	3.11	1.24	7.80	0.10
Iaccarino et al., 2020	1.17	0.89	1.55	0.81
Iaccarino et al., 2020	2.76	1.96	3.87	0.54
Imam Z et al., 2020	1.24	0.9	1.70	0.79
Incerti et al., 2021	1.65	1.5	1.81	0.86
Inciardi et al., 2020	3.47	1.02	11.82	0.04
Islam et al., 2020	2.71	1.18	6.24	0.16
Jiang Y et al., 2020	3.63	1.21	10.94	0.05
Jimeno et al., 2020	2.40	1.05	5.80	0.18
Jose et al., 2021	2.09	1.18	3.72	0.41
Jung et al., 2020	1.28	0.79	2.06	0.68
Kaeuffer et al., 2020	1.70	1	2.70	0.58
Karahan etal., 2020	2.40	1.23	4.68	0.29
Kim et al., 2020	1.82	1.25	2.67	0.65
Kim S et al., 2020	2.59	1.32	5.06	0.26
Krause et al., 2020	0.75	0.199	2.86	0.39
Krishnamoorthy et al., 2021	1.77	1.1	2.83	0.58
Krishnan et al., 2020	1.41	0.73	2.74	0.51
Kunal et al., 2020	2.38	0.99	5.69	0.18
Kute et al., 2021	2.03	0.91	4.51	0.27
Laake et al., 2020	2.78	1.53	5.06	0.28
Lee et al., 2021	2.24	1.56	3.22	0.59
Lee L et al., 2020	1.61	1.03	2.43	0.65
Lee Y et al., 2020	2.29	1.44	3.64	0.48
Li et al., 2021	2.30	1.46	3.63	0.48
Li G et al., 2021	1.04	0.39	2.73	0.45
Li K et al., 2020	0.88	0.18	4.34	0.22
Lievre et al., 2020	1.40	1.04	1.89	0.78
Ling et al., 2020	0.86	0.41	1.81	0.65
Liu et al., 2020	3.30	1.24	8.77	0.08
Liu et al., 2020	1.01	0.46	2.21	0.57
Llamas et al., 2021	1.94	1.27	2.95	0.59
Lorente et al., 2021	0.92	0.35	2.40	0.51
Lorente et al., 2021	0.34	0.04	2.99	0.35
Louapre et al., 2020	5.36	1.93	14.95	0.03
Lu Y et al., 2021	0.71	0.18	2.88	0.39
Lucar et al., 2021	5.94	1.45	24.25	0.01
Lunski et al., 2021	0.92	0.7	1.21	0.84
Marouf et al., 2021	3.46	1.18	10.13	0.06

Martinez-Lopez et al., 2020	1.12	0.48	2.63	0.49
Martins et al., 2021	1.69	0.46	6.13	0.13
Mash et al., 2020	1.84	1.24	2.73	0.63
Mato et al., 2020	2.89	1.42	5.90	0.20
Mayer et al., 2021	1.69	1.43	1.99	0.83
Mehta et al., 2021	1.07	1.04	1.10	0.87
Menager et al., 2021	0.38	0.04	3.27	0.31
Mendes et al., 2020	1.79	0.96	3.36	0.44
Moghaddam et al., 2020	2.88	0.39	21.29	0.01
Molinero et al., 2020	2.96	1.75	4.99	0.31
Morrison et al., 2020	0.67	0.27	1.62	0.66
Mostaza et al., 2020	1.20	0.77	1.88	0.72
Murthy et al., 2021	1.60	1.1	2.32	0.69
Nachega et al., 2020	3.53	2.16	5.77	0.27
Nachtigall et al., 2020	1.44	1.04	1.98	0.76
Nakamura et al., 2020	1.59	0.29	8.87	0.06
Namendys-Silva, 2021	3.03	1.51	6.09	0.19
Nepogodiev et al., 2020	1.15	0.84	1.57	0.80
Ng et al., 2020	1.16	1.02	1.32	0.86
Nie et al., 2020	3.88	0.65	22.96	0.01
Nikpouraghdam et al., 2020	1.24	0.65	2.34	0.58
Okoh et al., 2020	1.19	0.78	1.83	0.73
Otero et al., 2021	1.12	0.42	2.93	0.42
Oto et al., 2021	1.37	0.39	4.82	0.20
Ozdemir et al., 2021	2.36	1.23	4.53	0.30
Ozturk et al., 2020	1.94	1.4	2.70	0.68
Pascual et al., 2020	0.36	0.07	1.88	0.56
Park et al., 2020	2.43	1.51	3.90	0.44
Park et al., 2020	2.56	1.47	4.46	0.34
Parlak et al., 2021	0.43	0.08	2.25	0.48
Passamonti et al., 2020	1.26	0.76	2.09	0.67
Patel et al., 2020	1.29	0.85	1.97	0.72
Peiro et al., 2021	2.07	0.95	4.49	0.28
Pineiro et al., 2021	3.09	0.72	13.32	0.03
Plotnikow et al., 2020	1.35	0.23	8.03	0.08
Pongpirul et al., 2020	11.74	3.88	35.50	0.01
Qin W et al., 2021	1.08	0.35	3.35	0.34
Qin W et al., 2021	1.49	0.77	2.89	0.49
Ramaswamy et al., 2020	2.02	0.69	5.91	0.15
Ramos-Rincon et al., 2020	1.09	0.92	1.30	0.85
Rastad et al., 2021	1.89	0.73	4.90	0.22
Rajter et al., 2021	1.17	0.39	3.55	0.32
Recinella et al., 2020	0.57	0.22	1.50	0.68
Rivera-Izquierdo et al.,2020	4.83	2.5	9.30	0.10
Rodriguez et al., 2020	1.50	0.23	9.80	0.05

Rokni et al., 2020	1.47	0.55	3.92	0.29
Rosenthal et al., 2020	1.20	1.12	1.28	0.87
Rossi et al., 2020	1.34	0.91	1.96	0.73
Rottoli et al., 2020	1.00	0.48	2.05	0.61
Russo et al., 2020	1.34	0.82	2.19	0.66
Saha et al., 2021	1.37	0.74	2.53	0.56
Sai et al., 2021	1.11	0.32	3.90	0.27
Saifi et al., 2021	1.85	0.27	12.76	0.03
Salazer et al., 2021	1.32	1.12	1.56	0.85
Sands et al., 2020	1.57	1.21	2.03	0.78
Santos et al., 2020	33.00	3.46	314.55	0.00
Shahriarirad et al., 2020	1.84	0.35	9.75	0.05
Sharma, 2020	1.19	1.08	1.66	0.83
Shi et al., 2020	2.18	1.13	4.17	0.34
Shields et al., 2020	7.50	1.67	32.66	0.01
Smith et al., 2020	1.96	1.25	3.08	0.55
Stefan et al., 2020	0.69	0.11	4.18	0.23
Sun et al., 2020	1.18	0.64	2.20	0.61
Talavera et al., 2020	1.33	0.76	2.34	0.61
Tehrani et al., 2021	1.35	0.68	2.66	0.52
Tehrani et al., 2021	3.30	1.54	7.10	0.14
Tessitore et al., 2021	1.60	0.91	2.60	0.58
Tu et al., 2020	8.72	2.27	33.45	0.01
Turgutalp et al., 2021	1.01	0.65	1.58	0.76
Venturini et al., 2021	1.33	0.48	3.71	0.31
Vrillon et al., 2021	1.49	0.52	4.29	0.25
Vu et al., 2020	1.63	0.35	7.50	0.09
Wang et al., 2020	4.88	1.31	18.18	0.02
Wang et al., 2020	1.09	0.52	2.26	0.57
Wang Junhong et al., 2020	2.58	1.04	6.41	0.15
Webb et al., 2020	1.83	0.57	5.87	0.15
Xu Jingyuan et al., 2020	1.05	0.55	1.98	0.65
Xu Jiqian et al., 2020	0.80	0.41	1.58	0.71
Xu Peng et al., 2020	7.11	3.3	15.31	0.03
Y.Wang et al., 2020	3.28	1.2	8.98	0.08
Yan et al., 2020	2.83	1.42	5.62	0.22
Yang C. et al., 2021	1.65	0.73	3.75	0.34
Yang K. et al., 2020	0.41	0.09	1.81	0.58
Yao et al., 2021	2.98	1.03	8.64	0.08
Yarza et al., 2020	1.18	0.34	3.90	0.27
Yildirim et al., 2021	0.34	0.04	2.84	0.37
Yu C, 2020	2.52	1.62	3.94	0.45
Zhao Y et al., 2020	2.79	1.59	4.90	0.30
Zhou S et al., 2020	3.18	1.48	6.84	0.15
Zhou Wei et al., 2021	1.21	0.6	2.42	0.56

Zhu Y et al., 2021	0.93	0.58	1.49	0.76
Zou X et al., 2020	1.44	0.69	3.04	0.45
Zuo P et al., 2020	0.98	0.96	1.00	0.87
**Total (I squared=95.5%, p<0.01)**	**1.56**	**1.45**	**1.67**	**100.00**
