# Supplementary material for: Heterogeneity and Risk of Bias in Studies Examining Risk Factors for Severe Illness and Death in COVID-19: A Systematic Review and Meta-Analysis
Source: Pathogens. 2022 May 10;11(5):563. doi: 10.3390/pathogens11050563 (PMC9147100; doi:10.3390/pathogens11050563)
Supplement: Supplementary file 1 [file pathogens-11-00563-s001.zip › Table S9.pdf]

Heterogeneity and risk of bias in studies examining risk factors for severe illness and death in COVID-19: a systematic review and meta-analysis

Abraham Degarege *, Zaeema Naveed, Josiane Kabayundo, David Brett-Major

Table S9: Forest plot showing the relationship of diabetes with the odds of severe illness among COVID-19 patients

Author, year	OR	LCI	UCI
Abbas et al., 2020	1.97	1.09	3.53
Aiswarya et al., 2020	1.3	0.41	4.15
Allard et al., 2020	1.38	0.61	3.13
Alqahtani et al., 2020	18.45	9.28	36.69
Al-Samkari et al., 2020	1.98	1.28	3.06
Alsharidah etal., 2021	0.95	0.62	1.48
Basaran et al., 2021	3.58	1.44	8.89
Basaran et al., 2021	3.57	1.43	8.89
Bauer et al., 2020	1.47	1.06	2.04
Betti et al., 2021	0.71	0.19	2.61
Bhargava et al., 2020	2.91	1.62	5.25
Bolouri et al., 2021	2.11	0.71	6.27
Bravi et al., 2020	1.52	1.05	2.18
Cabello et al., 2021	6.14	1.01	37.21
Cai et al., 2020	4.28	1.78	10.28
Cekerevac et al., 2021	1.39	0.61	3.19
Cen et al., 2020	3.8	2.51	5.74
Chen at al., 2020	2.63	0.86	8.05
Chen Z et al., 2020	1.06	0.7	1.59
Cheng et al., 2020	1.74	0.58	5.02
Cizmecioglu et al., 2020	1.25	0.27	5.76
Colaneri et al., 2020	0.21	0.02	2
Deng et al., 2020	2.11	0.7	6.35
Du et al., 2020	1.43	0.63	3.22
Du et al., 2020	1.43	0.63	3.22
Duan et al., 2020	3.93	0.79	19.58
Feng et al., 2020	1.58	0.84	2.97
Gao et al., 2020	16.53	2.07	132.2
Ghaffari et al., 2021	1.78	1.07	2.98
Gostner et all., 2020	6.46	0.68	61.16
Guan et al., 2020	3.16	2.14	4.67
Hu et al., 2021	4.78	1.16	19.75
Hu et al.,2020	2.32	1.19	4.53
Huang et al., 2020	8.14	2.84	23.34
Jain et al., 2021	1.29	0.35	4.78
Ji et al., 2021	0.74	0.11	4.86
Jinag N et al., 2021	3.31	1.12	9.75
Kaeuffer et al., 2020	1.2	0.91	1.59

Karahan etal., 2020	2.69	1.25	5.77
Khan et al., 2020	2.37	1.28	4.37
Lara et al., 2020	1.66	0.61	4.47
Lee Y et al., 2020	2.26	1.43	3.58
Li et al., 2020	1.91	1.18	3.1
Li T et al., 2020	1.64	1.02	2.65
Liu J etal., 2021	4.04	0.87	18.81
Liu S et al., 2020	1.64	0.52	5.22
Molinero et al., 2020	1.7	1.07	2.74
Nachega et al., 2020	5.15	3.36	7.89
Nguyen et al., 2020	1.77	1.03	3.03
Pei et al., 2020	2.63	1.49	4.63
Pereira etal., 2020	1.43	0.58	3.54
Qin et al., 2020	1.48	0.86	2.55
Ren et al., 2020	1.89	0.65	5.48
Sadeghi-Haddad-Zavareh et al., 2020	1.02	0.67	1.57
Shahriarirad et al., 2020	2.57	0.6	10.93
Shi et al., 2020	3.15	1.27	7.81
Siepmann et al., 2020	3.68	1.005	13.47
Song et al, 2020	2.7	0.33	21.83
Stefan et al., 2020	1.43	0.36	5.61
Sun et al., 2020	1.89	0.28	12.65
Tao et al., 2020	7.44	2.4	23.11
Tessitore et al., 2021	2.9	1.7	5
Tian et al., 2020	0.81	0.43	1.51
To et al., 2020	1.37	0.15	11.93
Vee et al., 2020	56	15.5	202.31
Villa et al., 2021	0.75	0.12	4.66
Vizcarra et al., 2020	0.44	0.05	4.09
Wan et al., 2020	8.9	2.26	34.9
Wang et al., 2021	2.13	0.59	7.68
Wang Junhong et al., 2020	1.22	0.58	2.53
Wang Sijiao et al., 2020	6.94	1.65	29.22
Wang Wen et al., 2021	3.87	1	14.93
Wang Wenjun et al., 2021	1.33	1.04	1.71
Wang X. et al., 2020	3.36	1.38	8.15
Wei et al., 2020	1.01	0.47	2.15
Wei Z et al, 2021	2.85	1.35	6.01
Xie Y et al., 2020	8.33	1.99	34.87
Xiong et al., 2020	0.61	0.21	1.76
Xiong S et al., 2020	1.66	0.61	4.48
Yan et al., 2020	4.77	2.15	10.56
Yang Q et al., 2020	4.04	1.51	10.86
Yu et al. 2020	1.1	0.3	3.6
Yu Y et al., 2020	1.95	0.71	5.36

Zachariah et al., 2020	11.43	0.91	143.64
Zhang Hongmei et al., 2020	1.68	0.26	25.86
Zhang Hongyan et al., 2020	4.01	1.36	11.86
Zhang Jing et al., 2020	1.39	0.9	2.15
Zhang Lijuan et al., 2020	1.18	0.37	3.73
Zhang Nannan et al., 2021	1.24	0.13	11.75
Zhang W. et al., 2021	2.04	1.28	3.28
Zhang Y. et al., 2021	1.74	0.85	3.58
Zhang, 2020	17.6	4.84	63.98
Zhao J. et al., 2021	4.26	2.44	7.48
Zheng B. et al., 2021	3.25	0.87	12.18
Zhou Jun et al., 2021	2.1	0.7	6.68
Zhou Yiwu et al., 2020	1.73	0.28	8.62
**Total (I squared=17.4%, p=0.079)**	**1.51**	**1.36**	**1.67**
