# Supplementary material for: Heterogeneity and Risk of Bias in Studies Examining Risk Factors for Severe Illness and Death in COVID-19: A Systematic Review and Meta-Analysis
Source: Pathogens. 2022 May 10;11(5):563. doi: 10.3390/pathogens11050563 (PMC9147100; doi:10.3390/pathogens11050563)
Supplement: Supplementary file 1 [file pathogens-11-00563-s001.zip › Figure S7.pdf]

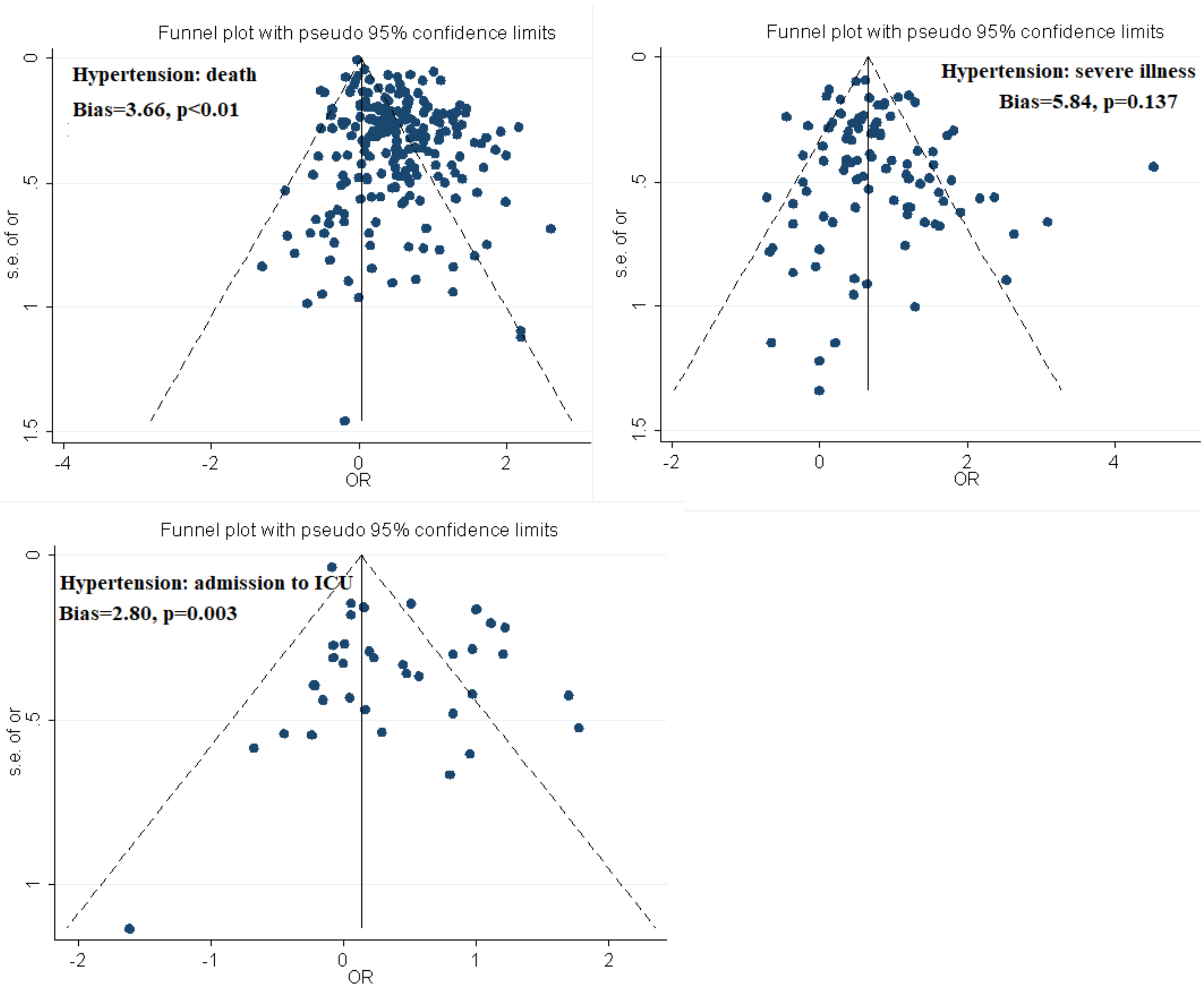

Funnel plot with pseudo 95% confidence limits
Hypertension: death
Bias=3.66, p<0.01
s.e. of or
OR
Funnel plot with pseudo 95% confidence limits
Hypertension: severe illness
Bias=5.84, p=0.137
s.e. of or
OR
Funnel plot with pseudo 95% confidence limits
Hypertension: admission to ICU
Bias=2.80, p=0.003
s.e. of or
OR

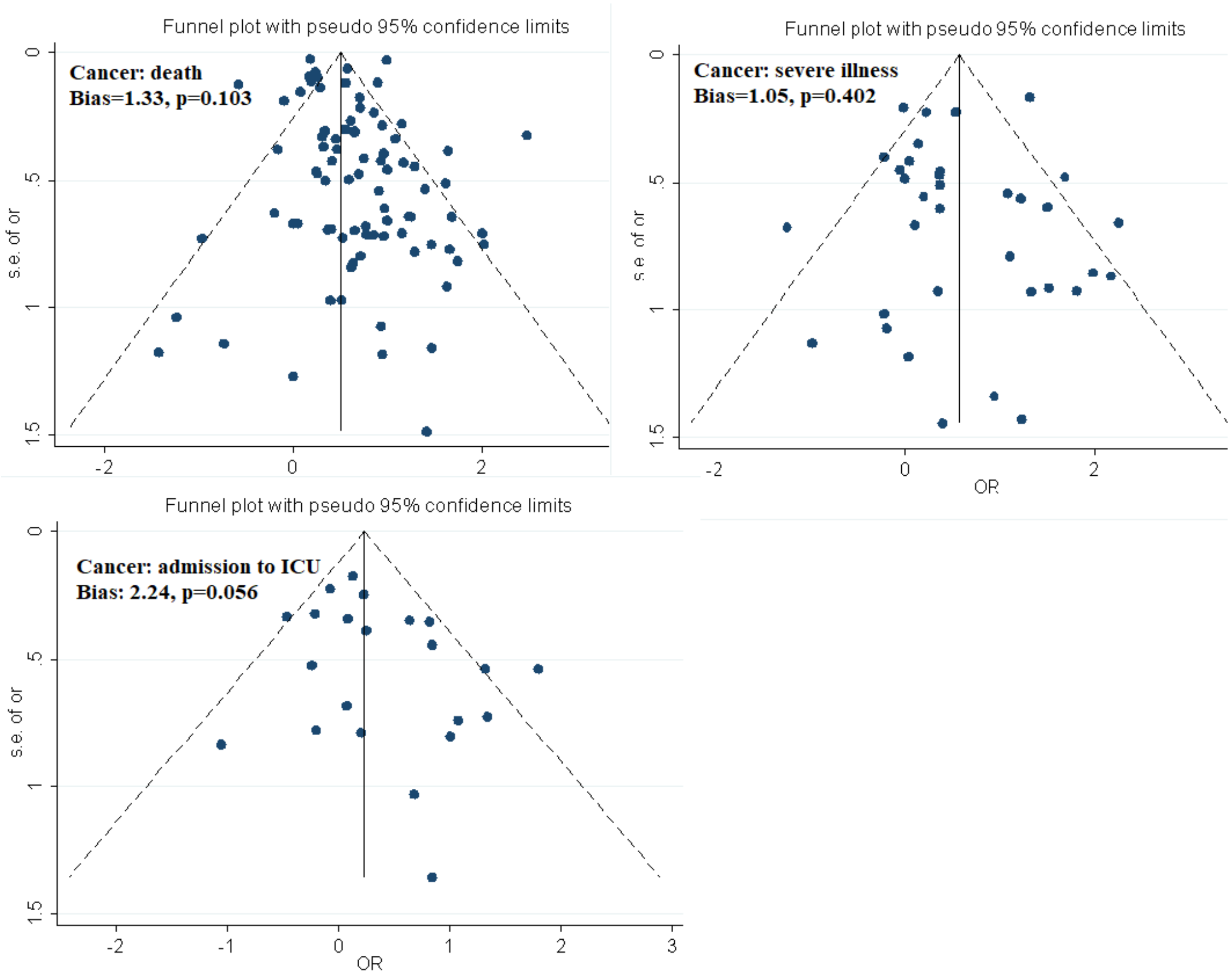
Funnel plot with pseudo 95% confidence limits
Cancer: death
Bias=1.33, p=0.103
s.e. of or
0
.5
1
1.5
-2
0
2
Funnel plot with pseudo 95% confidence limits
Cancer: severe illness
Bias=1.05, p=0.402
s.e. of or
0
.5
1
1.5
-2
0
2
OR
Funnel plot with pseudo 95% confidence limits
Cancer: admission to ICU
Bias: 2.24, p=0.056
s.e. of or
0
.5
1
1.5
-2
-1
0
1
2
3
OR

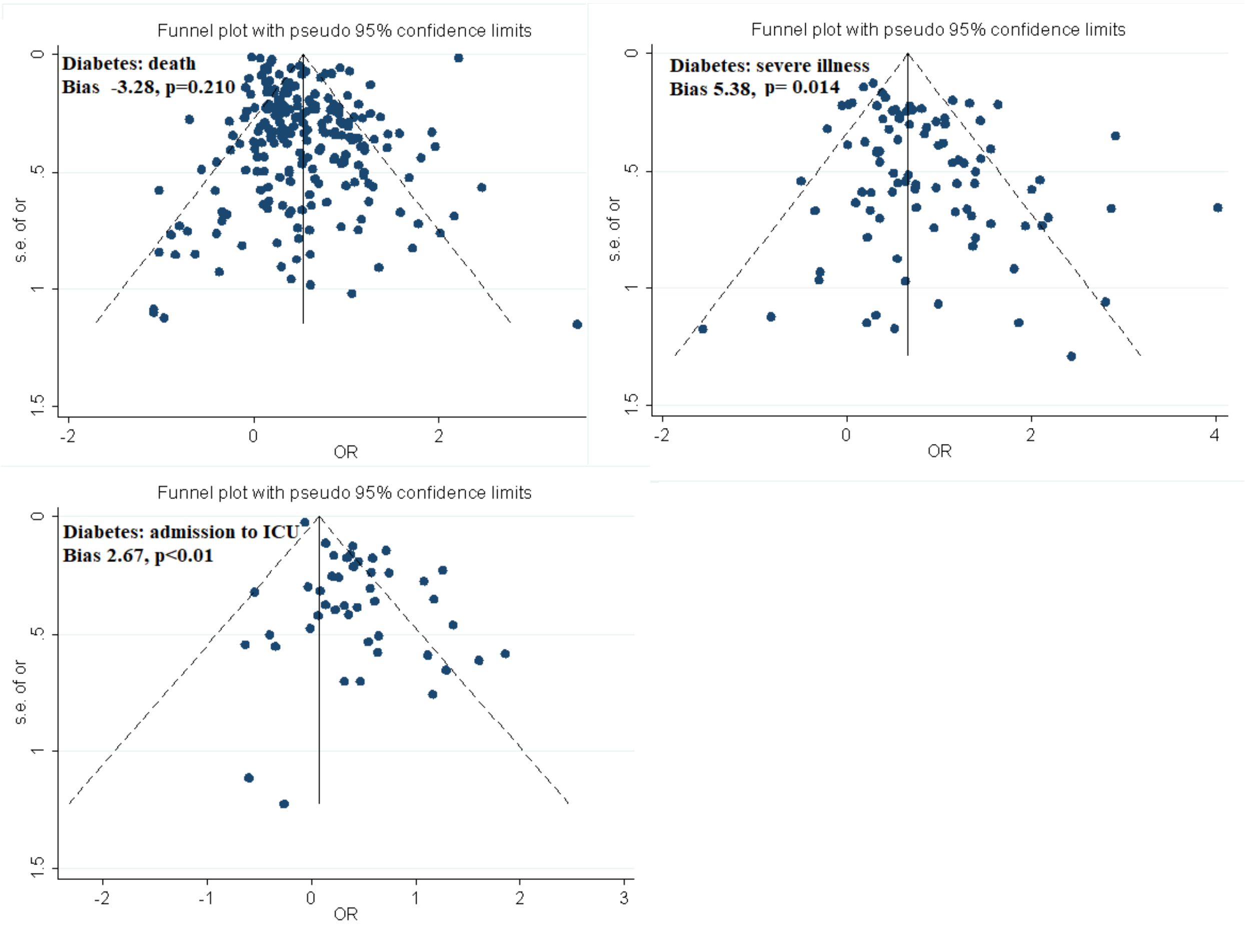
Funnel plot with pseudo 95% confidence limits
Diabetes: death
Bias -3.28, p=0.210
s.e. of or
OR
Funnel plot with pseudo 95% confidence limits
Diabetes: severe illness
Bias 5.38, p= 0.014
s.e. of or
OR
Funnel plot with pseudo 95% confidence limits
Diabetes: admission to ICU
Bias 2.67, p<0.01
s.e. of or
OR

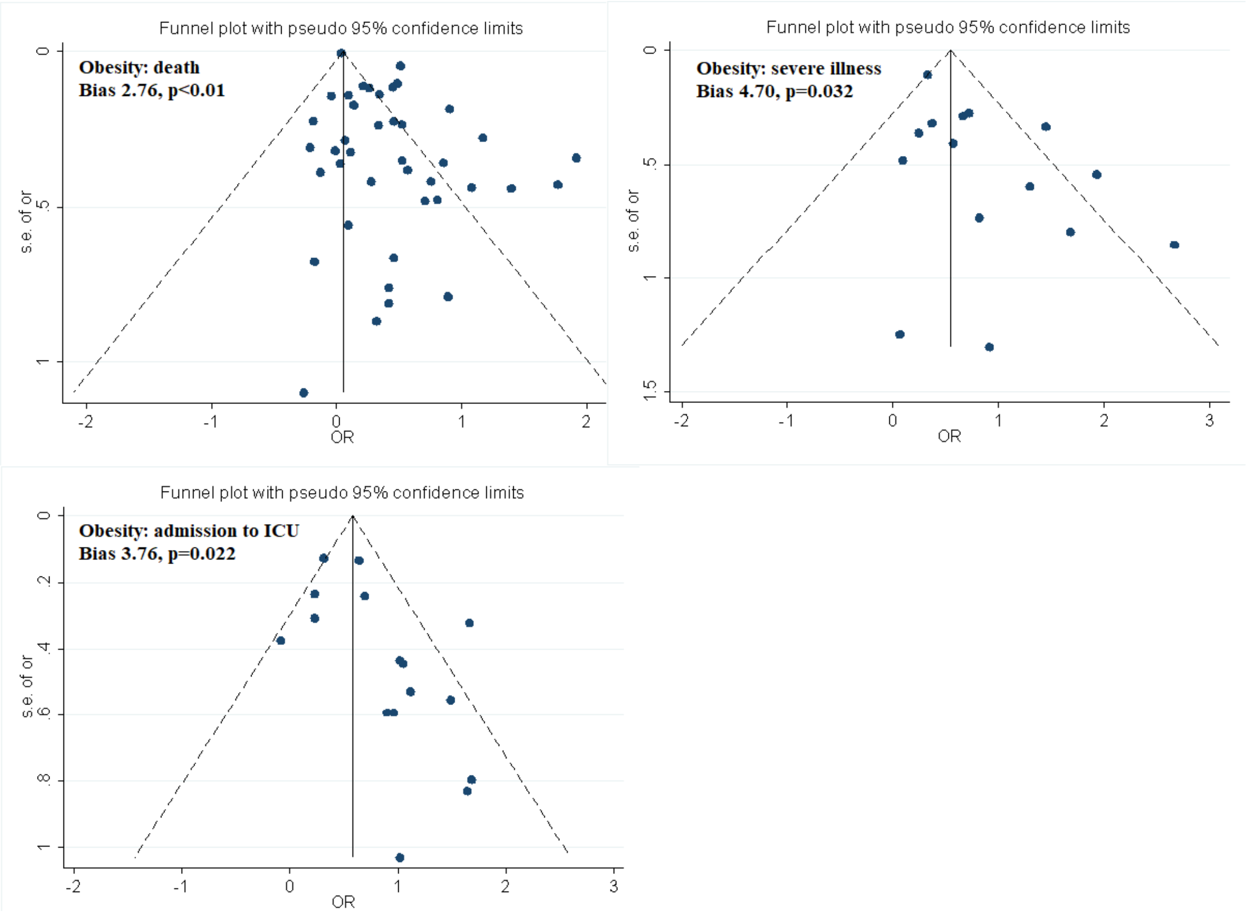

Funnel plot with pseudo 95% confidence limits
Obesity: death
Bias 2.76, p<0.01
s.e. of or
0
.5
1
-2
-1
0
1
2
OR
Funnel plot with pseudo 95% confidence limits
Obesity: severe illness
Bias 4.70, p=0.032
s.e. of or
0
.5
1
1.5
-2
-1
0
1
2
3
OR
Funnel plot with pseudo 95% confidence limits
Obesity: admission to ICU
Bias 3.76, p=0.022
s.e. of or
0
.2
.4
.6
.8
1
-2
-1
0
1
2
3
OR

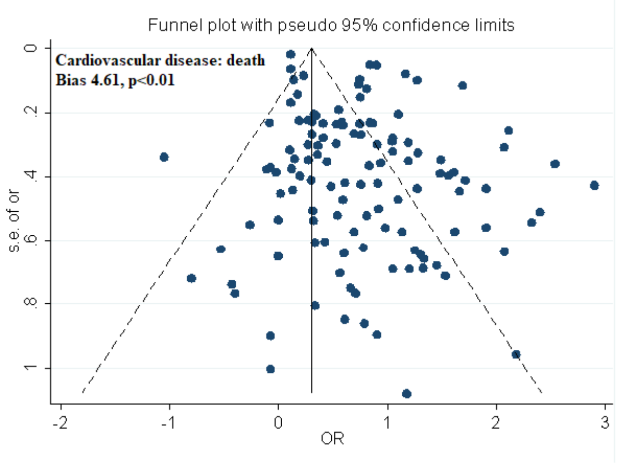
Funnel plot with pseudo 95% confidence limits
Cardiovascular disease: death
Bias 4.61, p<0.01
s.e. of or
OR

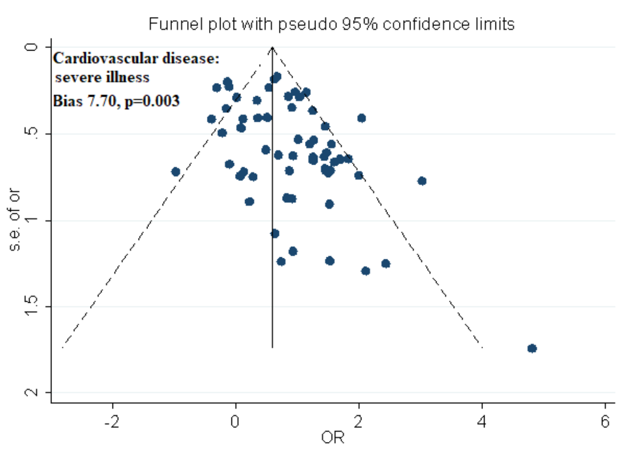
Funnel plot with pseudo 95% confidence limits
Cardiovascular disease:
severe illness
Bias 7.70, p=0.003
s.e. of or
OR

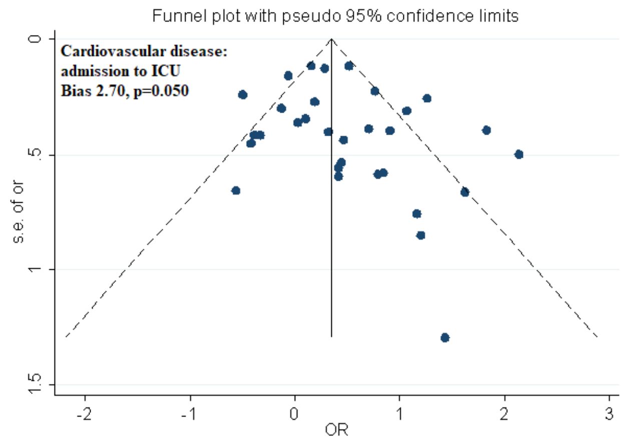
Funnel plot with pseudo 95% confidence limits
Cardiovascular disease:
admission to ICU
Bias 2.70, p=0.050
s.e. of or
OR

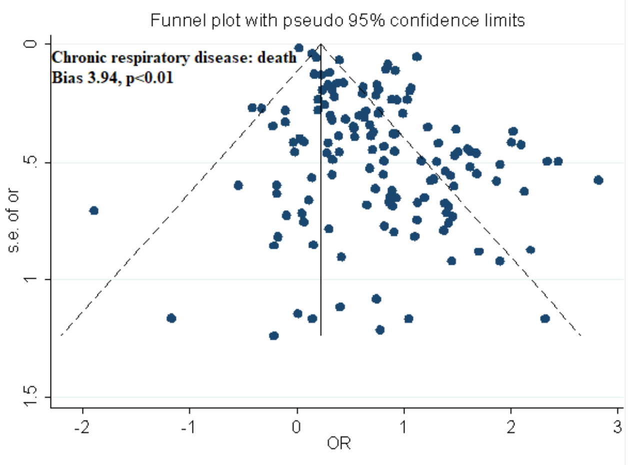
Funnel plot with pseudo 95% confidence limits
Chronic respiratory disease: death
Bias 3.94, p<0.01
s.e. of or
OR
0
.5
1
1.5
-2
-1
0
1
2
3

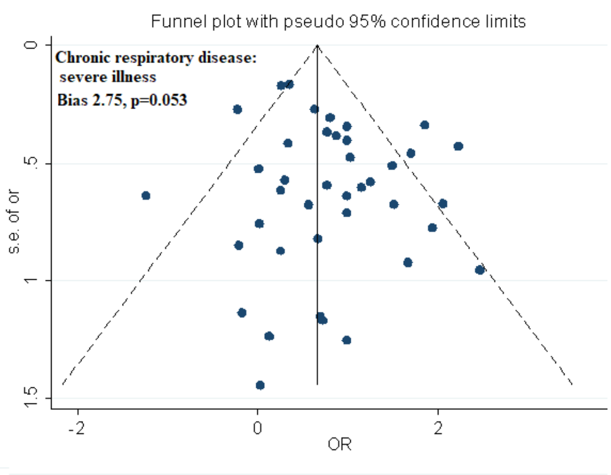
Funnel plot with pseudo 95% confidence limits
Chronic respiratory disease:
severe illness
Bias 2.75, p=0.053
s.e. of or
OR
0
.5
1
1.5
-2
0
2

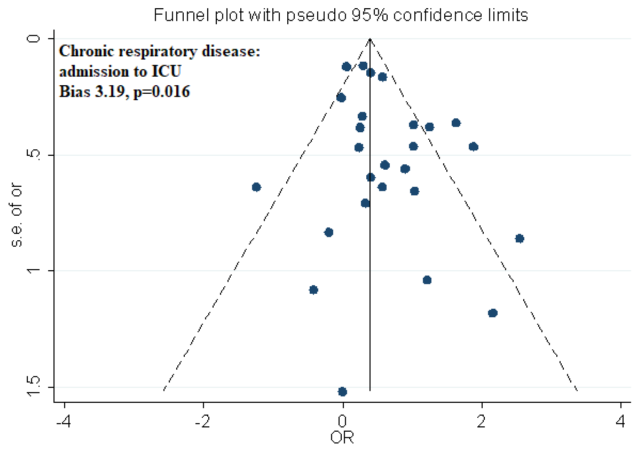
Funnel plot with pseudo 95% confidence limits
Chronic respiratory disease:
admission to ICU
Bias 3.19, p=0.016
s.e. of or
OR
0
.5
1
1.5
-4
-2
0
2
4

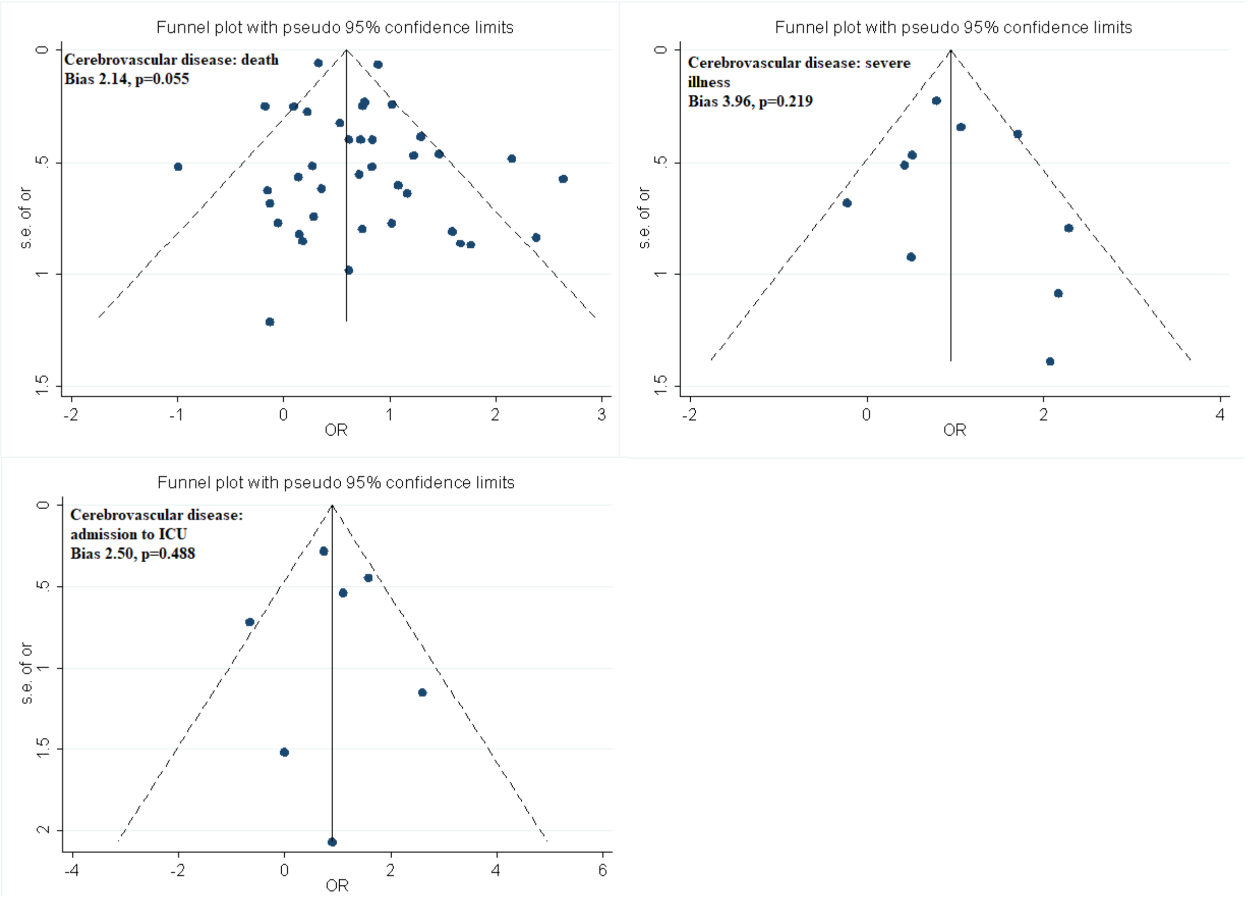
Funnel plot with pseudo 95% confidence limits
Cerebrovascular disease: death
Bias 2.14, p=0.055
s.e. of or
OR
Funnel plot with pseudo 95% confidence limits
Cerebrovascular disease: severe illness
Bias 3.96, p=0.219
s.e. of or
OR
Funnel plot with pseudo 95% confidence limits
Cerebrovascular disease: admission to ICU
Bias 2.50, p=0.488
s.e. of or
OR

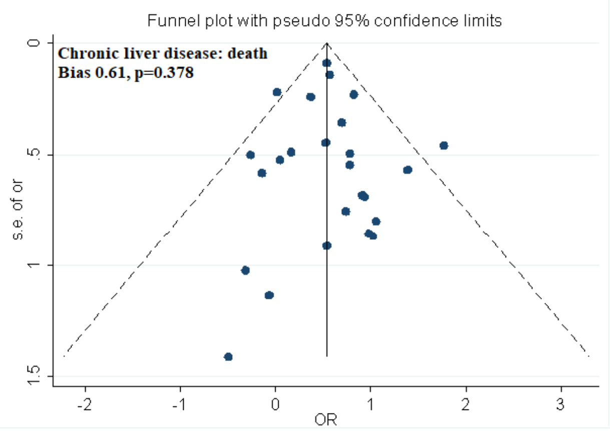
Funnel plot with pseudo 95% confidence limits
Chronic liver disease: death
Bias 0.61, p=0.378
s.e. of or
0
.5
1
1.5
-2
-1
0
1
2
3
OR

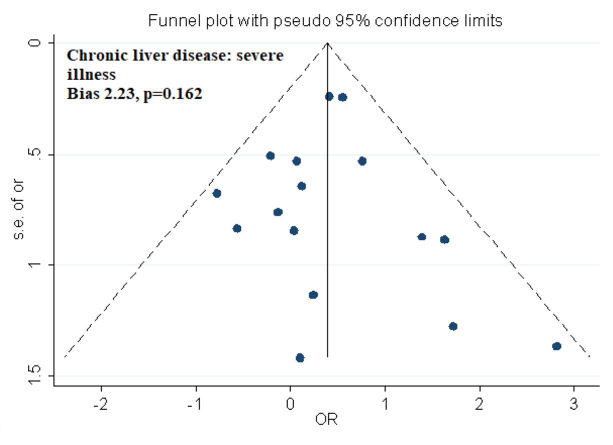
Funnel plot with pseudo 95% confidence limits
Chronic liver disease: severe illness
Bias 2.23, p=0.162
s.e. of or
0
.5
1
1.5
-2
-1
0
1
2
3
OR

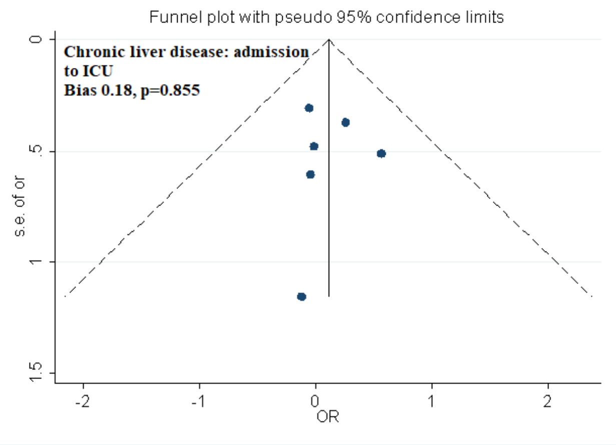
Funnel plot with pseudo 95% confidence limits
Chronic liver disease: admission to ICU
Bias 0.18, p=0.855
s.e. of or
0
.5
1
1.5
-2
-1
0
1
2
OR

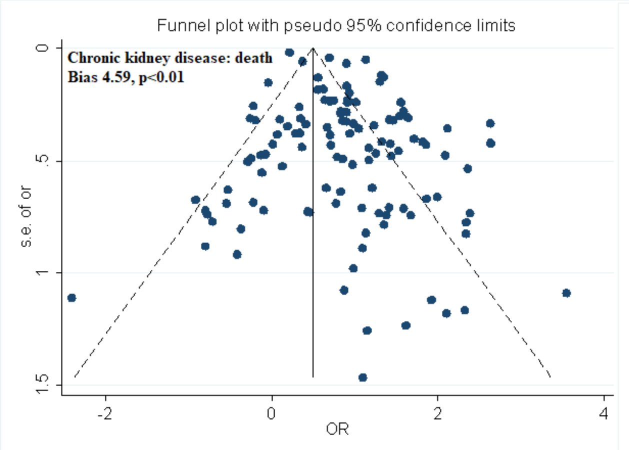
Funnel plot with pseudo 95% confidence limits
Chronic kidney disease: death
Bias 4.59, p<0.01
s.e. of or
OR

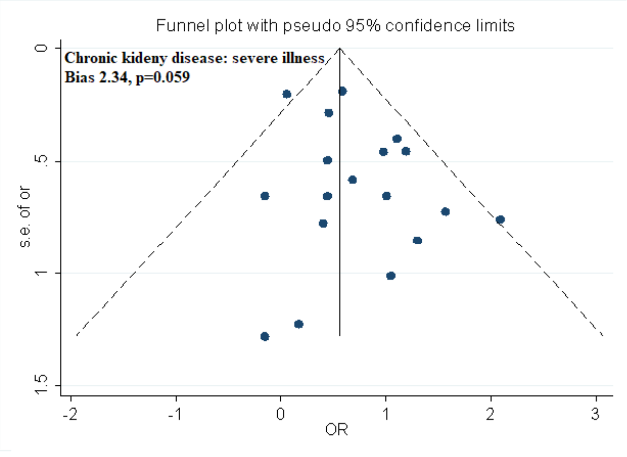
Funnel plot with pseudo 95% confidence limits
Chronic kideny disease: severe illness
Bias 2.34, p=0.059
s.e. of or
OR

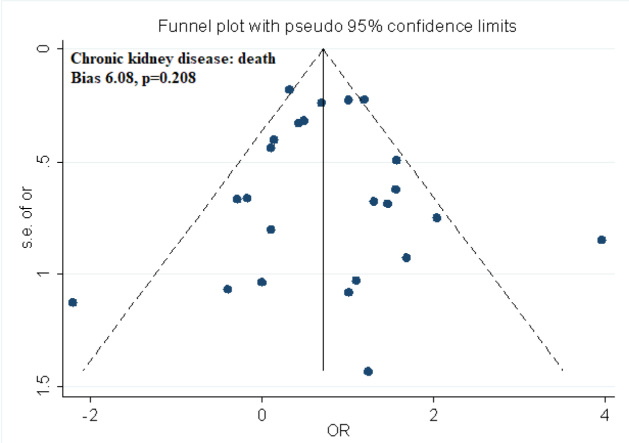
Funnel plot with pseudo 95% confidence limits
Chronic kidney disease: death
Bias 6.08, p=0.208
s.e. of or
OR

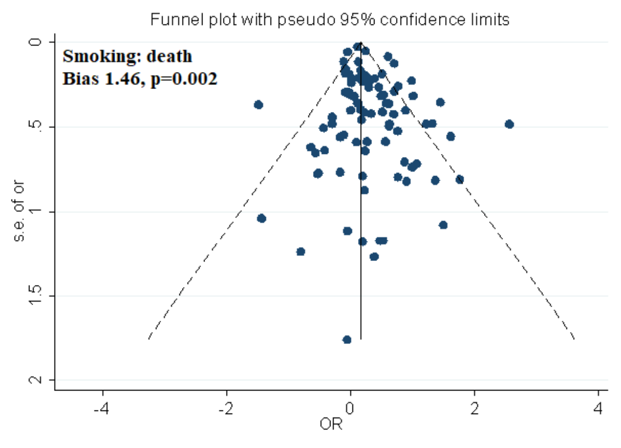
Funnel plot with pseudo 95% confidence limits
Smoking: death
Bias 1.46, p=0.002
s.e. of or
OR

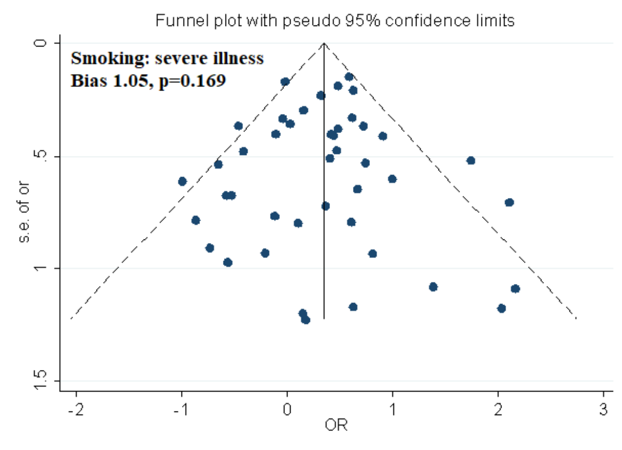
Funnel plot with pseudo 95% confidence limits
Smoking: severe illness
Bias 1.05, p=0.169
s.e. of or
OR

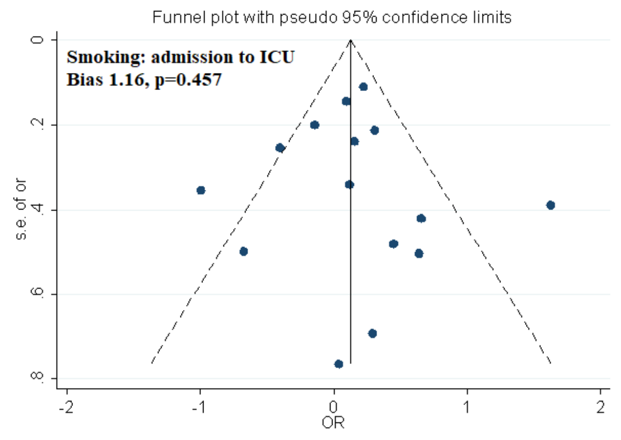
Funnel plot with pseudo 95% confidence limits
Smoking: admission to ICU
Bias 1.16, p=0.457
s.e. of or
OR

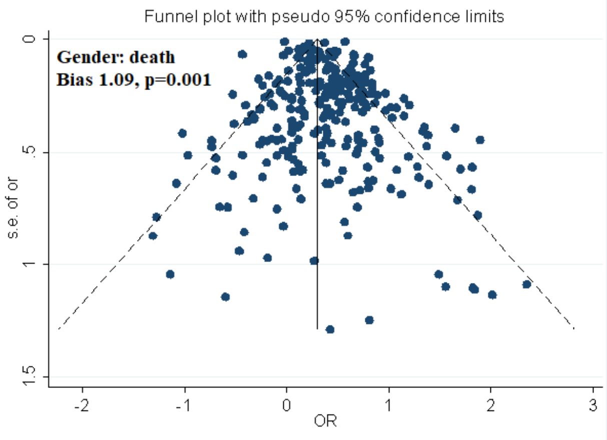
Funnel plot with pseudo 95% confidence limits
Gender: death
Bias 1.09, p=0.001
s.e. of or
OR
0
.5
1
1.5
-2
-1
0
1
2
3

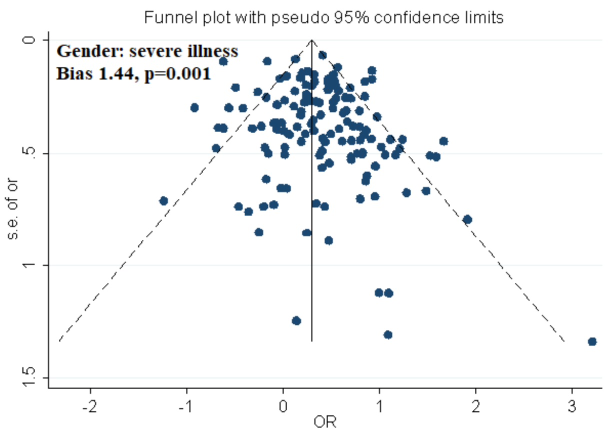
Funnel plot with pseudo 95% confidence limits
Gender: severe illness
Bias 1.44, p=0.001
s.e. of or
OR
0
.5
1
1.5
-2
-1
0
1
2
3

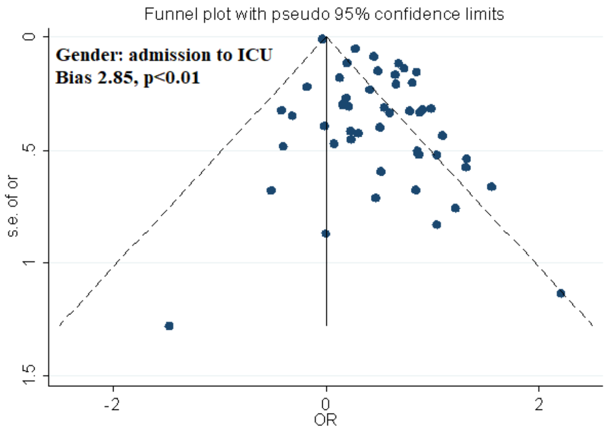
Funnel plot with pseudo 95% confidence limits
Gender: admission to ICU
Bias 2.85, p<0.01
s.e. of or
OR
0
.5
1
1.5
-2
0
2

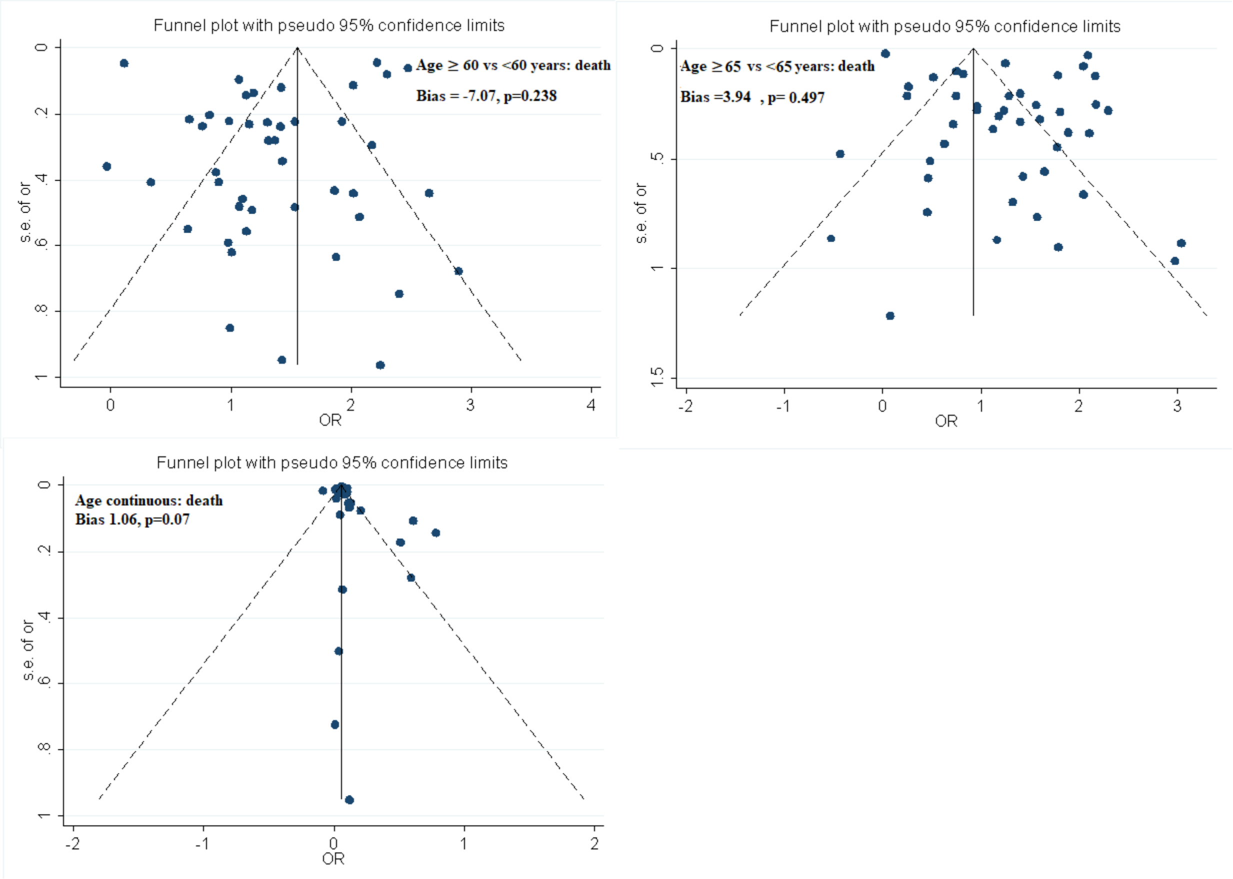

Funnel plot with pseudo 95% confidence limits
Age ≥ 60 vs <60 years: death
Bias = -7.07, p=0.238
s.e. of or
OR
Funnel plot with pseudo 95% confidence limits
Age ≥ 65 vs <65 years: death
Bias =3.94 , p= 0.497
s.e. of or
OR
Funnel plot with pseudo 95% confidence limits
Age continuous: death
Bias 1.06, p=0.07
s.e. of or
OR

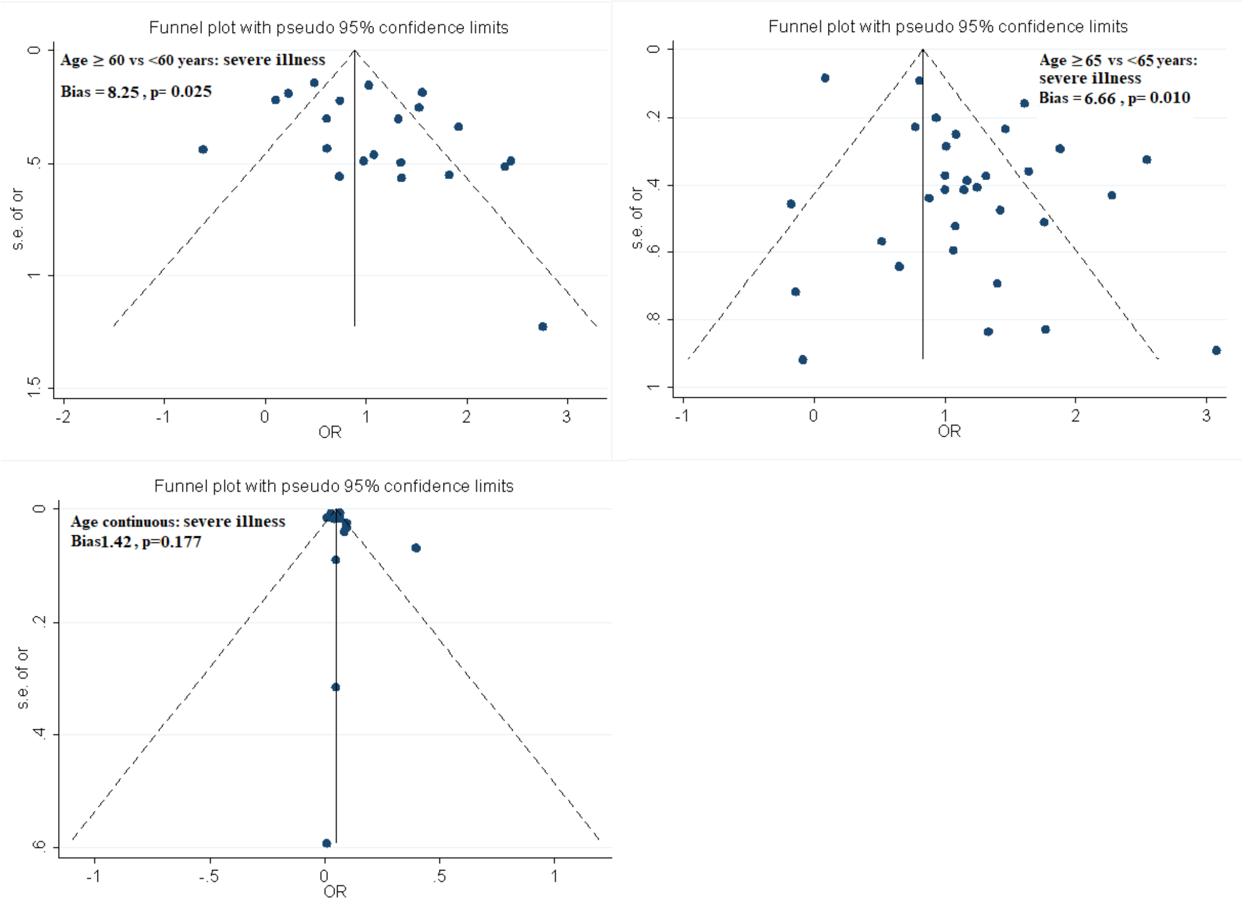
Funnel plot with pseudo 95% confidence limits
Age ≥ 60 vs <60 years: severe illness
Bias = 8.25 , p= 0.025
s.e. of or
OR
Funnel plot with pseudo 95% confidence limits
Age ≥ 65 vs <65 years: severe illness
Bias = 6.66 , p= 0.010
s.e. of or
OR
Funnel plot with pseudo 95% confidence limits
Age continuous: severe illness
Bias1.42 , p=0.177
s.e. of or
OR

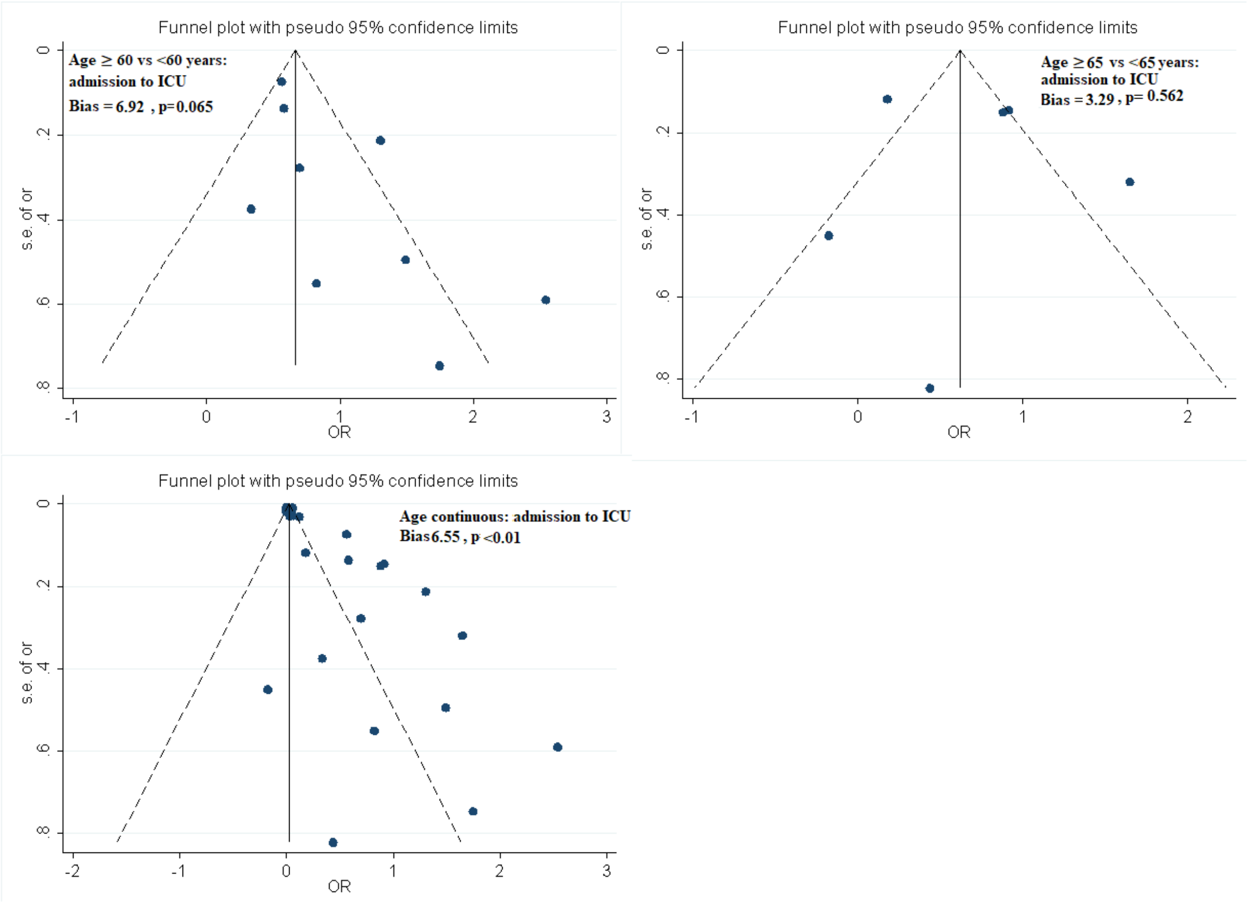

Figure S7: Funnel plots showing the odds ratio of death, severe illness, and admission to the ICU against the standard errors based on comorbidity and age
